# Supplementary material for: Geometric entropy of plant leaves: A measure of morphological complexity
Source: PLoS One. 2024 Jan 2;19(1):e0293596. doi: 10.1371/journal.pone.0293596 (PMC10760904; doi:10.1371/journal.pone.0293596)
Supplement: S1 Data — (ZIP) [file pone.0293596.s001.zip › S1_data/README.docx]

**S1 Data.** Scanned images of plant leaves collected from Trivandrum, Kerala, India (112 RGB JPG images).
