# Supplementary figures and images for: Geometric entropy of plant leaves: A measure of morphological complexity (part 2 of 2)
Source: PLoS One. 2024 Jan 2;19(1):e0293596. doi: 10.1371/journal.pone.0293596 (PMC10760904; doi:10.1371/journal.pone.0293596)

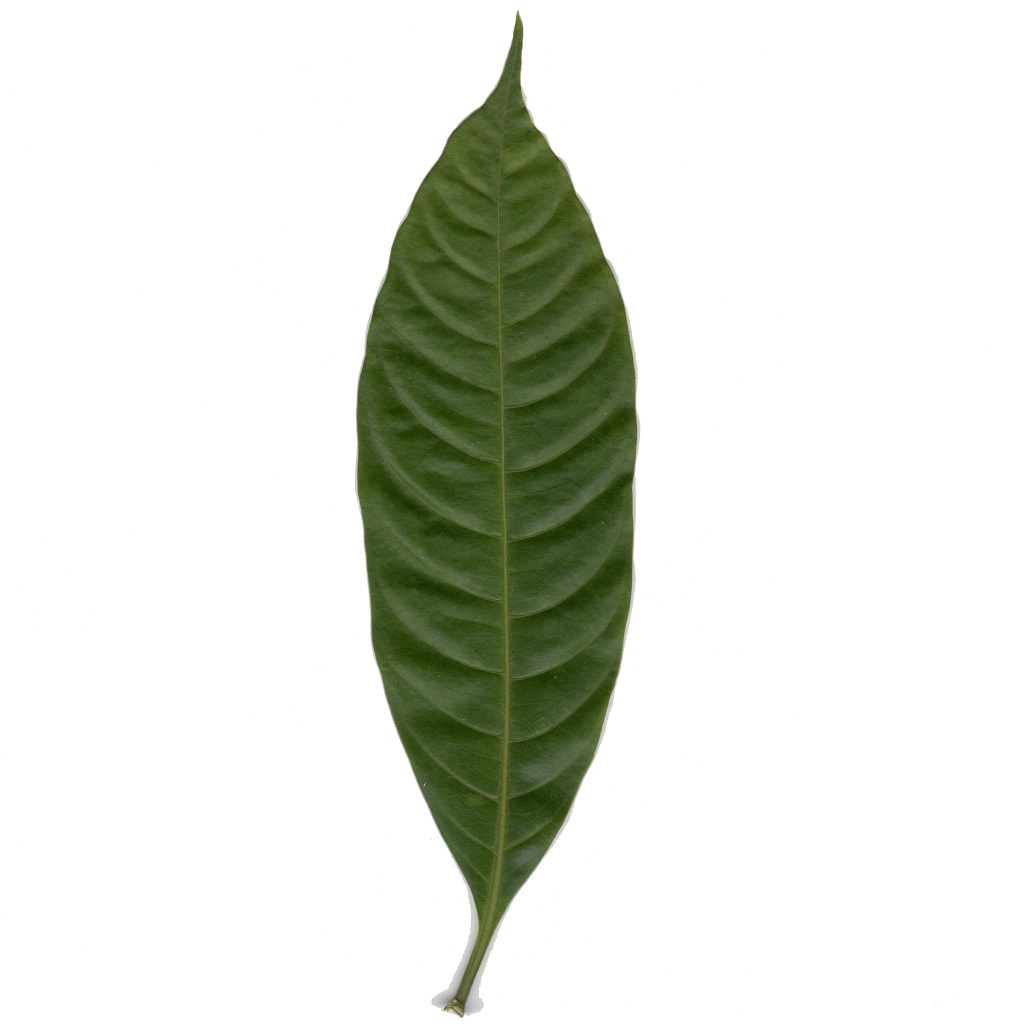

Supplement: S1 Data — (ZIP) [file pone.0293596.s001.zip › S1_data/Tabernaemontana alternifolia.jpg]

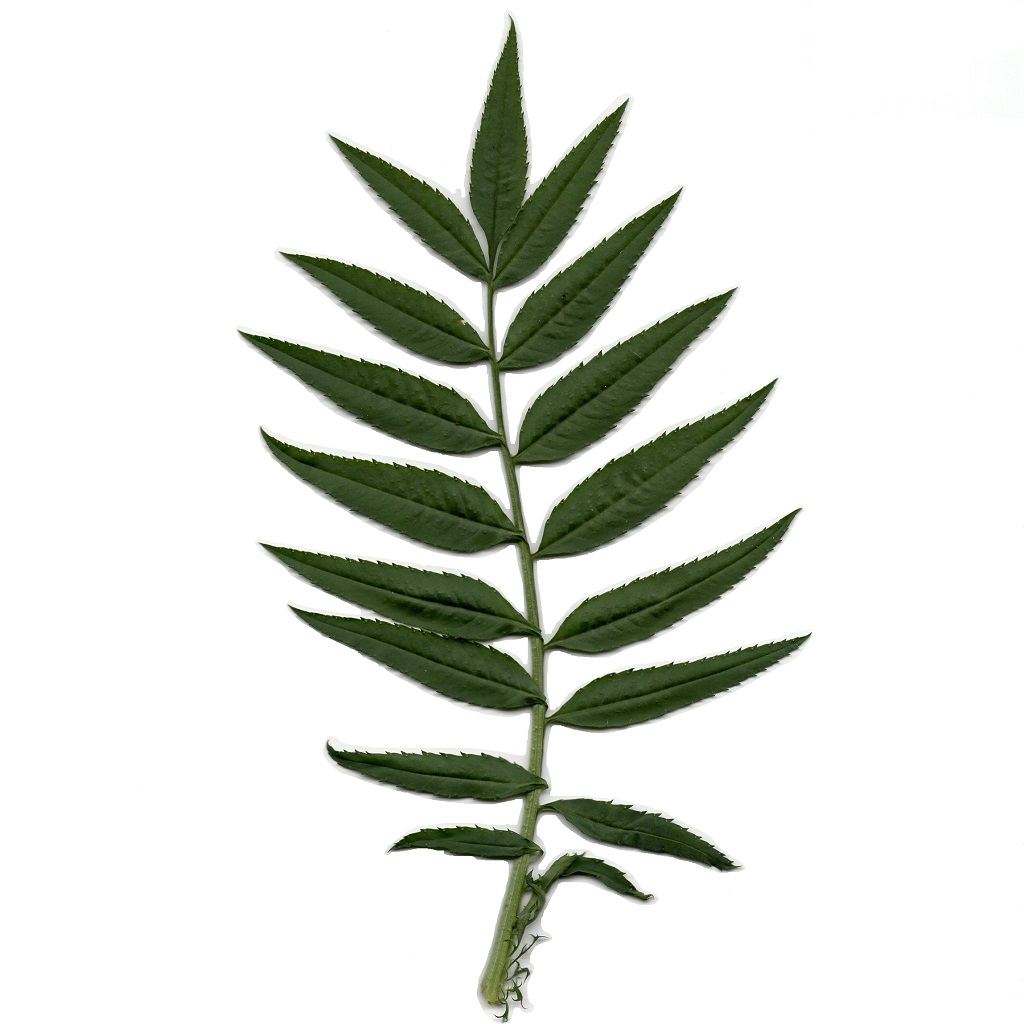

Supplement: S1 Data — (ZIP) [file pone.0293596.s001.zip › S1_data/Tagetes erecta.jpg]

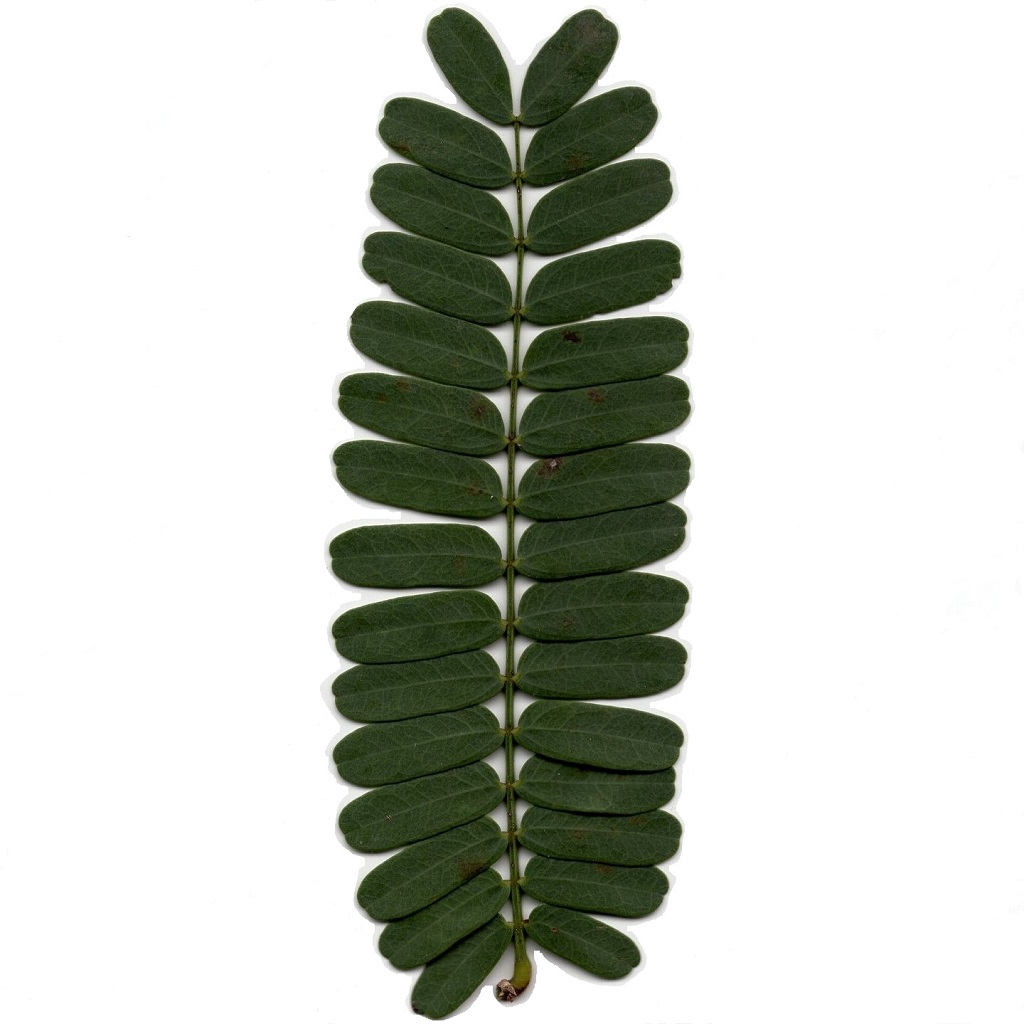

Supplement: S1 Data — (ZIP) [file pone.0293596.s001.zip › S1_data/Tamarindus indica.jpg]

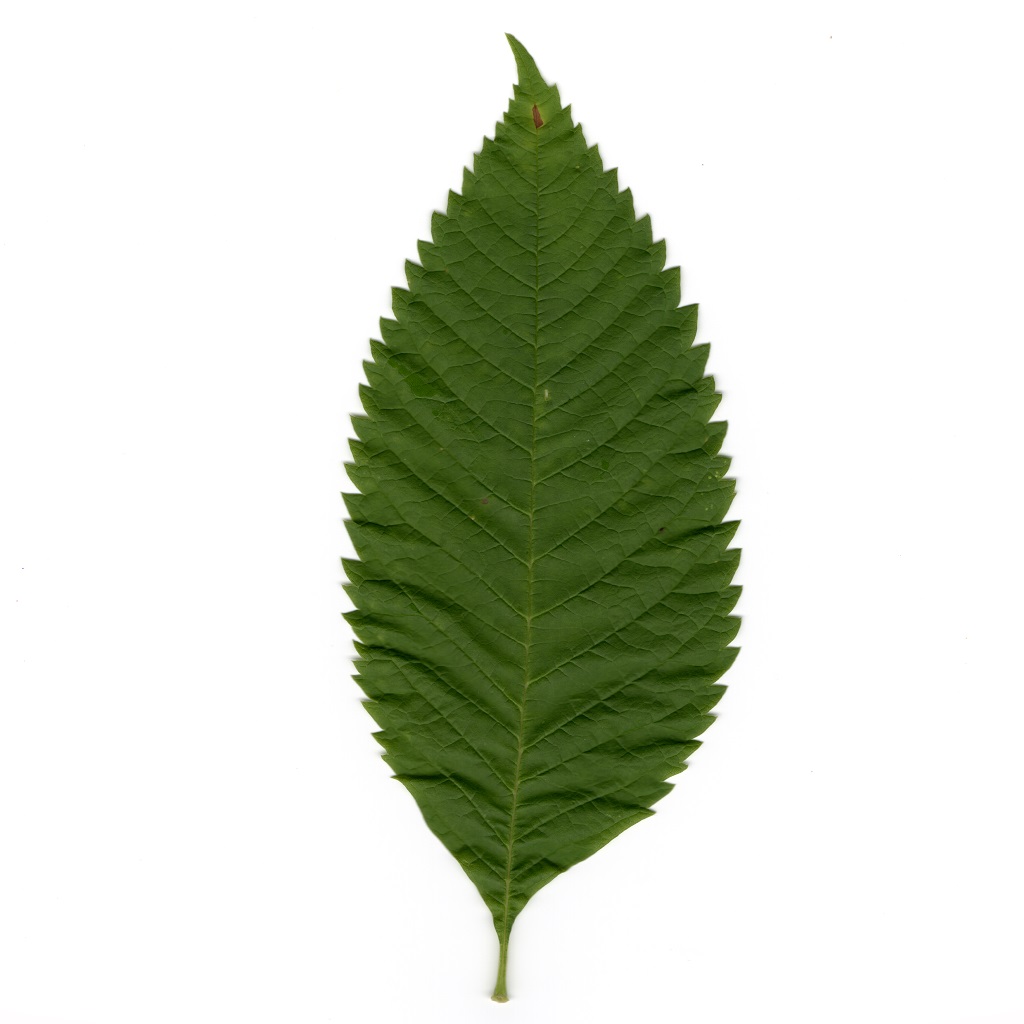

Supplement: S1 Data — (ZIP) [file pone.0293596.s001.zip › S1_data/Tecoma stans.jpg]

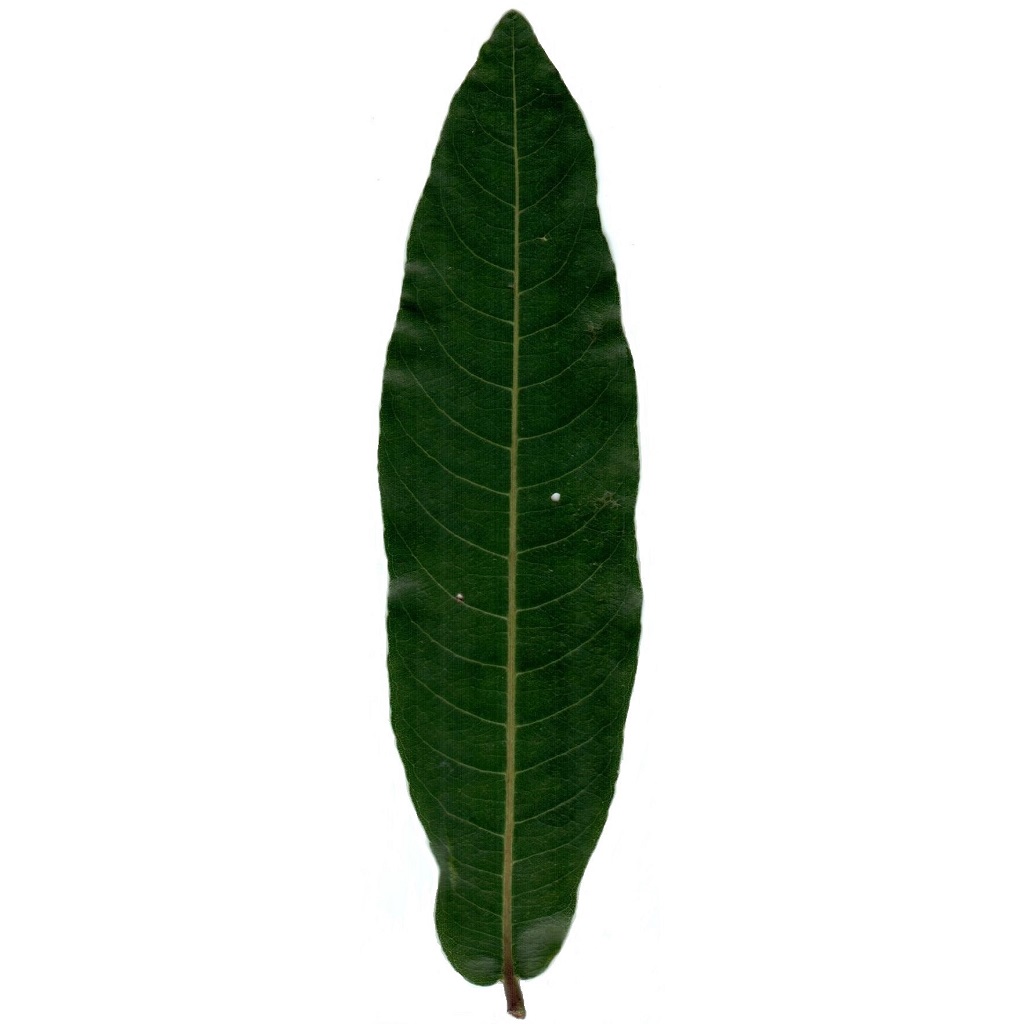

Supplement: S1 Data — (ZIP) [file pone.0293596.s001.zip › S1_data/Terminalia arjuna.jpg]

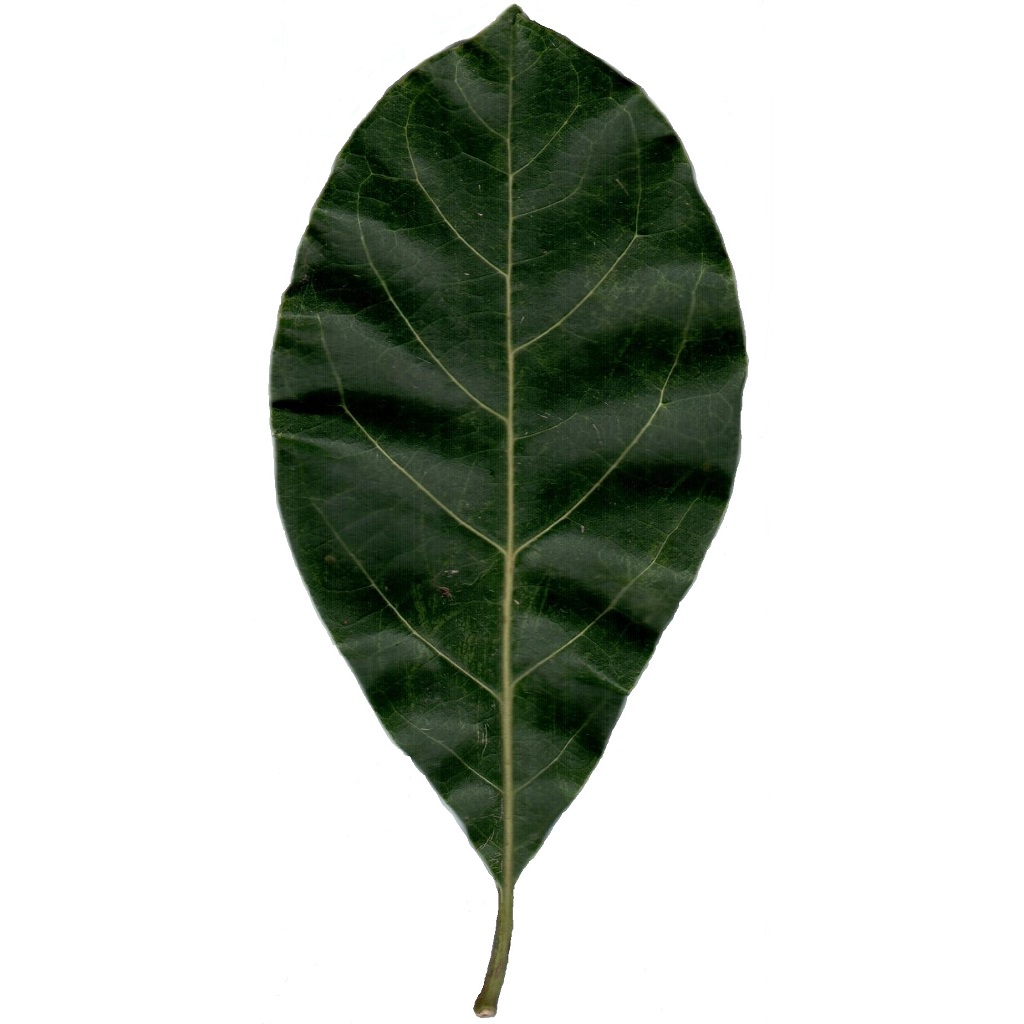

Supplement: S1 Data — (ZIP) [file pone.0293596.s001.zip › S1_data/Terminalia bellirica.jpg]

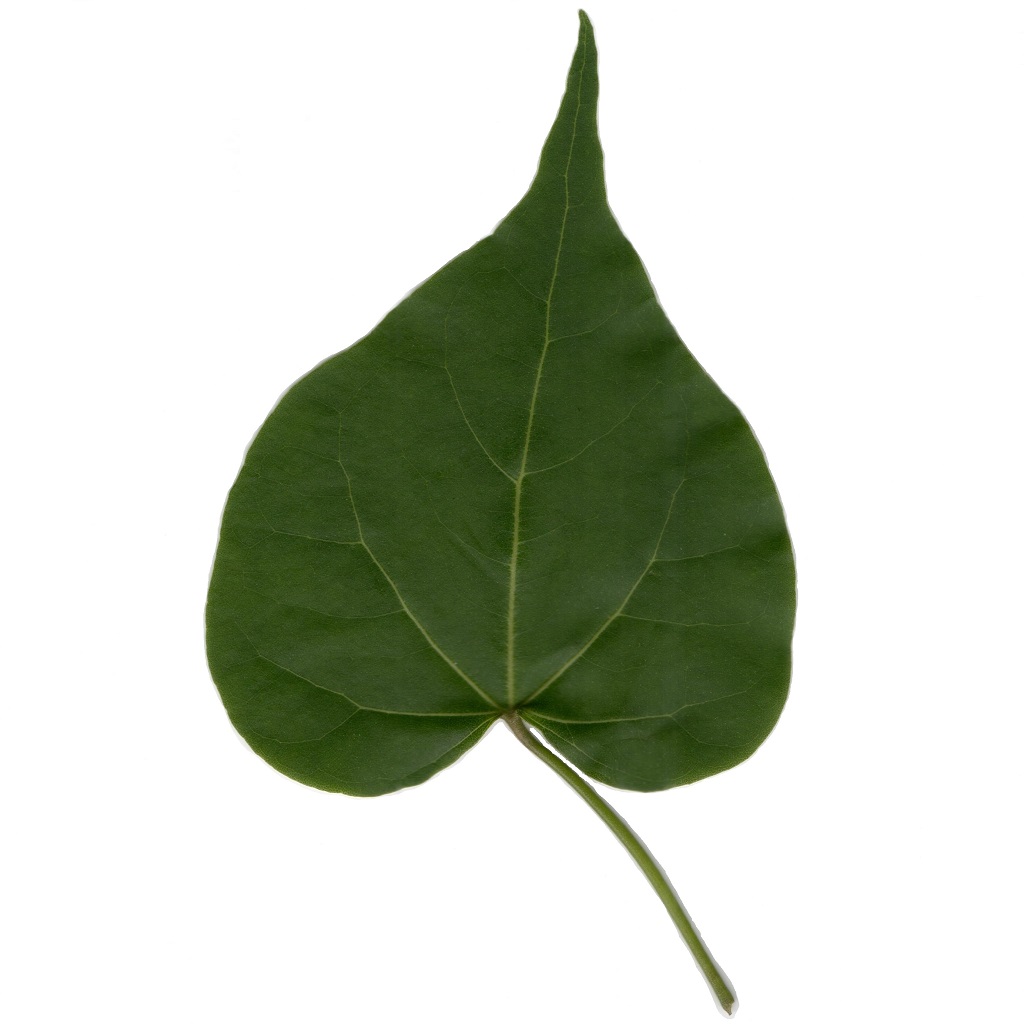

Supplement: S1 Data — (ZIP) [file pone.0293596.s001.zip › S1_data/Thespesia populnea.jpg]

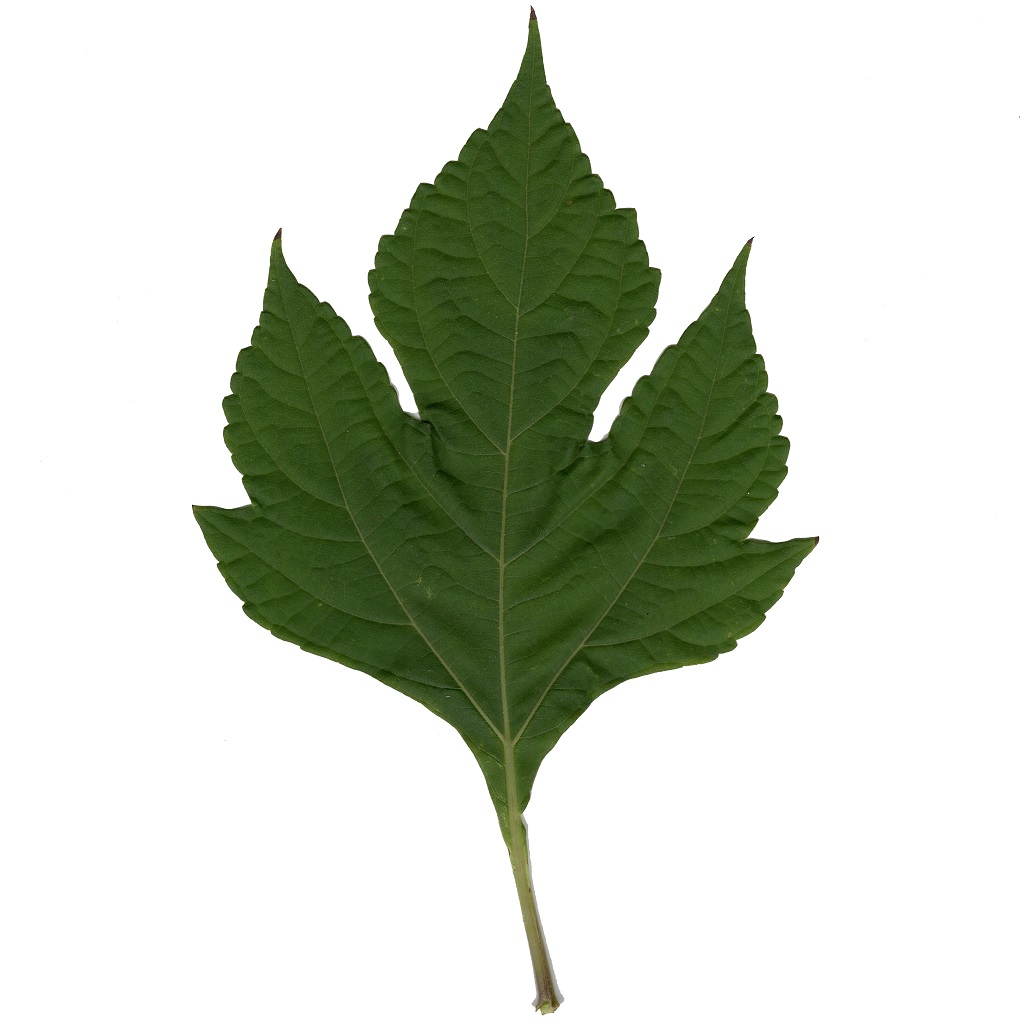

Supplement: S1 Data — (ZIP) [file pone.0293596.s001.zip › S1_data/Tithonia diversifolia.jpg]

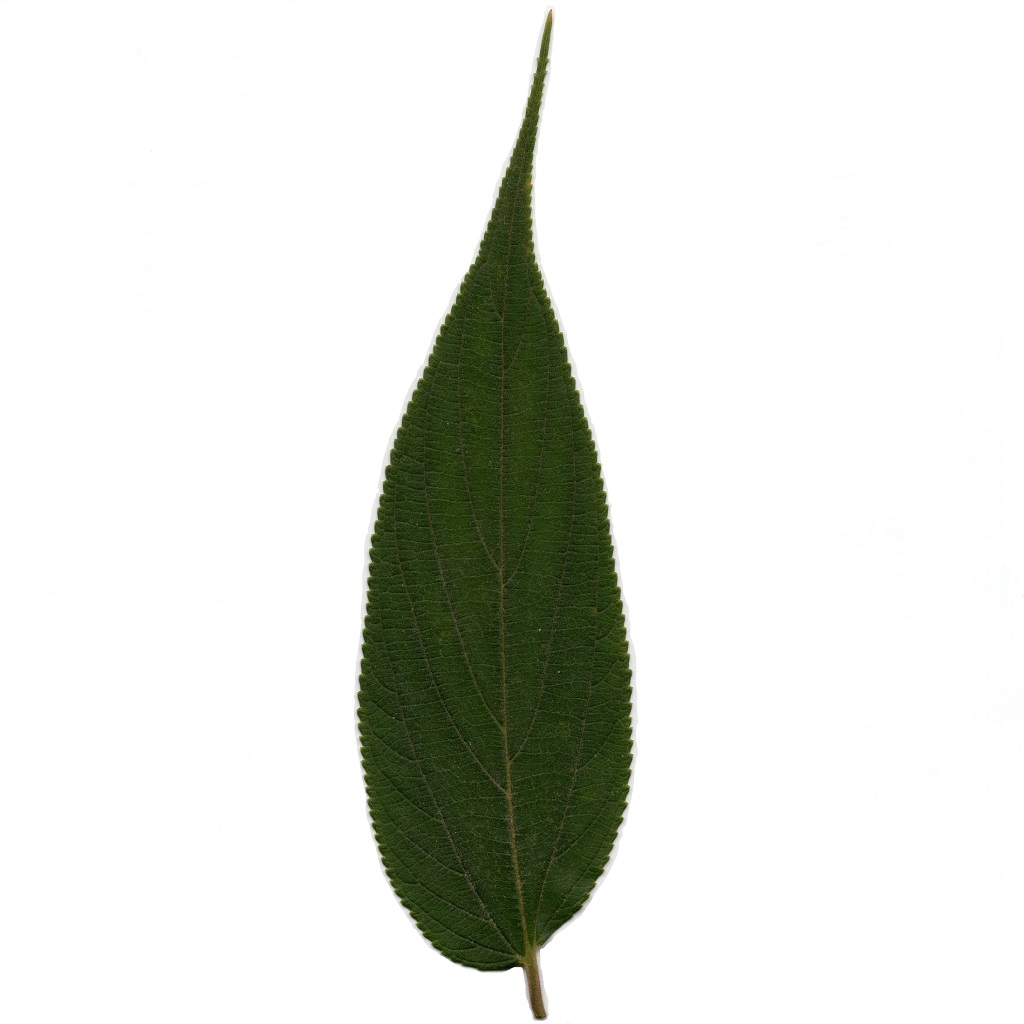

Supplement: S1 Data — (ZIP) [file pone.0293596.s001.zip › S1_data/Trema tomentosa.jpg]

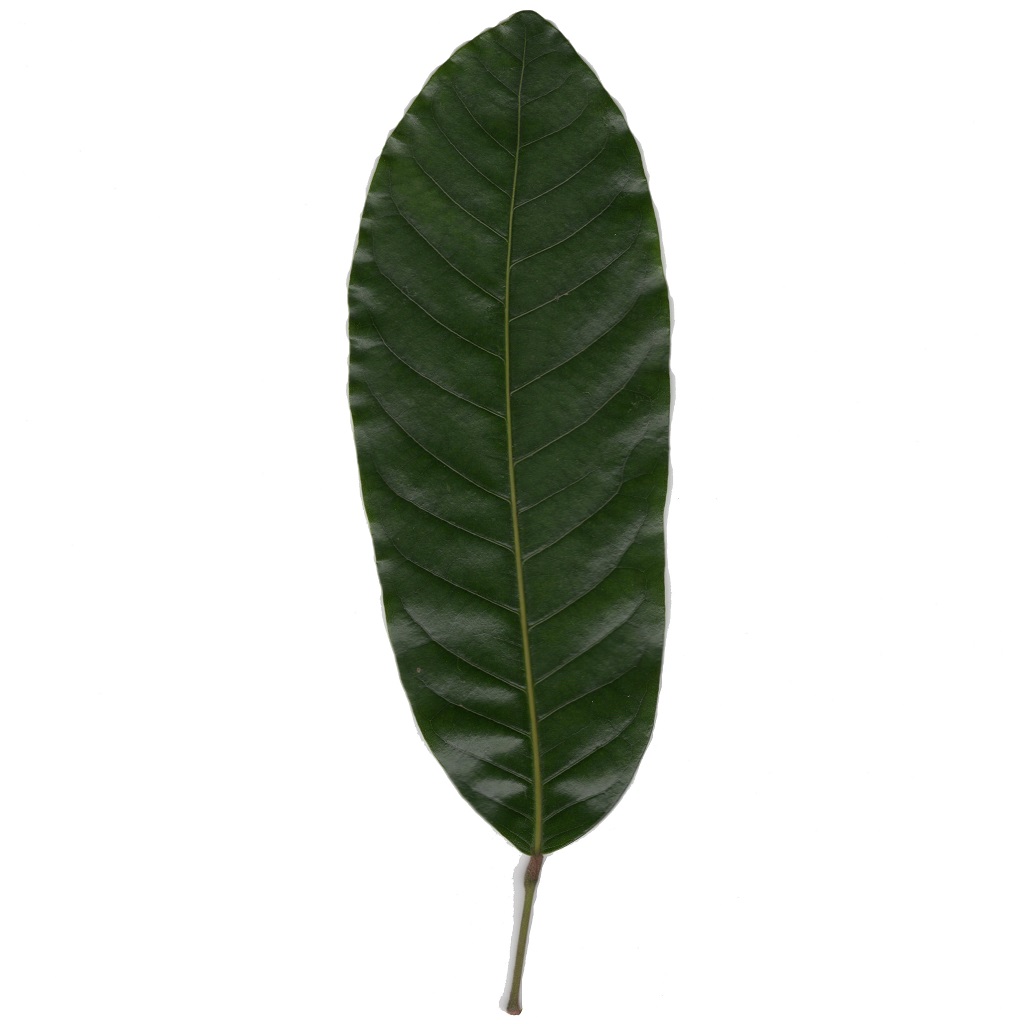

Supplement: S1 Data — (ZIP) [file pone.0293596.s001.zip › S1_data/Vateria indica.jpg]

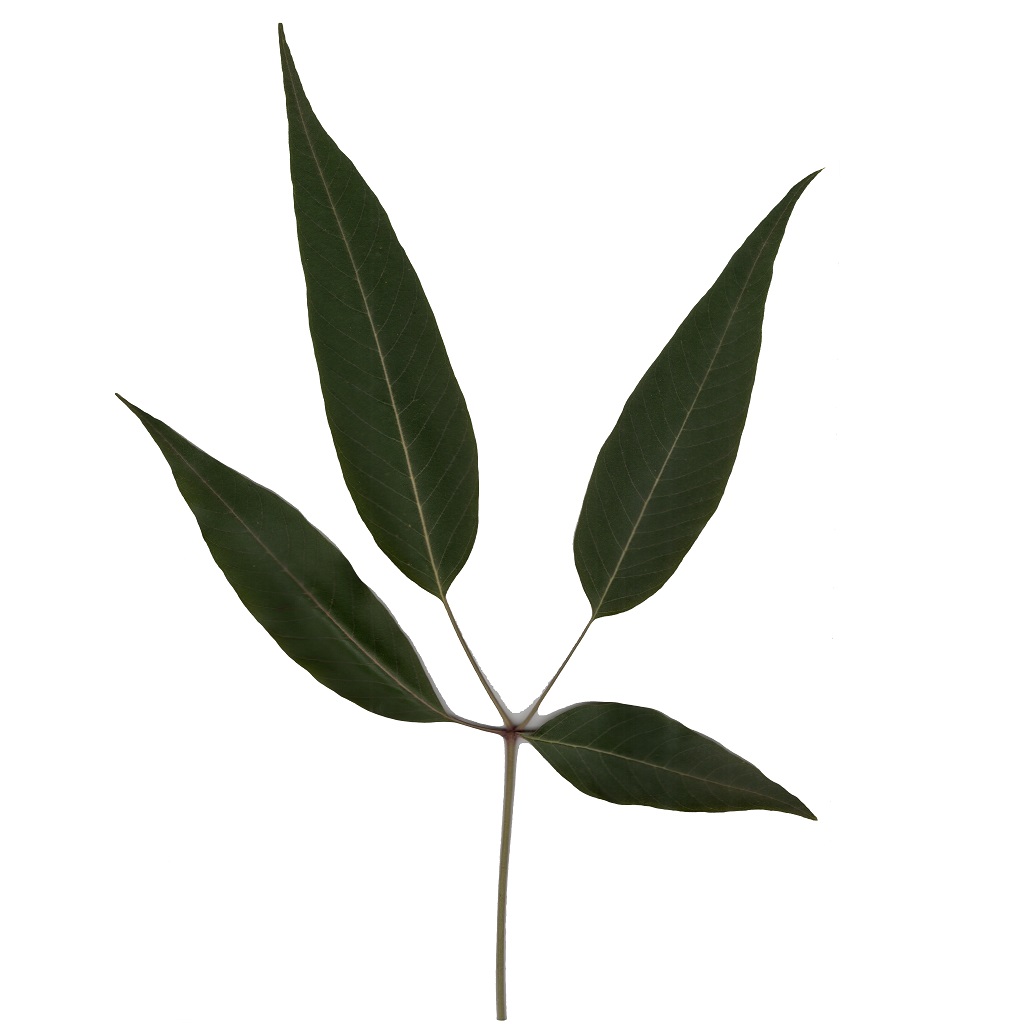

Supplement: S1 Data — (ZIP) [file pone.0293596.s001.zip › S1_data/Vitex negundo.jpg]

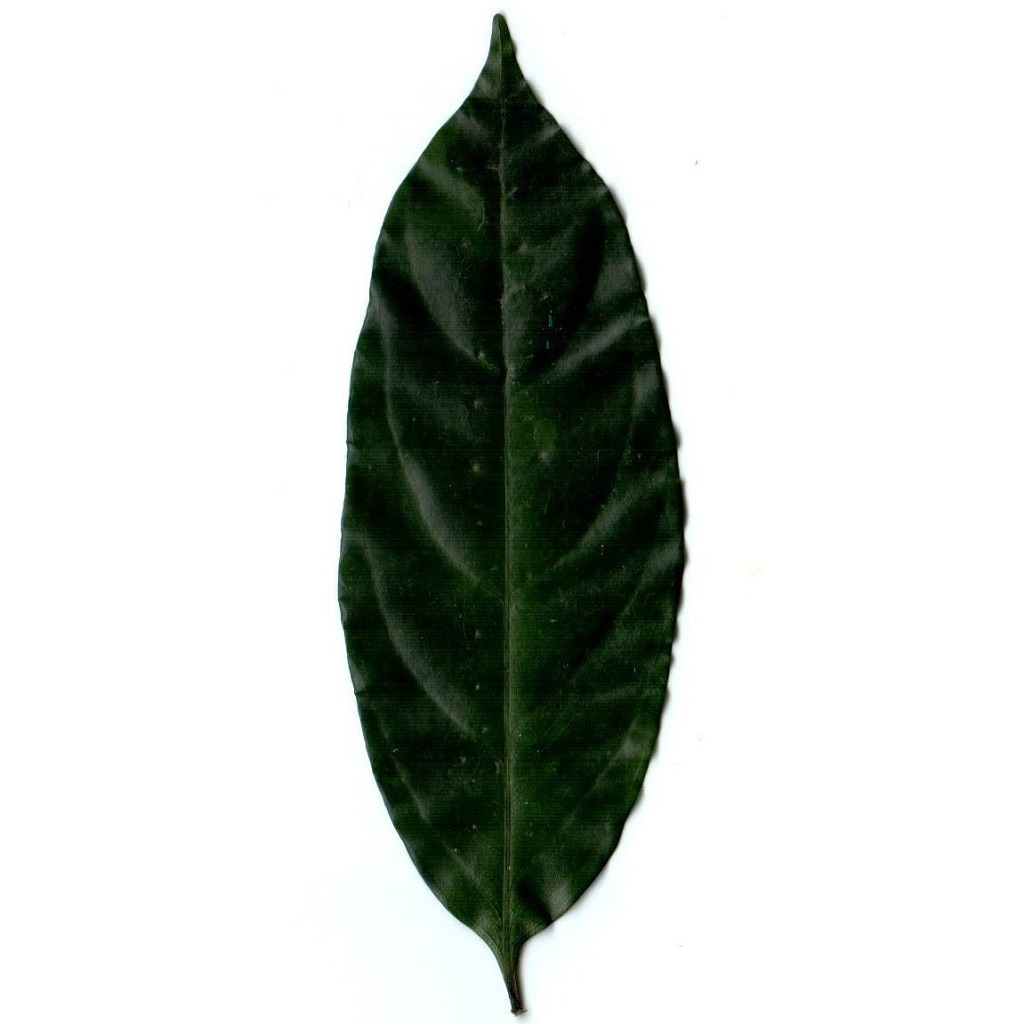

Supplement: S1 Data — (ZIP) [file pone.0293596.s001.zip › S1_data/Xanthophyllum flavescens.jpg]
