# Supplementary figures and images for: Geometric entropy of plant leaves: A measure of morphological complexity (part 1 of 2)
Source: PLoS One. 2024 Jan 2;19(1):e0293596. doi: 10.1371/journal.pone.0293596 (PMC10760904; doi:10.1371/journal.pone.0293596)

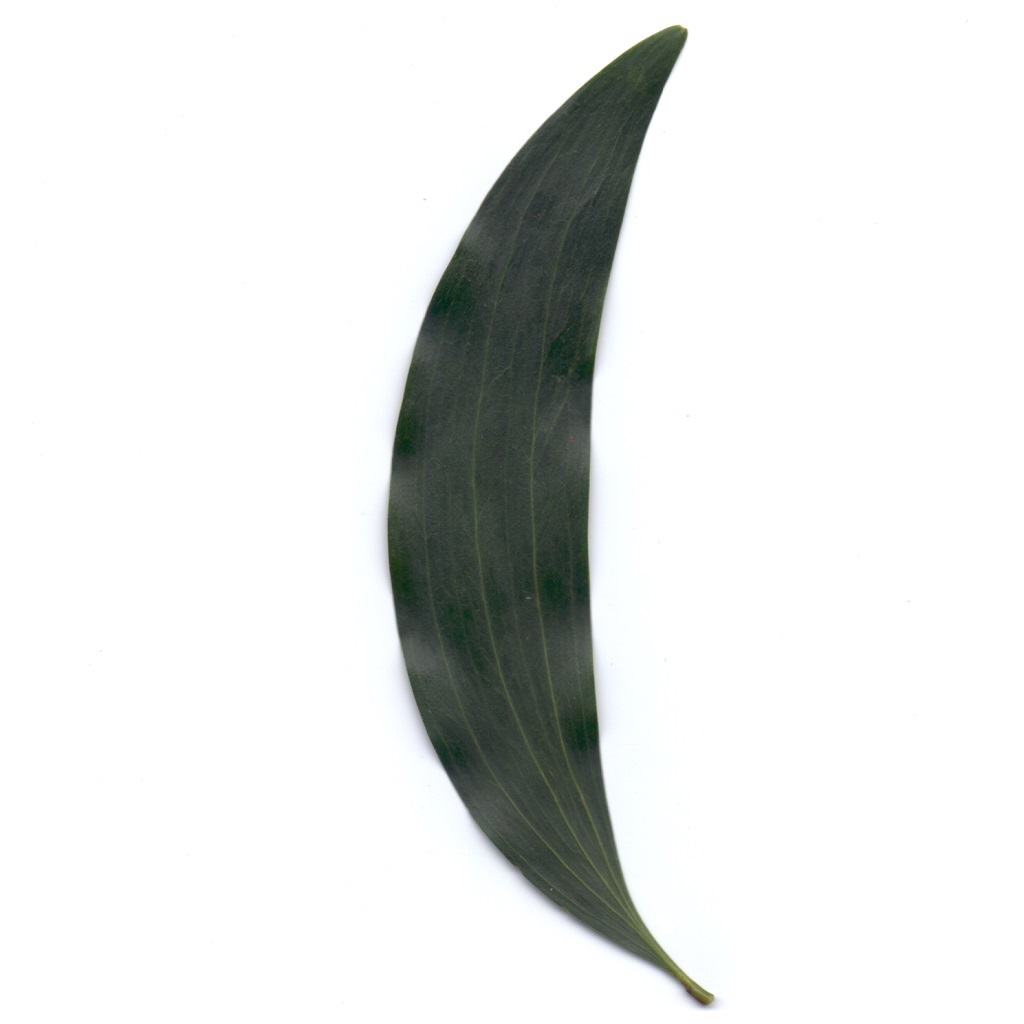

Supplement: S1 Data — (ZIP) [file pone.0293596.s001.zip › S1_data/Acacia auriculiformis.jpg]

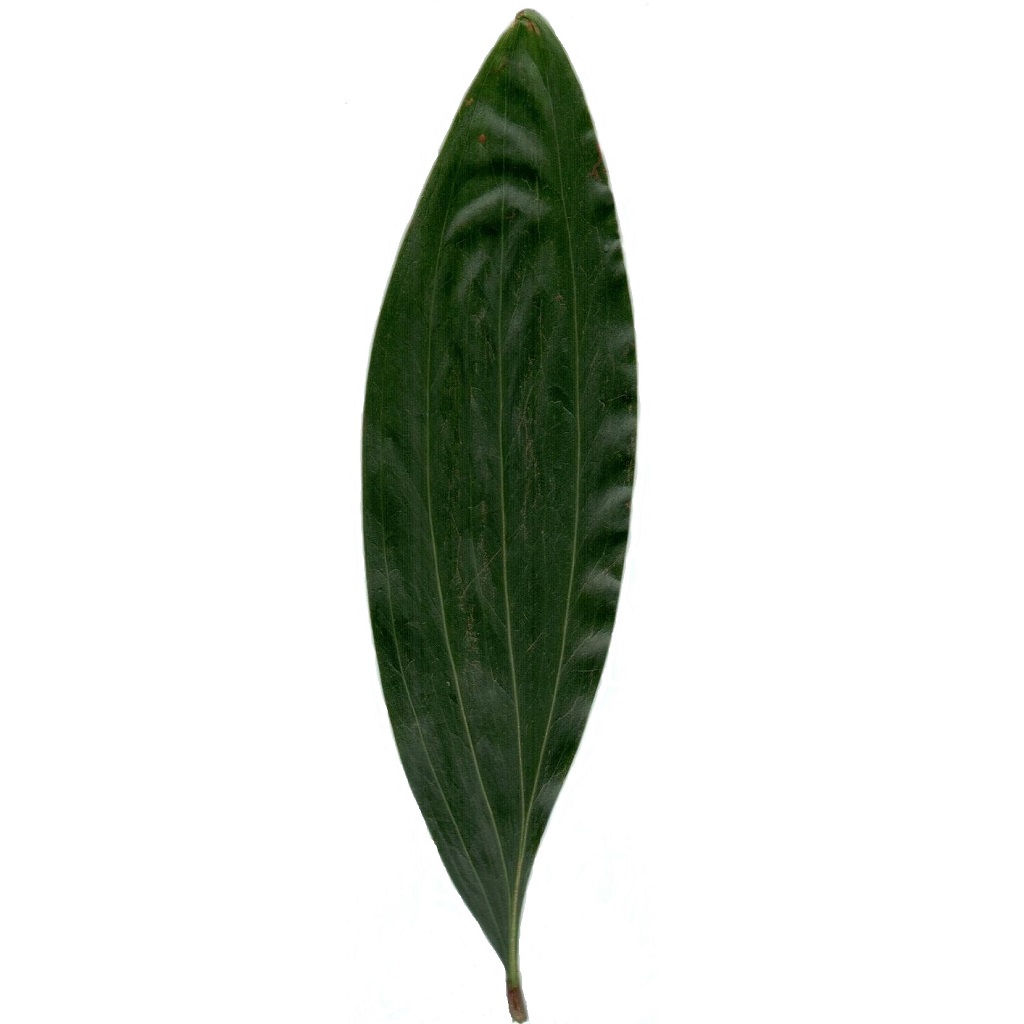

Supplement: S1 Data — (ZIP) [file pone.0293596.s001.zip › S1_data/Acacia mangium.jpg]

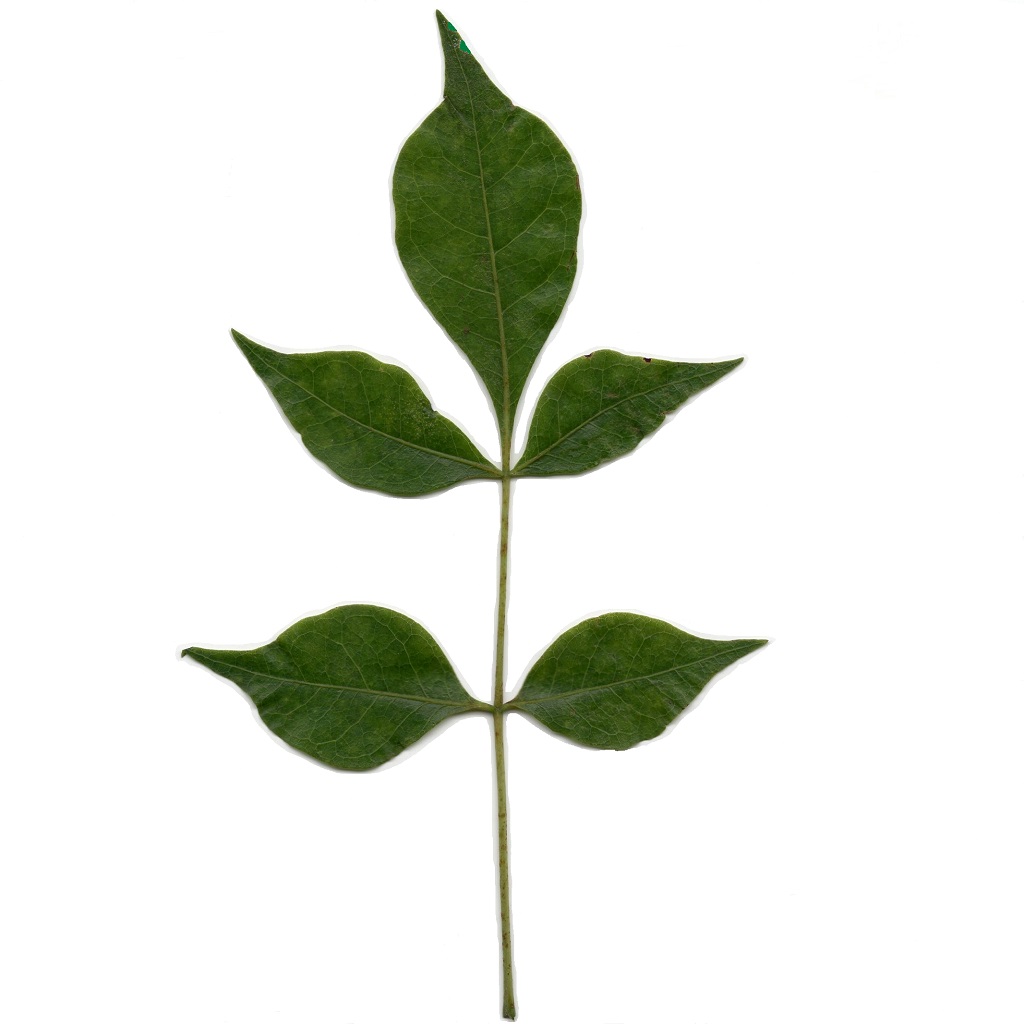

Supplement: S1 Data — (ZIP) [file pone.0293596.s001.zip › S1_data/Aegle marmelos.jpg]

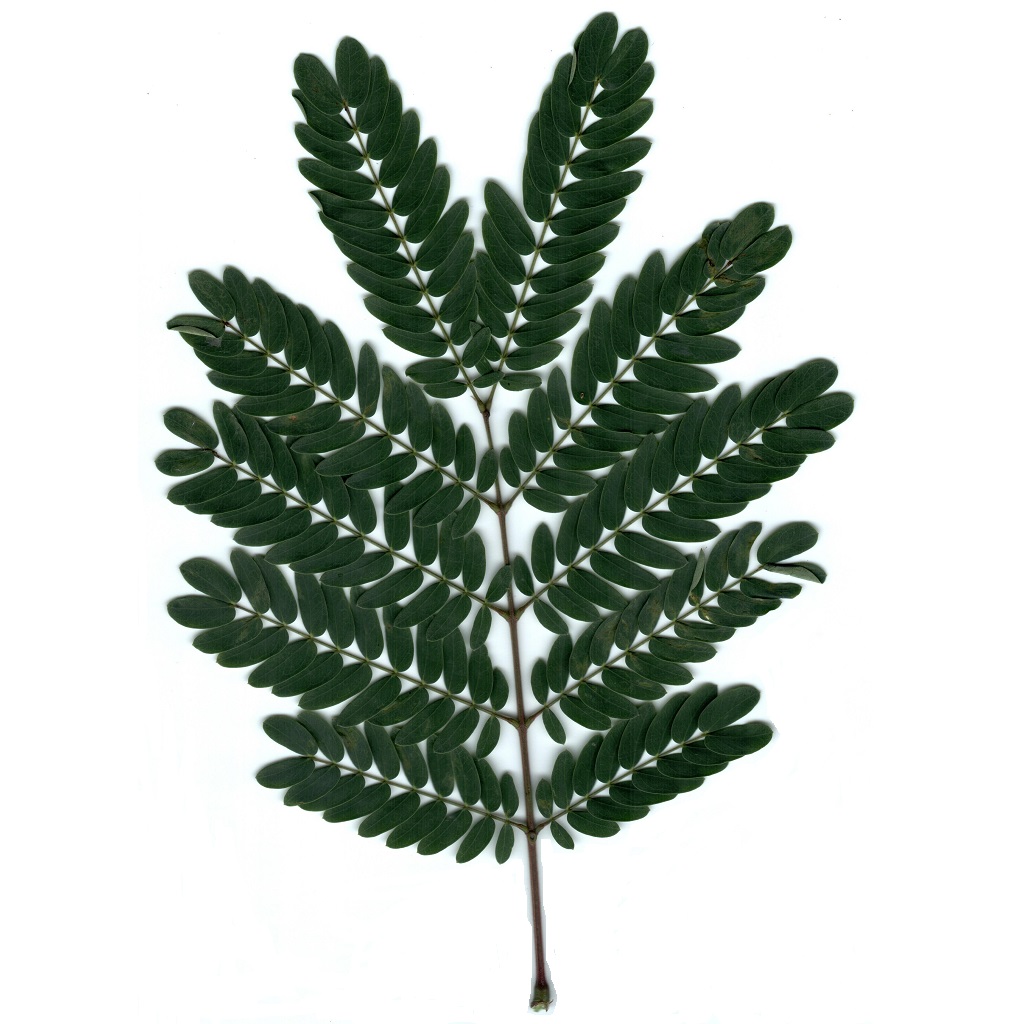

Supplement: S1 Data — (ZIP) [file pone.0293596.s001.zip › S1_data/Albizia odoratissima.jpg]

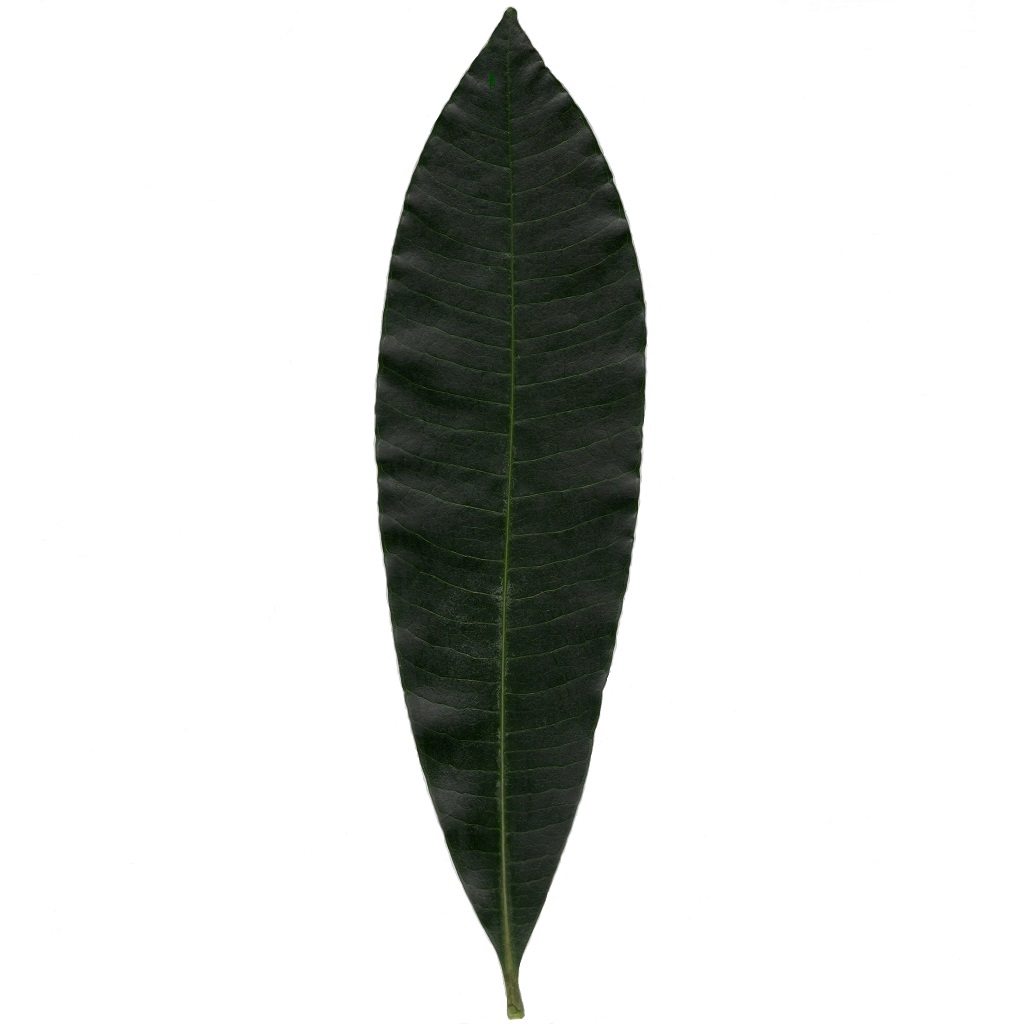

Supplement: S1 Data — (ZIP) [file pone.0293596.s001.zip › S1_data/Alstonia scholaris.jpg]

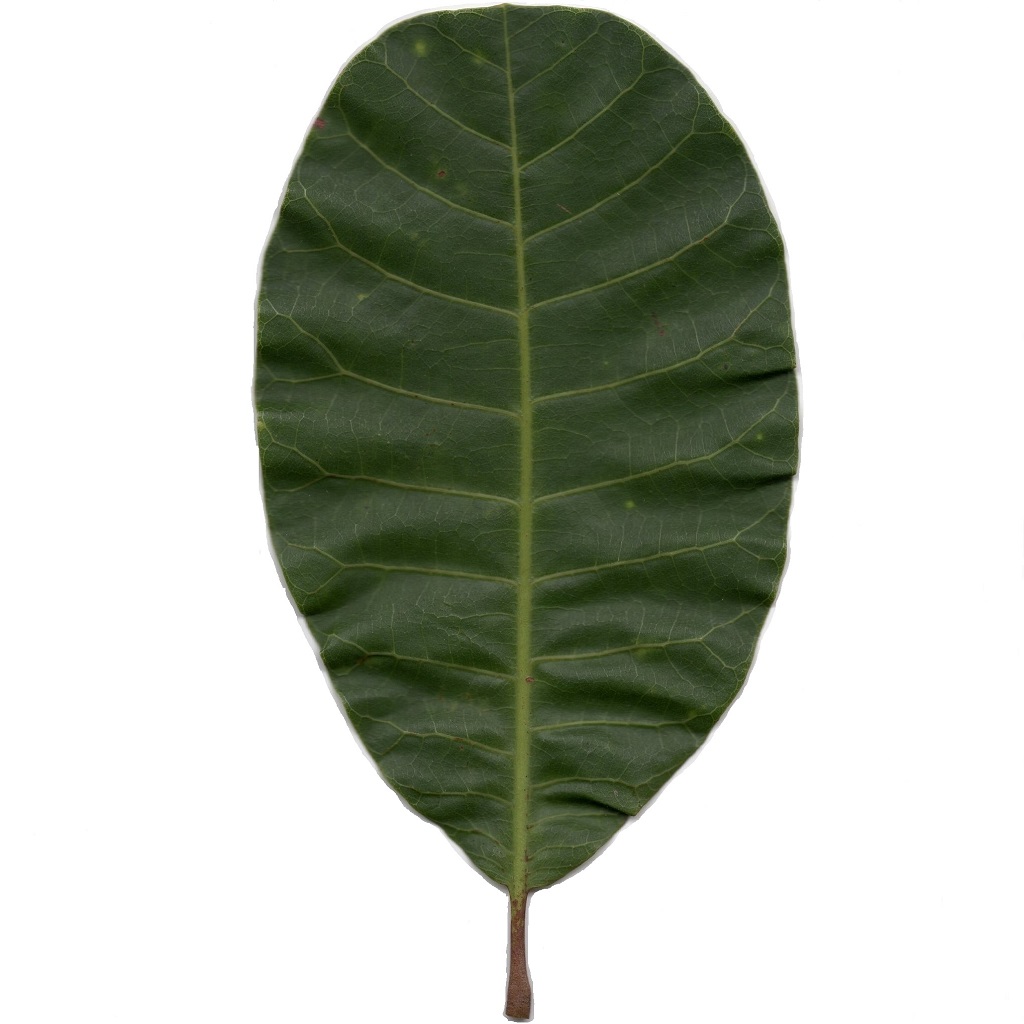

Supplement: S1 Data — (ZIP) [file pone.0293596.s001.zip › S1_data/Anacardium occidentale.jpg]

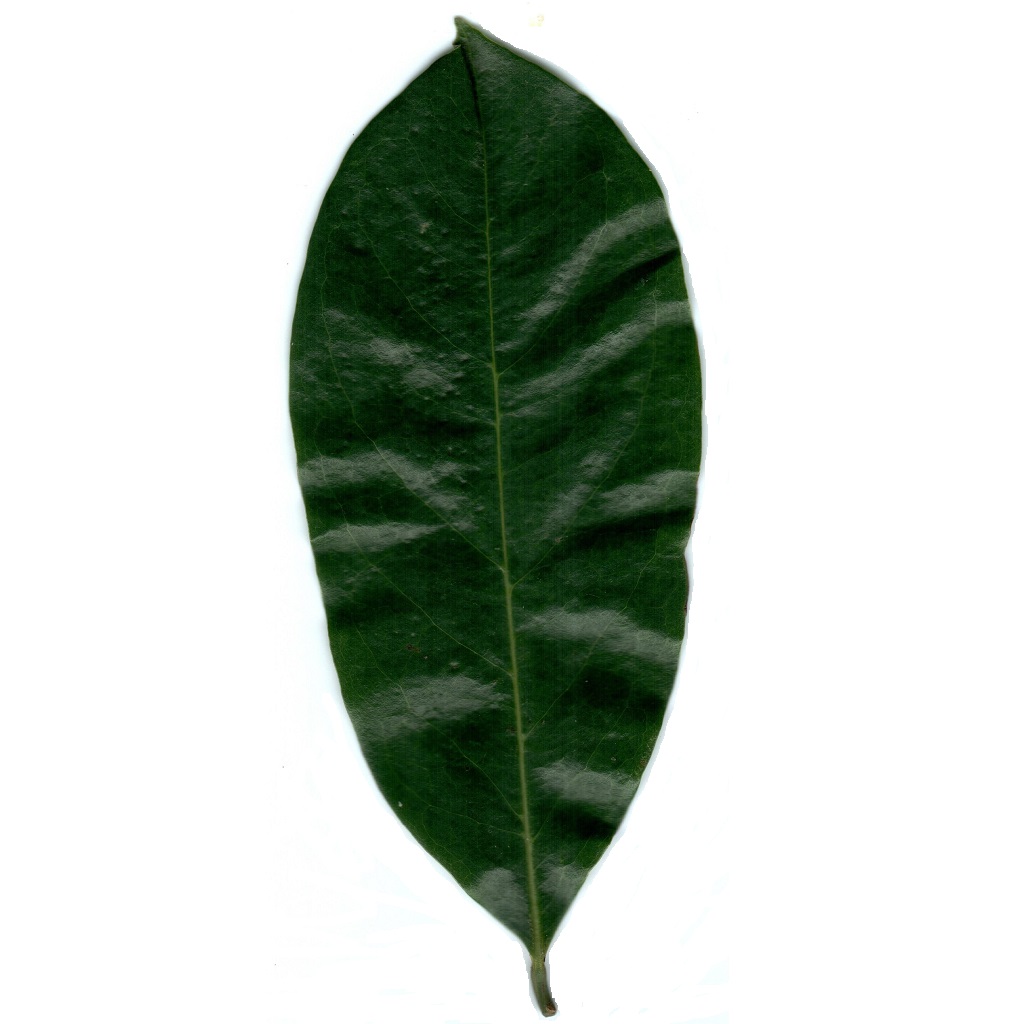

Supplement: S1 Data — (ZIP) [file pone.0293596.s001.zip › S1_data/Annona muricata.jpg]

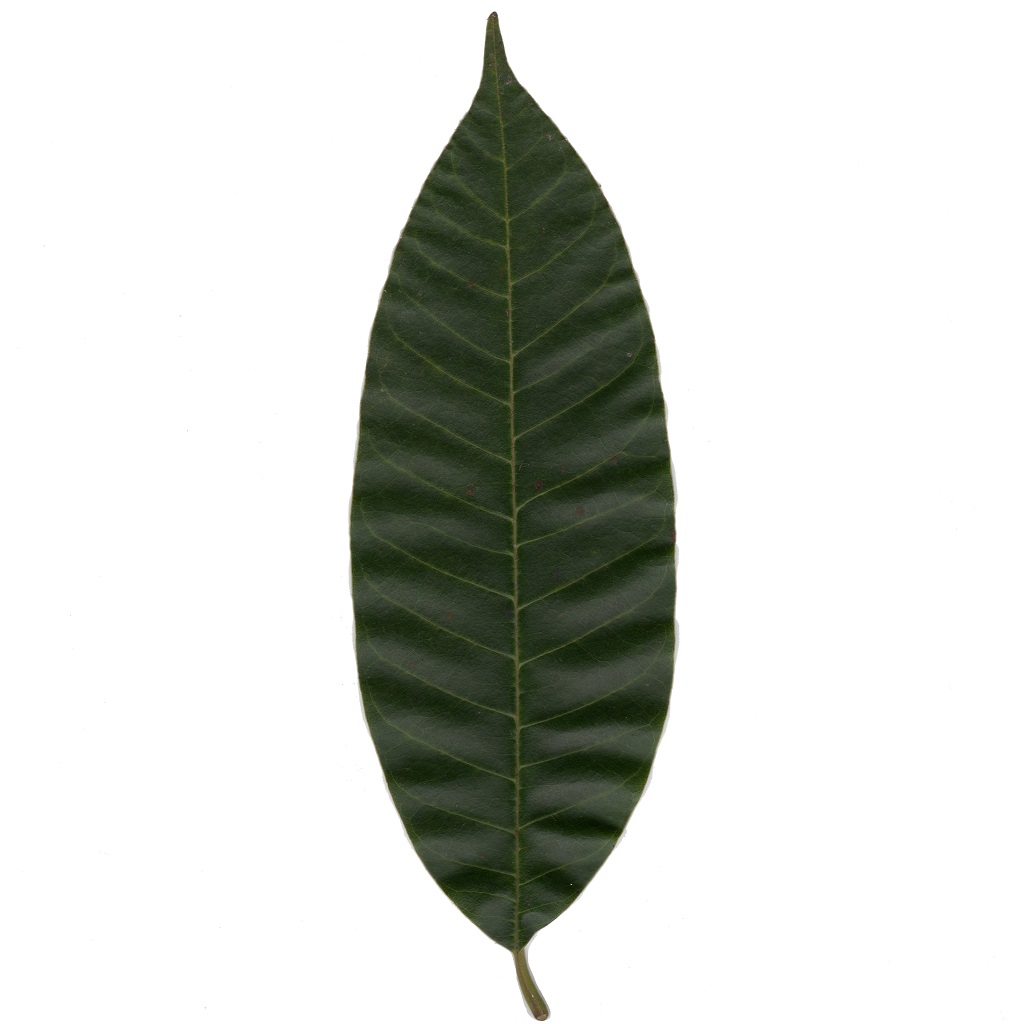

Supplement: S1 Data — (ZIP) [file pone.0293596.s001.zip › S1_data/Annona reticulata.jpg]

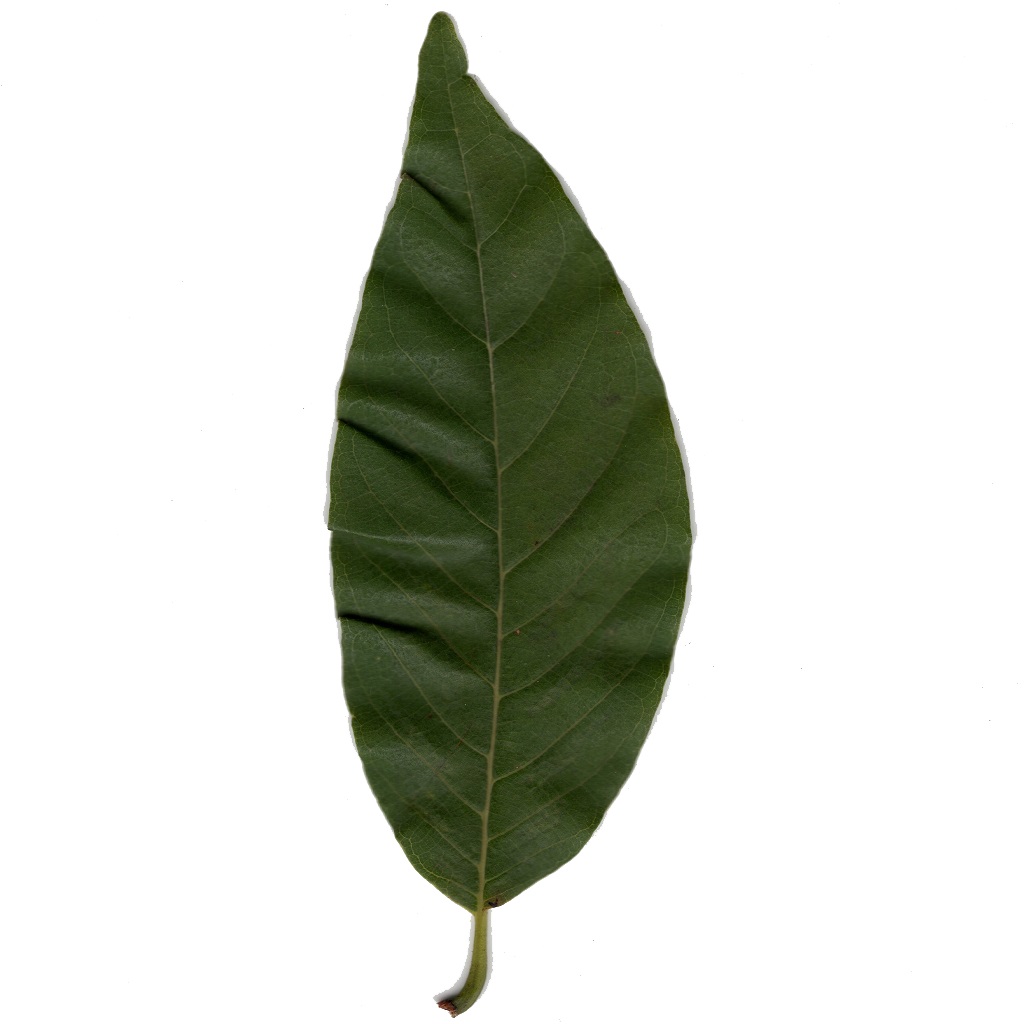

Supplement: S1 Data — (ZIP) [file pone.0293596.s001.zip › S1_data/Annona squamosa.jpg]

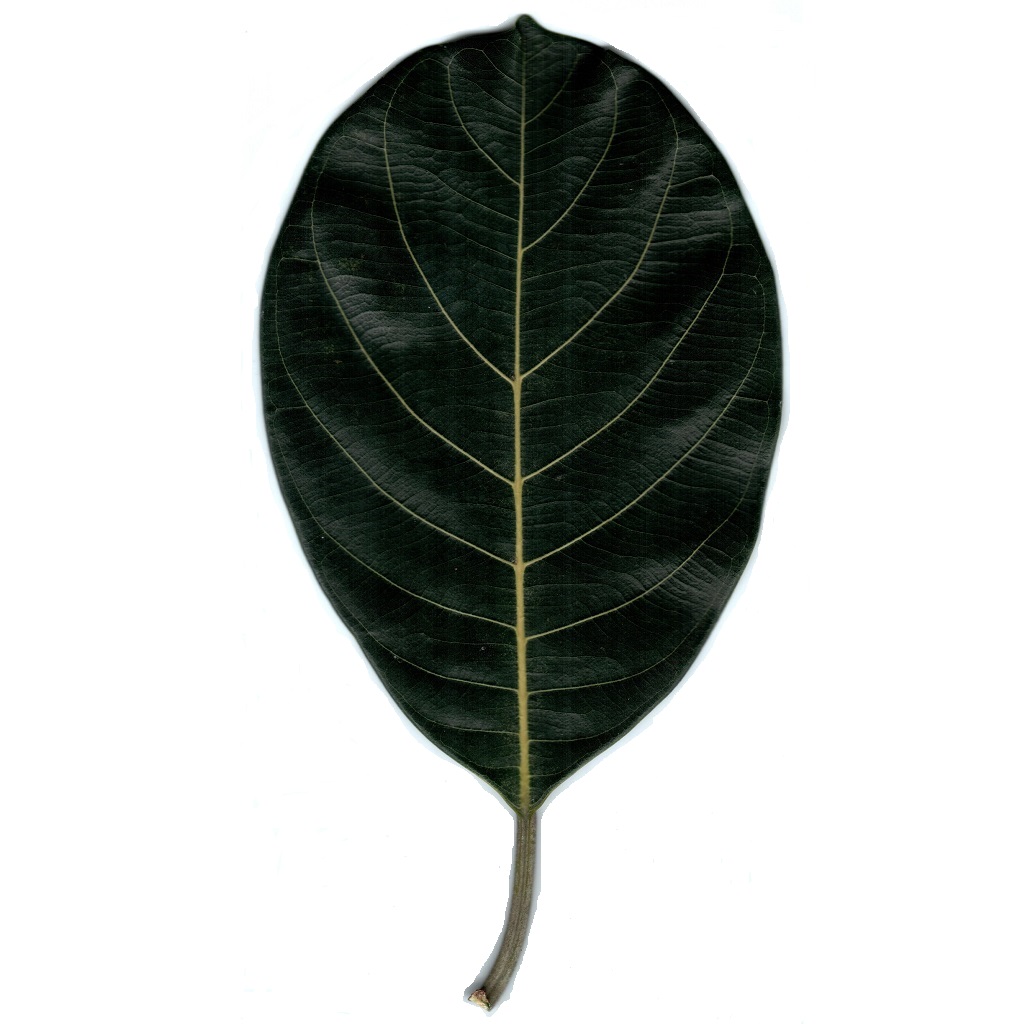

Supplement: S1 Data — (ZIP) [file pone.0293596.s001.zip › S1_data/Artocarpus heterophyllus.jpg]

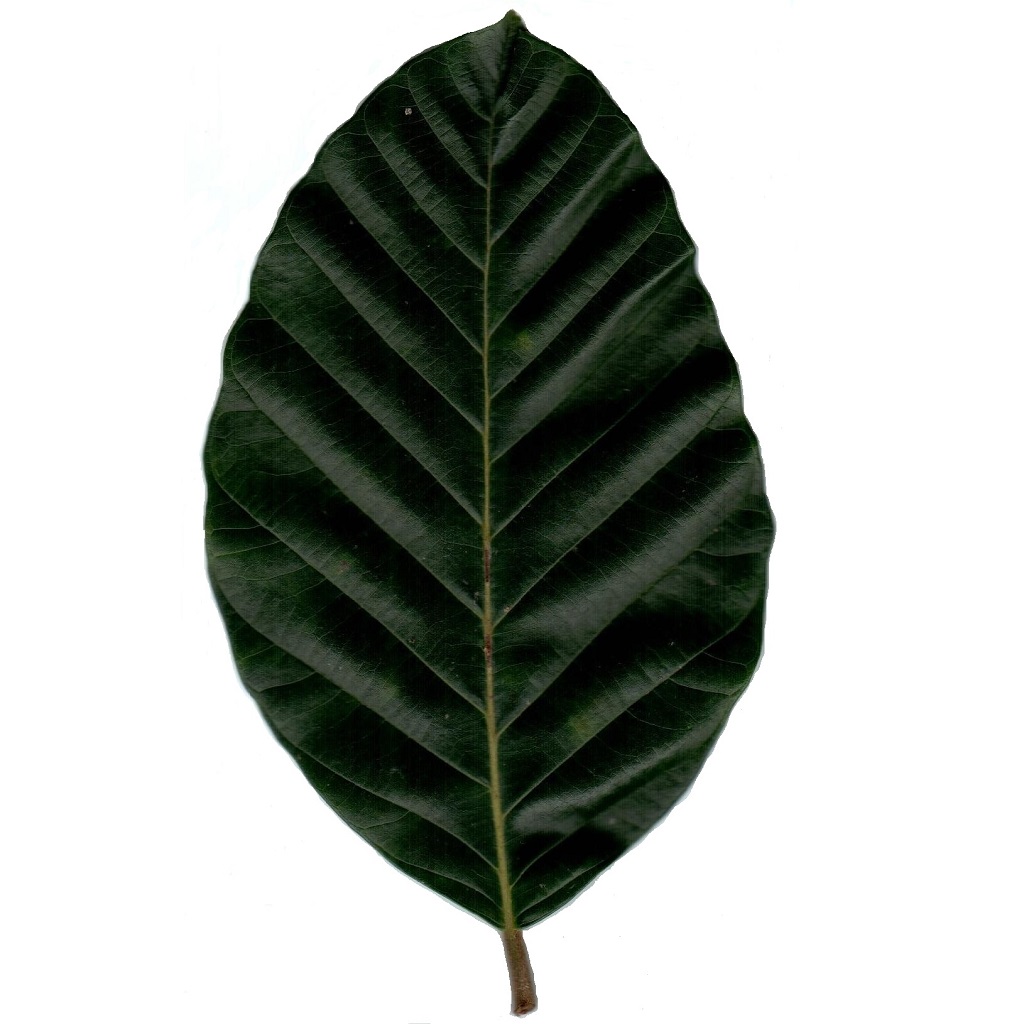

Supplement: S1 Data — (ZIP) [file pone.0293596.s001.zip › S1_data/Artocarpus hirsutus.jpg]

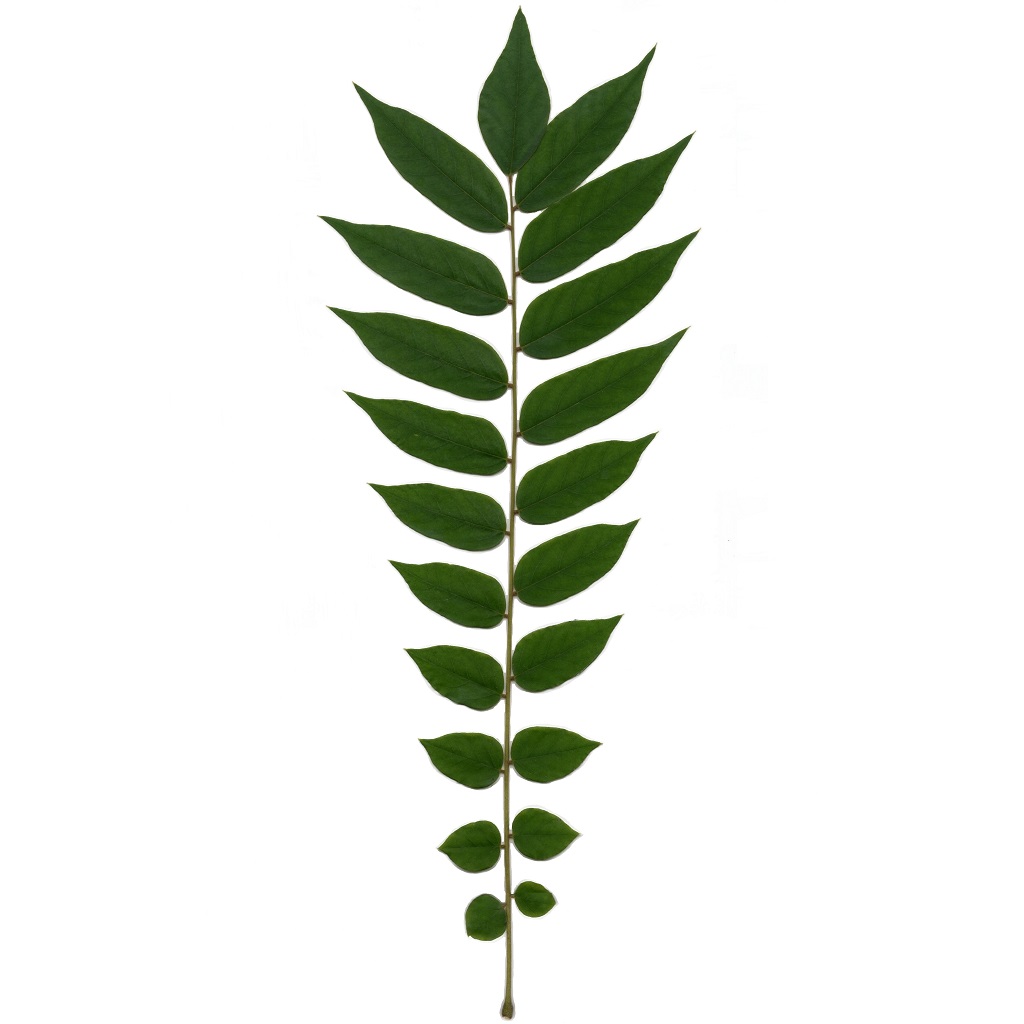

Supplement: S1 Data — (ZIP) [file pone.0293596.s001.zip › S1_data/Averrhoa bilimbi.jpg]

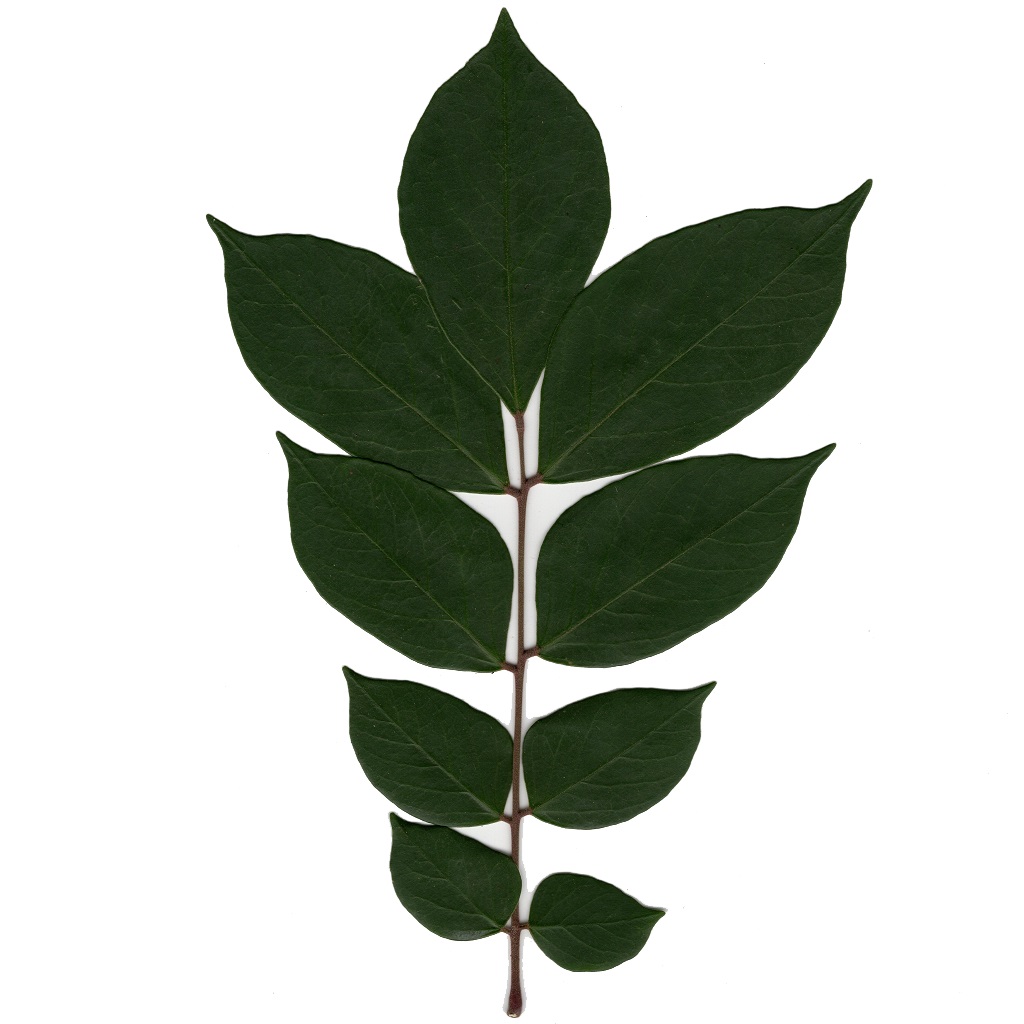

Supplement: S1 Data — (ZIP) [file pone.0293596.s001.zip › S1_data/Averrhoa carambola.jpg]

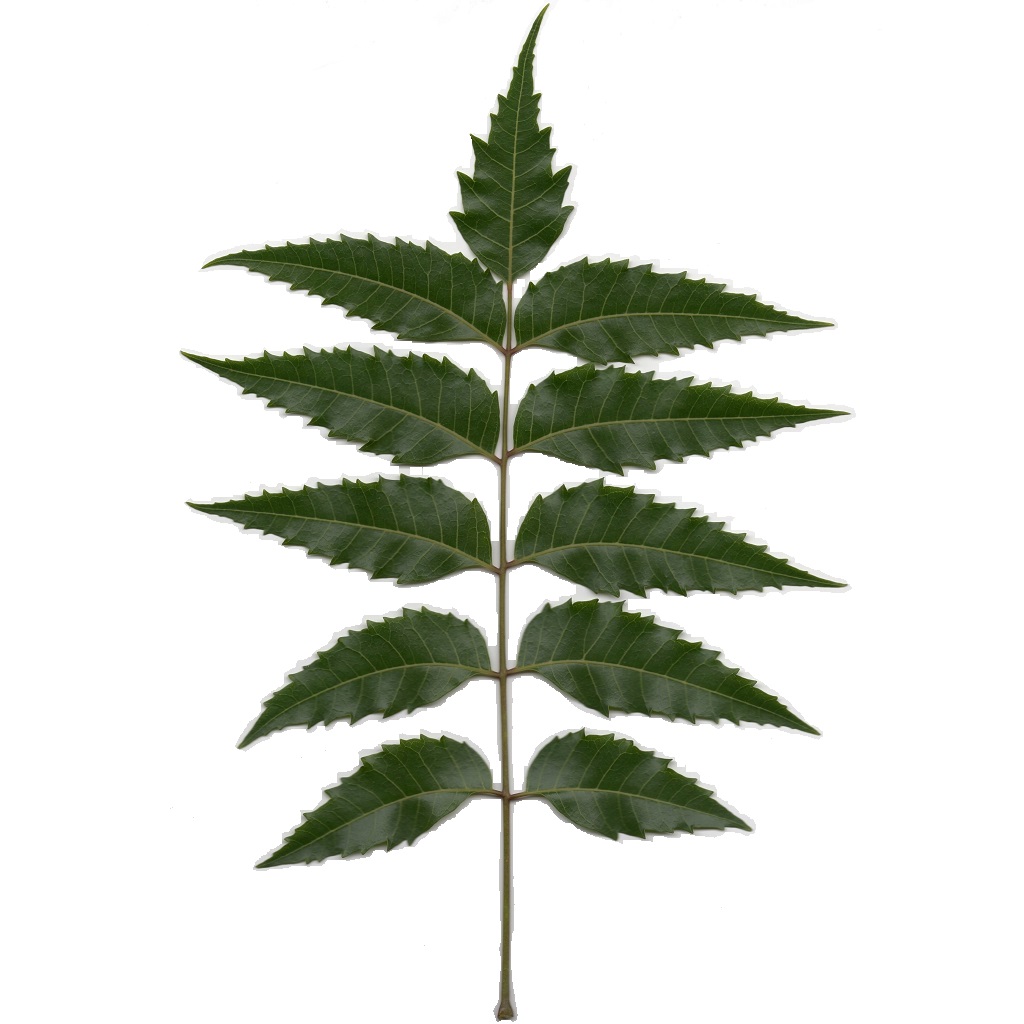

Supplement: S1 Data — (ZIP) [file pone.0293596.s001.zip › S1_data/Azadirachta indica.jpg]

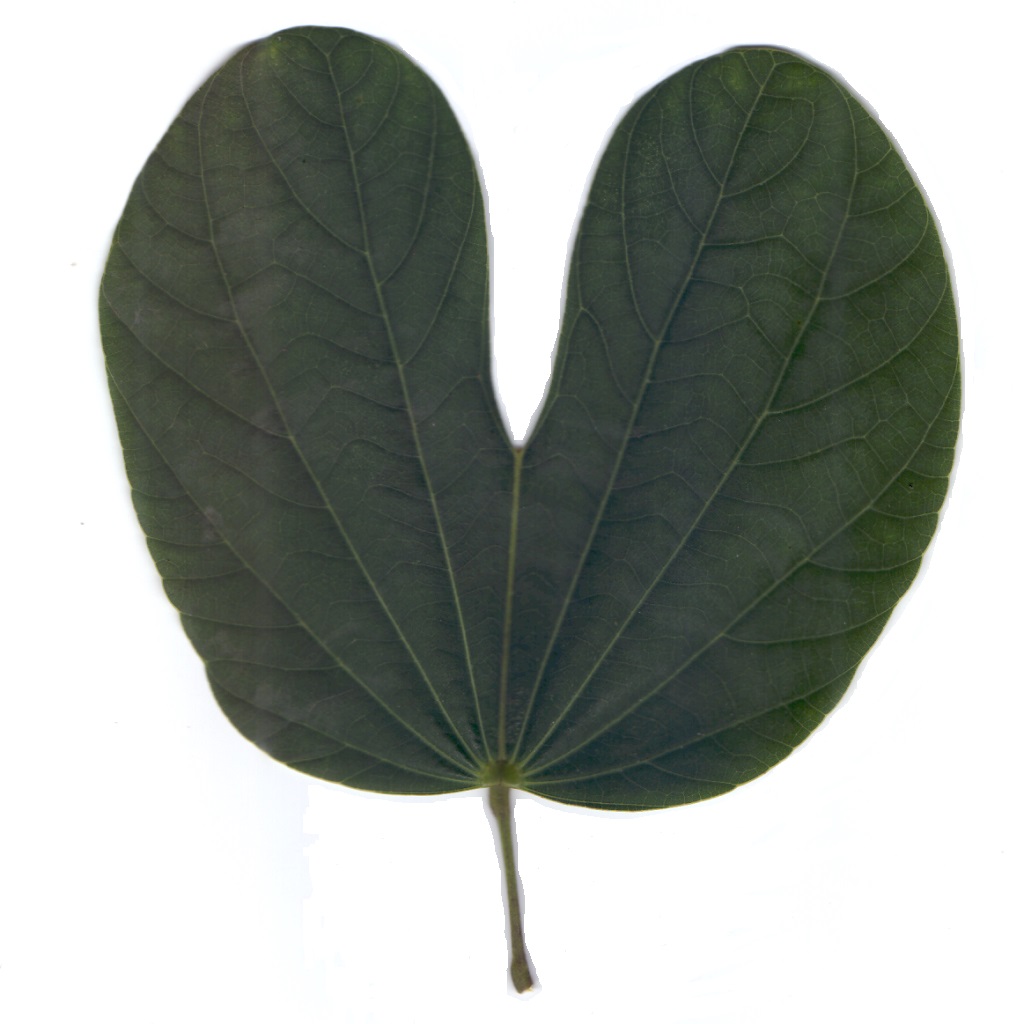

Supplement: S1 Data — (ZIP) [file pone.0293596.s001.zip › S1_data/Bauhinia purpurea.jpg]

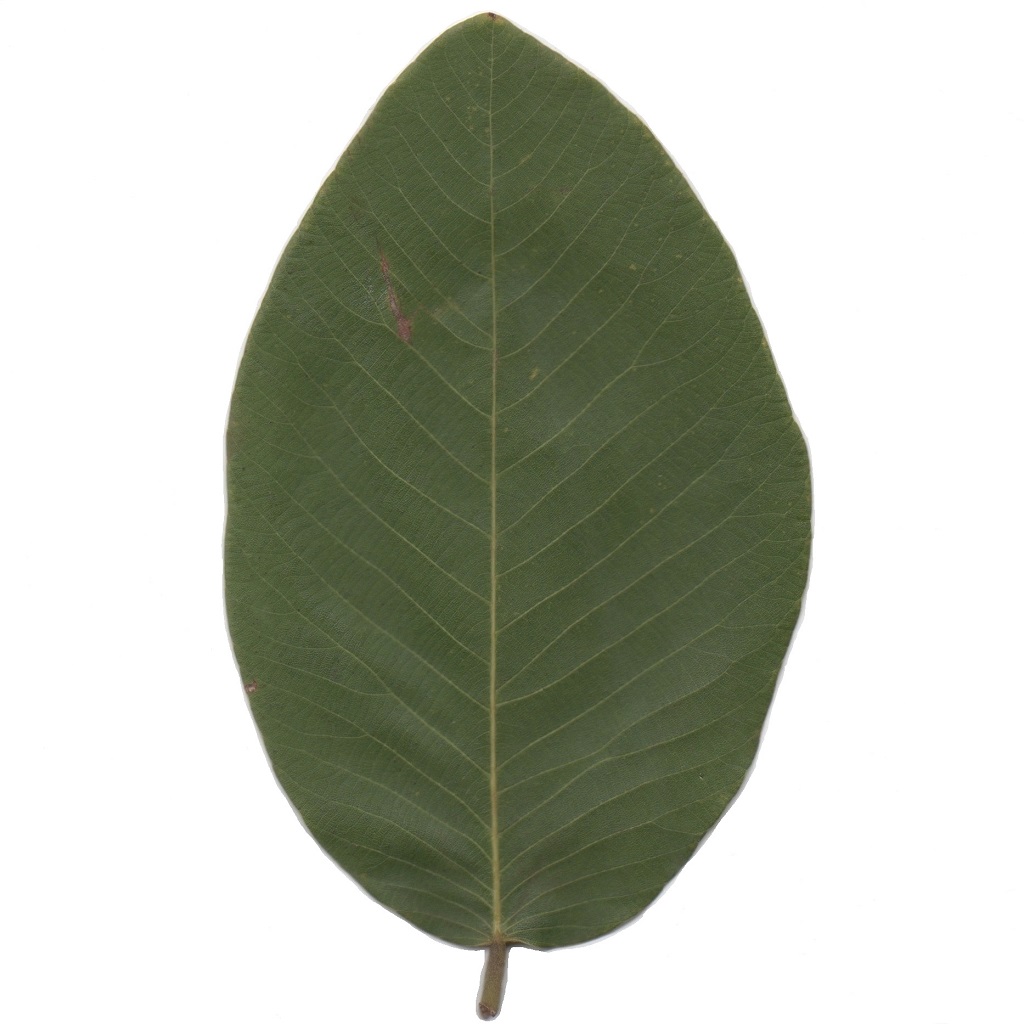

Supplement: S1 Data — (ZIP) [file pone.0293596.s001.zip › S1_data/Briedelia retusa.jpg]

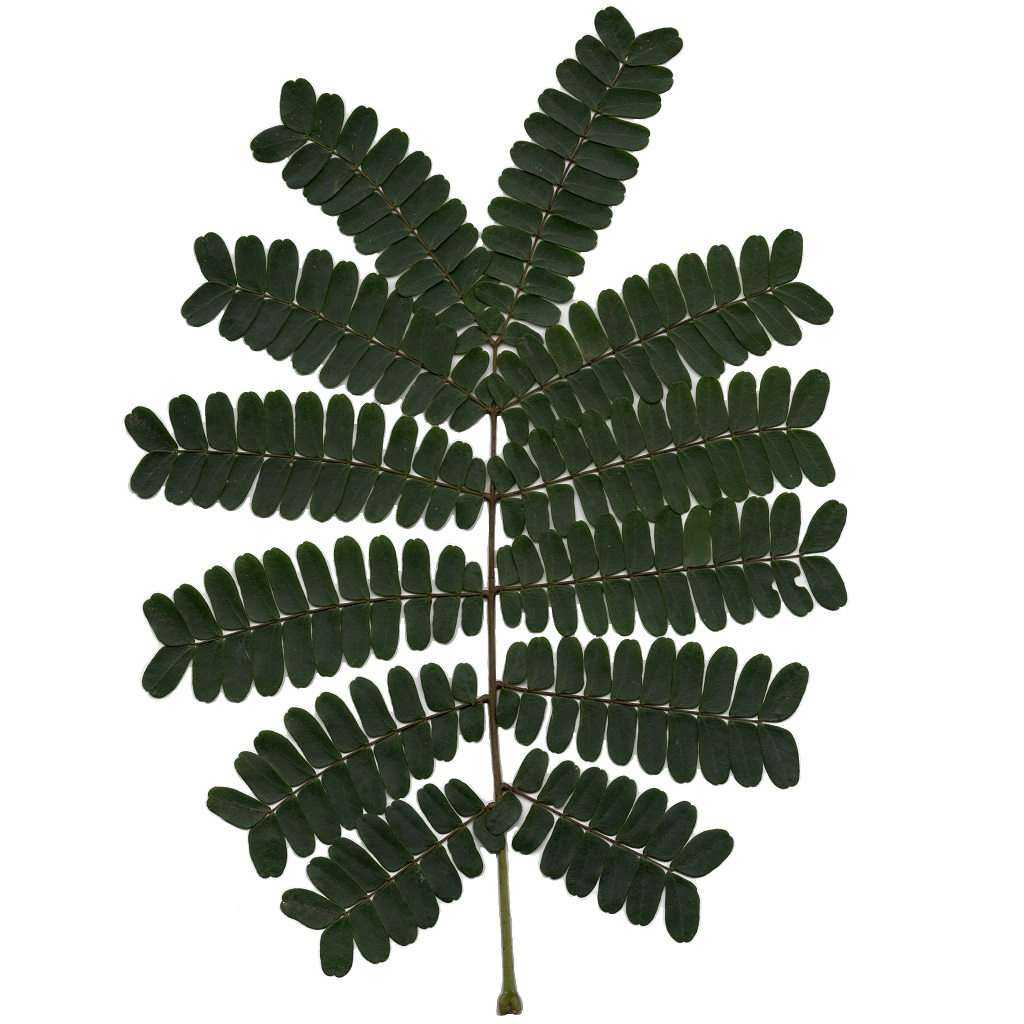

Supplement: S1 Data — (ZIP) [file pone.0293596.s001.zip › S1_data/Caesalpenia sappan.jpg]

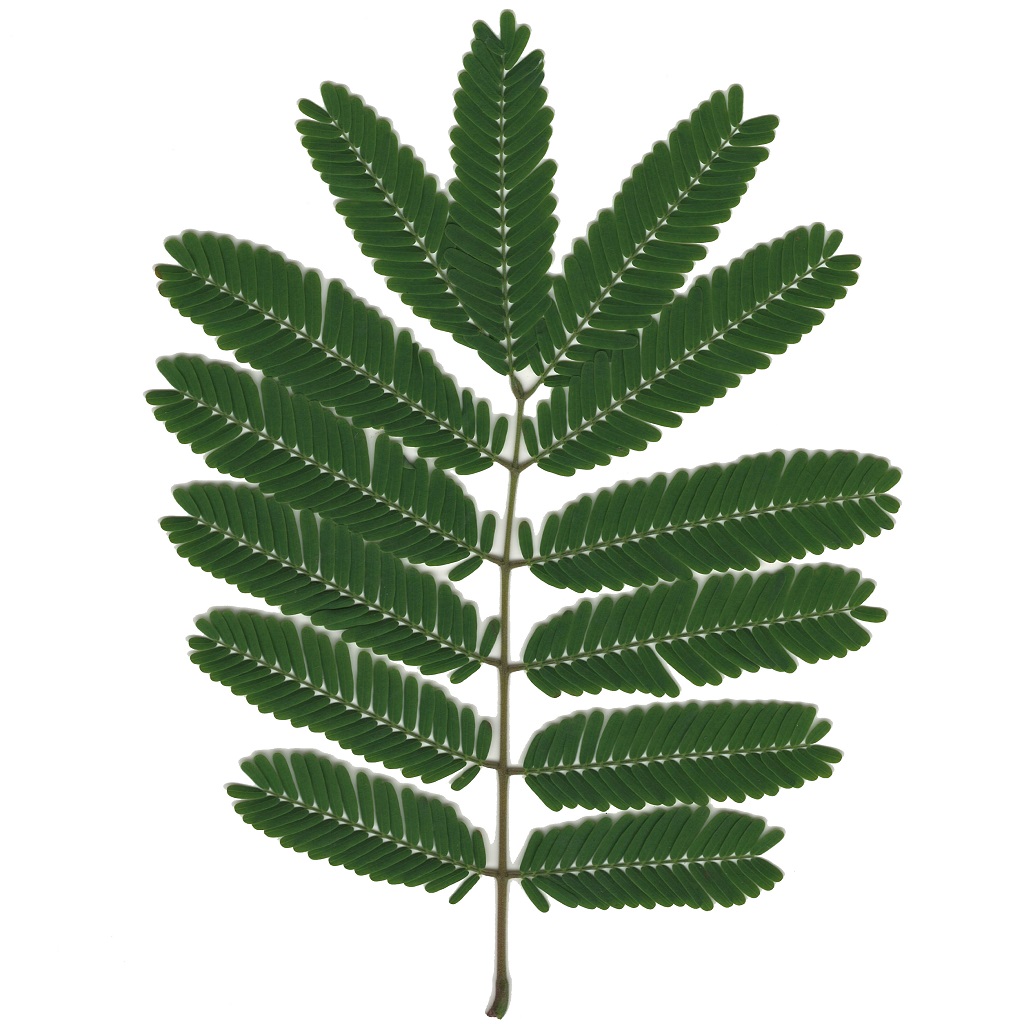

Supplement: S1 Data — (ZIP) [file pone.0293596.s001.zip › S1_data/Caesalpinia coriaria.jpg]

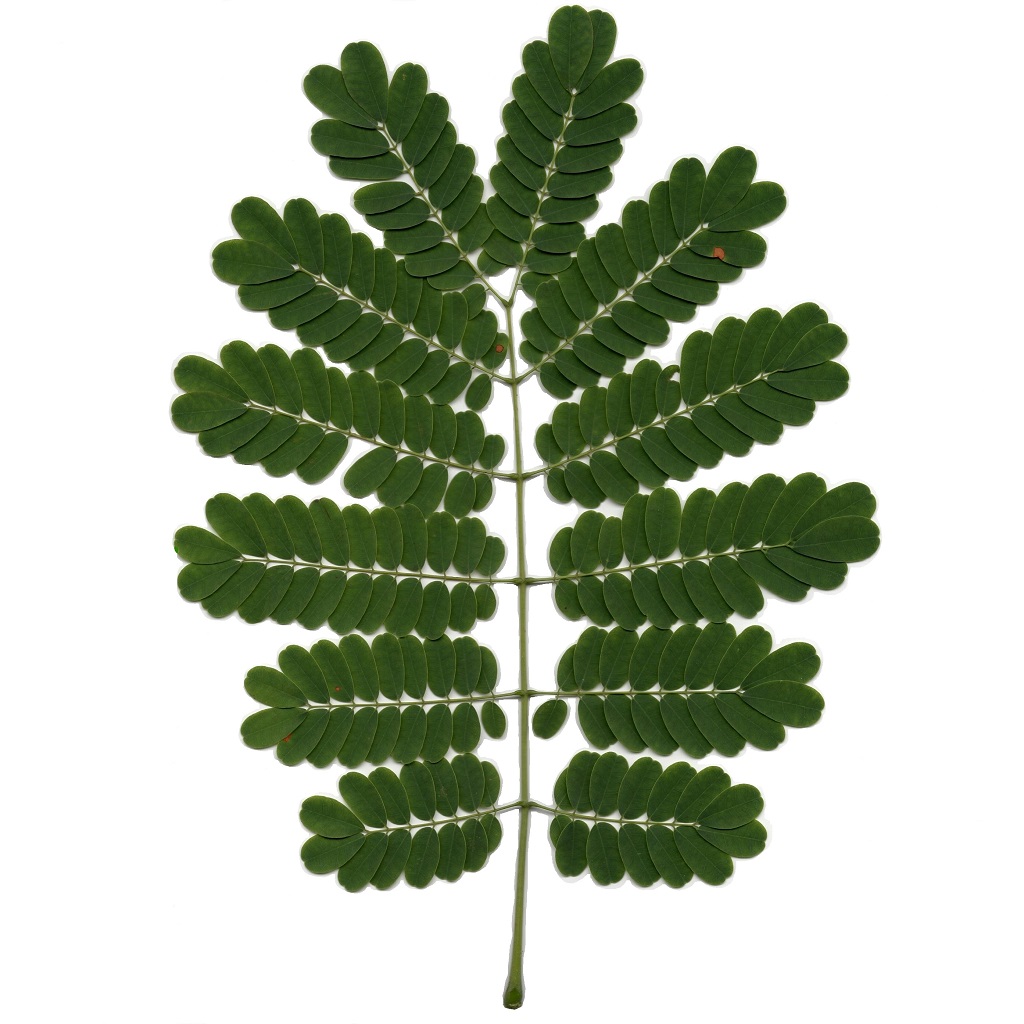

Supplement: S1 Data — (ZIP) [file pone.0293596.s001.zip › S1_data/Caesalpinia pulcherrima.jpg]

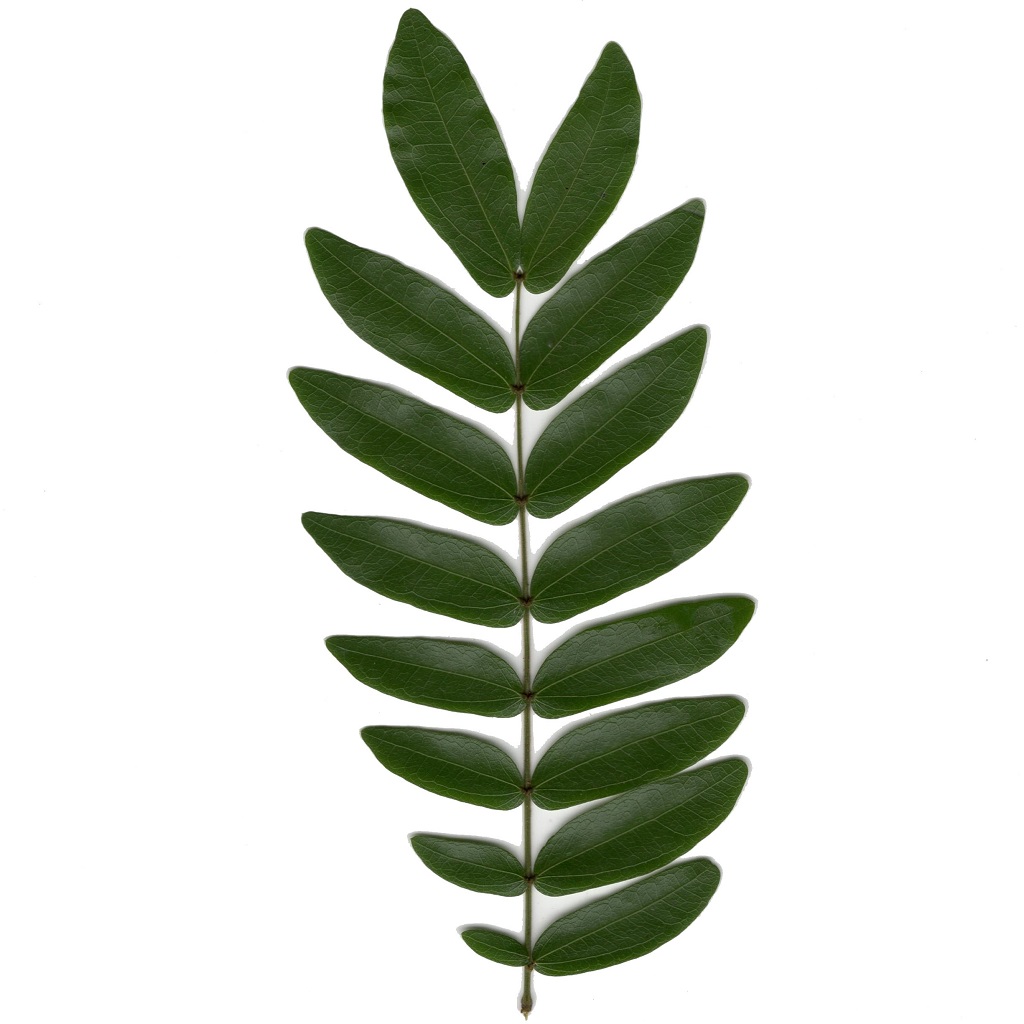

Supplement: S1 Data — (ZIP) [file pone.0293596.s001.zip › S1_data/Calliandra haematocephala.jpg]

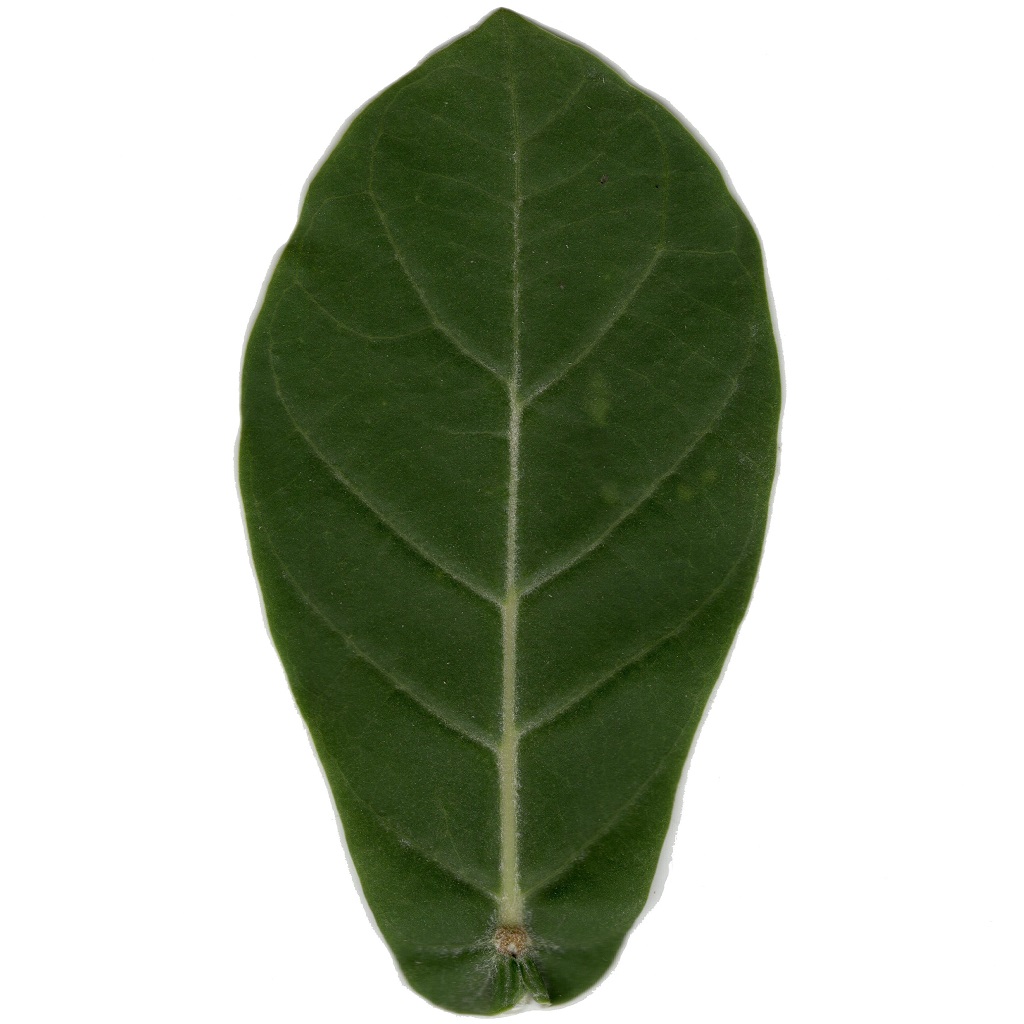

Supplement: S1 Data — (ZIP) [file pone.0293596.s001.zip › S1_data/Calotropis gigantea.jpg]

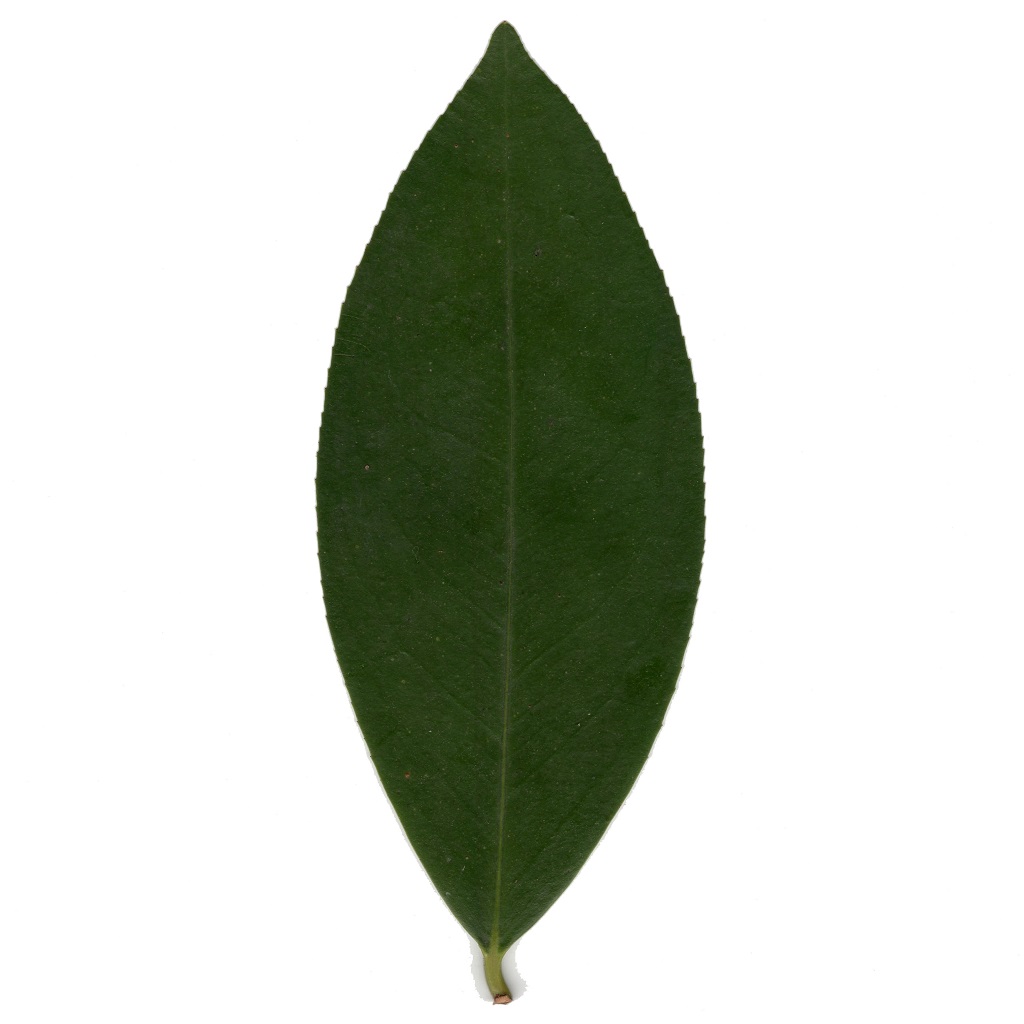

Supplement: S1 Data — (ZIP) [file pone.0293596.s001.zip › S1_data/Carallia brachiata.jpg]

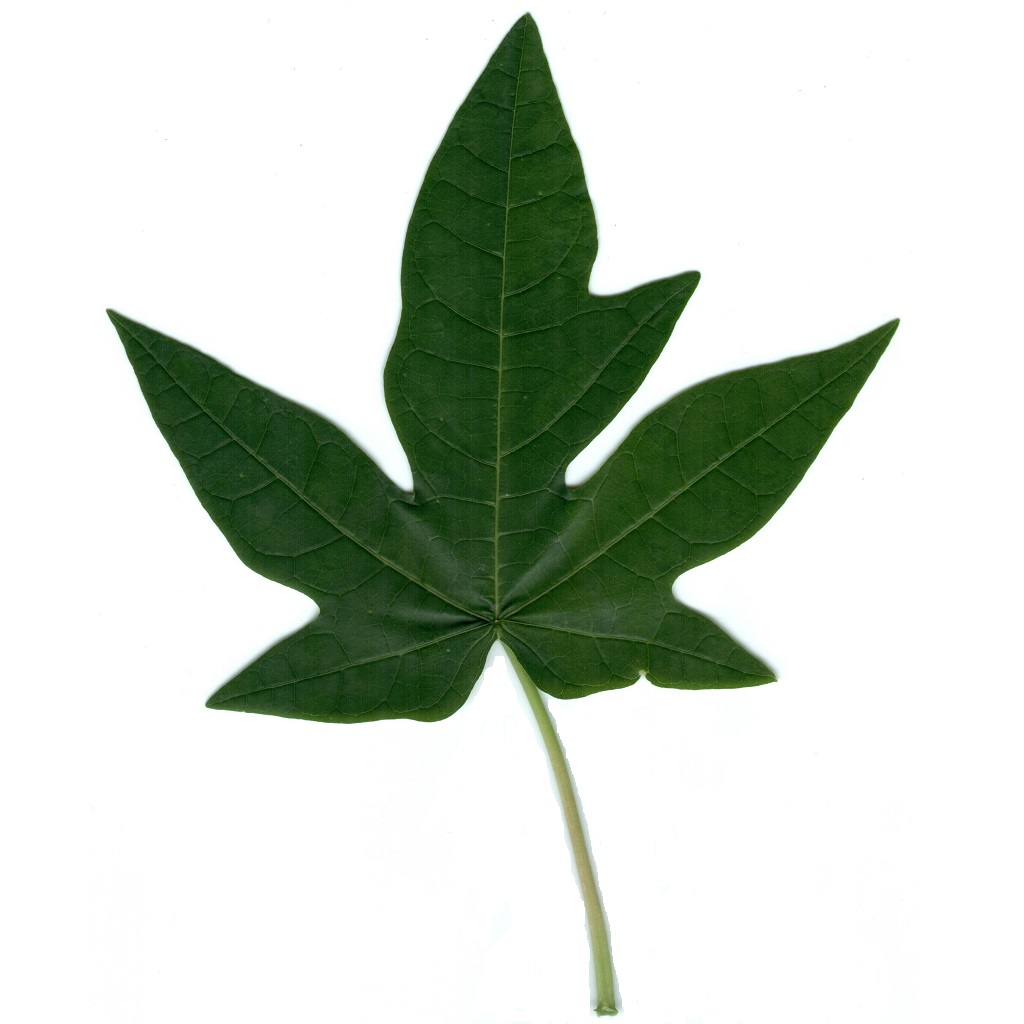

Supplement: S1 Data — (ZIP) [file pone.0293596.s001.zip › S1_data/Carica pappaya.jpg]

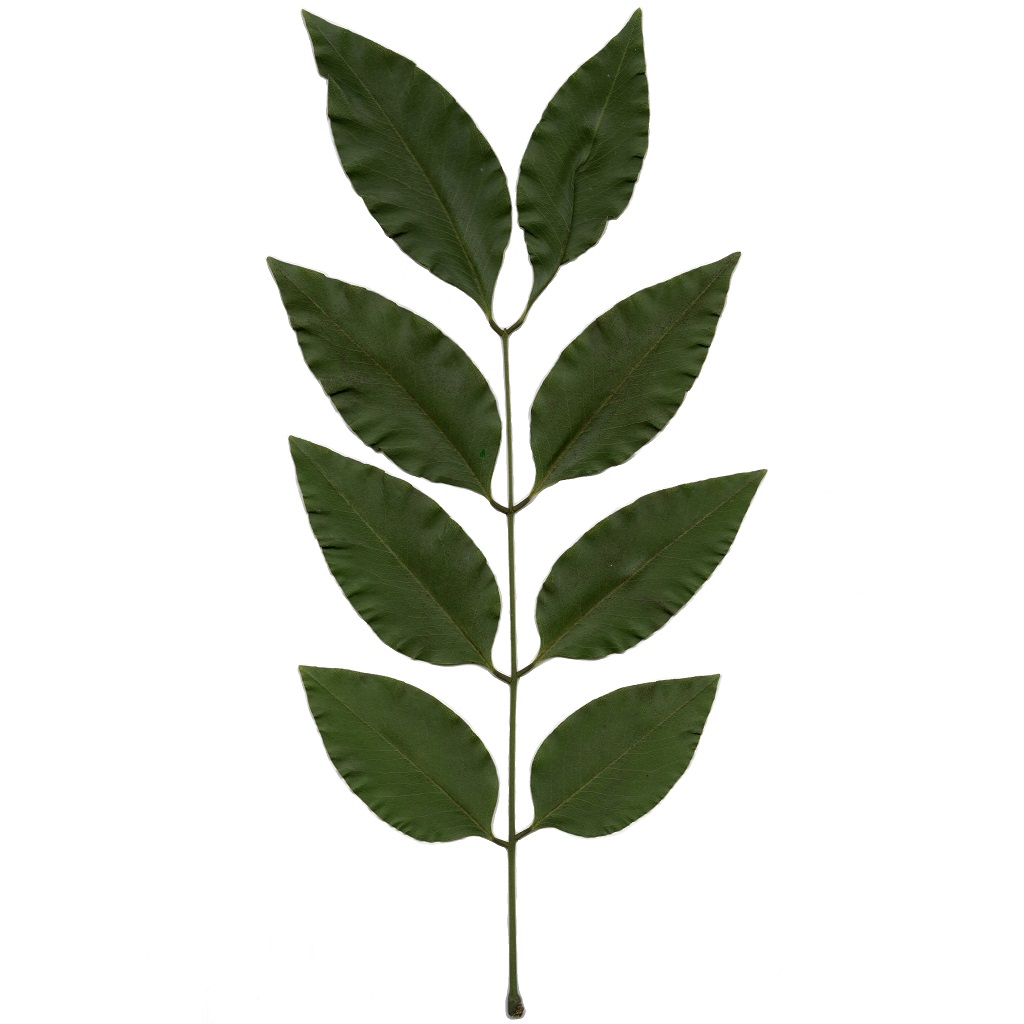

Supplement: S1 Data — (ZIP) [file pone.0293596.s001.zip › S1_data/Cassia fistula.jpg]

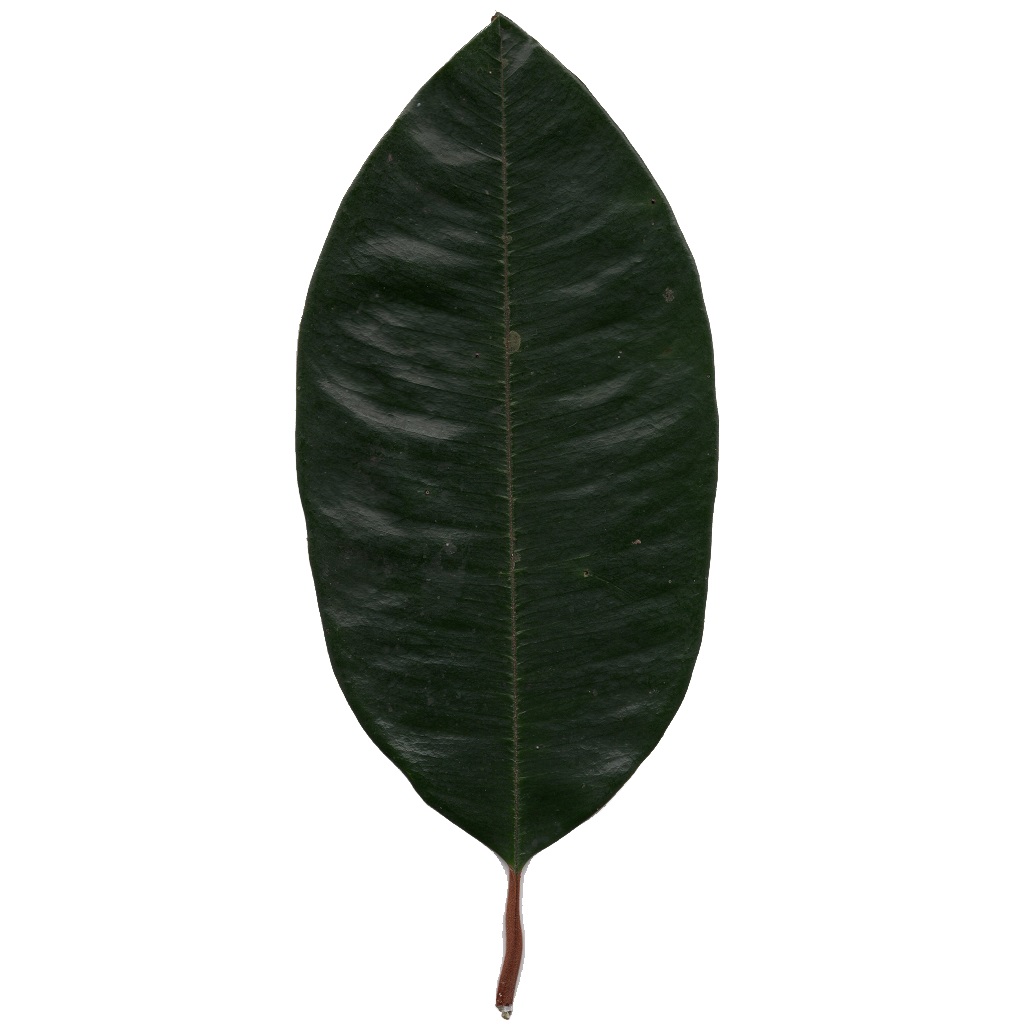

Supplement: S1 Data — (ZIP) [file pone.0293596.s001.zip › S1_data/Chrysophyllum cainito.jpg]

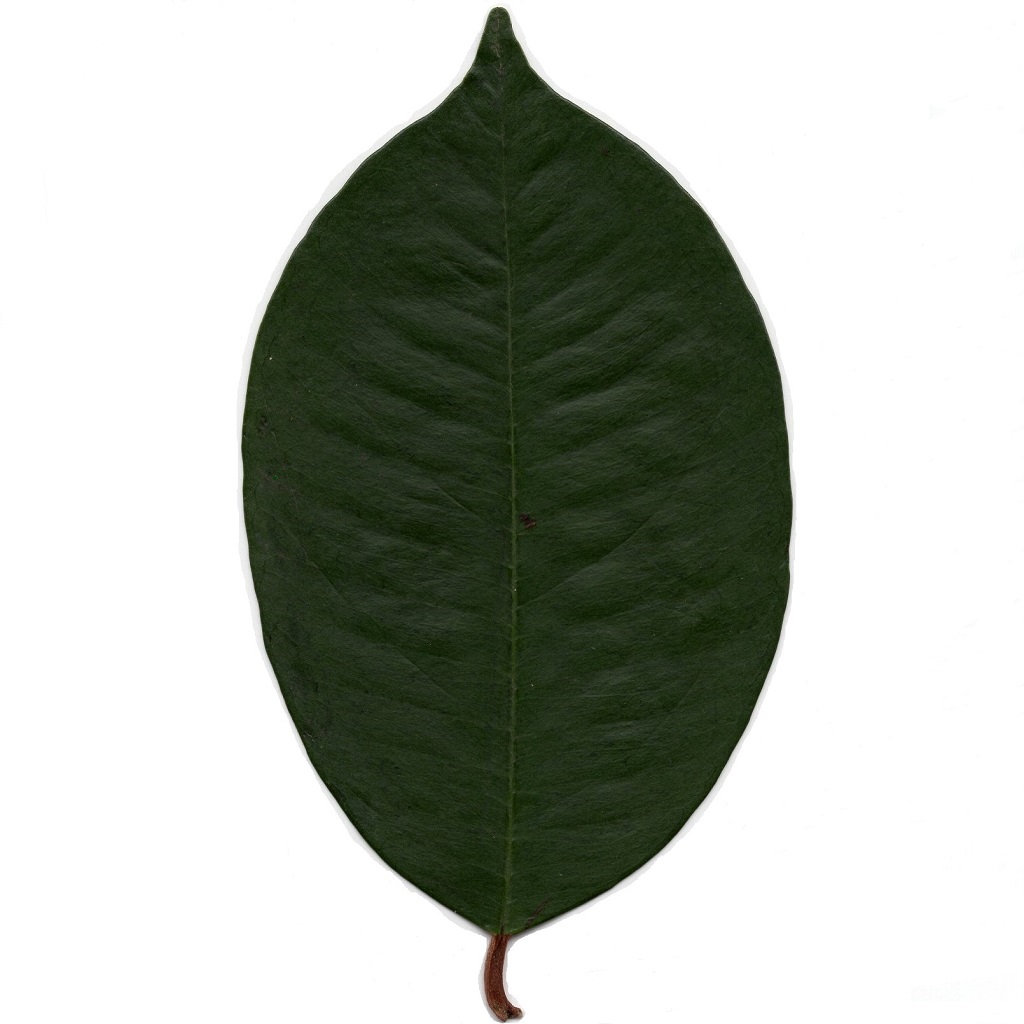

Supplement: S1 Data — (ZIP) [file pone.0293596.s001.zip › S1_data/Chrysophyllum oliviforme.jpg]

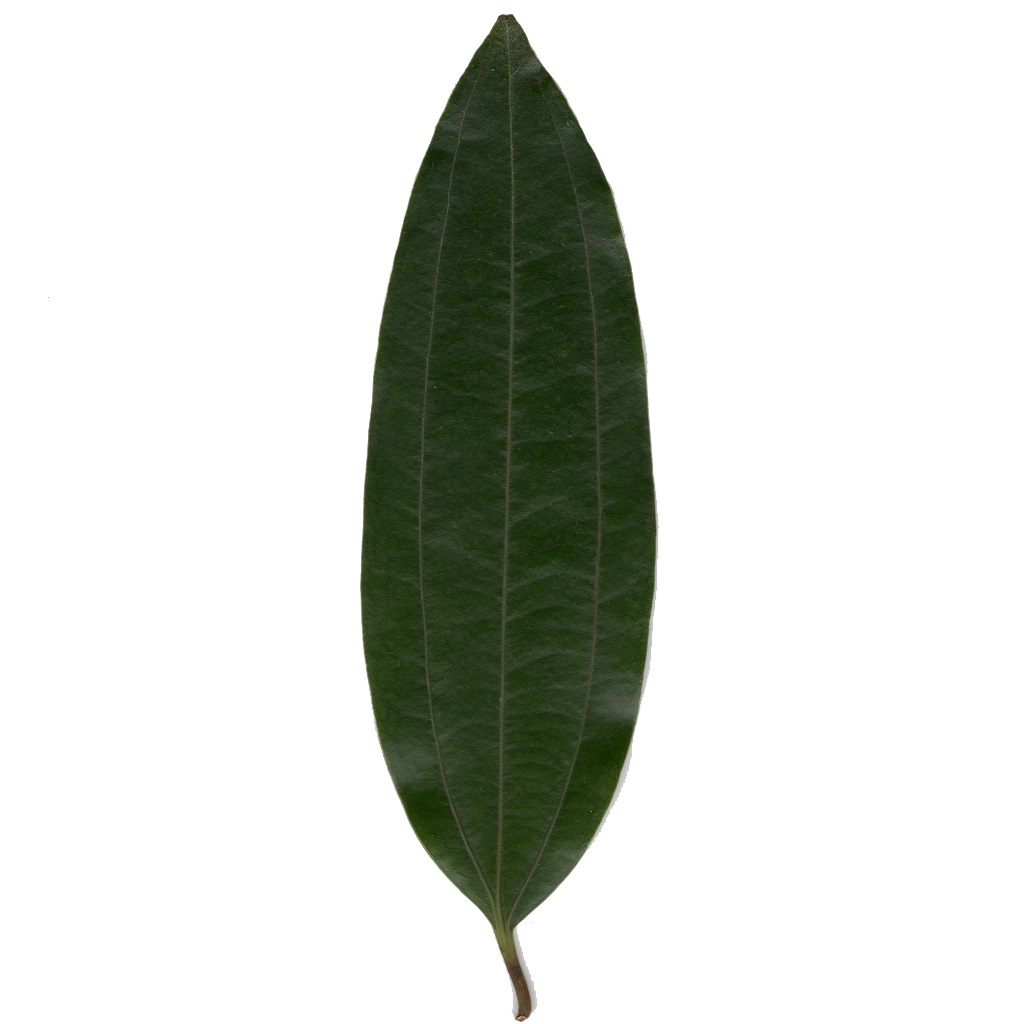

Supplement: S1 Data — (ZIP) [file pone.0293596.s001.zip › S1_data/Cinnamomum tamala.jpg]

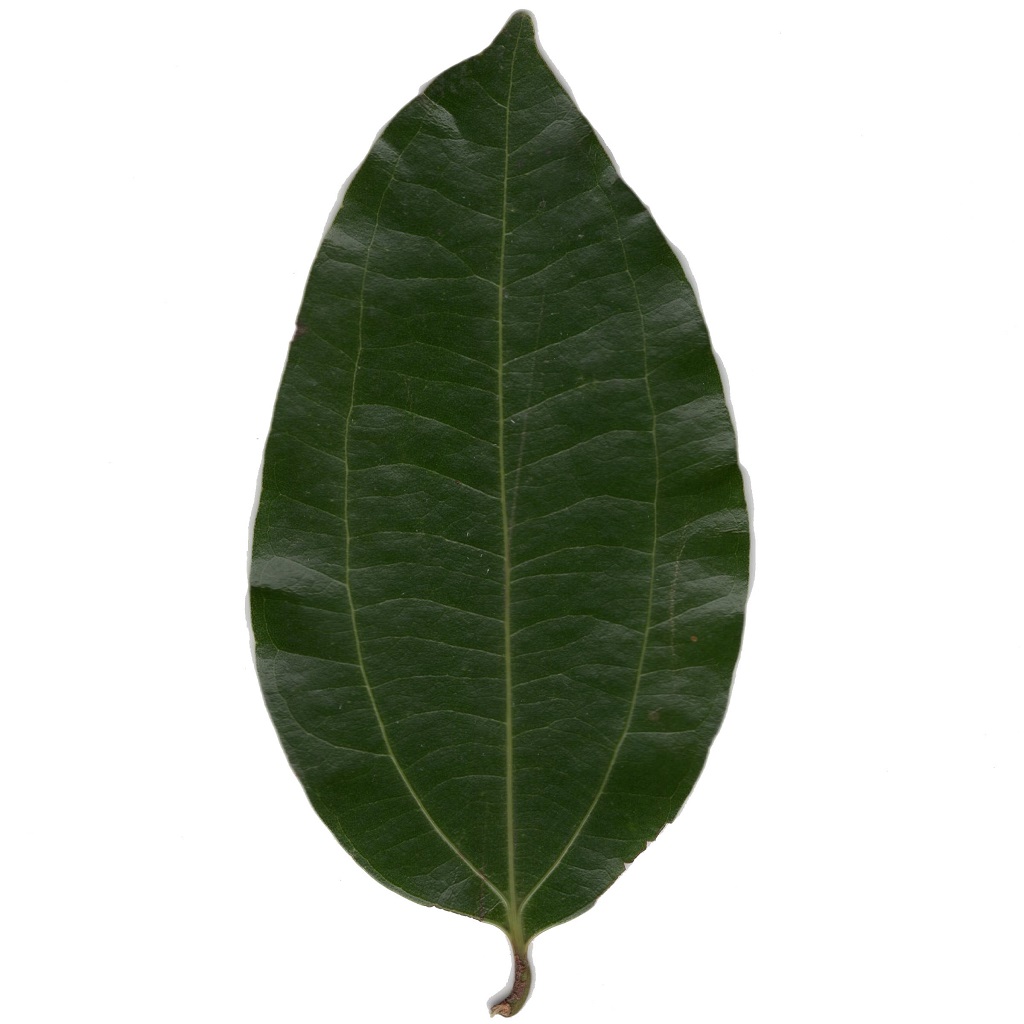

Supplement: S1 Data — (ZIP) [file pone.0293596.s001.zip › S1_data/Cinnamomum verum.jpg]

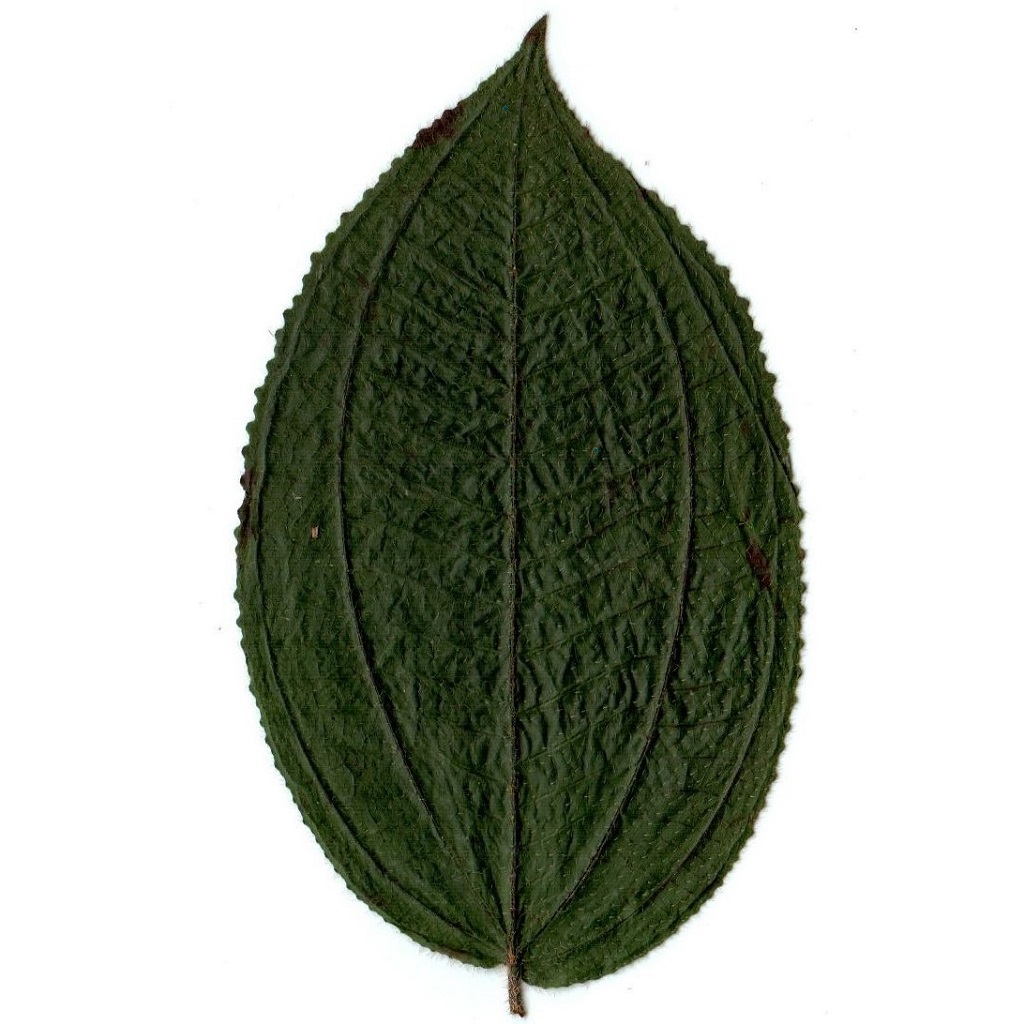

Supplement: S1 Data — (ZIP) [file pone.0293596.s001.zip › S1_data/Clidemia hirta.jpg]

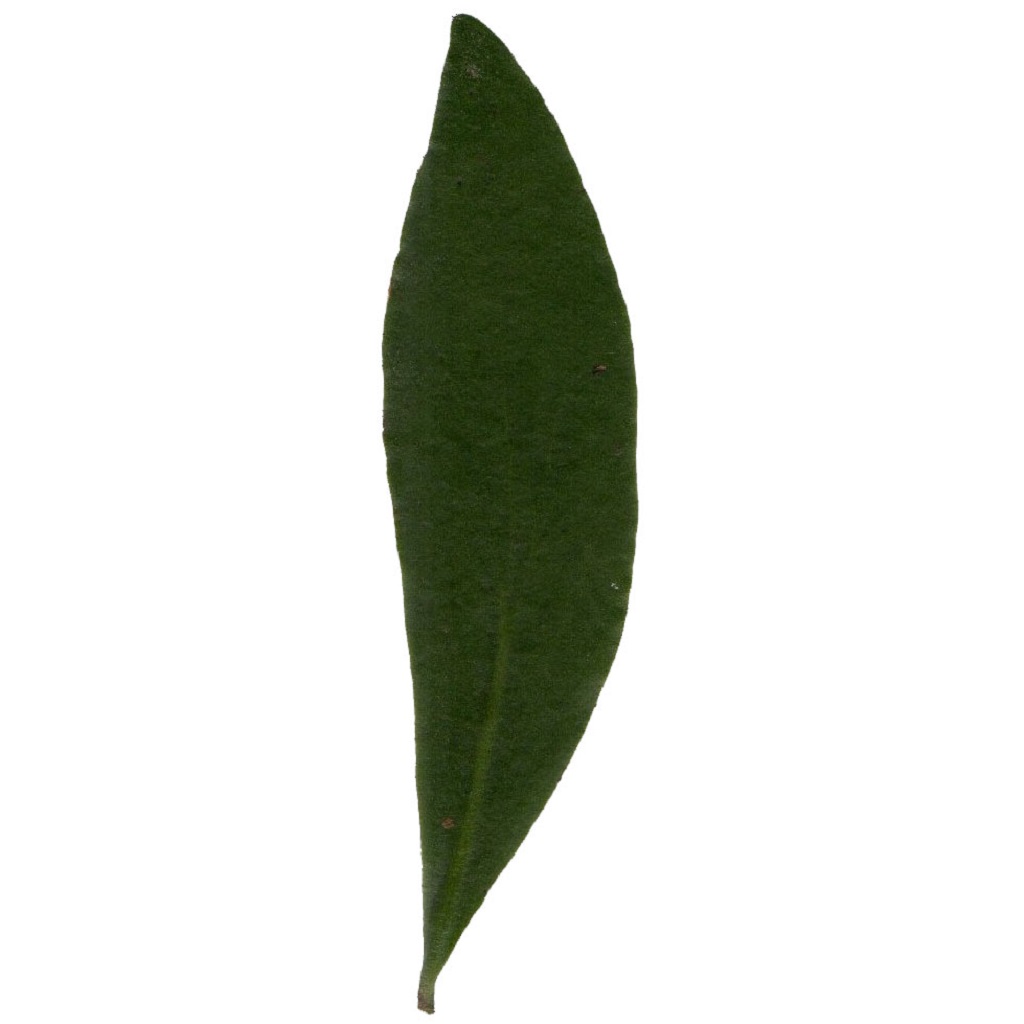

Supplement: S1 Data — (ZIP) [file pone.0293596.s001.zip › S1_data/Dendrophthoe falcata.jpg]

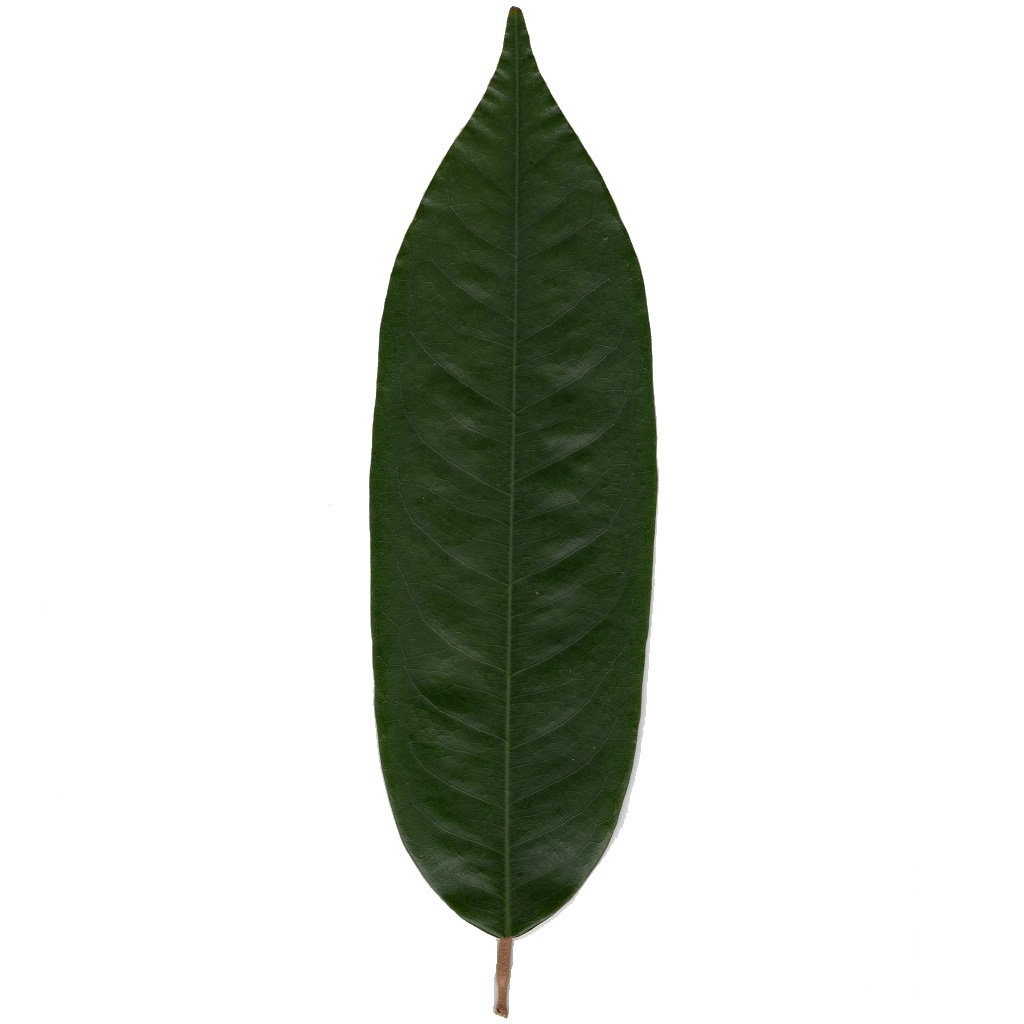

Supplement: S1 Data — (ZIP) [file pone.0293596.s001.zip › S1_data/Durio zibethinus.jpg]

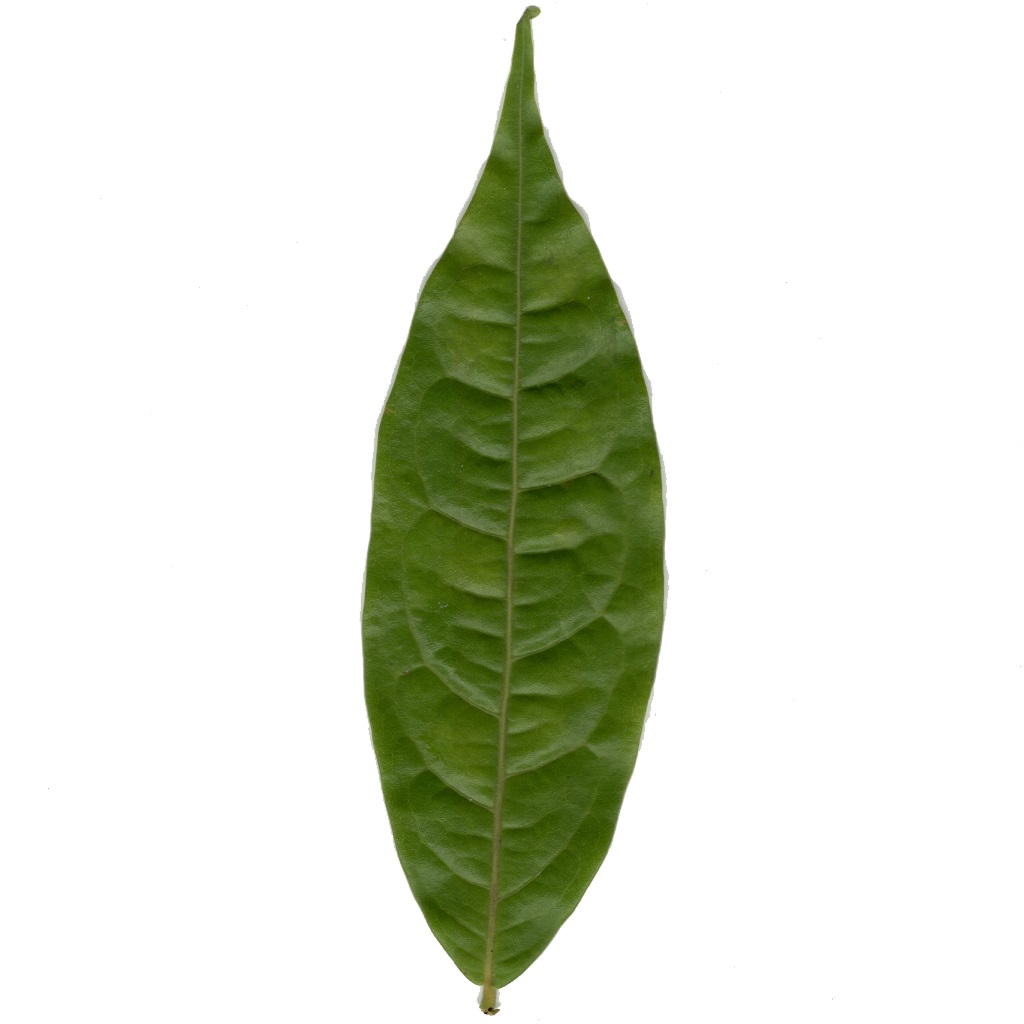

Supplement: S1 Data — (ZIP) [file pone.0293596.s001.zip › S1_data/Eugenia victoriana.jpg]

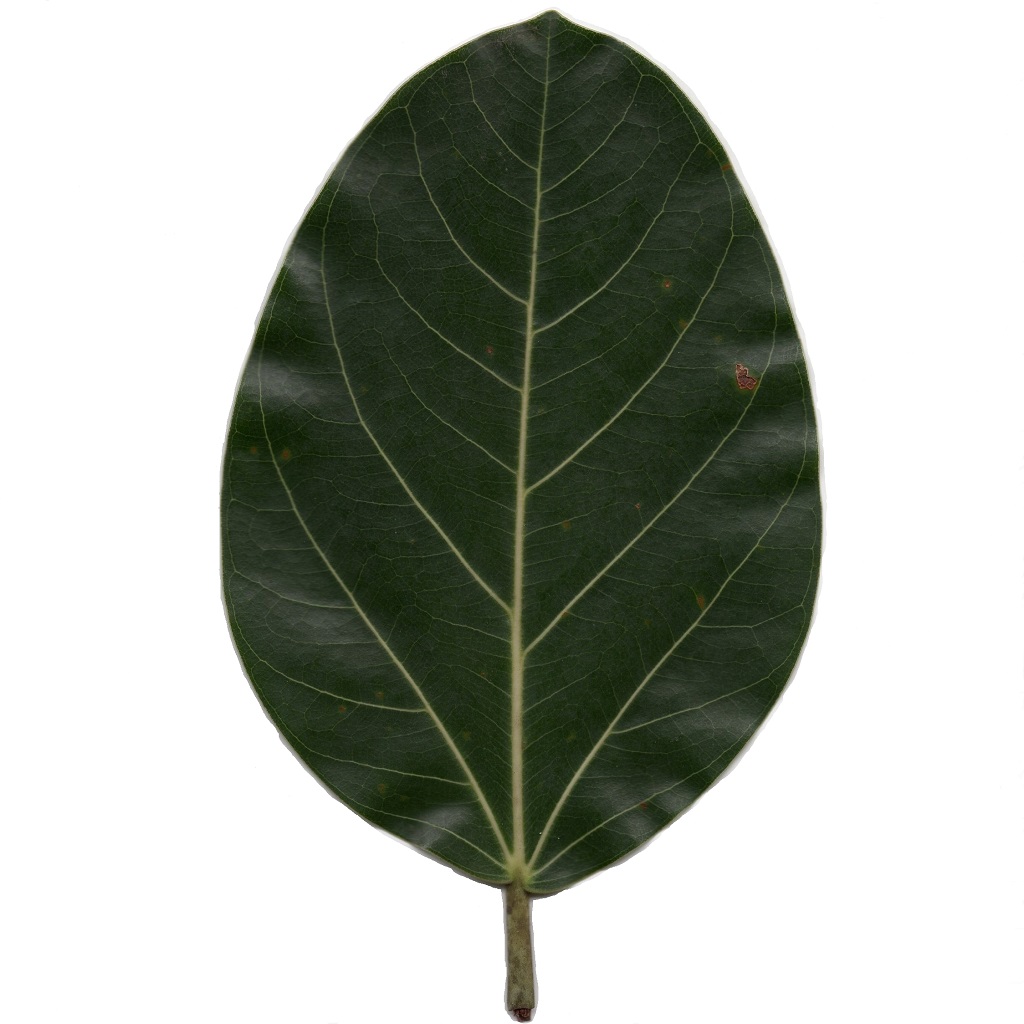

Supplement: S1 Data — (ZIP) [file pone.0293596.s001.zip › S1_data/Ficus benghalensis.jpg]

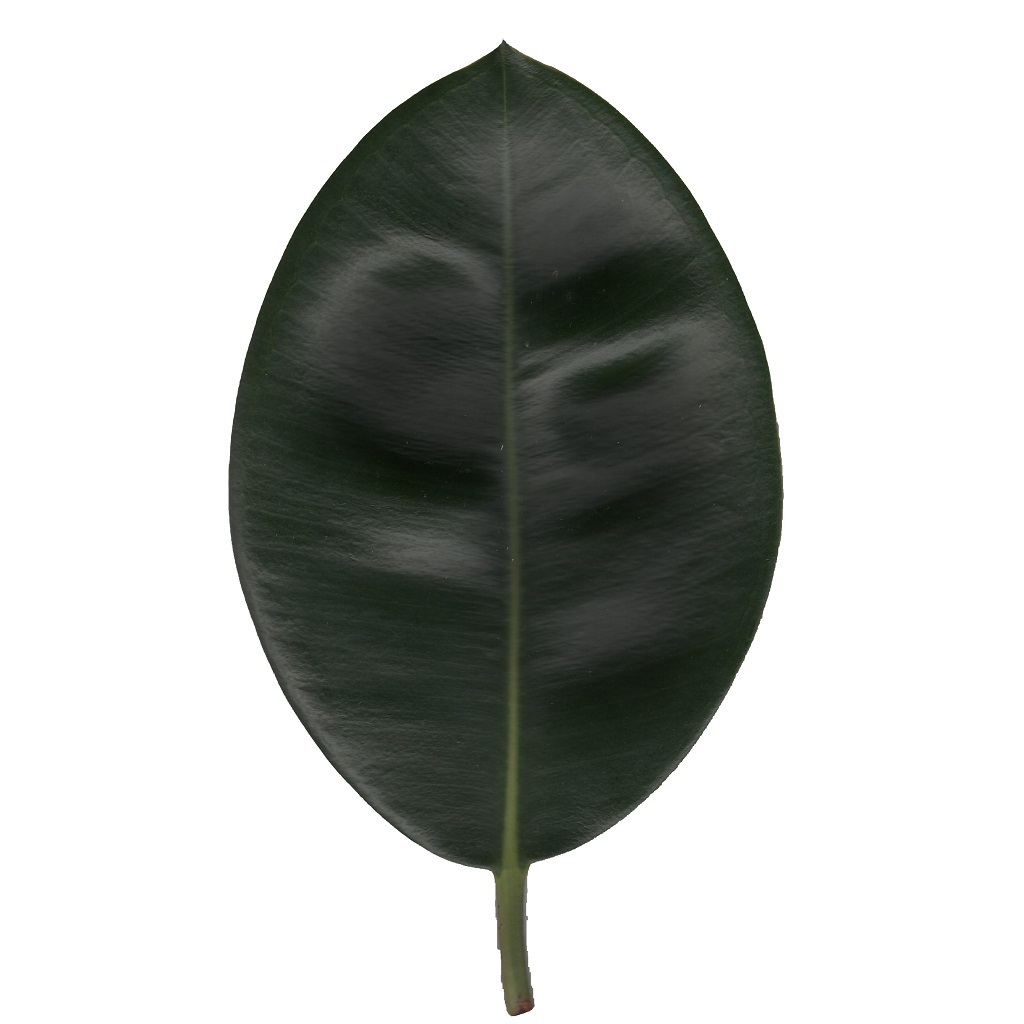

Supplement: S1 Data — (ZIP) [file pone.0293596.s001.zip › S1_data/Ficus elastica.jpg]

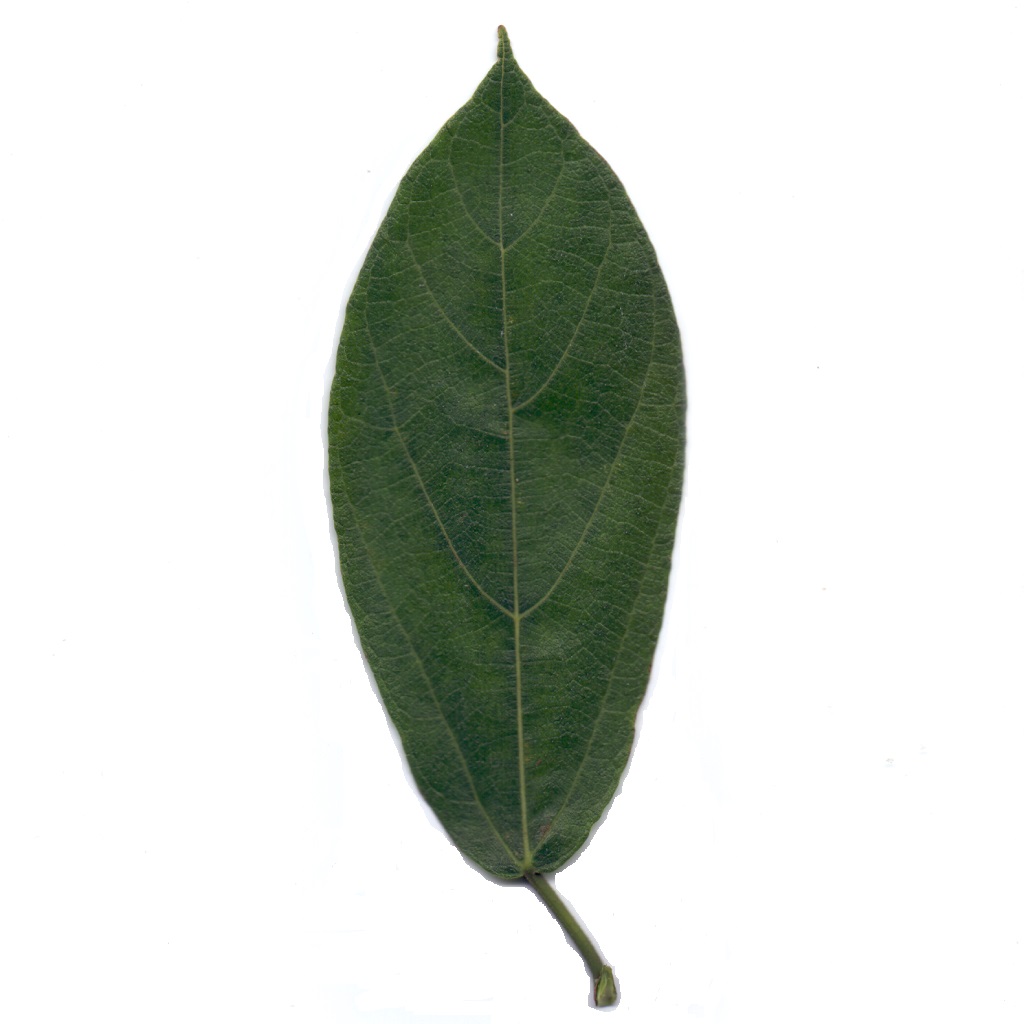

Supplement: S1 Data — (ZIP) [file pone.0293596.s001.zip › S1_data/Ficus exasperata.jpg]

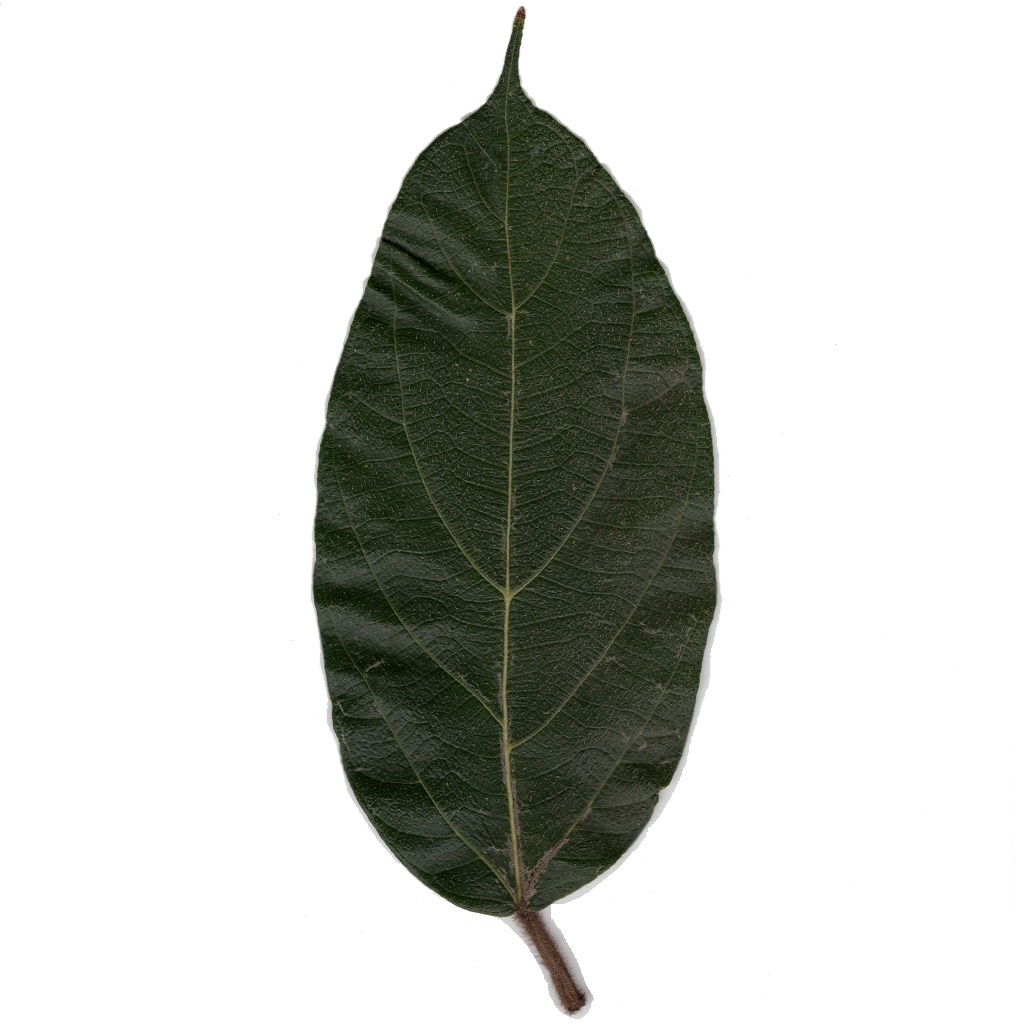

Supplement: S1 Data — (ZIP) [file pone.0293596.s001.zip › S1_data/Ficus hispida.jpg]

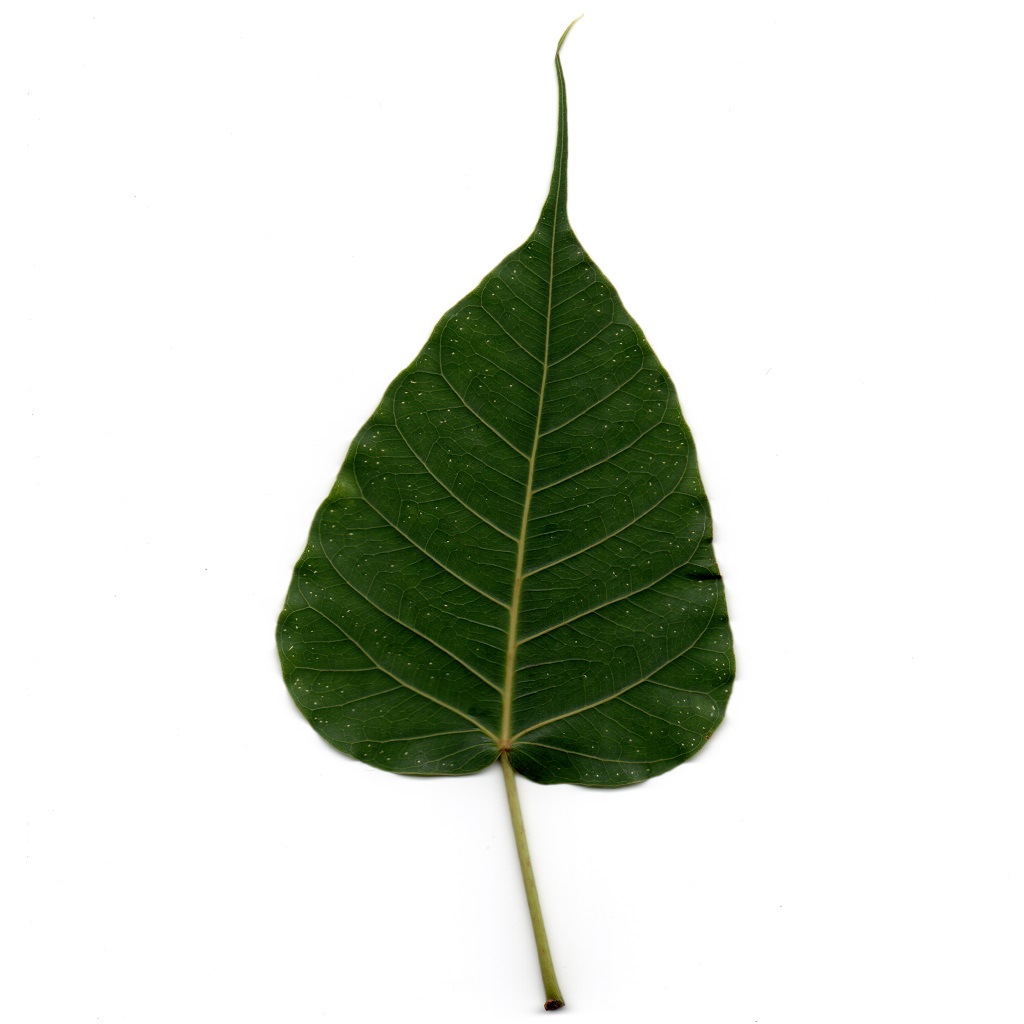

Supplement: S1 Data — (ZIP) [file pone.0293596.s001.zip › S1_data/ficus religosa.jpg]

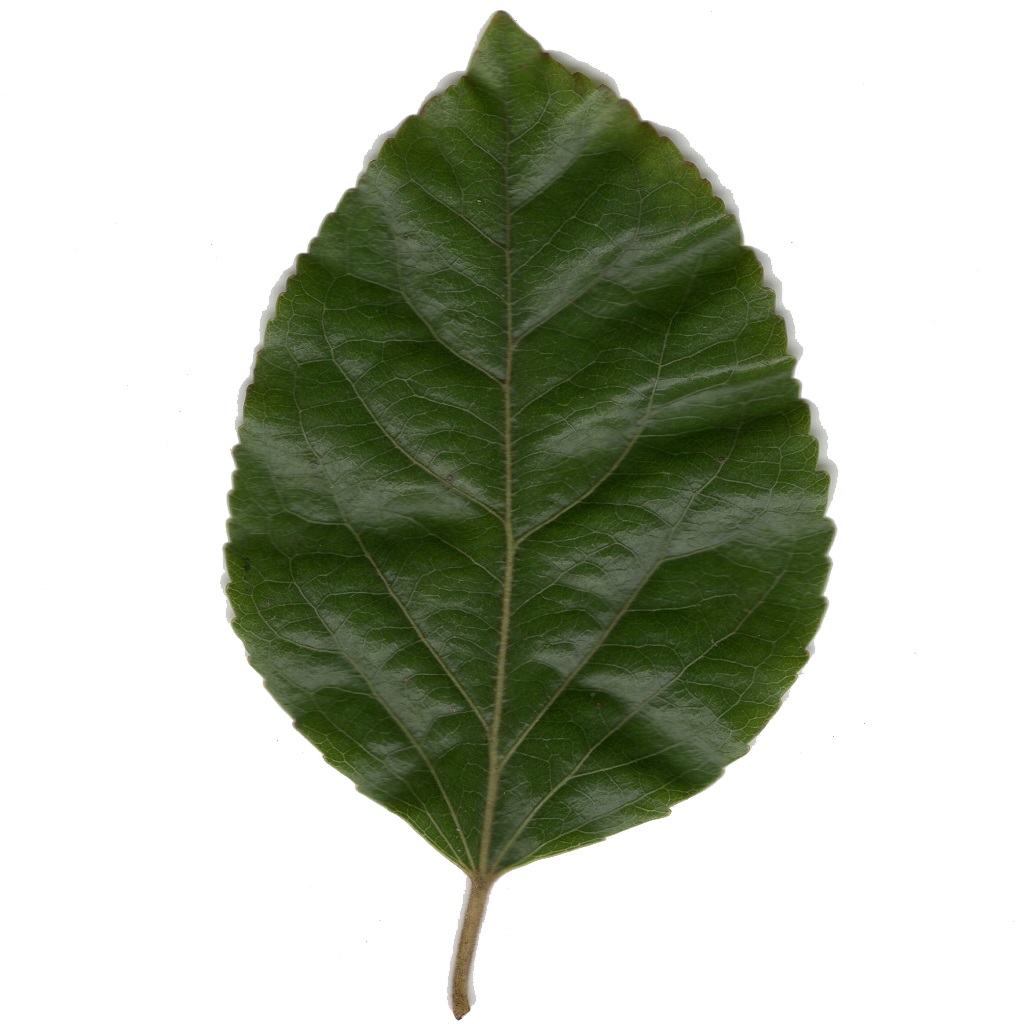

Supplement: S1 Data — (ZIP) [file pone.0293596.s001.zip › S1_data/Flacourtia jangomas.jpg]

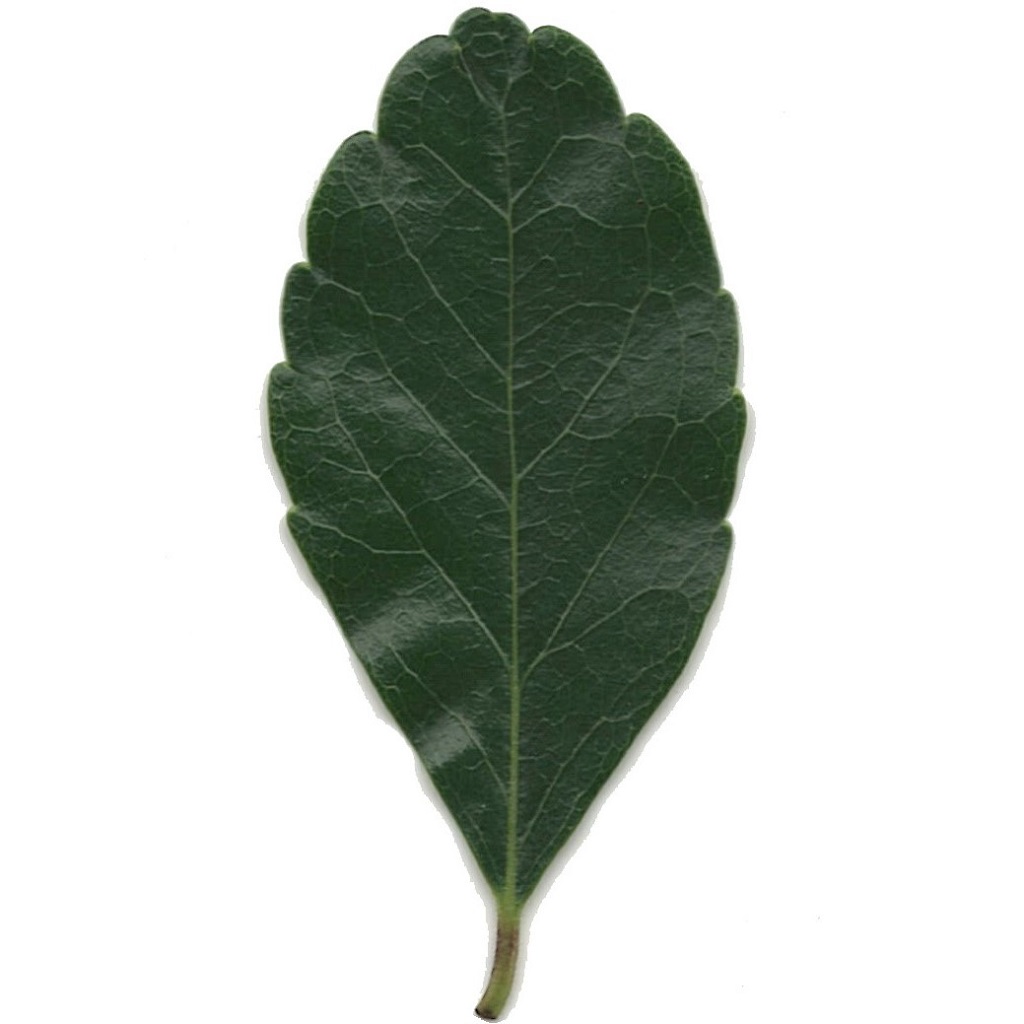

Supplement: S1 Data — (ZIP) [file pone.0293596.s001.zip › S1_data/Flacourtia sepiaria.jpg]

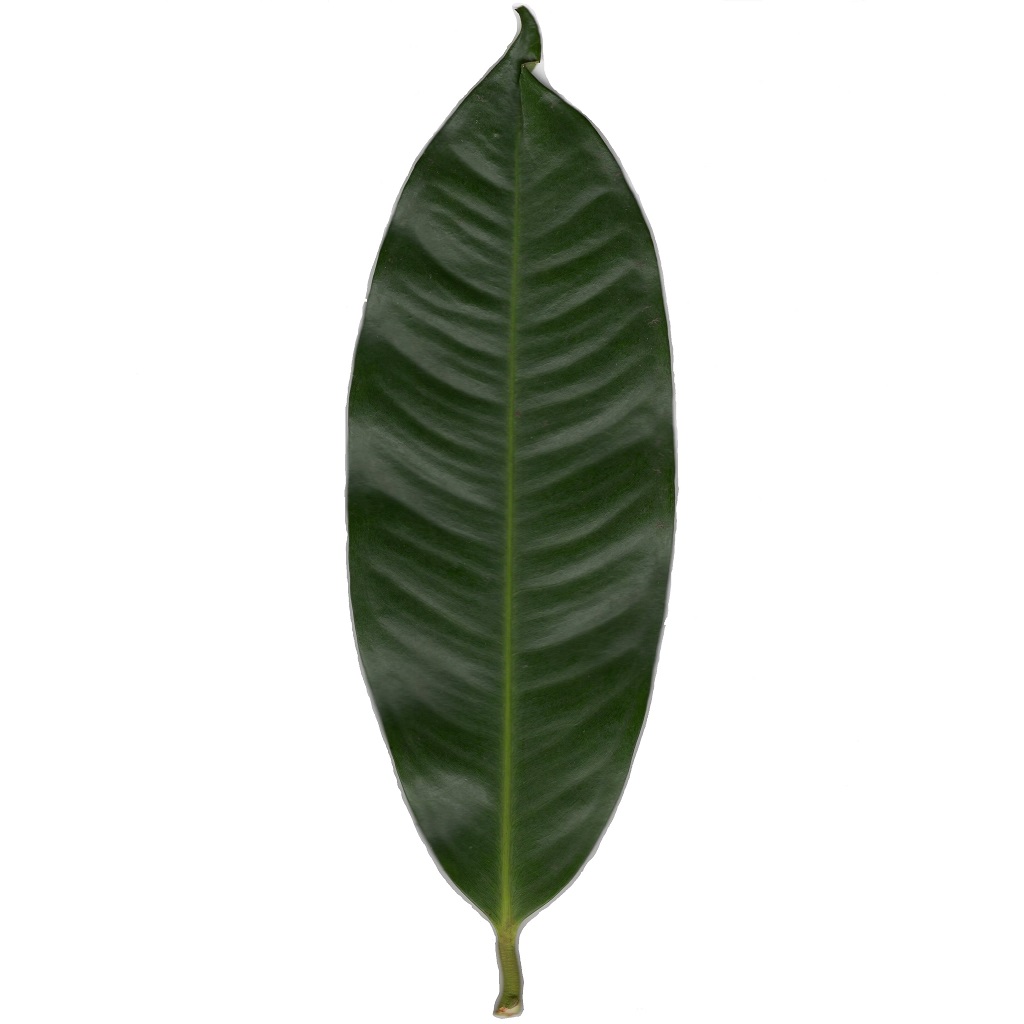

Supplement: S1 Data — (ZIP) [file pone.0293596.s001.zip › S1_data/Garcinia mangostana.jpg]

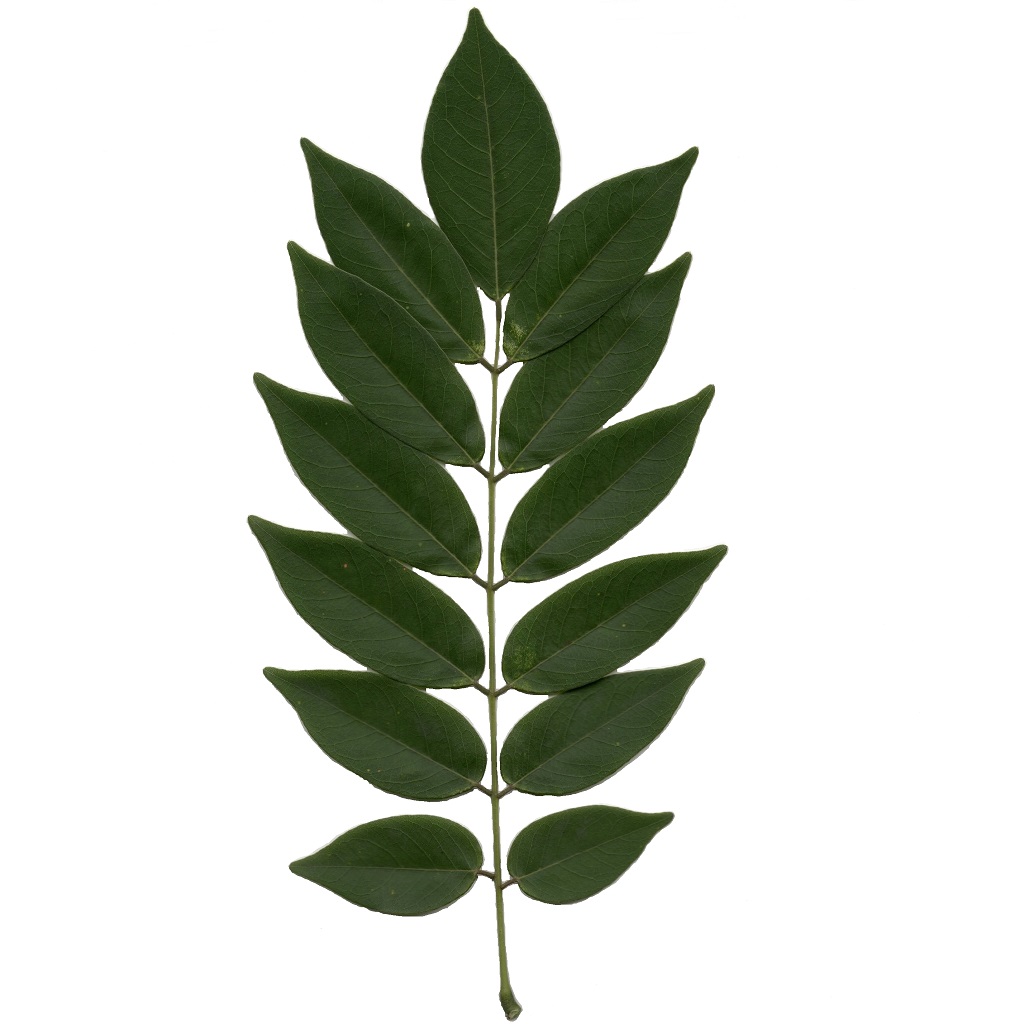

Supplement: S1 Data — (ZIP) [file pone.0293596.s001.zip › S1_data/Gliricidia sepium.jpg]

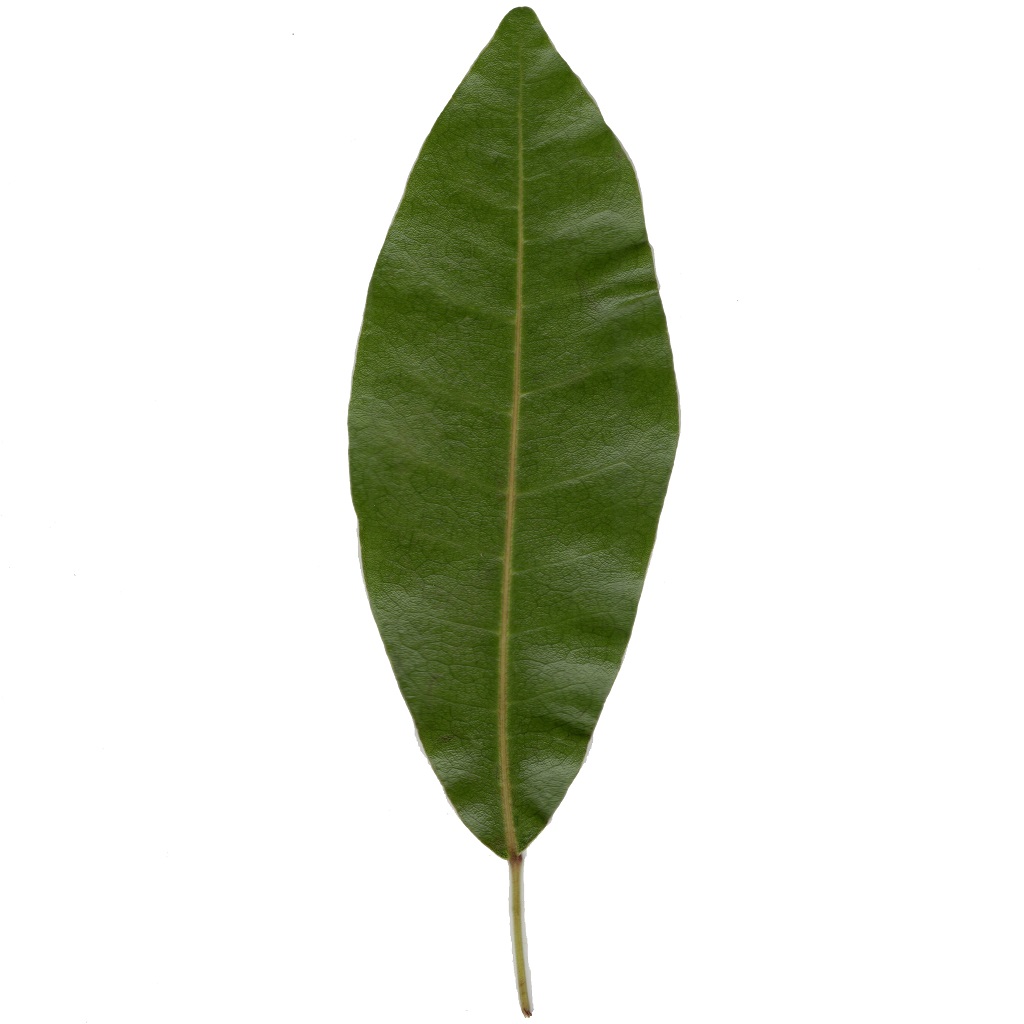

Supplement: S1 Data — (ZIP) [file pone.0293596.s001.zip › S1_data/Handroanthus impetiginosus.jpg]

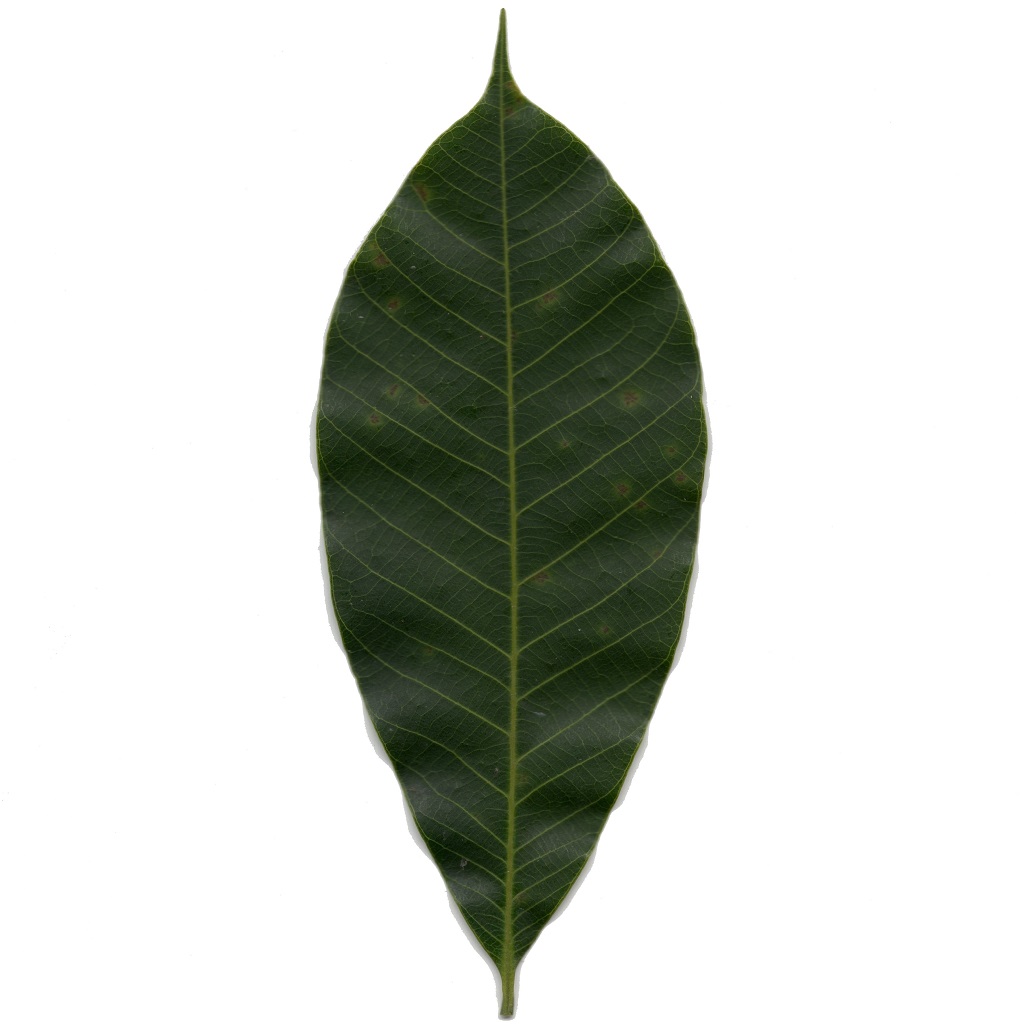

Supplement: S1 Data — (ZIP) [file pone.0293596.s001.zip › S1_data/Hevea brasiliensis.jpg]

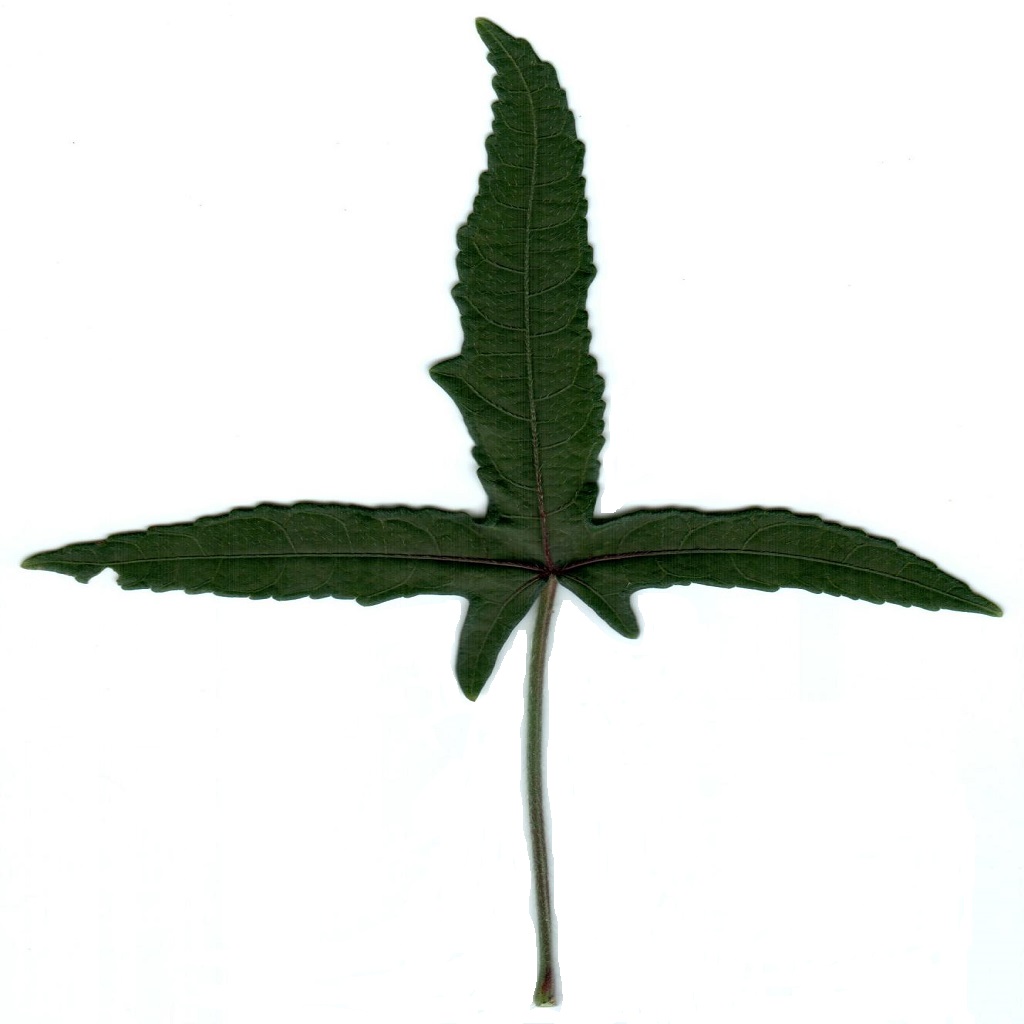

Supplement: S1 Data — (ZIP) [file pone.0293596.s001.zip › S1_data/Hibiscus cannabinus.jpg]

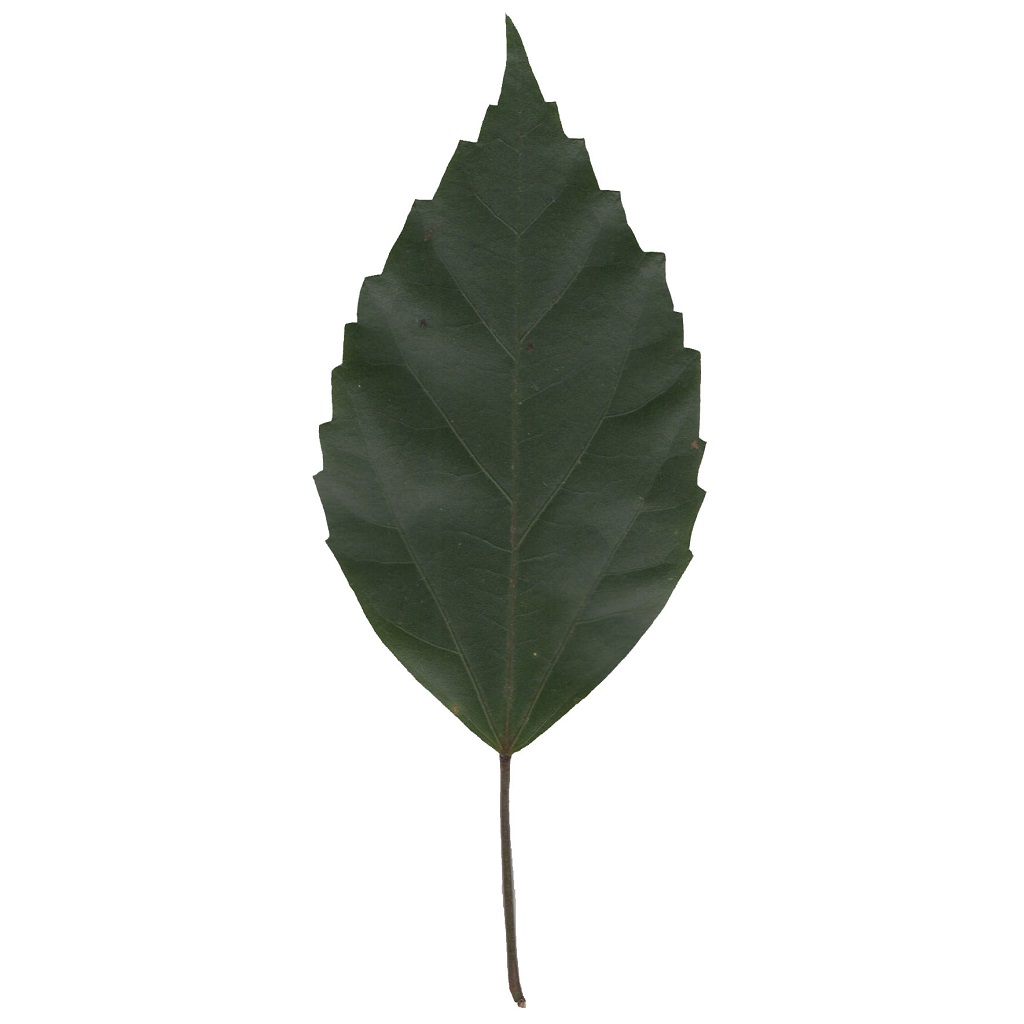

Supplement: S1 Data — (ZIP) [file pone.0293596.s001.zip › S1_data/Hibiscus rosa-sinensis.jpg]

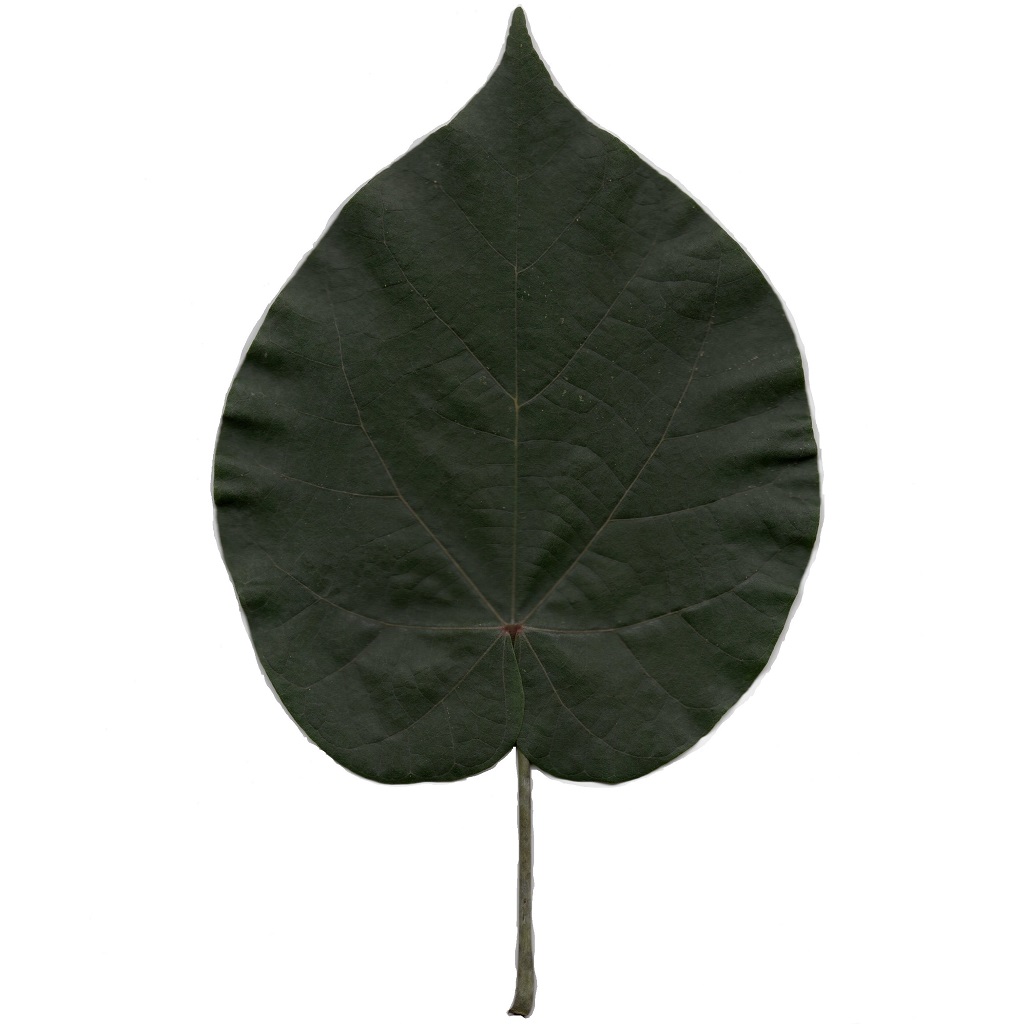

Supplement: S1 Data — (ZIP) [file pone.0293596.s001.zip › S1_data/Hibiscus tiliaceus.jpg]

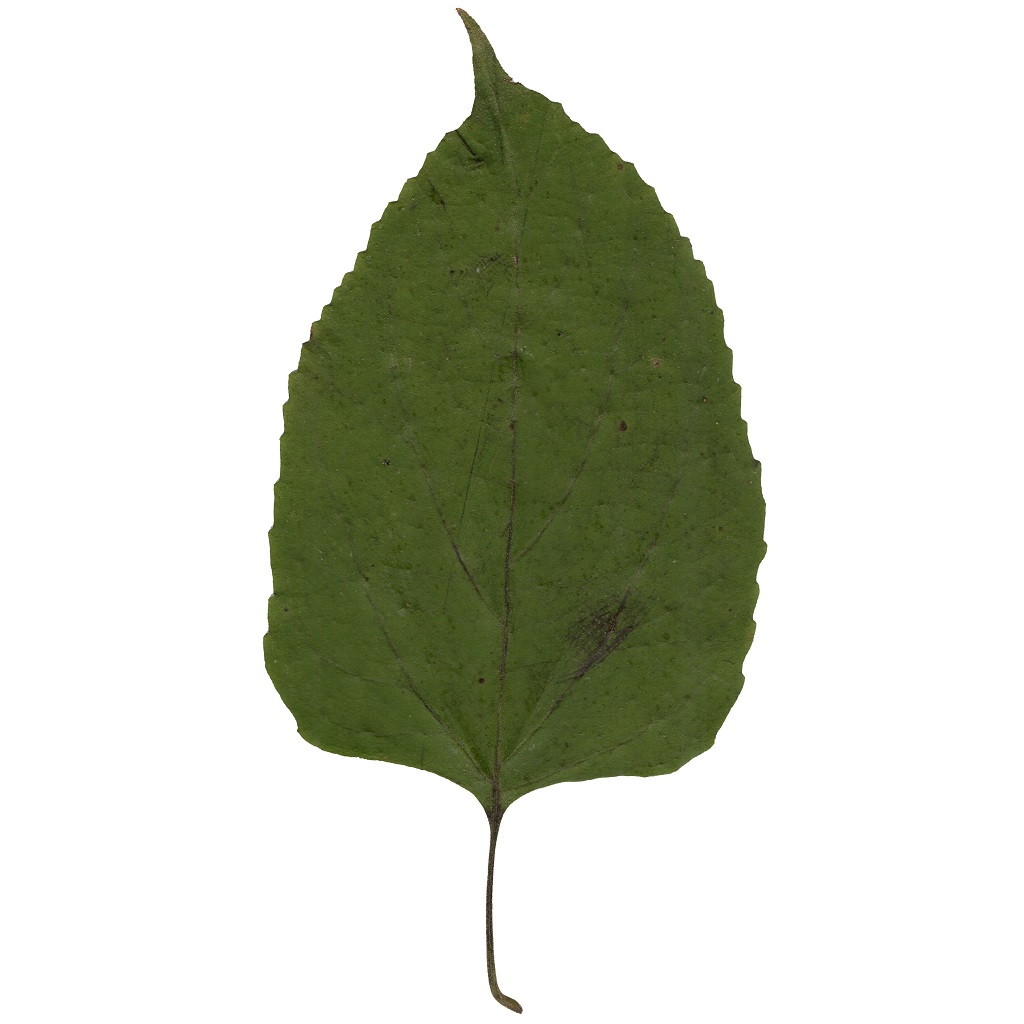

Supplement: S1 Data — (ZIP) [file pone.0293596.s001.zip › S1_data/Holmskioldia sanguinea.jpg]

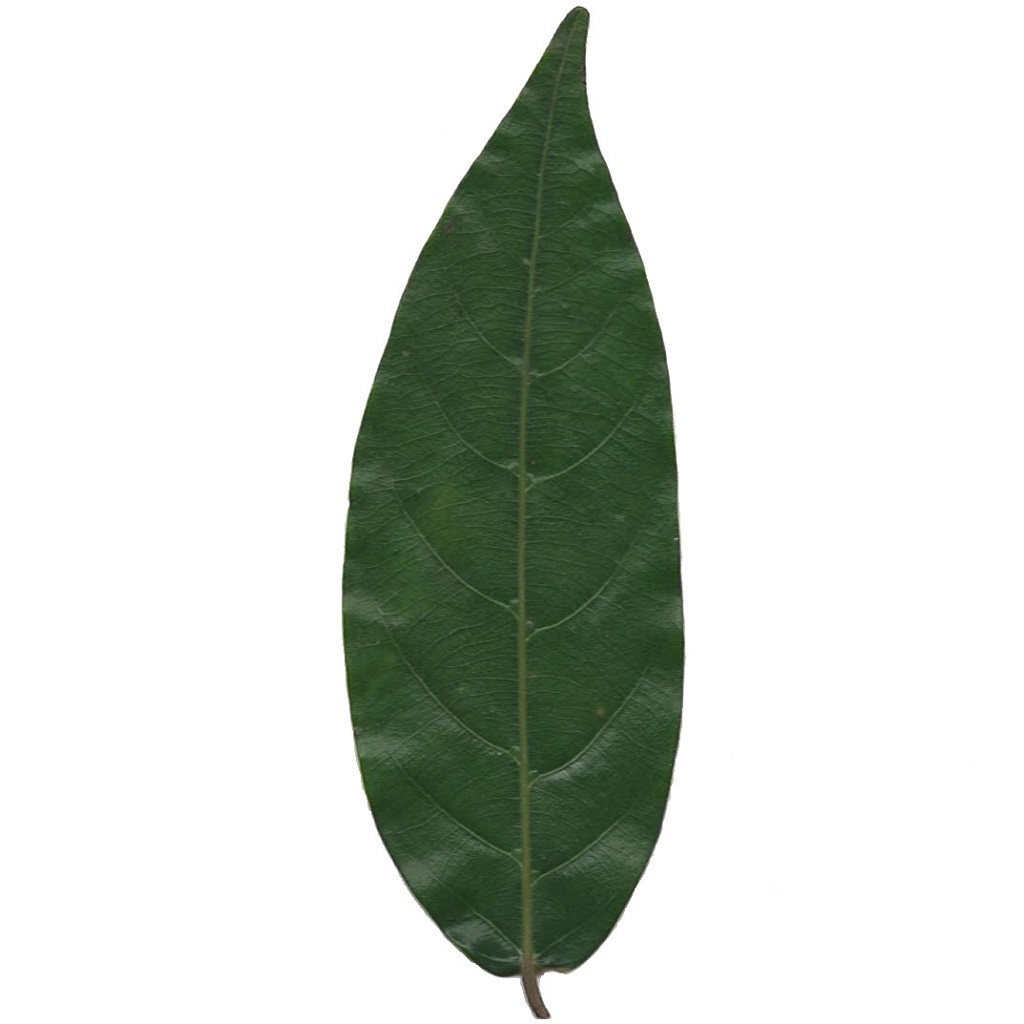

Supplement: S1 Data — (ZIP) [file pone.0293596.s001.zip › S1_data/Hopea parviflora.jpg]

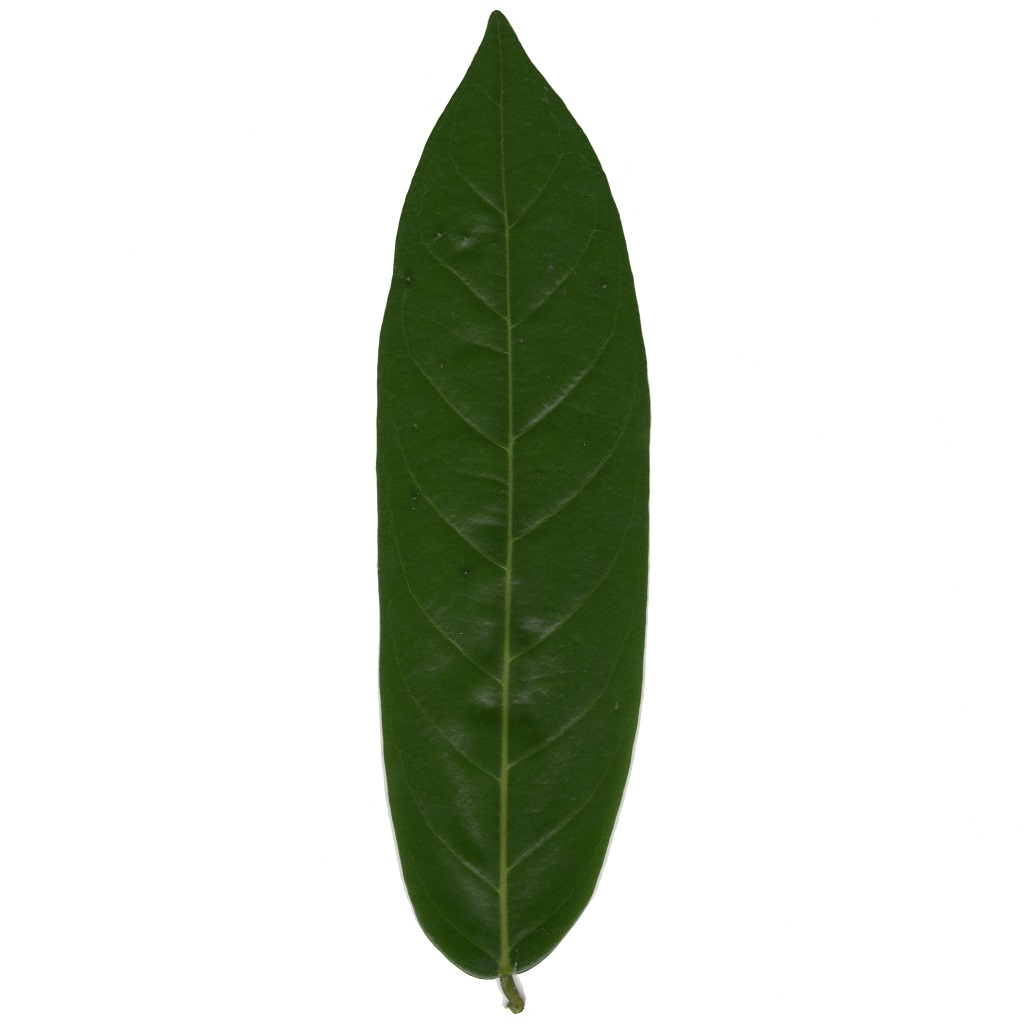

Supplement: S1 Data — (ZIP) [file pone.0293596.s001.zip › S1_data/Hopea ponga.jpg]

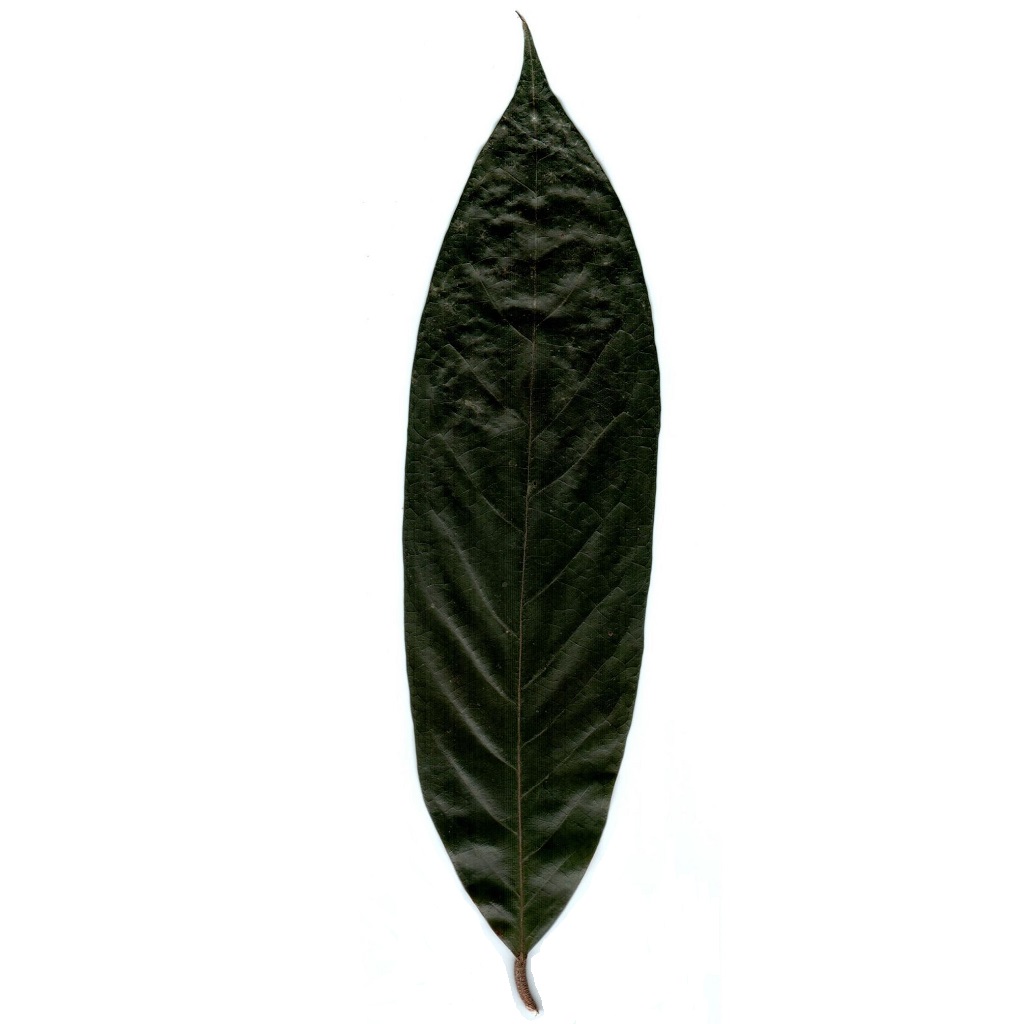

Supplement: S1 Data — (ZIP) [file pone.0293596.s001.zip › S1_data/Hydnocarpus Pentandra.jpg]

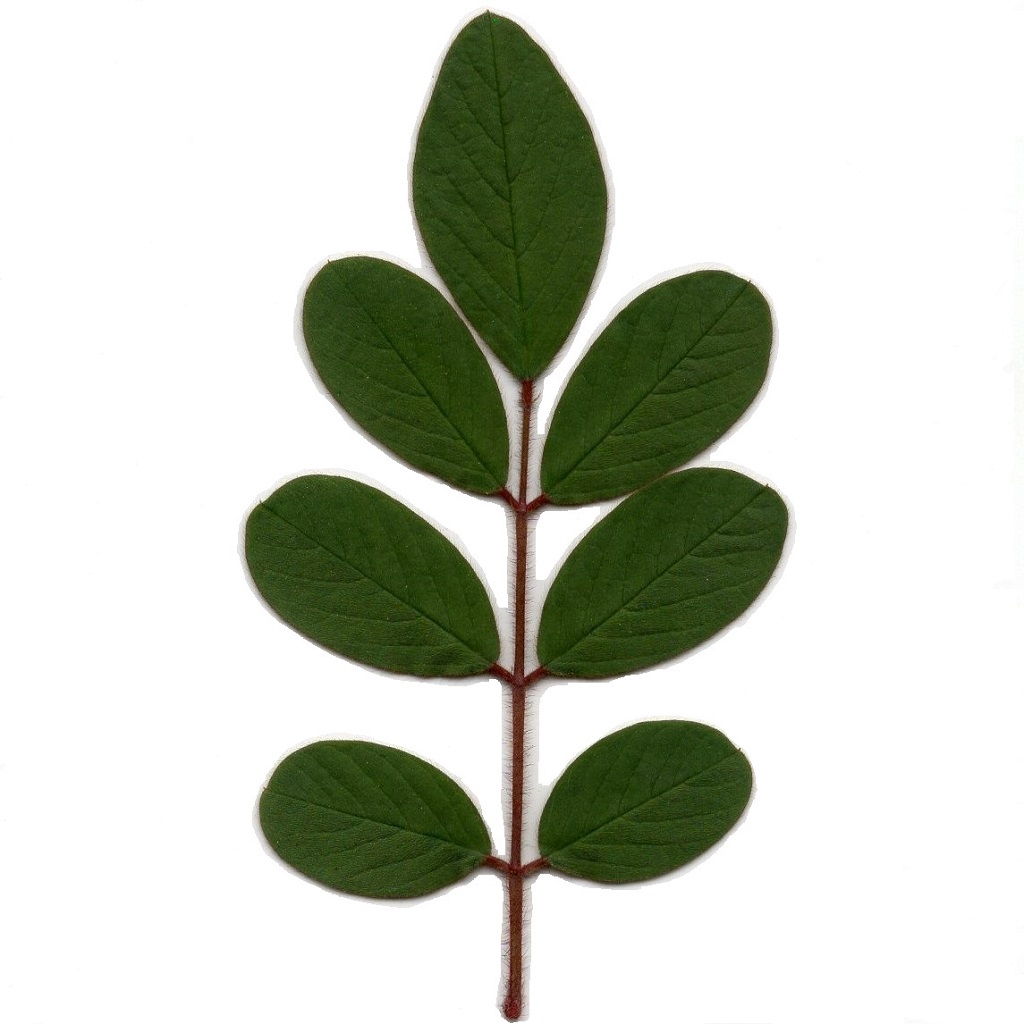

Supplement: S1 Data — (ZIP) [file pone.0293596.s001.zip › S1_data/Indigofera hirsuta.jpg]

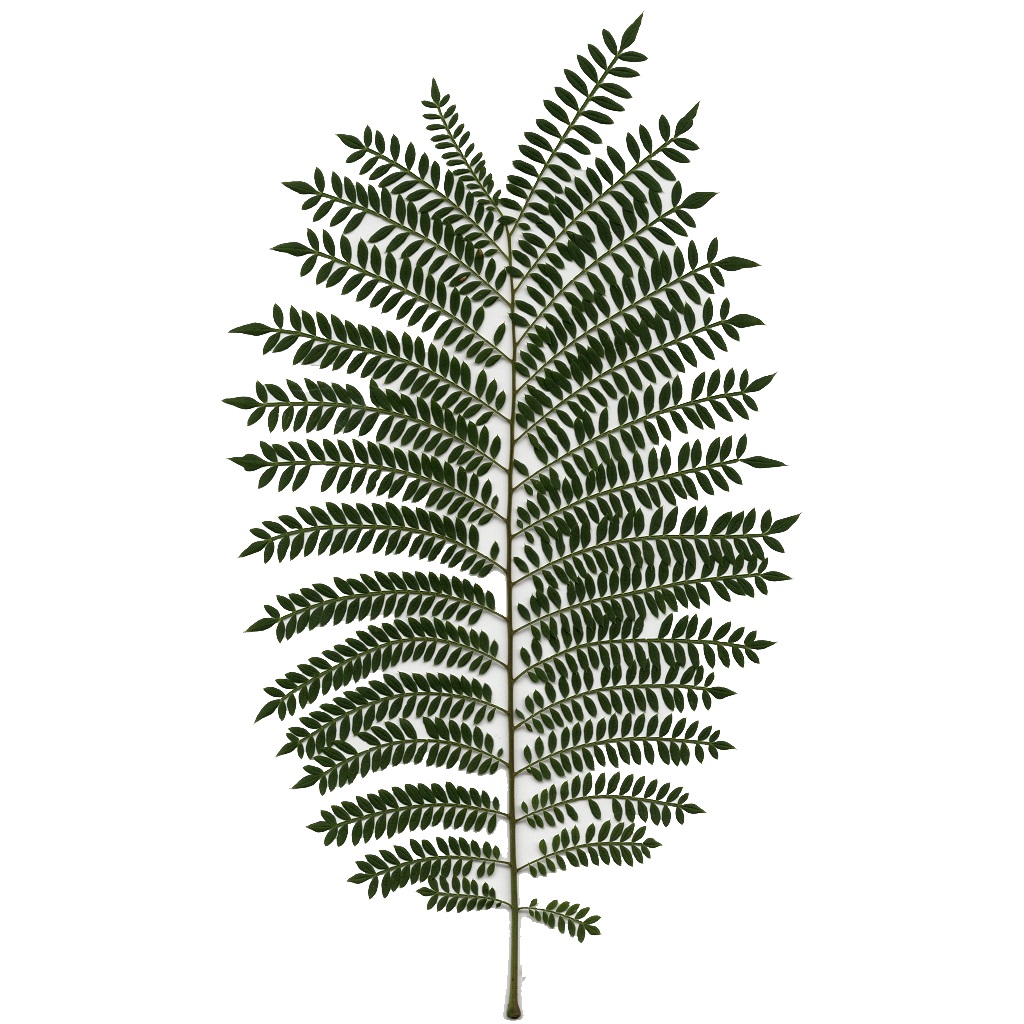

Supplement: S1 Data — (ZIP) [file pone.0293596.s001.zip › S1_data/Jacaranda mimosifolia.jpg]

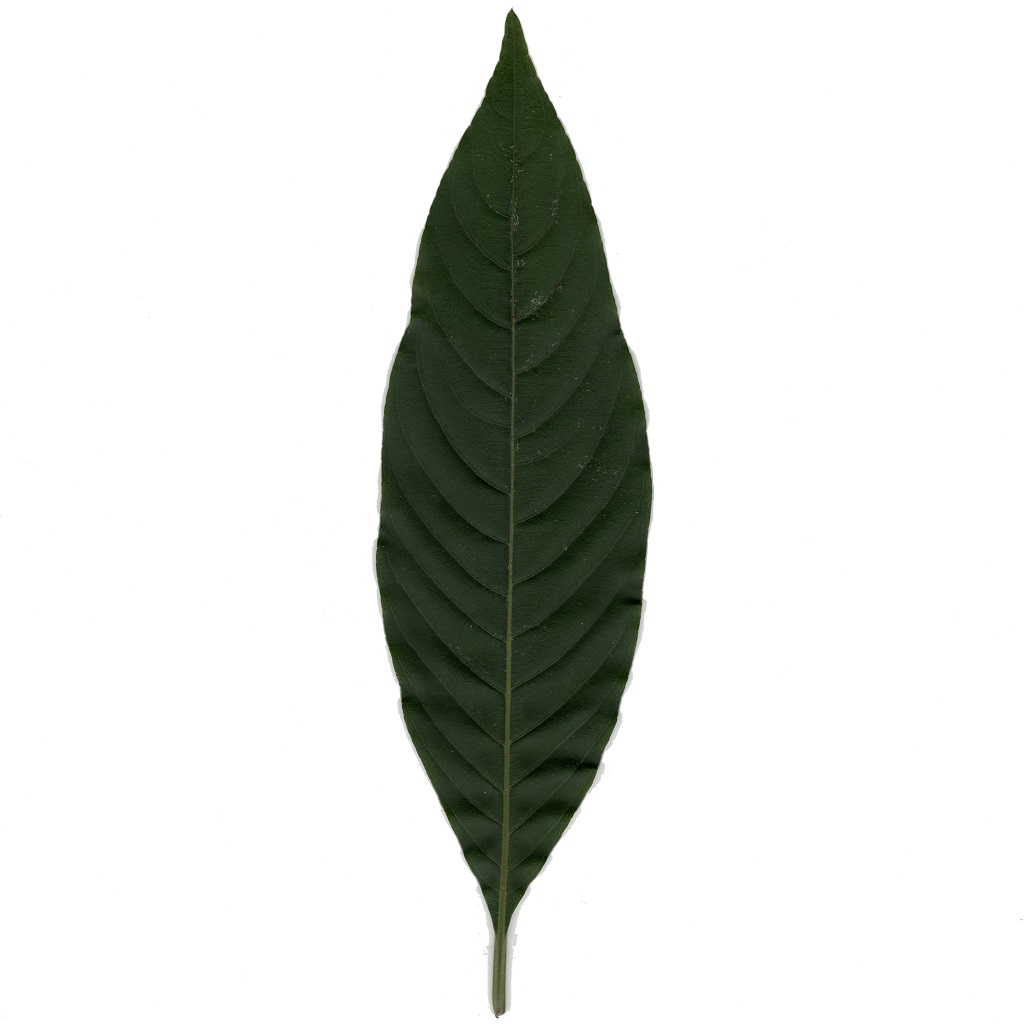

Supplement: S1 Data — (ZIP) [file pone.0293596.s001.zip › S1_data/Justicia adhatoda.jpg]

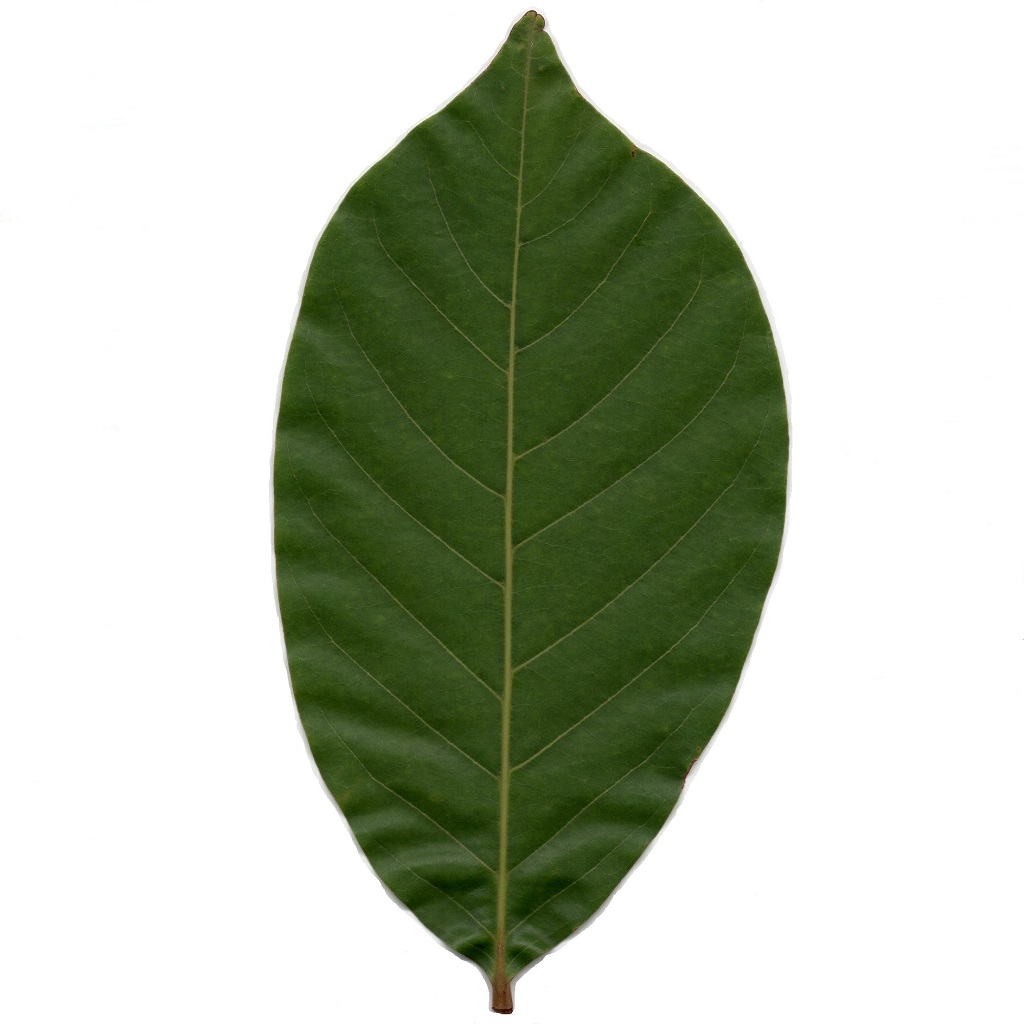

Supplement: S1 Data — (ZIP) [file pone.0293596.s001.zip › S1_data/Lagerstroemia speciosa.jpg]

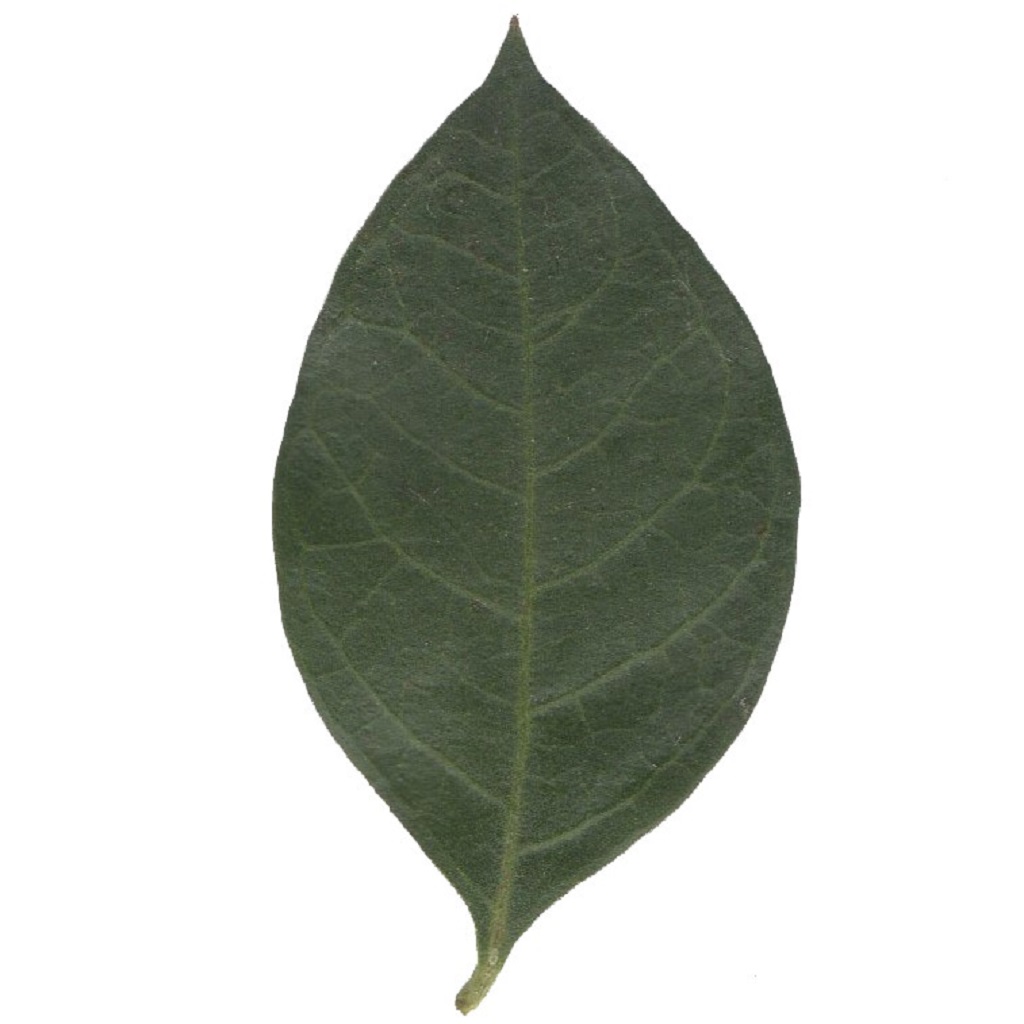

Supplement: S1 Data — (ZIP) [file pone.0293596.s001.zip › S1_data/Lawsonia inermis.jpg]

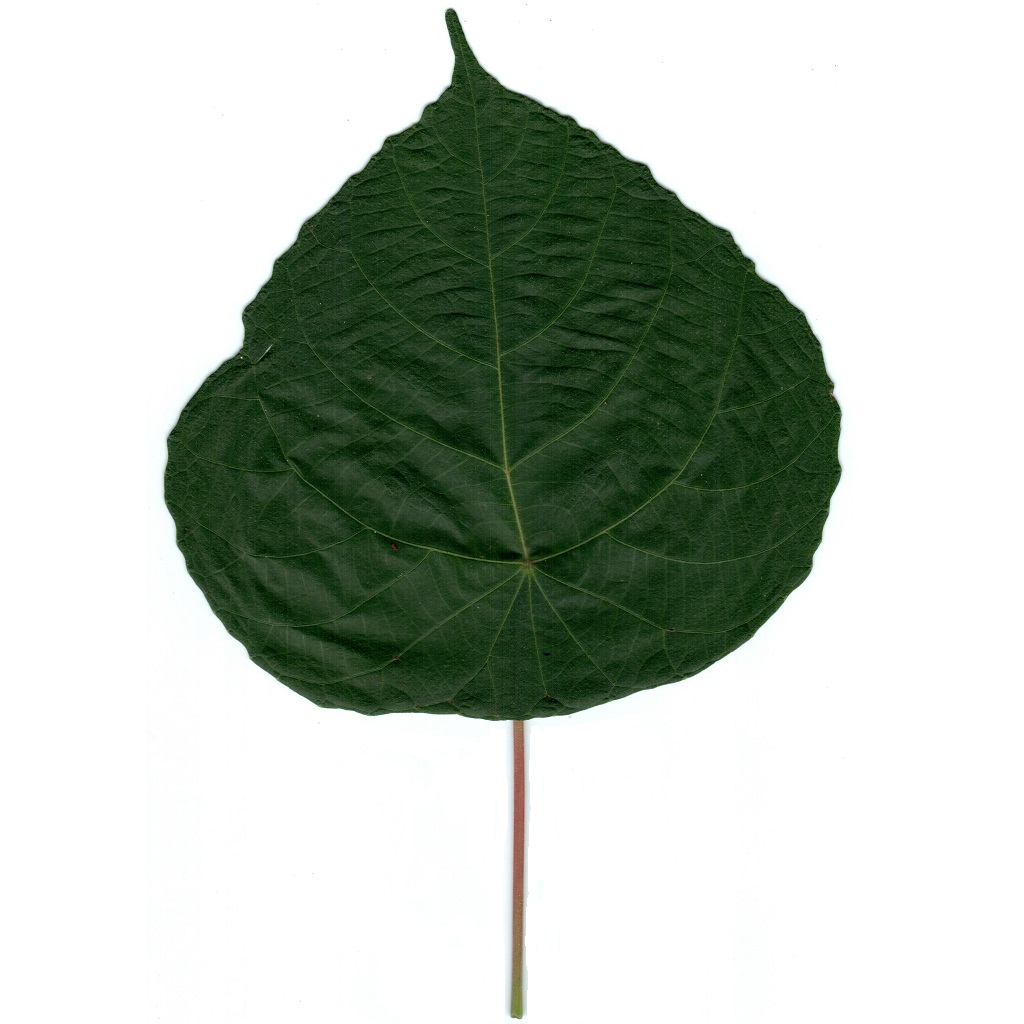

Supplement: S1 Data — (ZIP) [file pone.0293596.s001.zip › S1_data/Macaranga peltata.jpg]

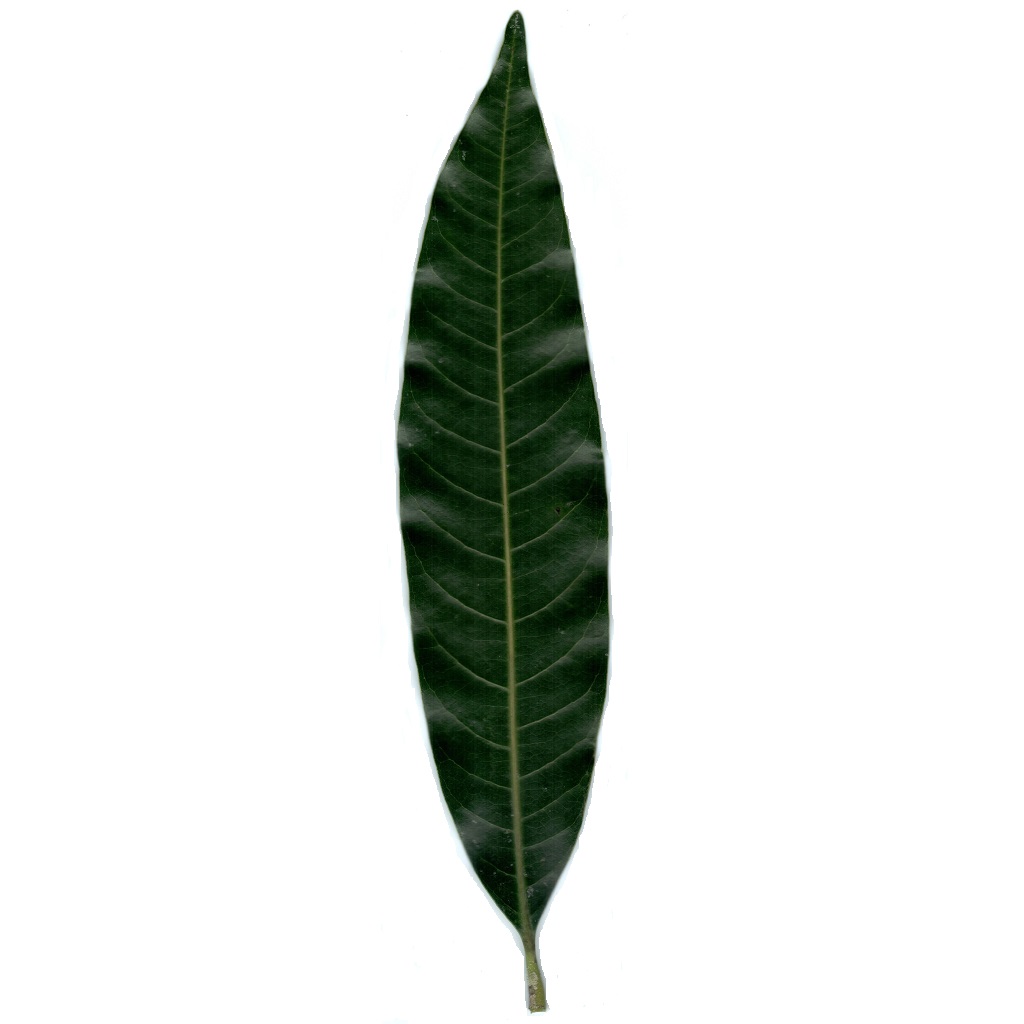

Supplement: S1 Data — (ZIP) [file pone.0293596.s001.zip › S1_data/Mangifera indica.jpg]

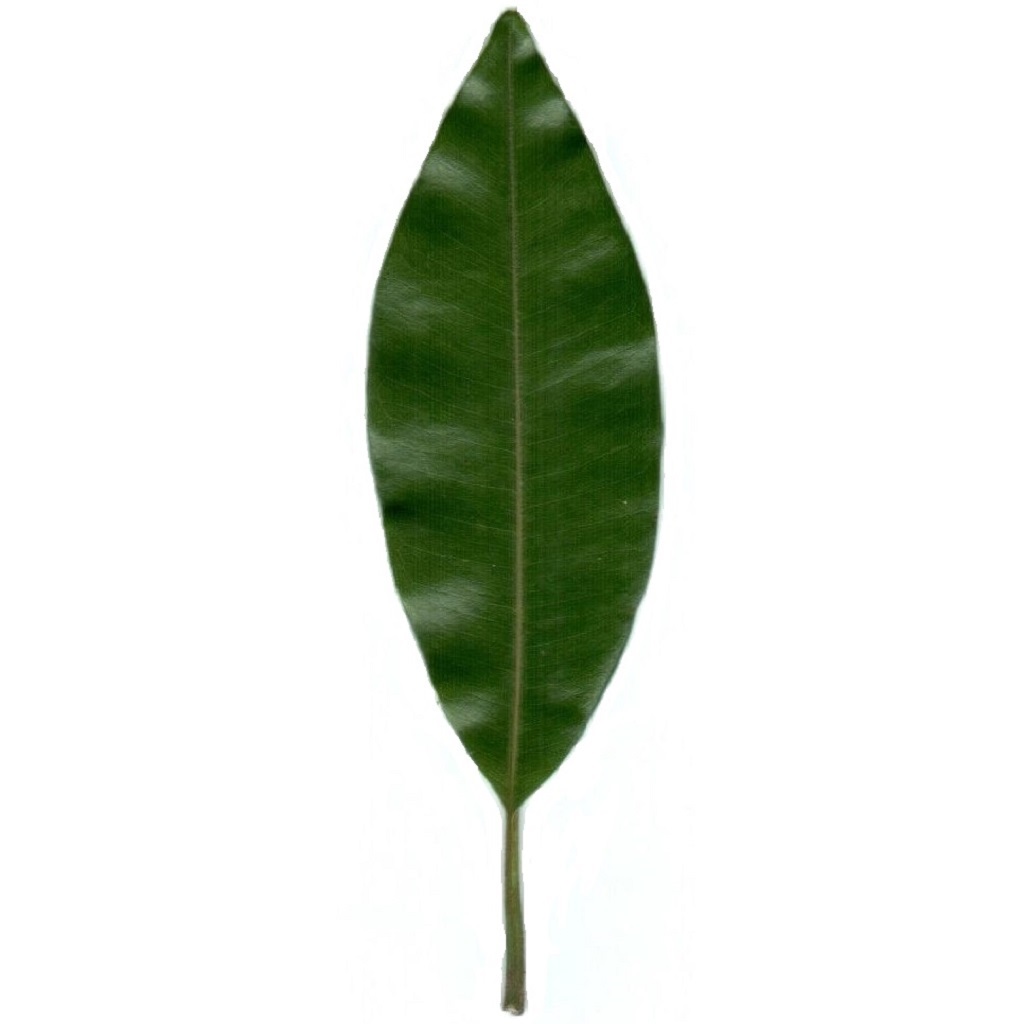

Supplement: S1 Data — (ZIP) [file pone.0293596.s001.zip › S1_data/Manilkara zapota.jpg]

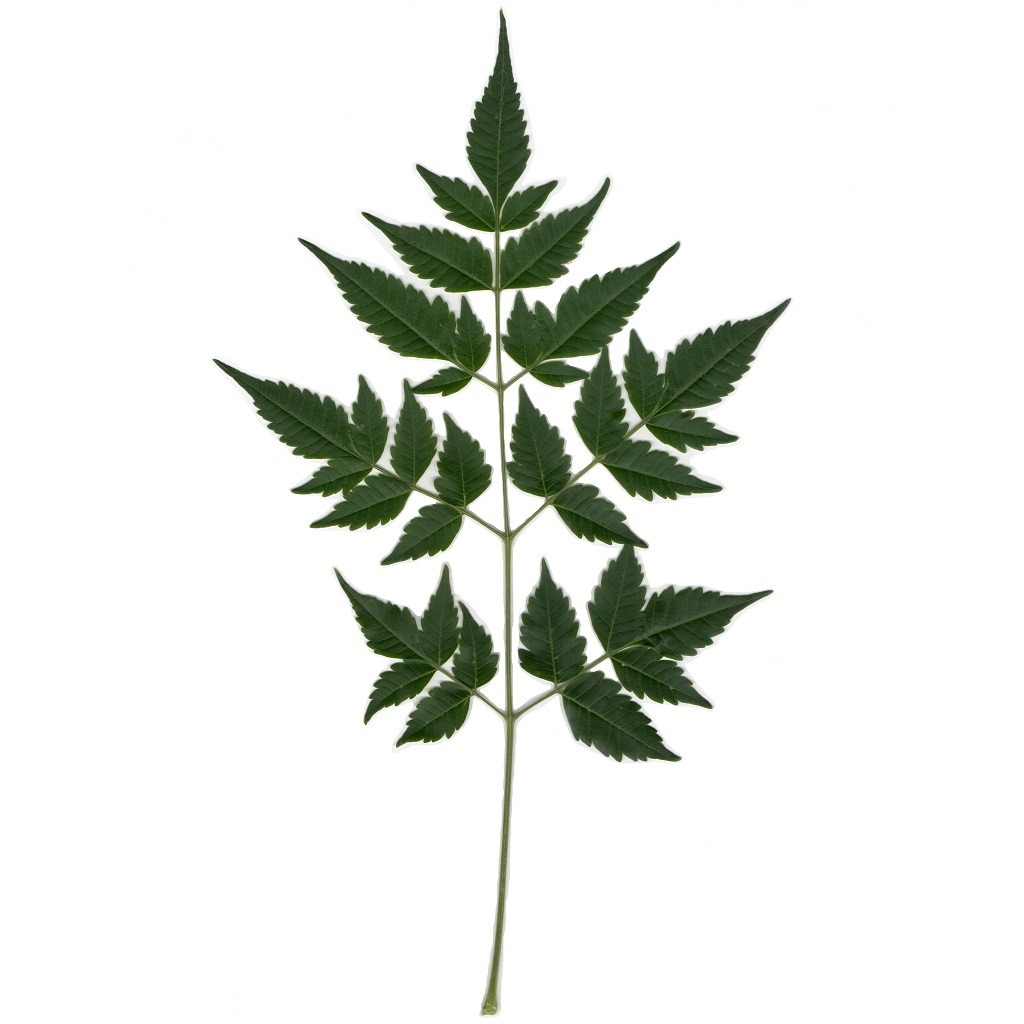

Supplement: S1 Data — (ZIP) [file pone.0293596.s001.zip › S1_data/Melia azedarach.jpg]

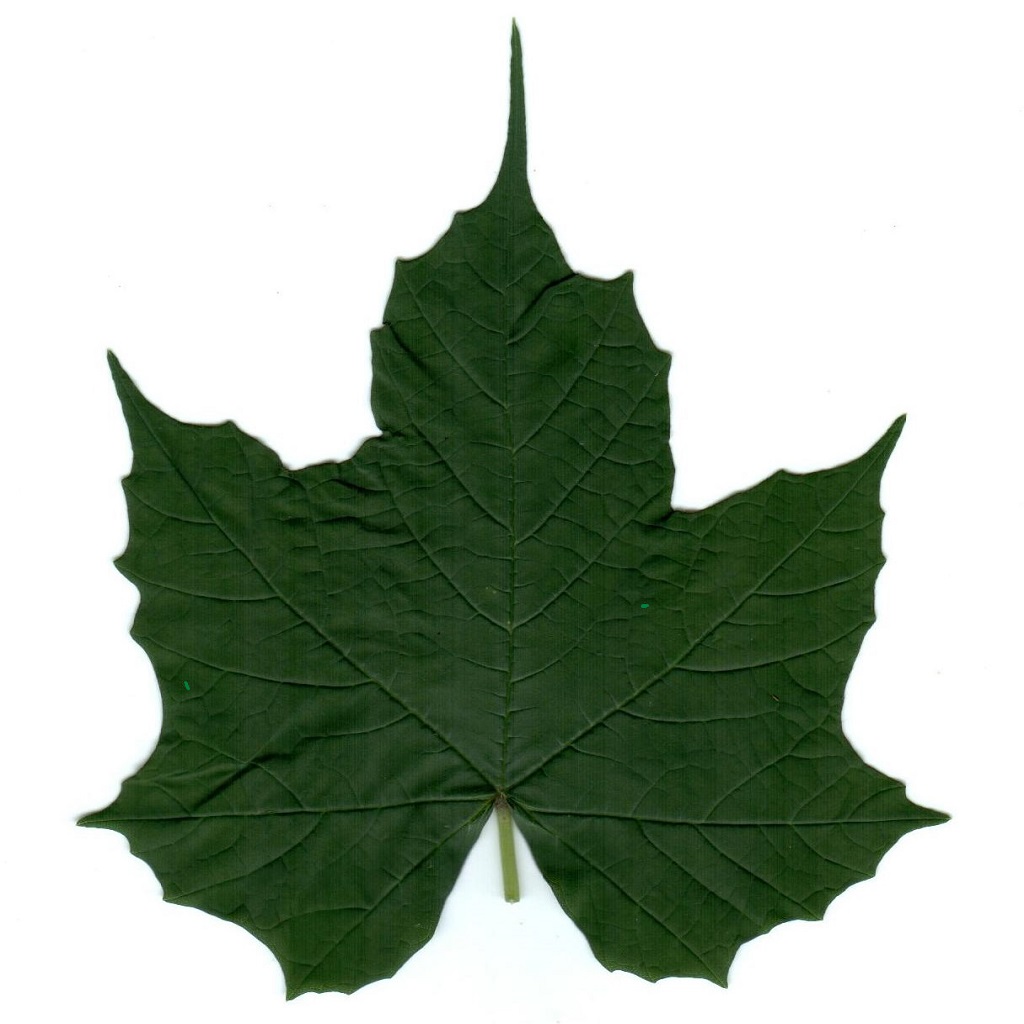

Supplement: S1 Data — (ZIP) [file pone.0293596.s001.zip › S1_data/Merremia vitifolia.jpg]

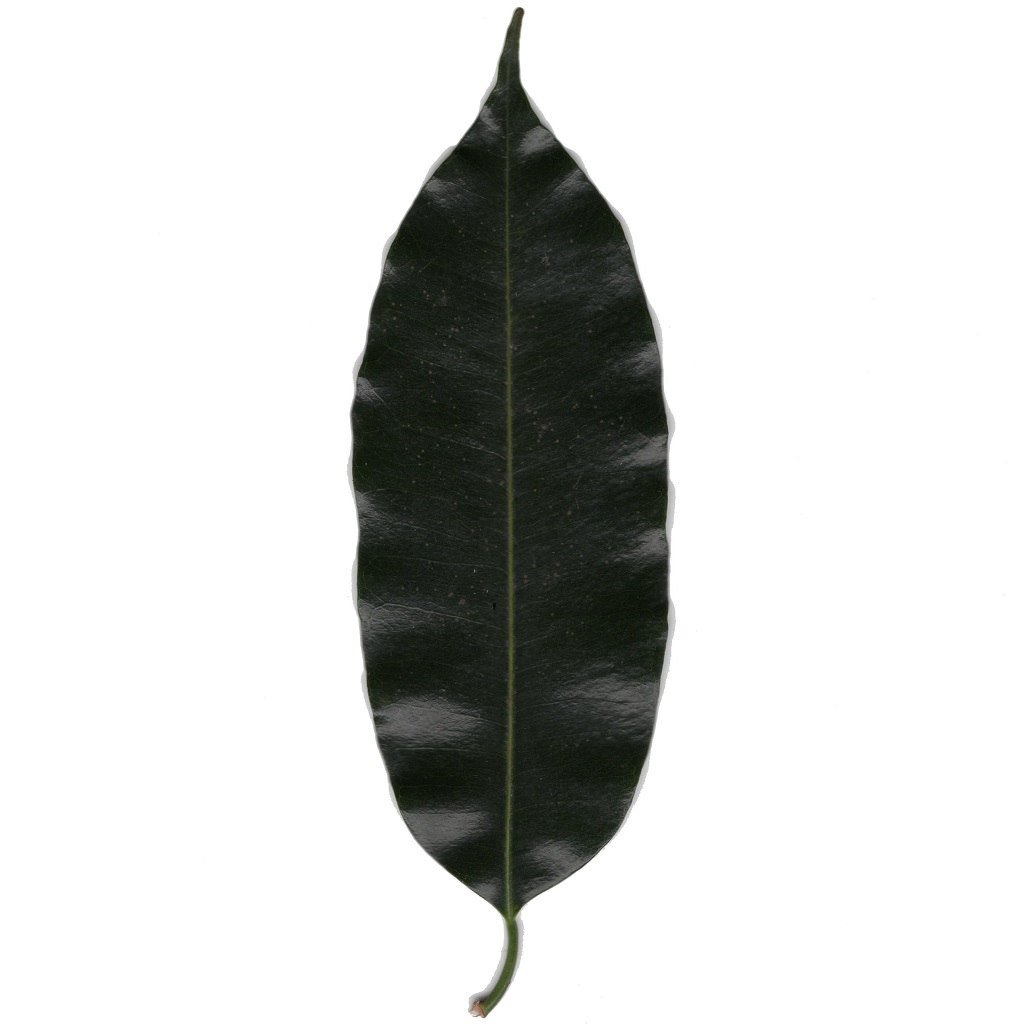

Supplement: S1 Data — (ZIP) [file pone.0293596.s001.zip › S1_data/Mimusops elengi.jpg]

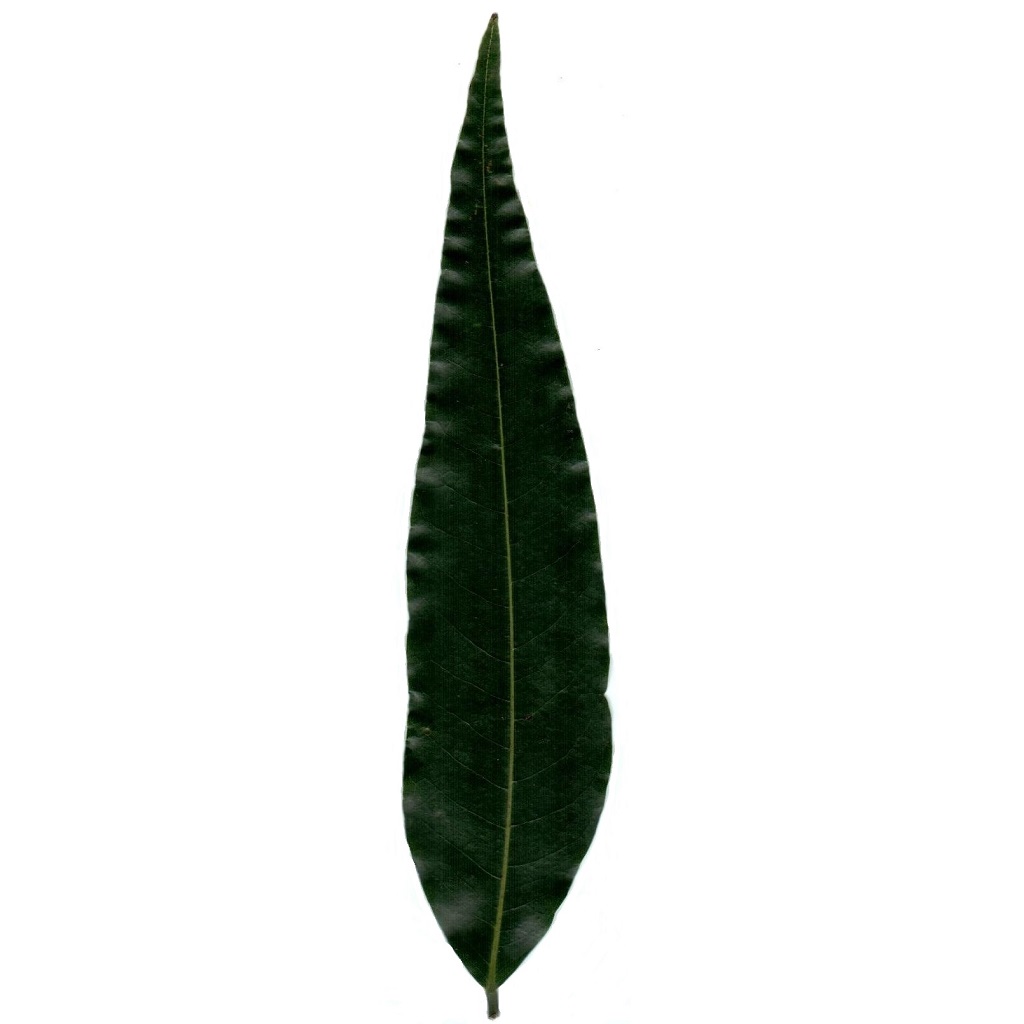

Supplement: S1 Data — (ZIP) [file pone.0293596.s001.zip › S1_data/Monoon longifolium.jpg]

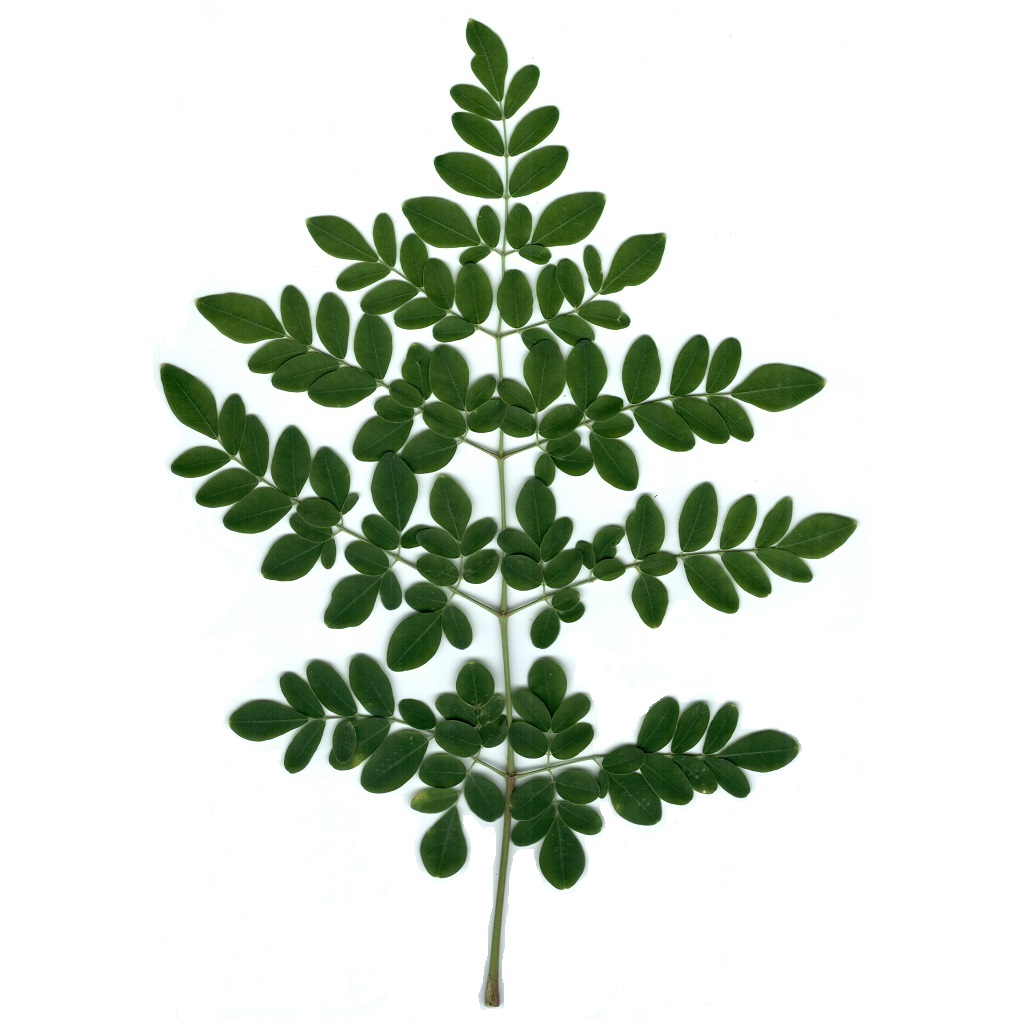

Supplement: S1 Data — (ZIP) [file pone.0293596.s001.zip › S1_data/Moringa oleifera.jpg]

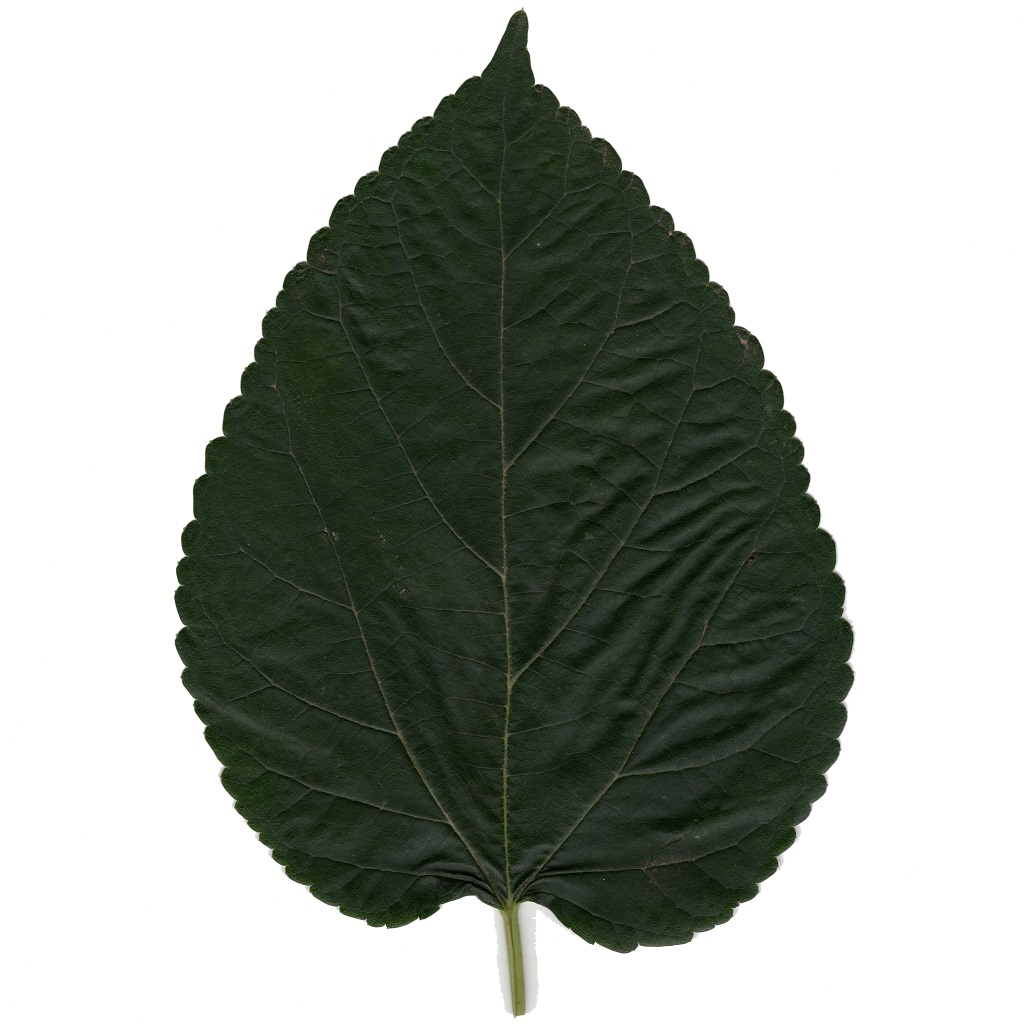

Supplement: S1 Data — (ZIP) [file pone.0293596.s001.zip › S1_data/Morus alba.jpg]

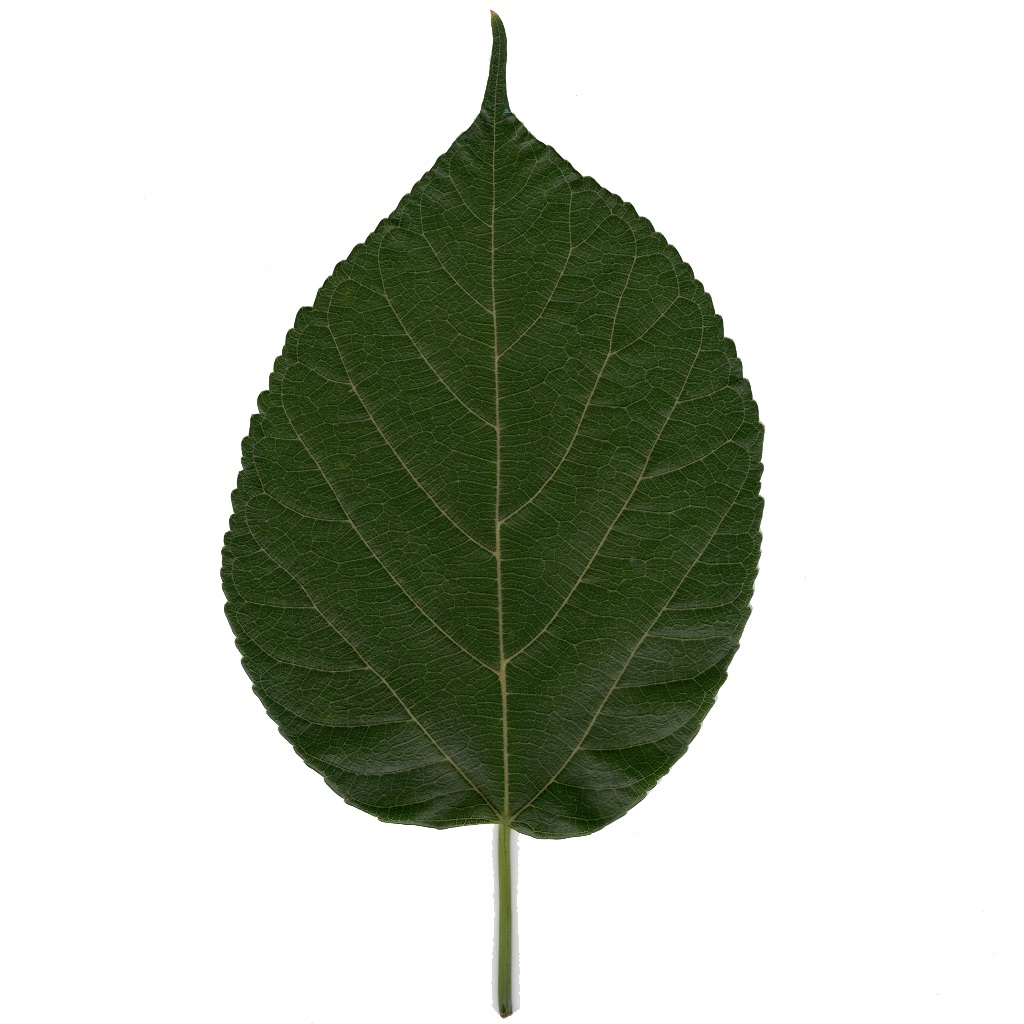

Supplement: S1 Data — (ZIP) [file pone.0293596.s001.zip › S1_data/Morus macroura.jpg]

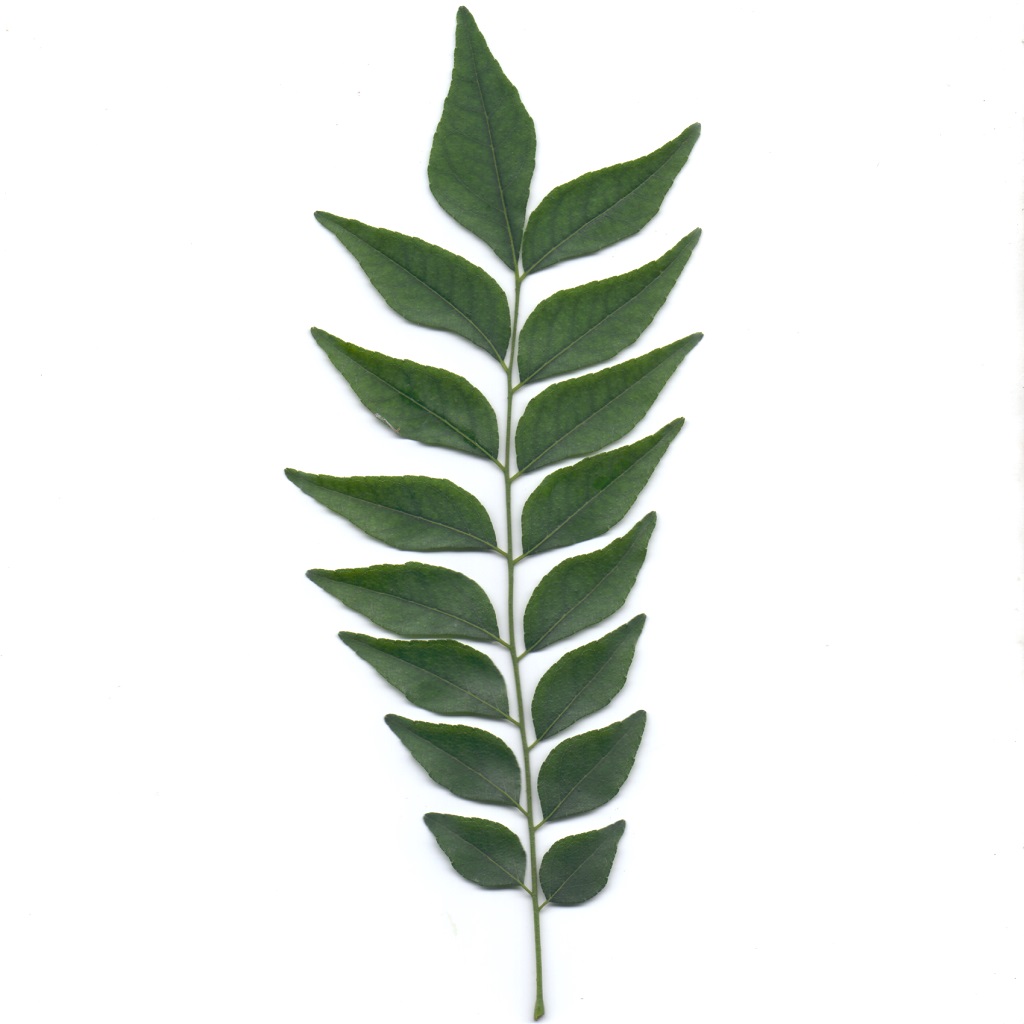

Supplement: S1 Data — (ZIP) [file pone.0293596.s001.zip › S1_data/Murraya koenigii.jpg]

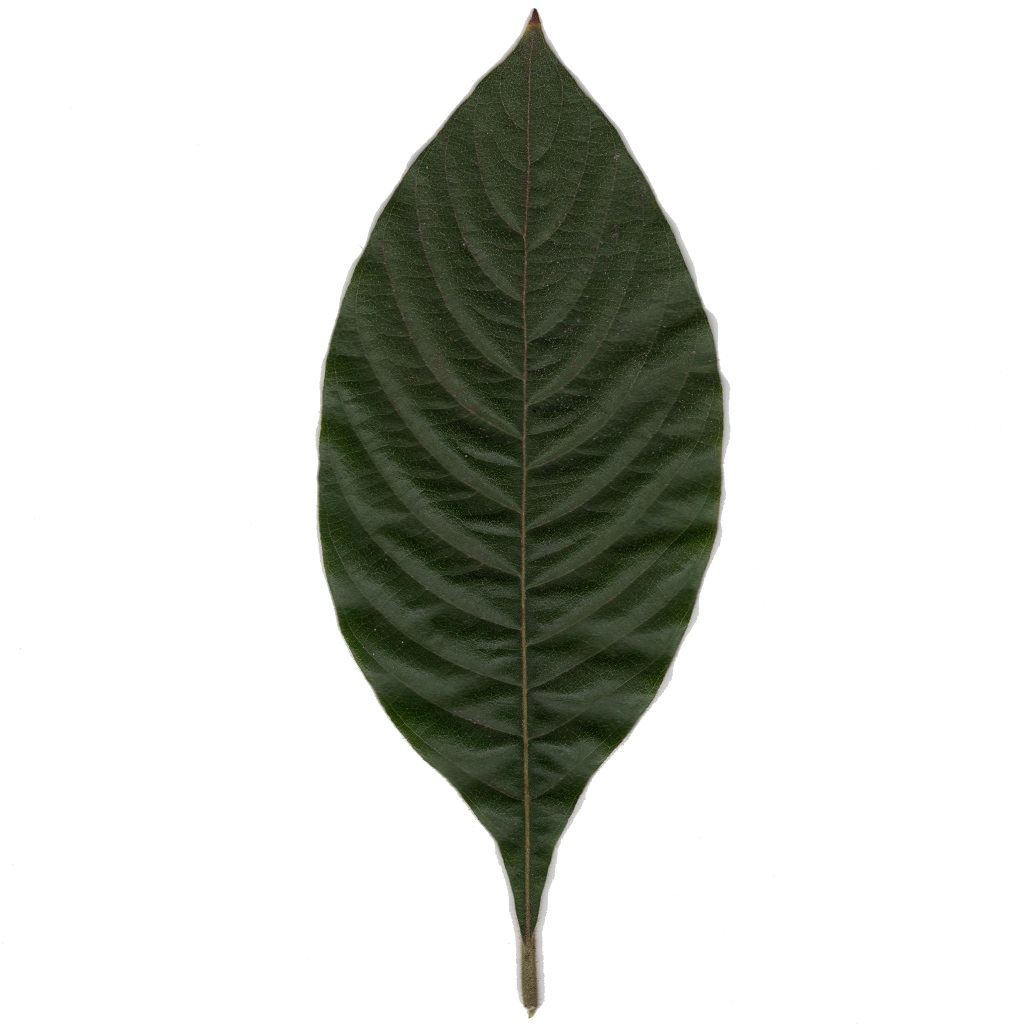

Supplement: S1 Data — (ZIP) [file pone.0293596.s001.zip › S1_data/Mussaenda philippica.jpg]

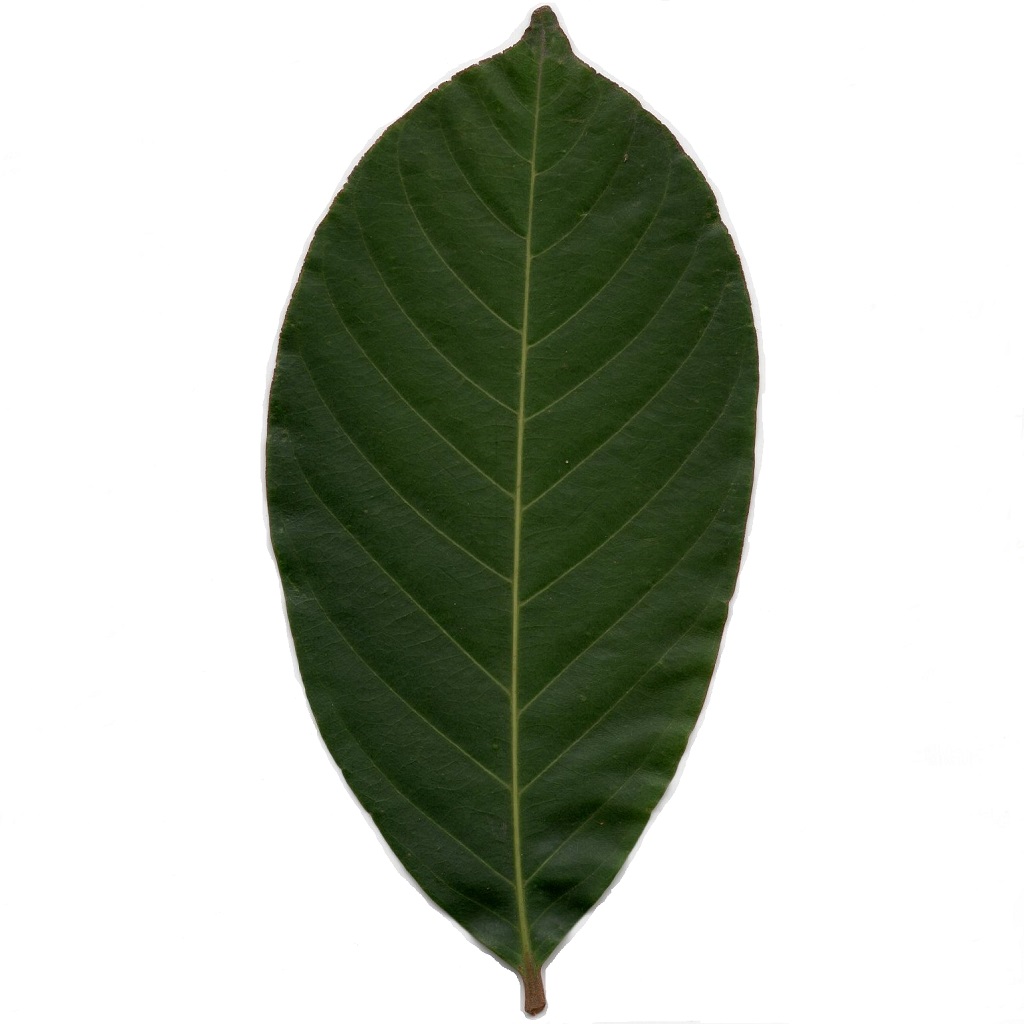

Supplement: S1 Data — (ZIP) [file pone.0293596.s001.zip › S1_data/Nephelium lappaceum.jpg]

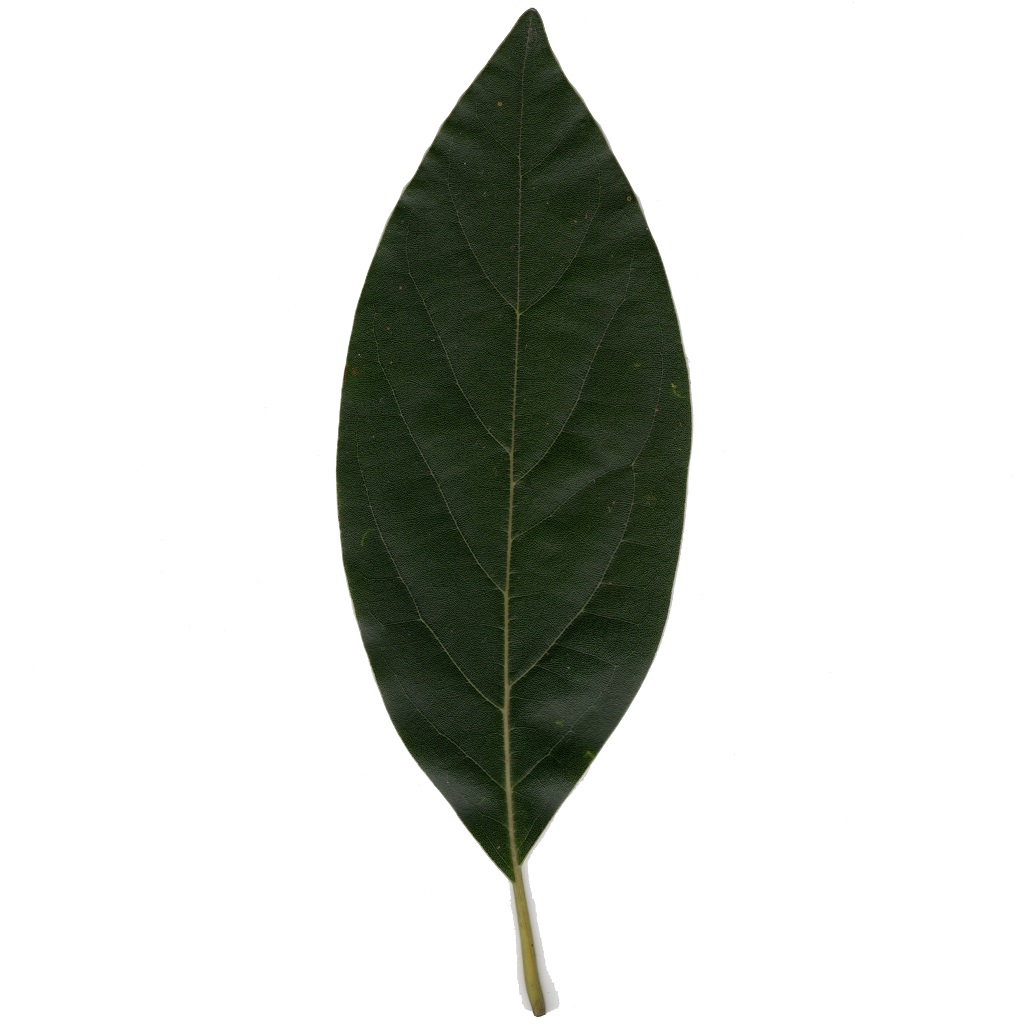

Supplement: S1 Data — (ZIP) [file pone.0293596.s001.zip › S1_data/Nephelium mutabile.jpg]

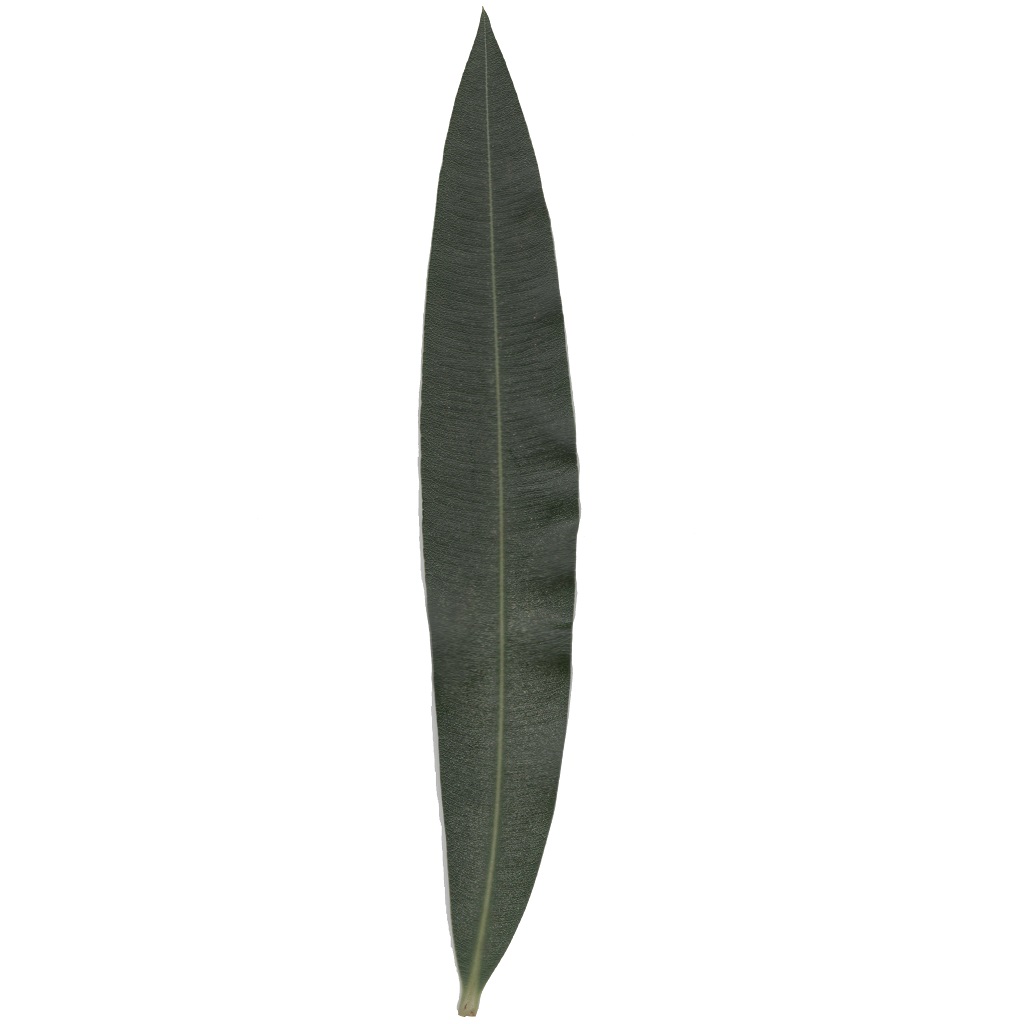

Supplement: S1 Data — (ZIP) [file pone.0293596.s001.zip › S1_data/Nerium oleander.jpg]

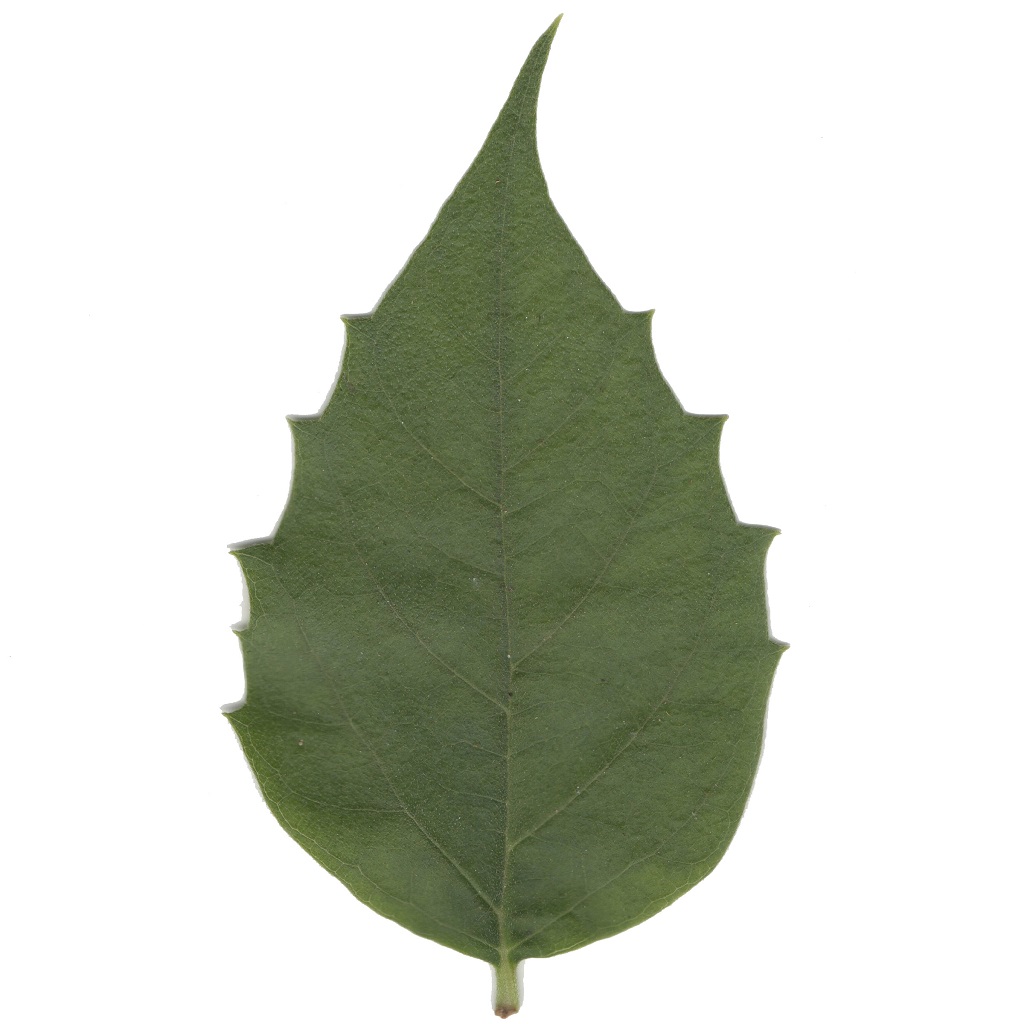

Supplement: S1 Data — (ZIP) [file pone.0293596.s001.zip › S1_data/Nyctanthes arbor-tristis.jpg]

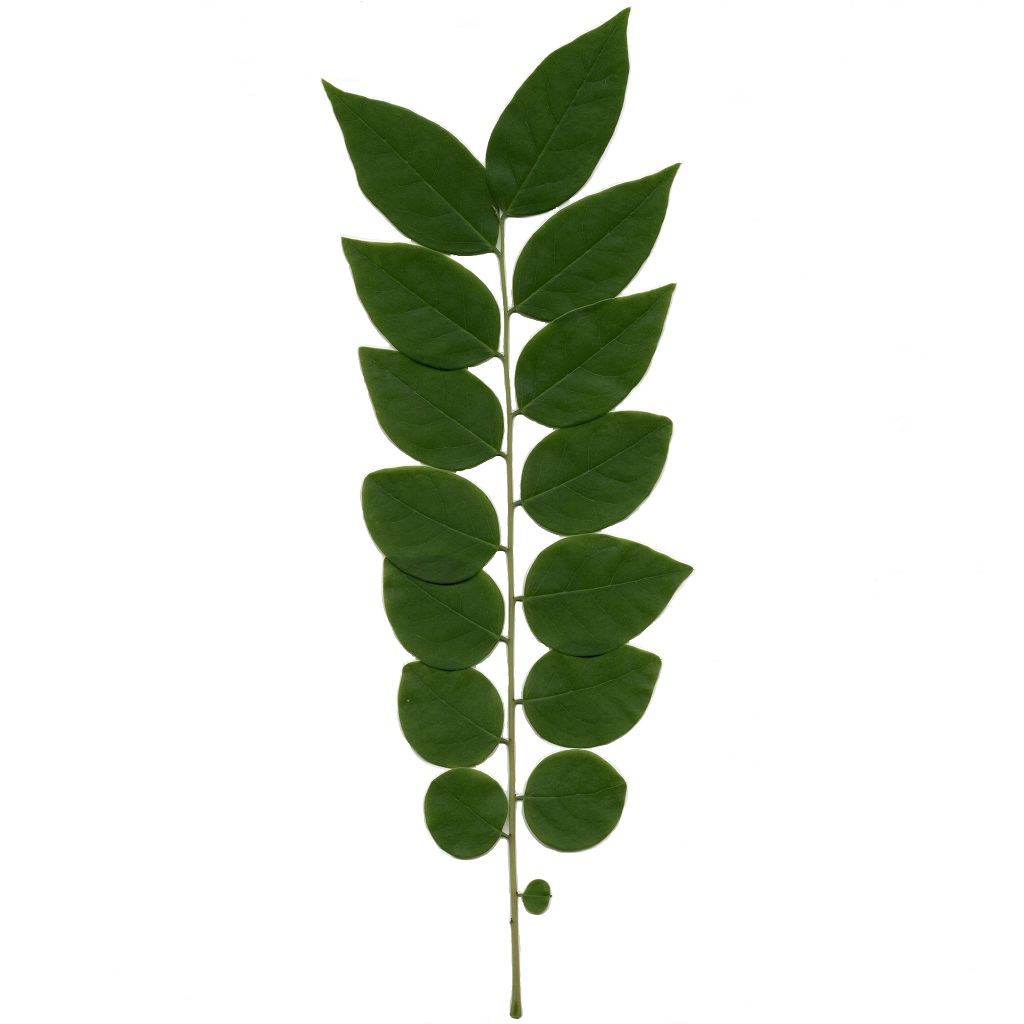

Supplement: S1 Data — (ZIP) [file pone.0293596.s001.zip › S1_data/phyllanthus acidus.jpg]

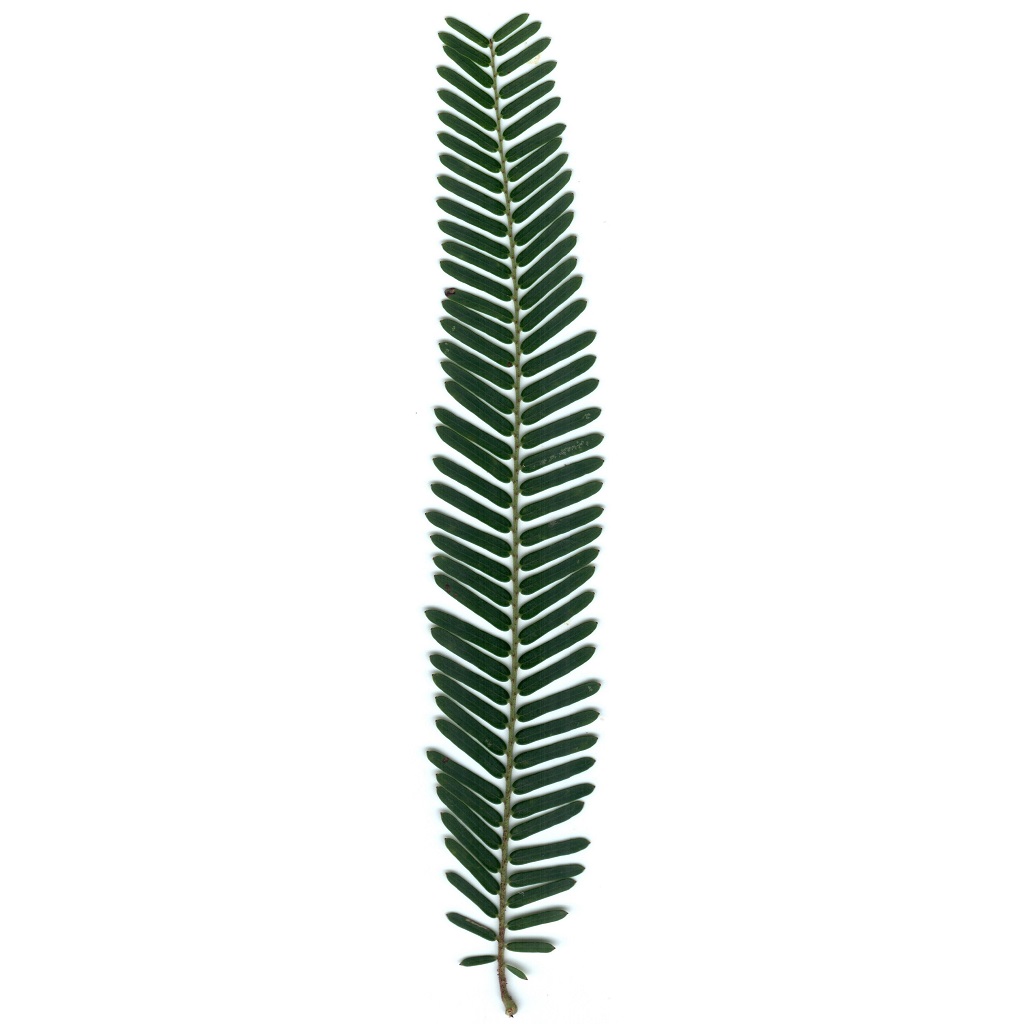

Supplement: S1 Data — (ZIP) [file pone.0293596.s001.zip › S1_data/Phyllanthus emblica.jpg]

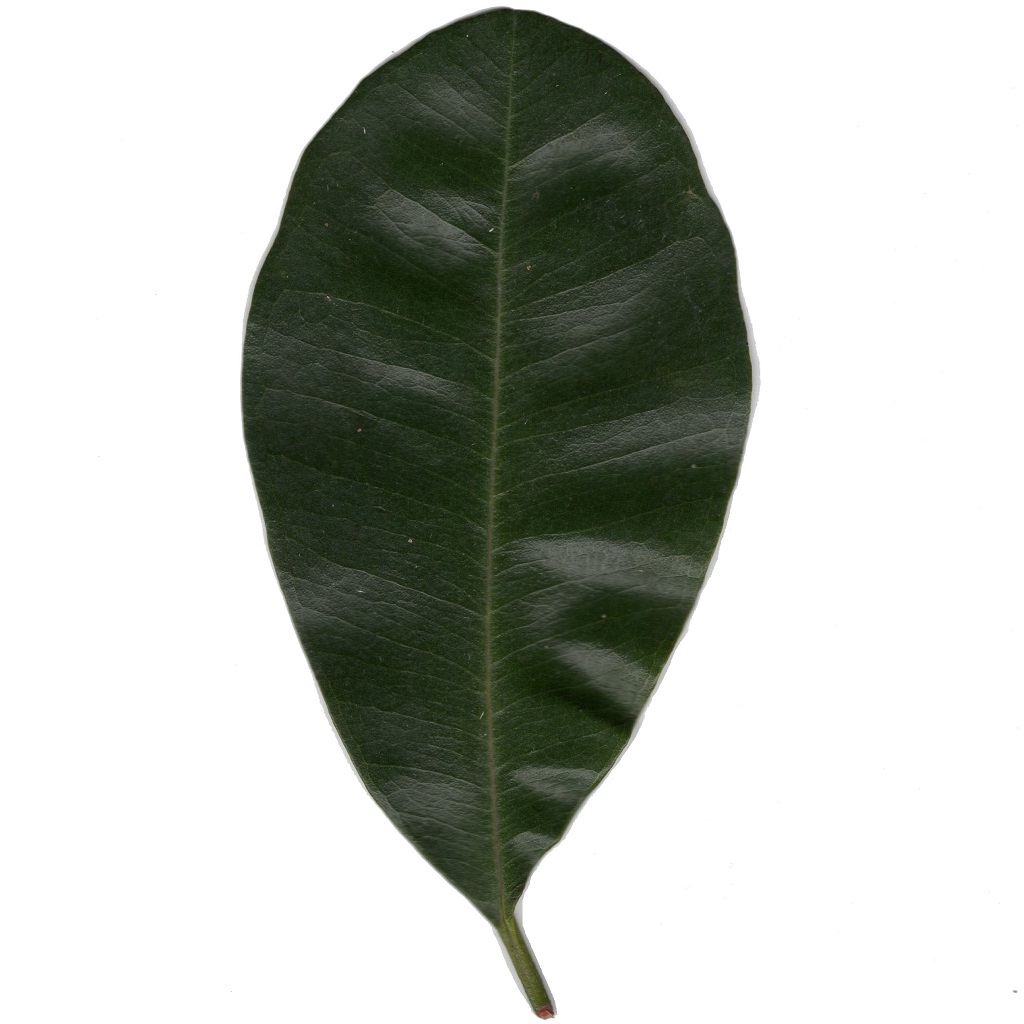

Supplement: S1 Data — (ZIP) [file pone.0293596.s001.zip › S1_data/Pimenta dioica.jpg]

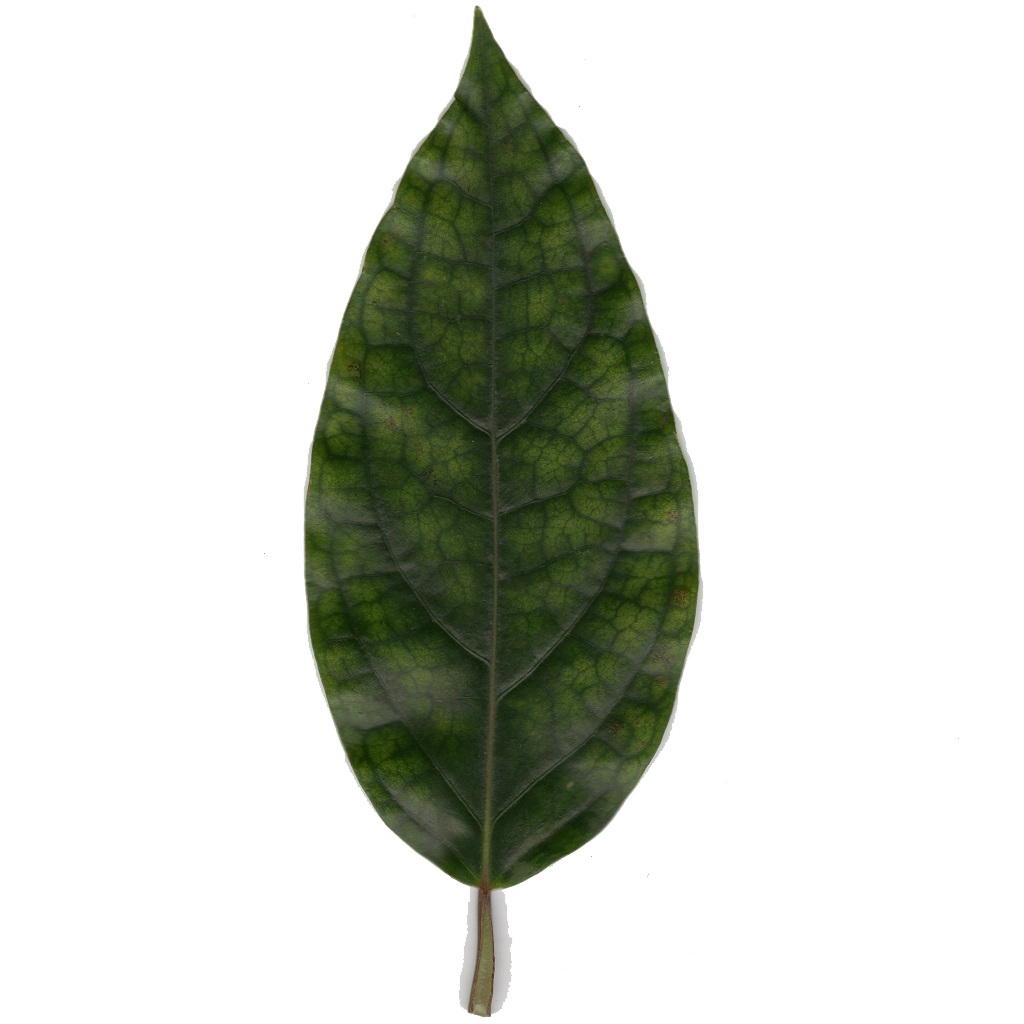

Supplement: S1 Data — (ZIP) [file pone.0293596.s001.zip › S1_data/Piper longum.jpg]

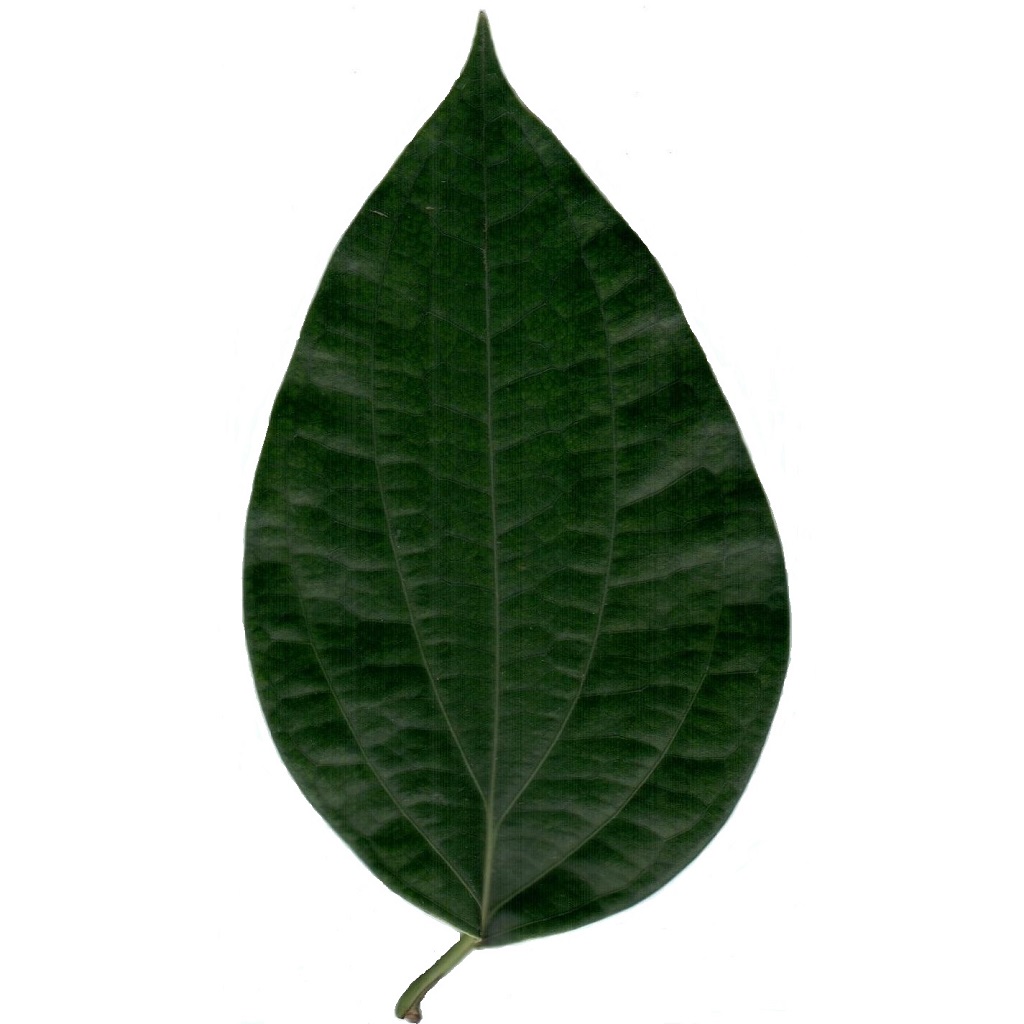

Supplement: S1 Data — (ZIP) [file pone.0293596.s001.zip › S1_data/Piper nigrum.jpg]

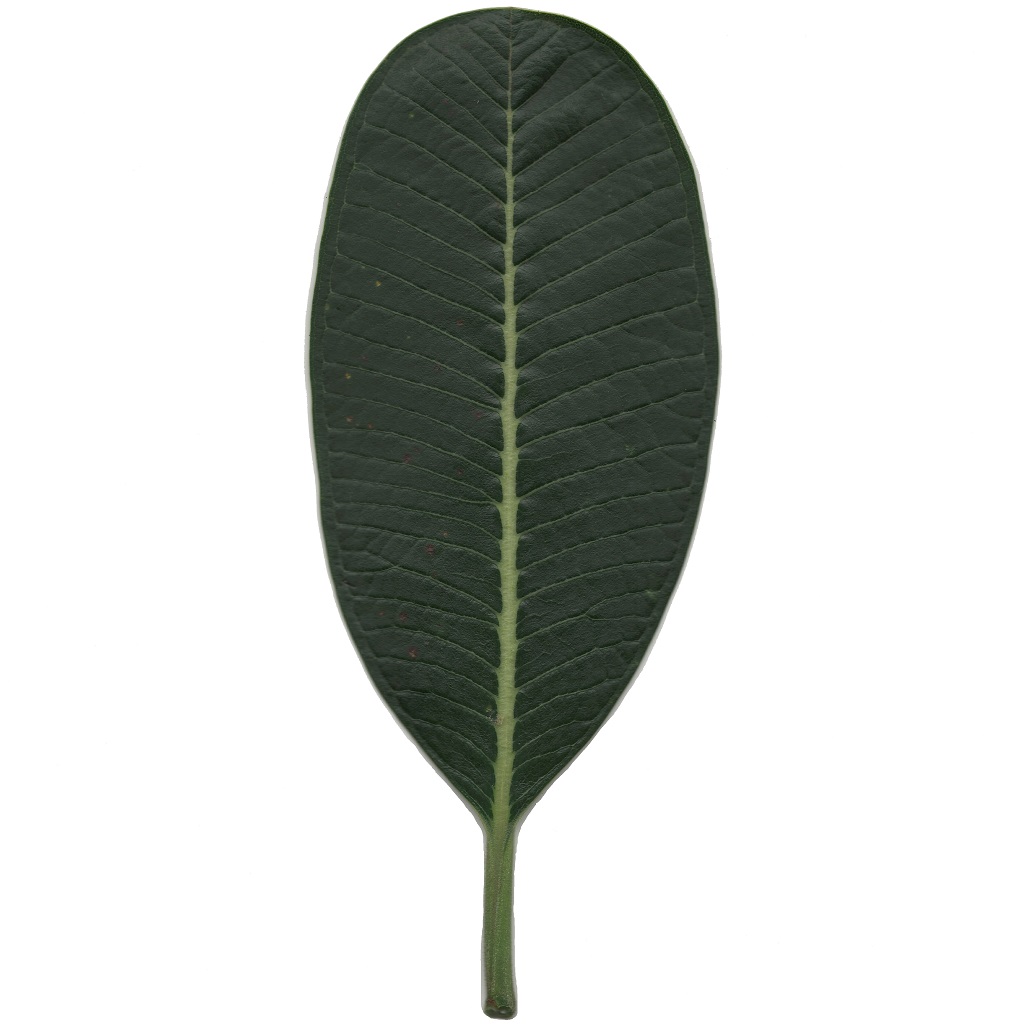

Supplement: S1 Data — (ZIP) [file pone.0293596.s001.zip › S1_data/Plumeria alba.jpg]

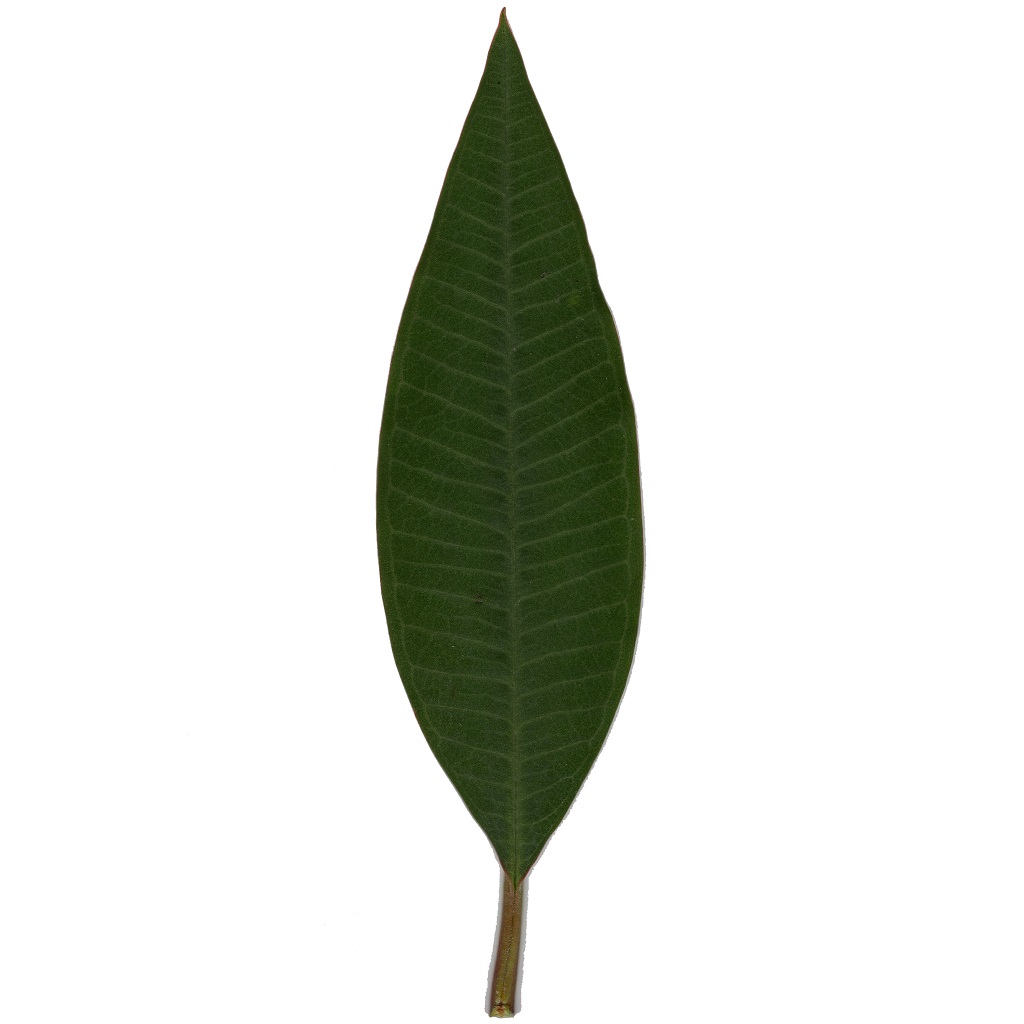

Supplement: S1 Data — (ZIP) [file pone.0293596.s001.zip › S1_data/Plumeria rubra.jpg]

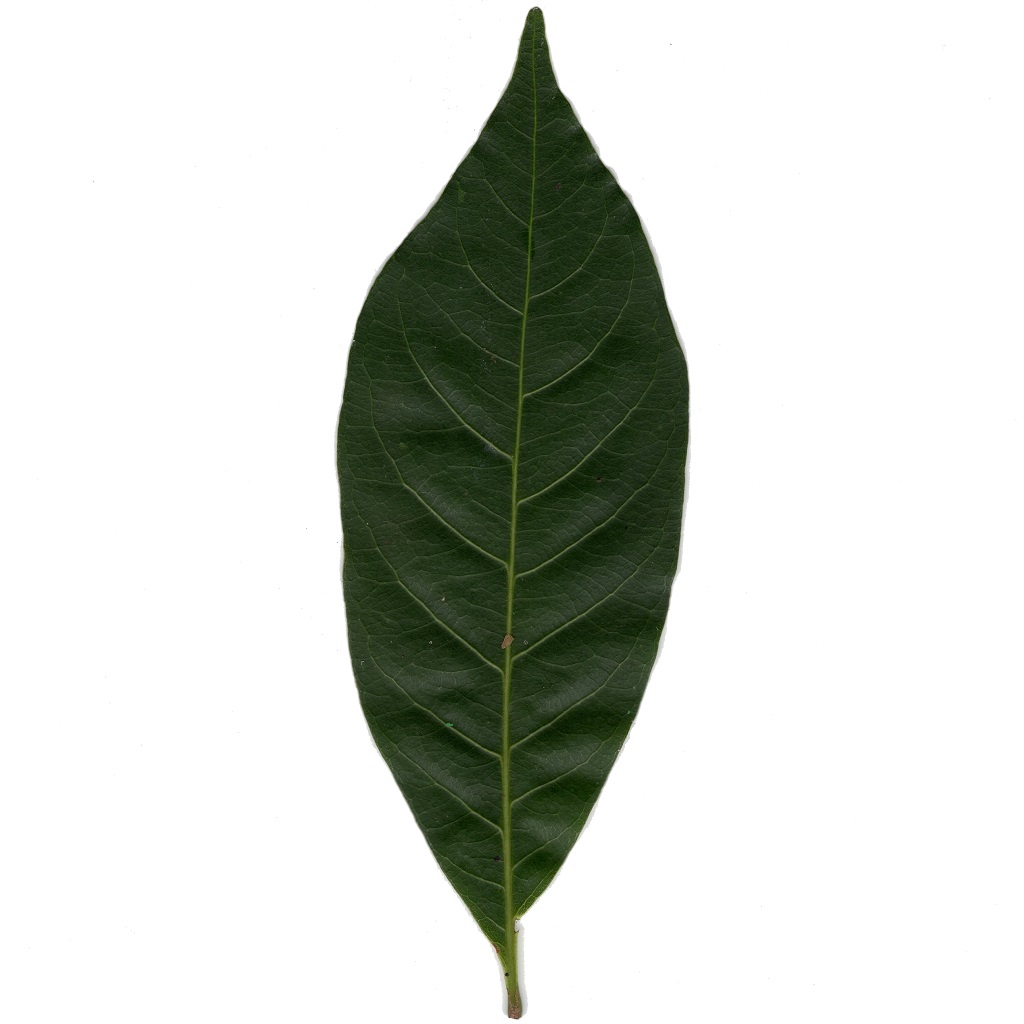

Supplement: S1 Data — (ZIP) [file pone.0293596.s001.zip › S1_data/Pouteria caimito.jpg]

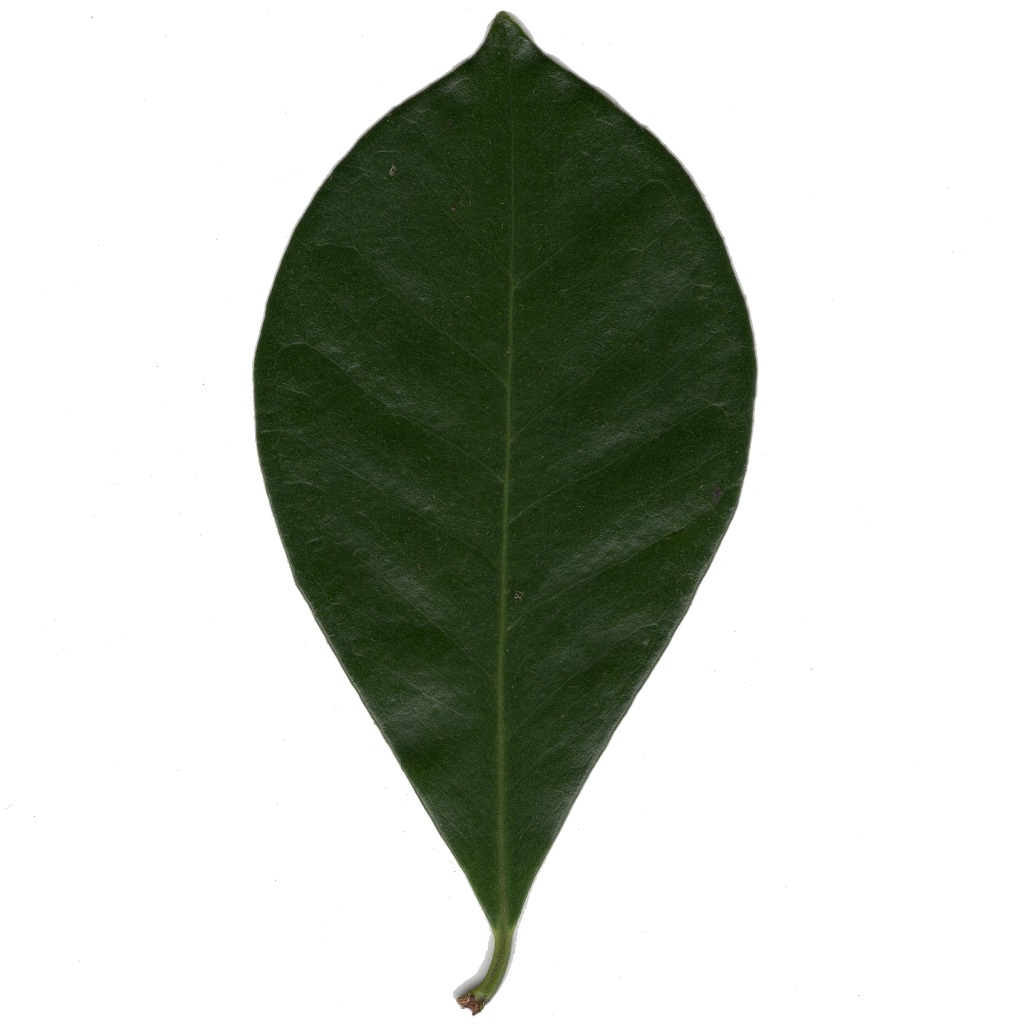

Supplement: S1 Data — (ZIP) [file pone.0293596.s001.zip › S1_data/Psidium cattleianum.jpg]

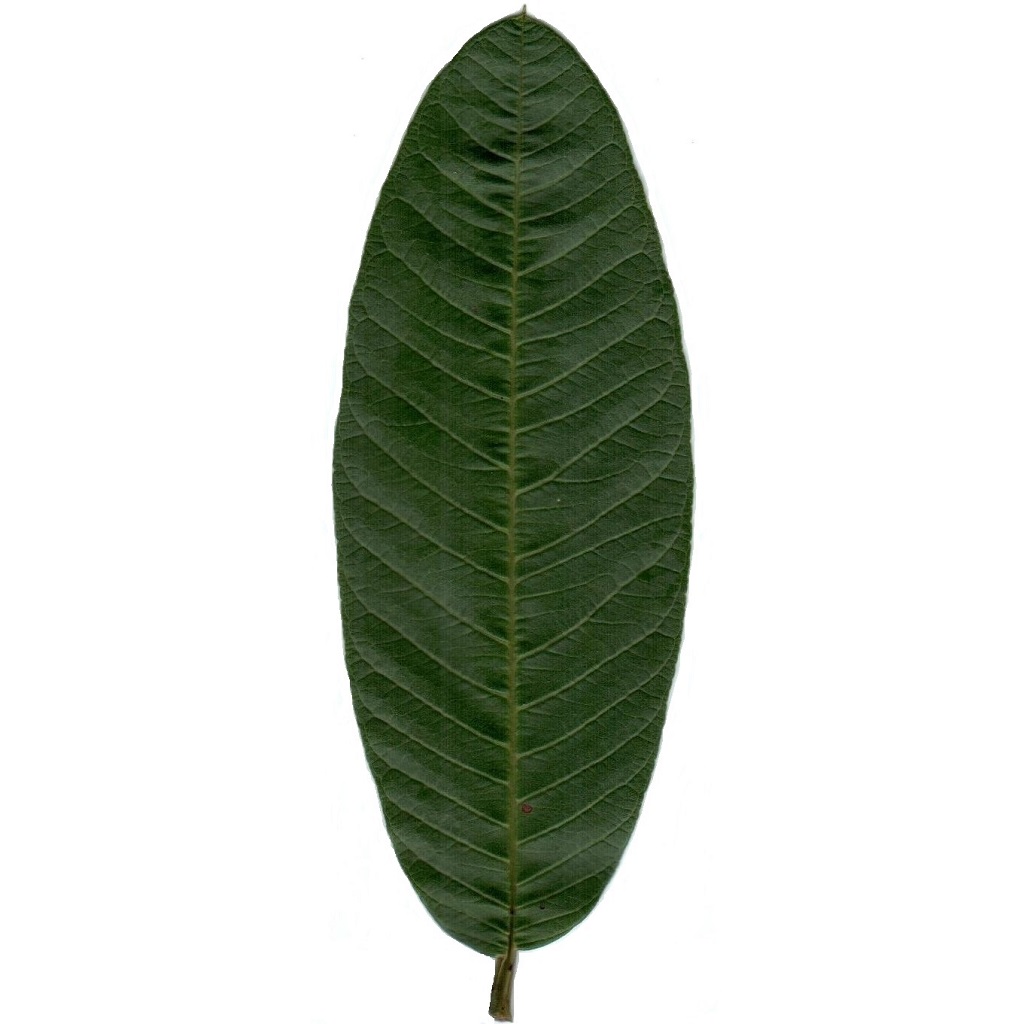

Supplement: S1 Data — (ZIP) [file pone.0293596.s001.zip › S1_data/Psidium guajava.jpg]

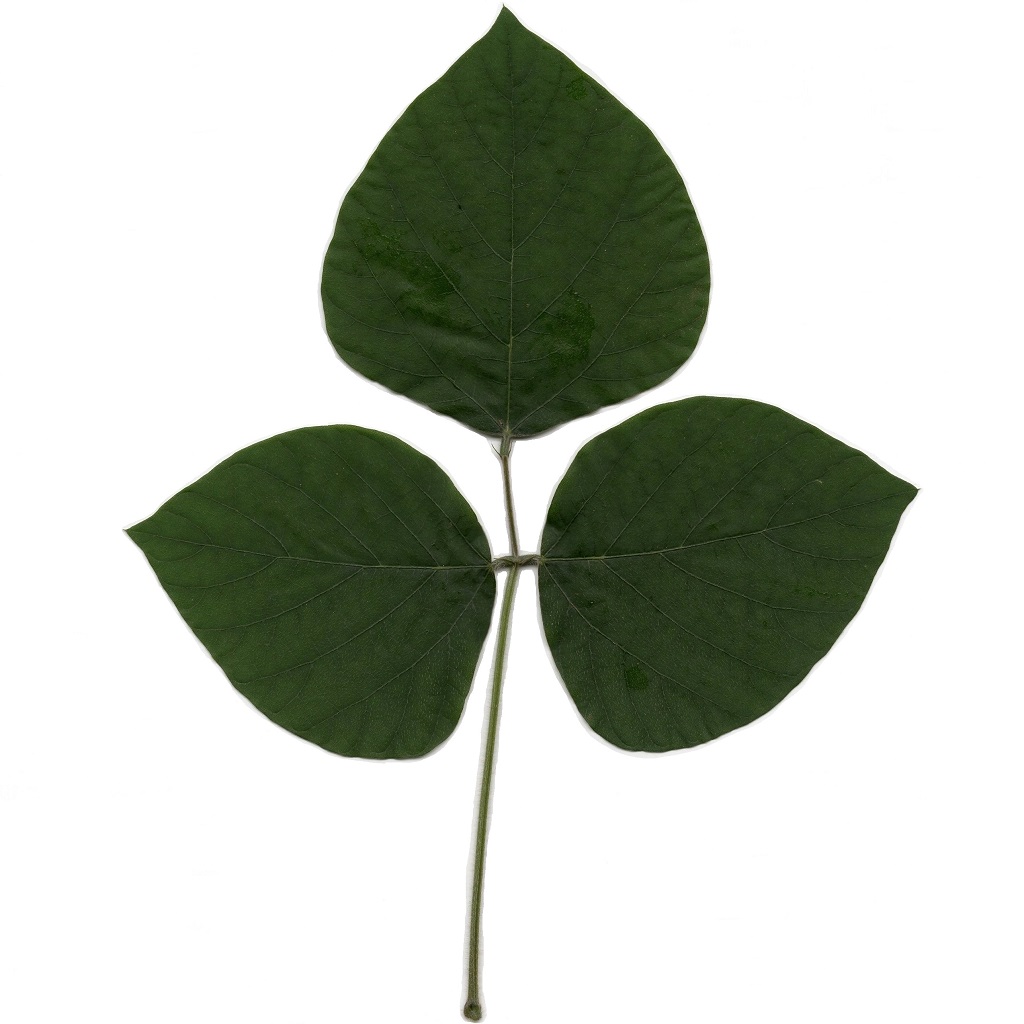

Supplement: S1 Data — (ZIP) [file pone.0293596.s001.zip › S1_data/Pueraria phaseoloides.jpg]

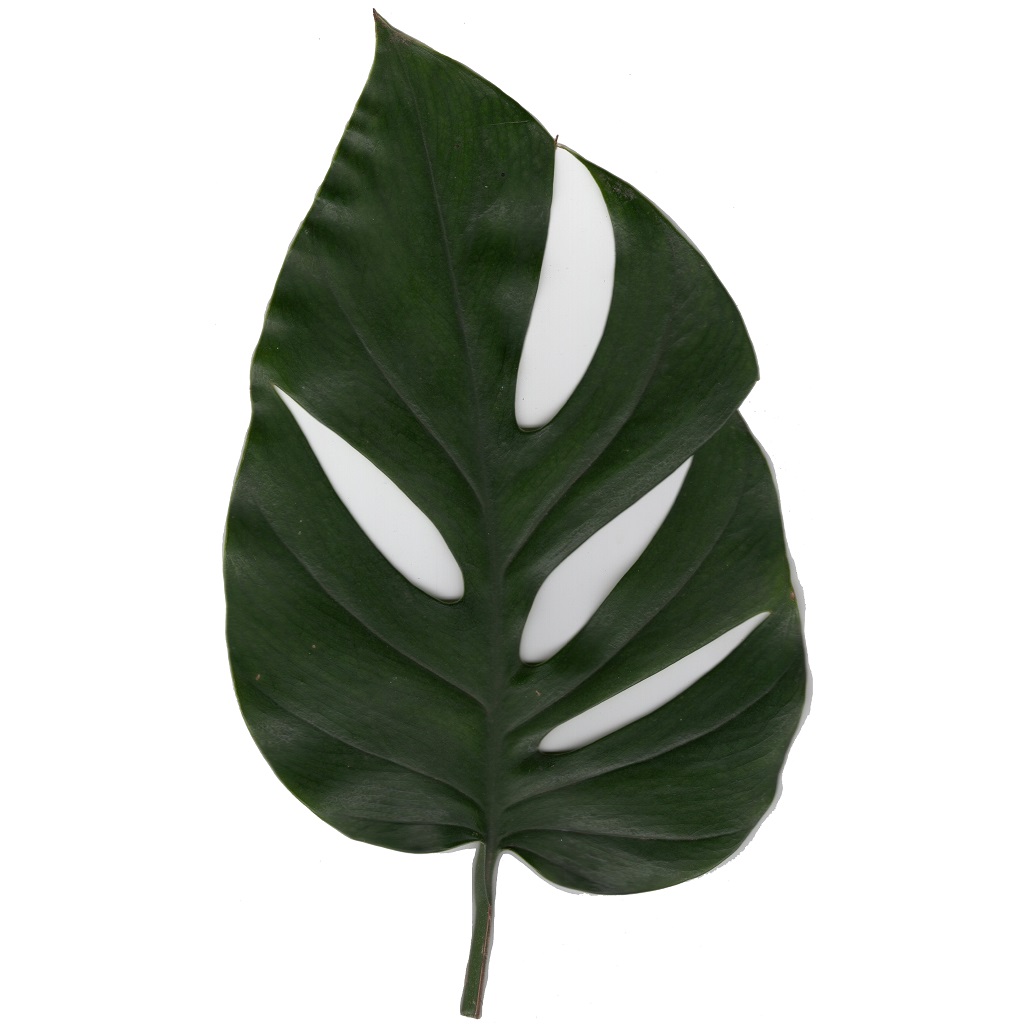

Supplement: S1 Data — (ZIP) [file pone.0293596.s001.zip › S1_data/Rhaphidophora tetrasperma.jpg]

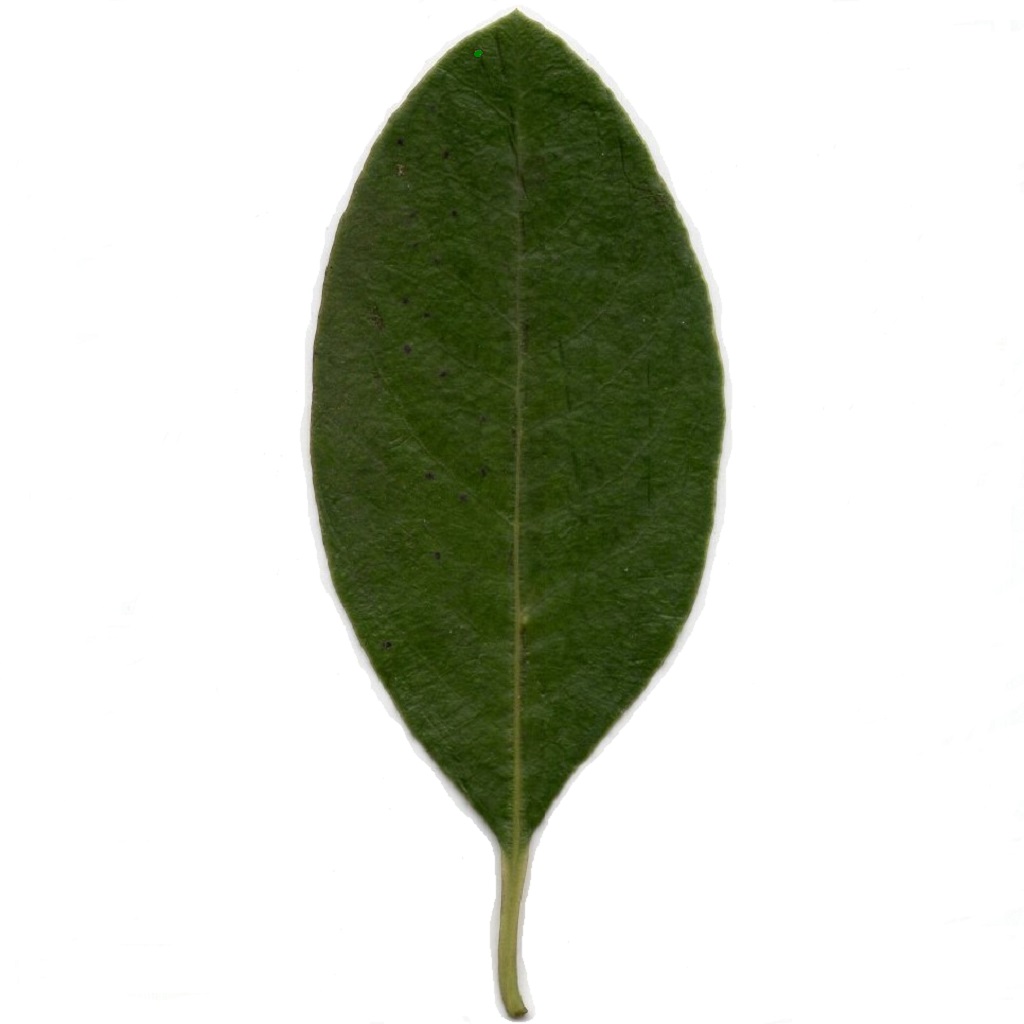

Supplement: S1 Data — (ZIP) [file pone.0293596.s001.zip › S1_data/Santalum album.jpg]

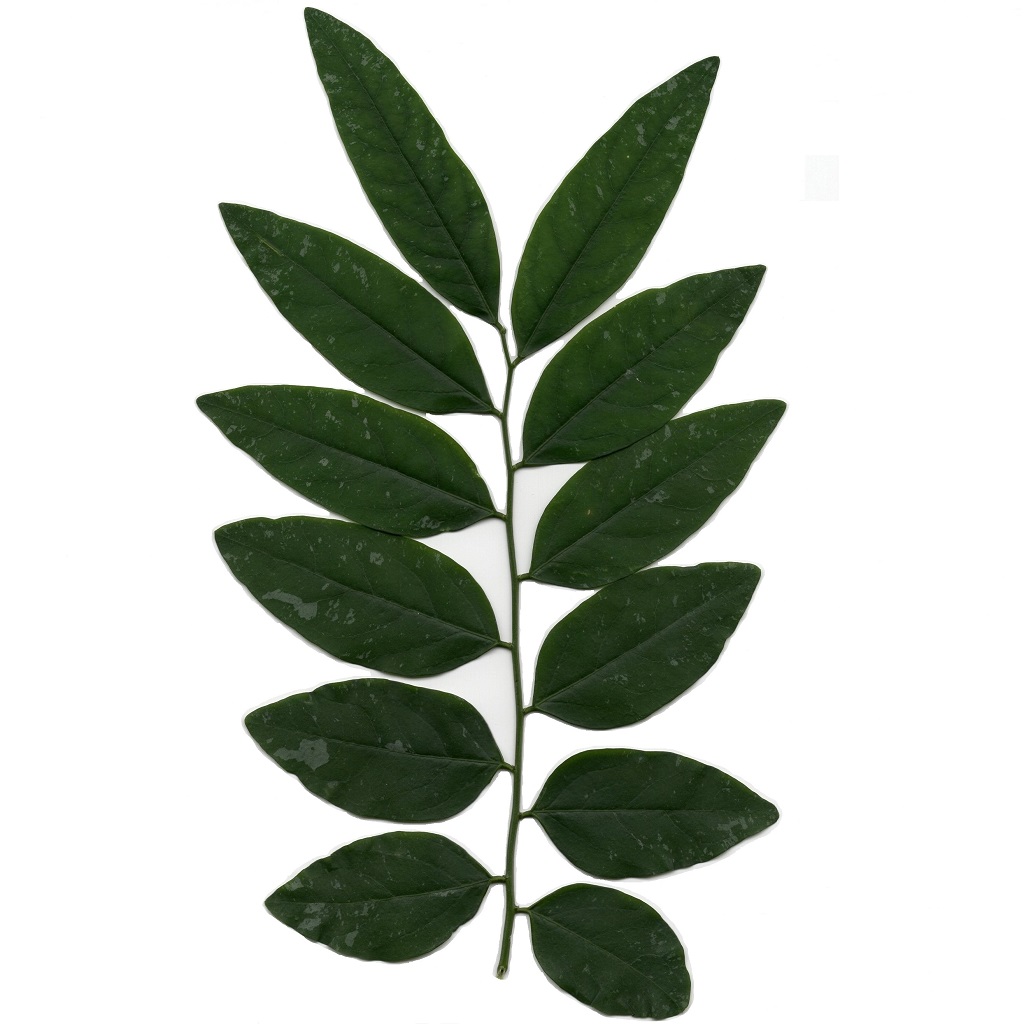

Supplement: S1 Data — (ZIP) [file pone.0293596.s001.zip › S1_data/Sauropus androgynus.jpg]

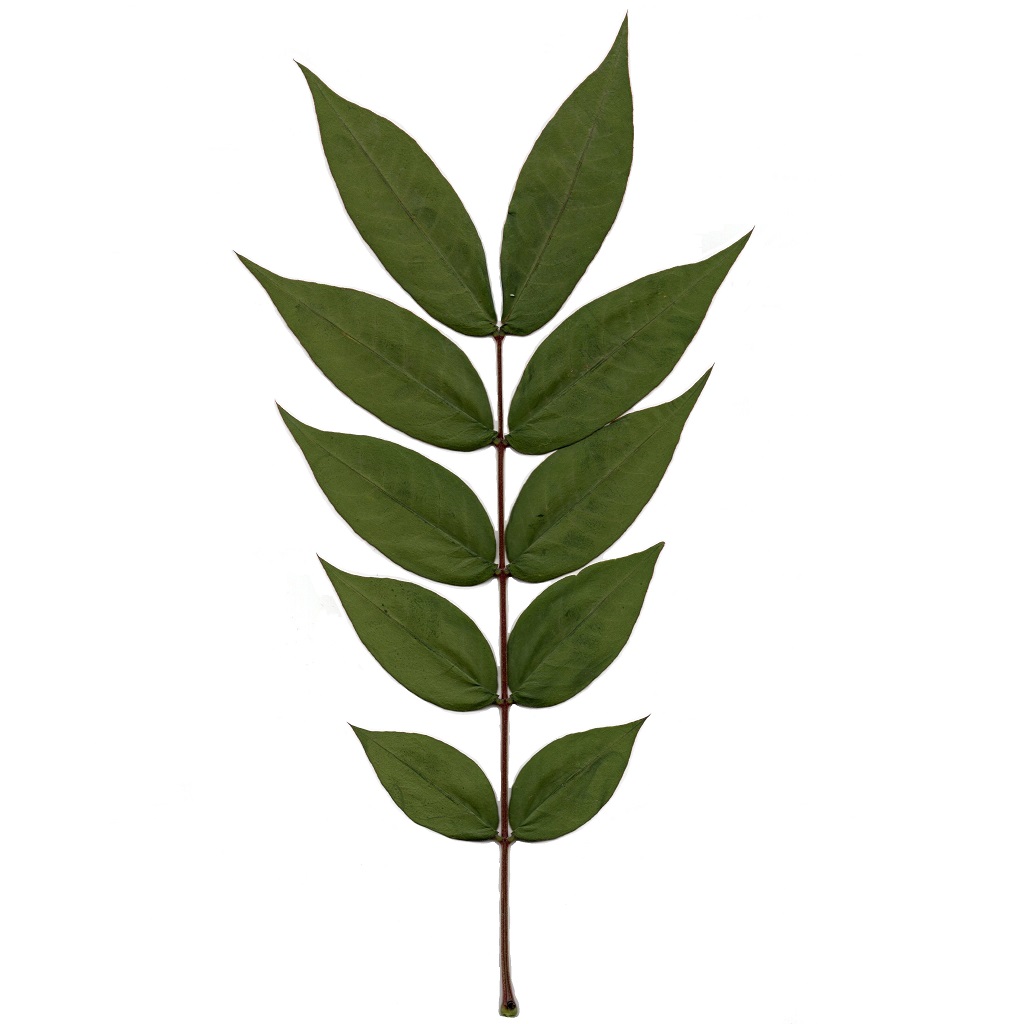

Supplement: S1 Data — (ZIP) [file pone.0293596.s001.zip › S1_data/Senna occidentalis.jpg]

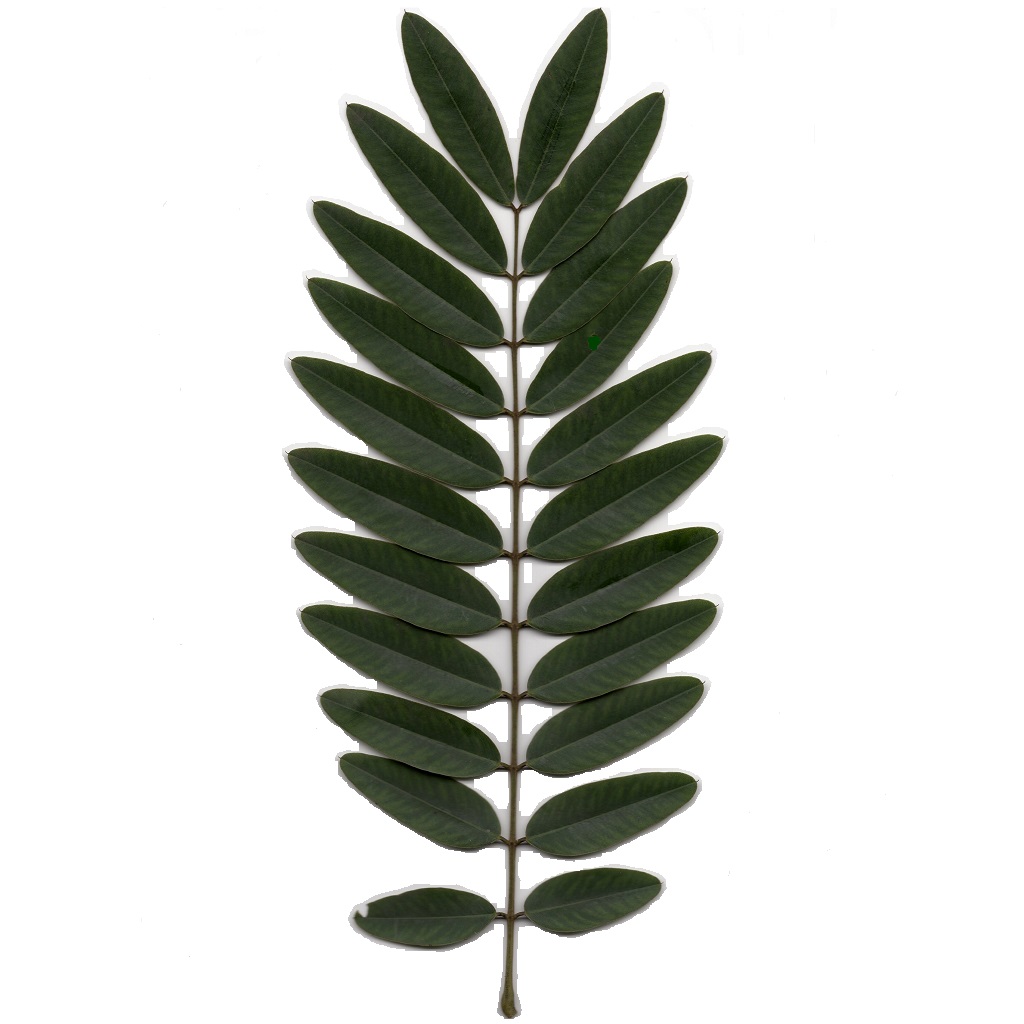

Supplement: S1 Data — (ZIP) [file pone.0293596.s001.zip › S1_data/Senna siamea.jpg]

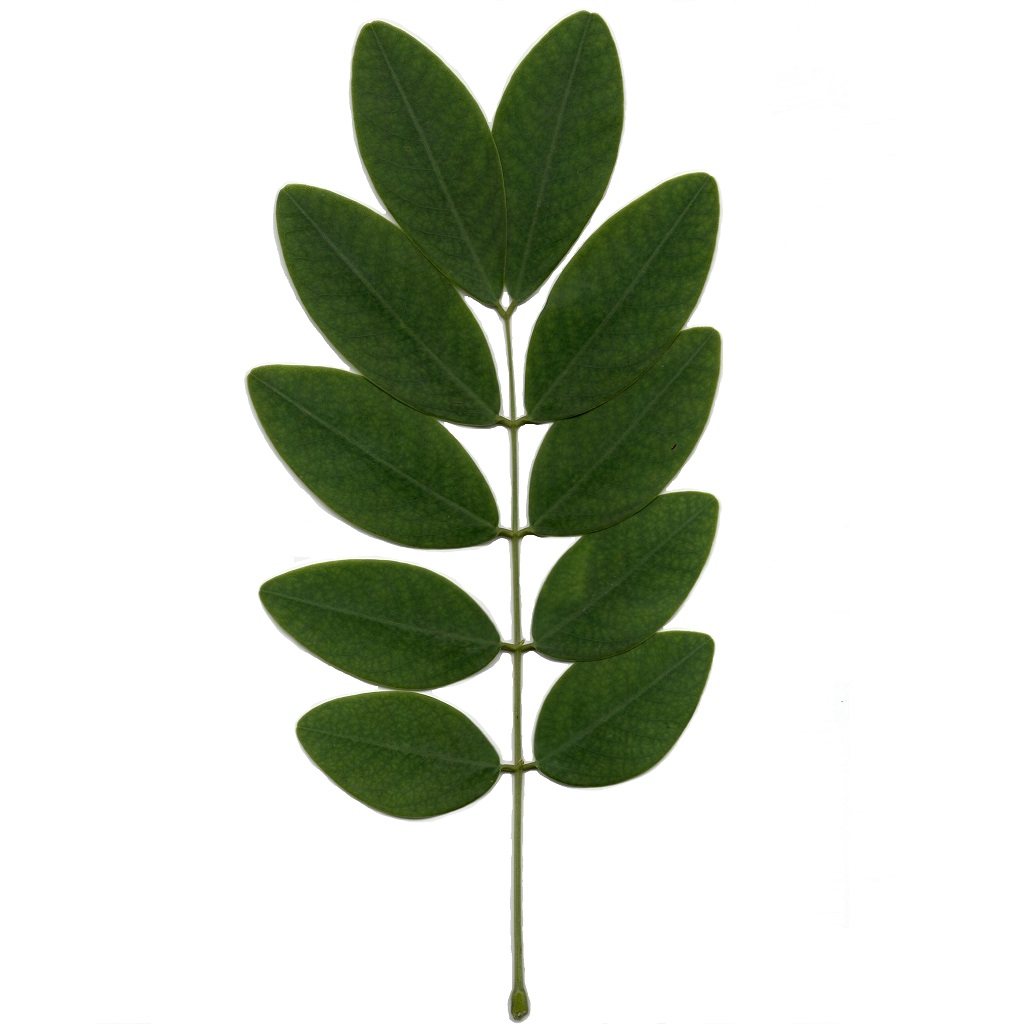

Supplement: S1 Data — (ZIP) [file pone.0293596.s001.zip › S1_data/Senna surattensis.jpg]

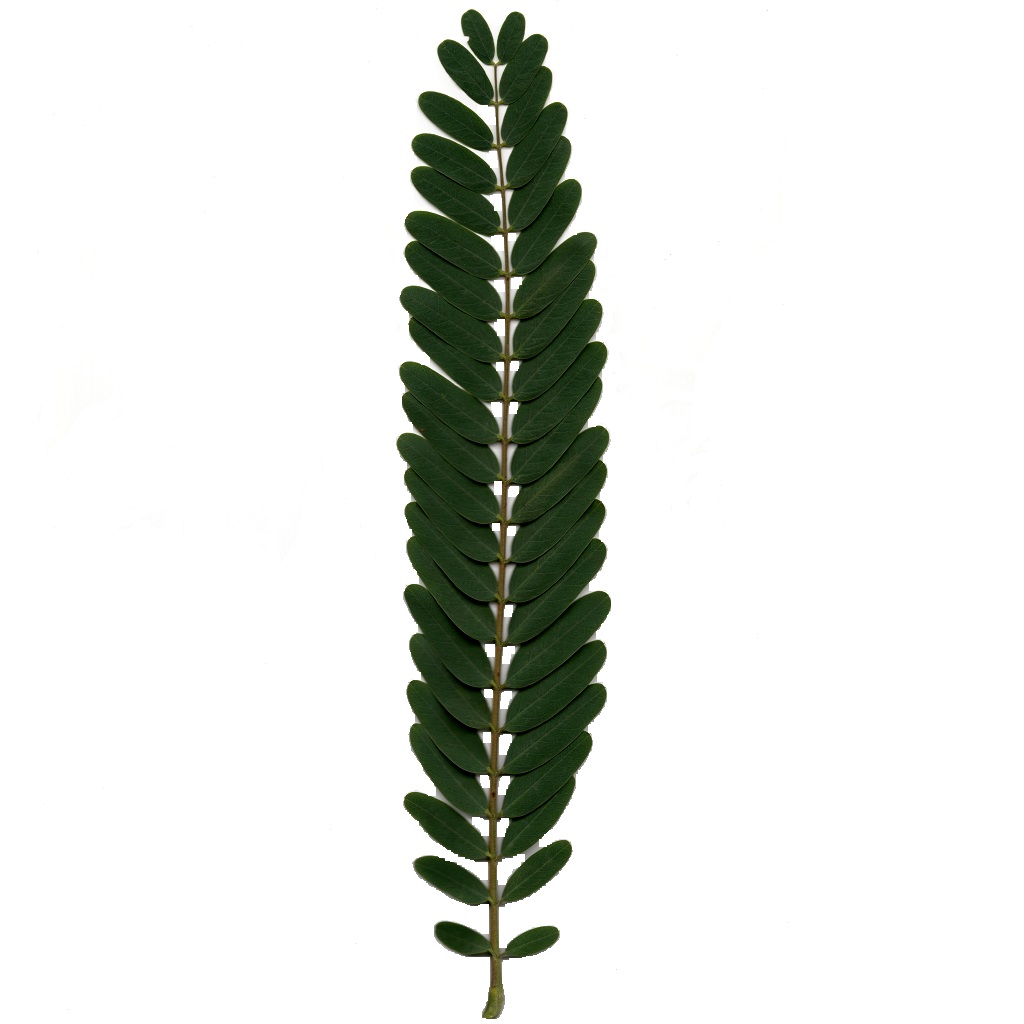

Supplement: S1 Data — (ZIP) [file pone.0293596.s001.zip › S1_data/Sesbania grandiflora.jpg]

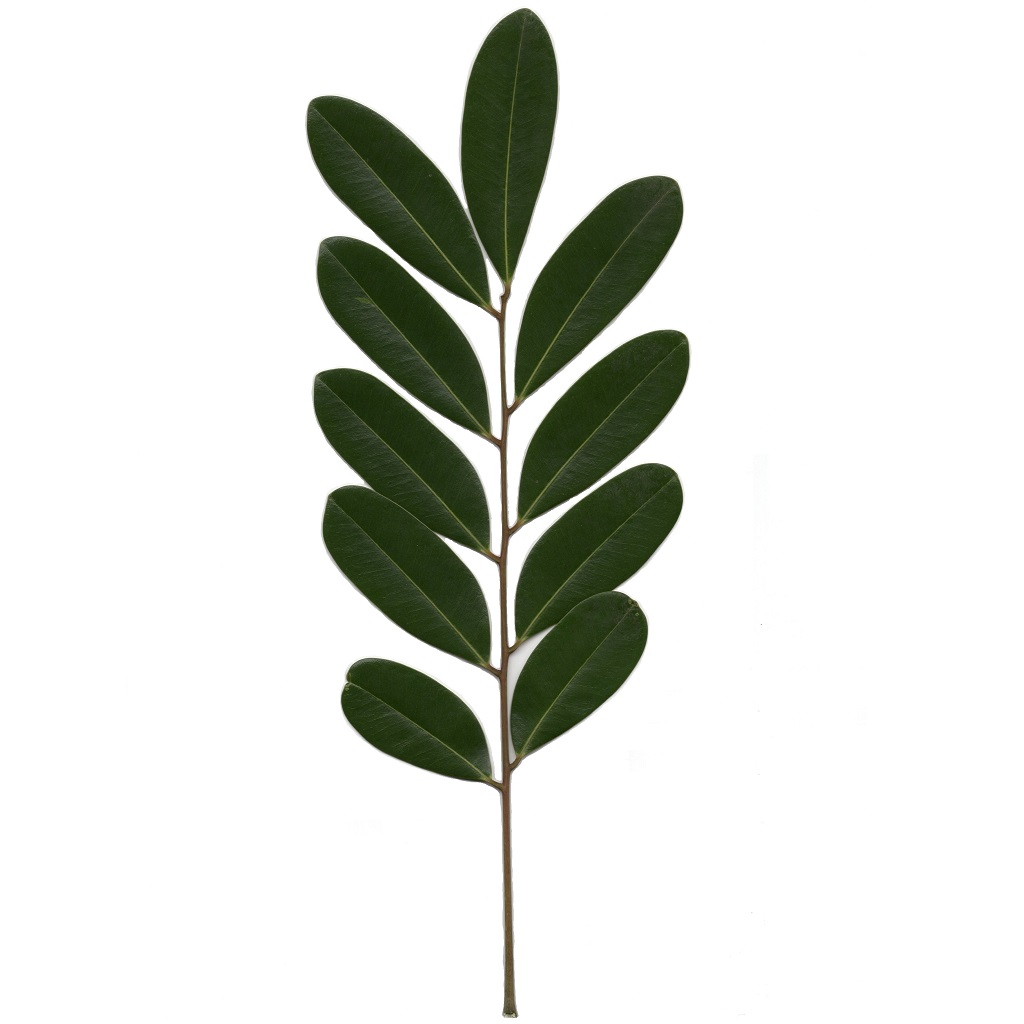

Supplement: S1 Data — (ZIP) [file pone.0293596.s001.zip › S1_data/Simarouba glauca.jpg]

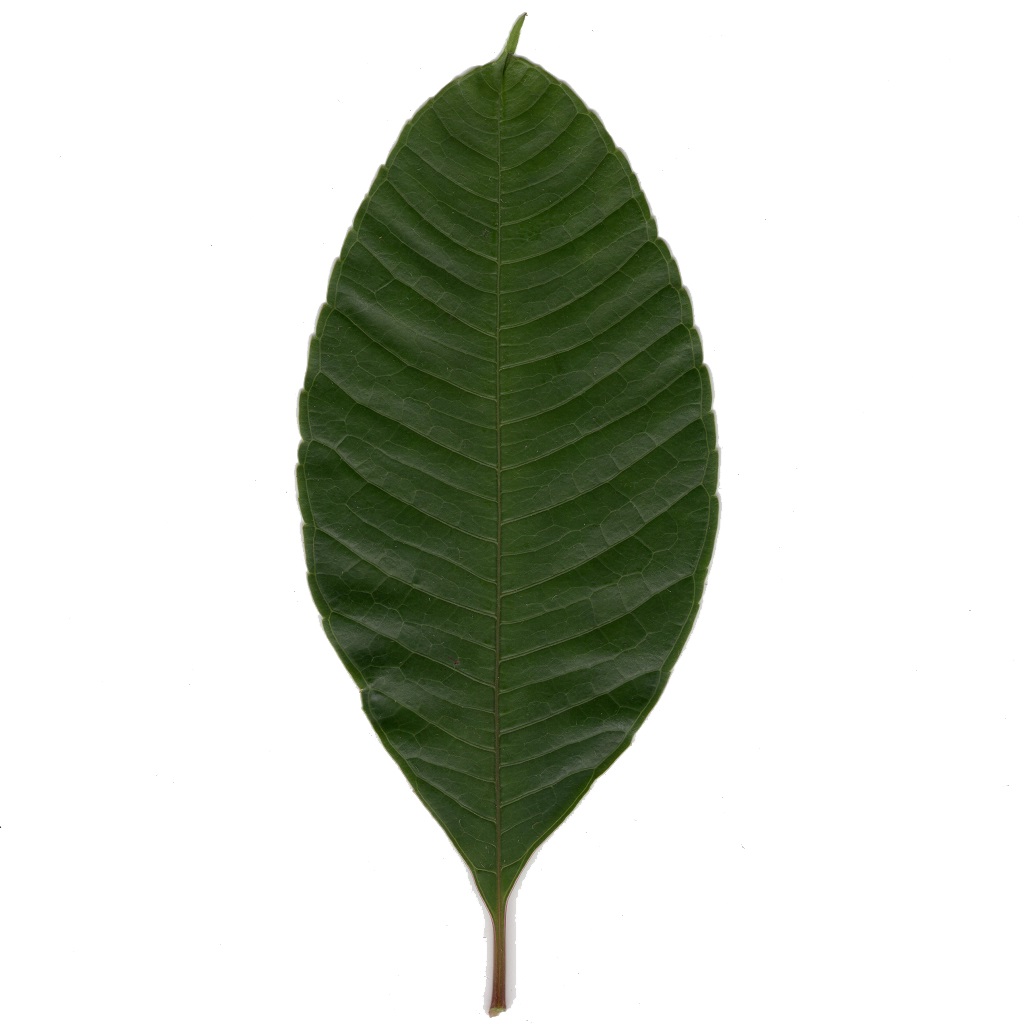

Supplement: S1 Data — (ZIP) [file pone.0293596.s001.zip › S1_data/Spondias pinnata.jpg]

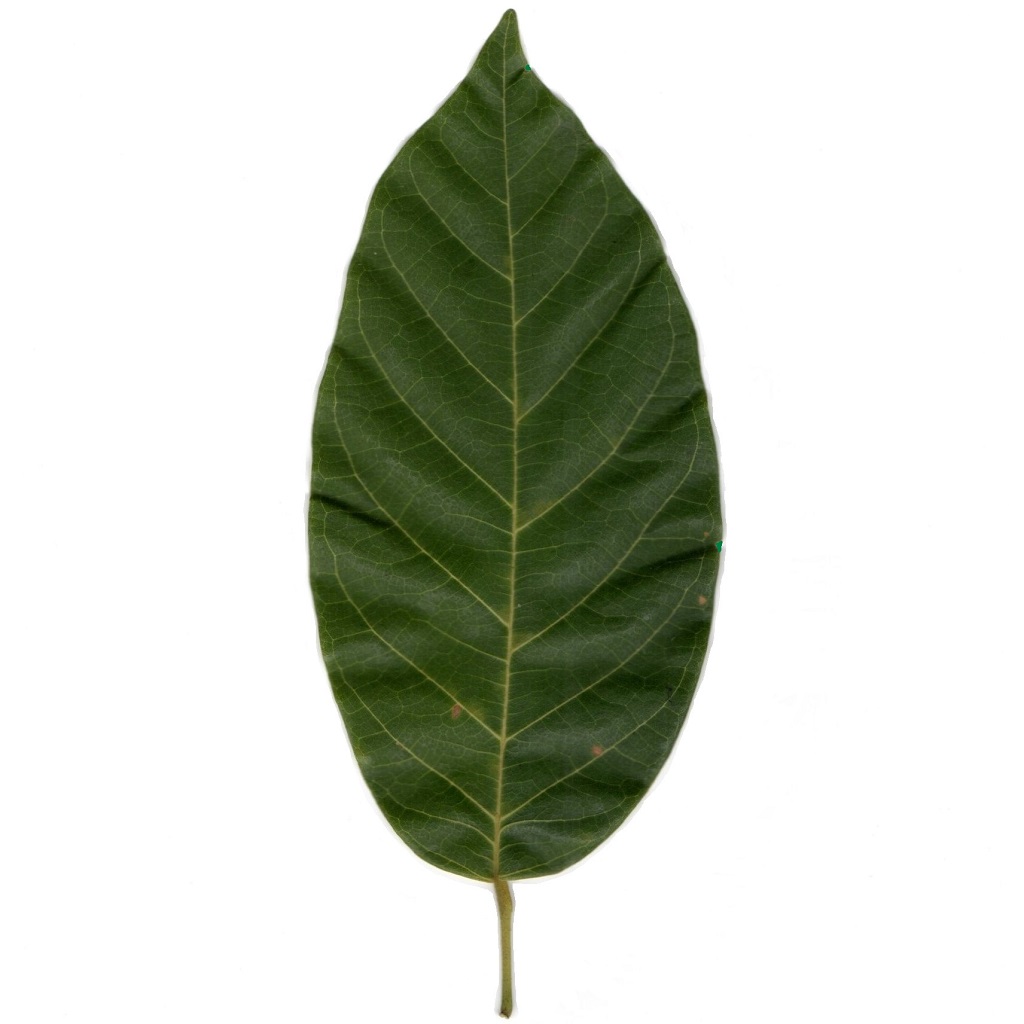

Supplement: S1 Data — (ZIP) [file pone.0293596.s001.zip › S1_data/Sterculia balanghas.jpg]

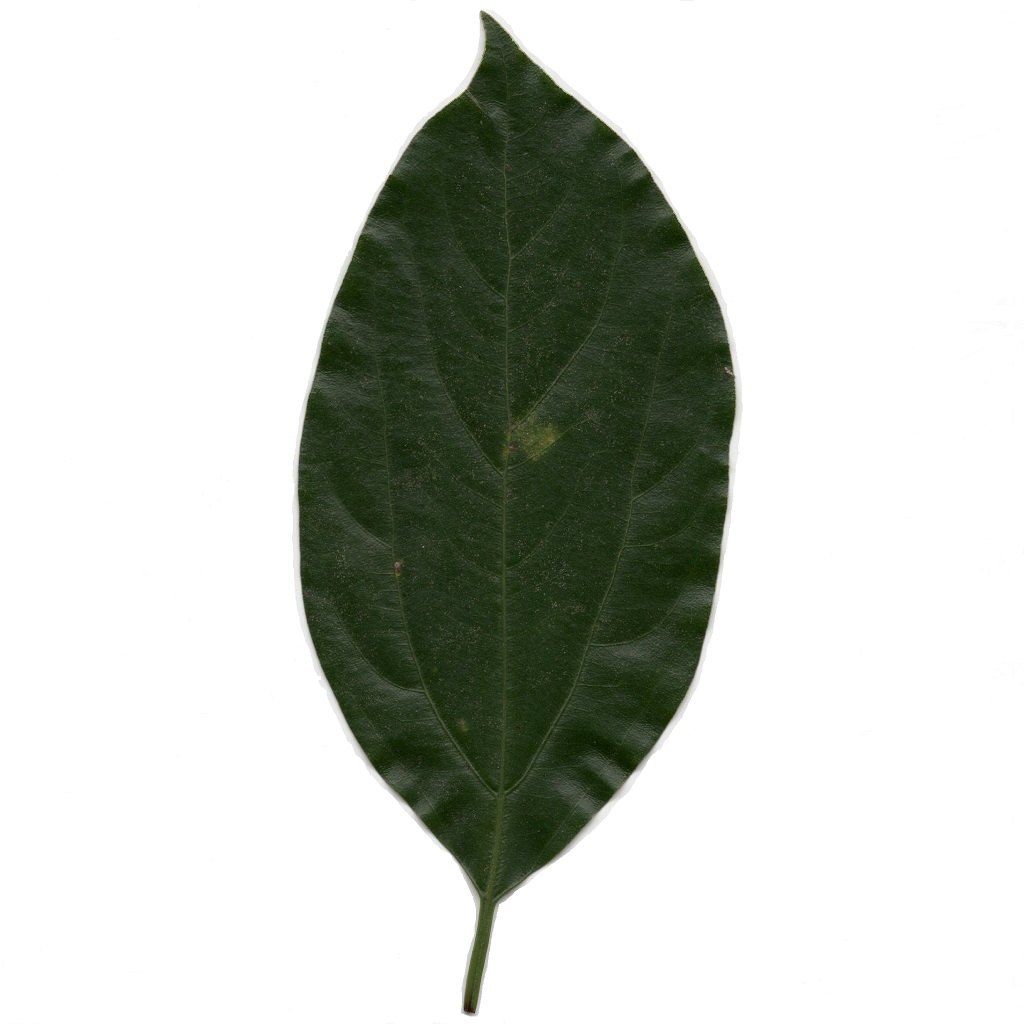

Supplement: S1 Data — (ZIP) [file pone.0293596.s001.zip › S1_data/Strychnos nux-vomica.jpg]

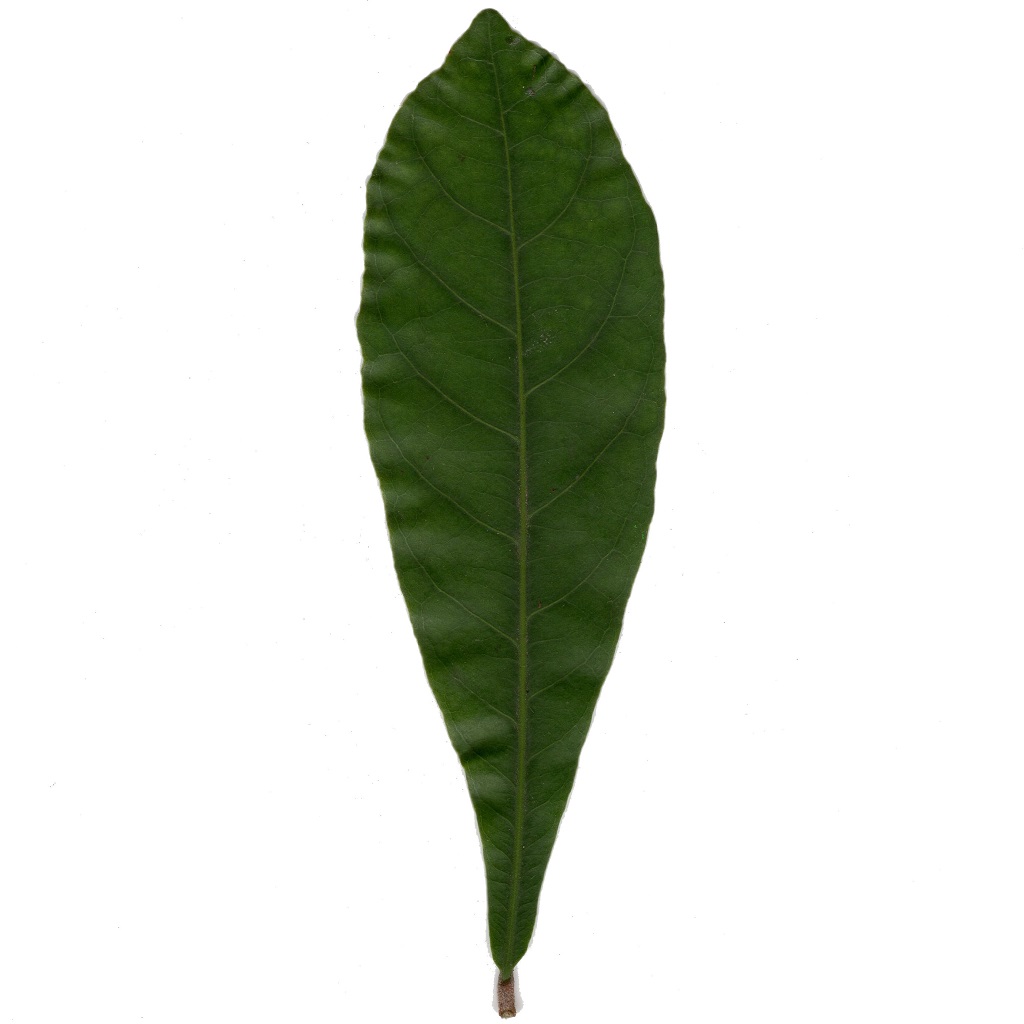

Supplement: S1 Data — (ZIP) [file pone.0293596.s001.zip › S1_data/Synsepalum dulcificum.jpg]

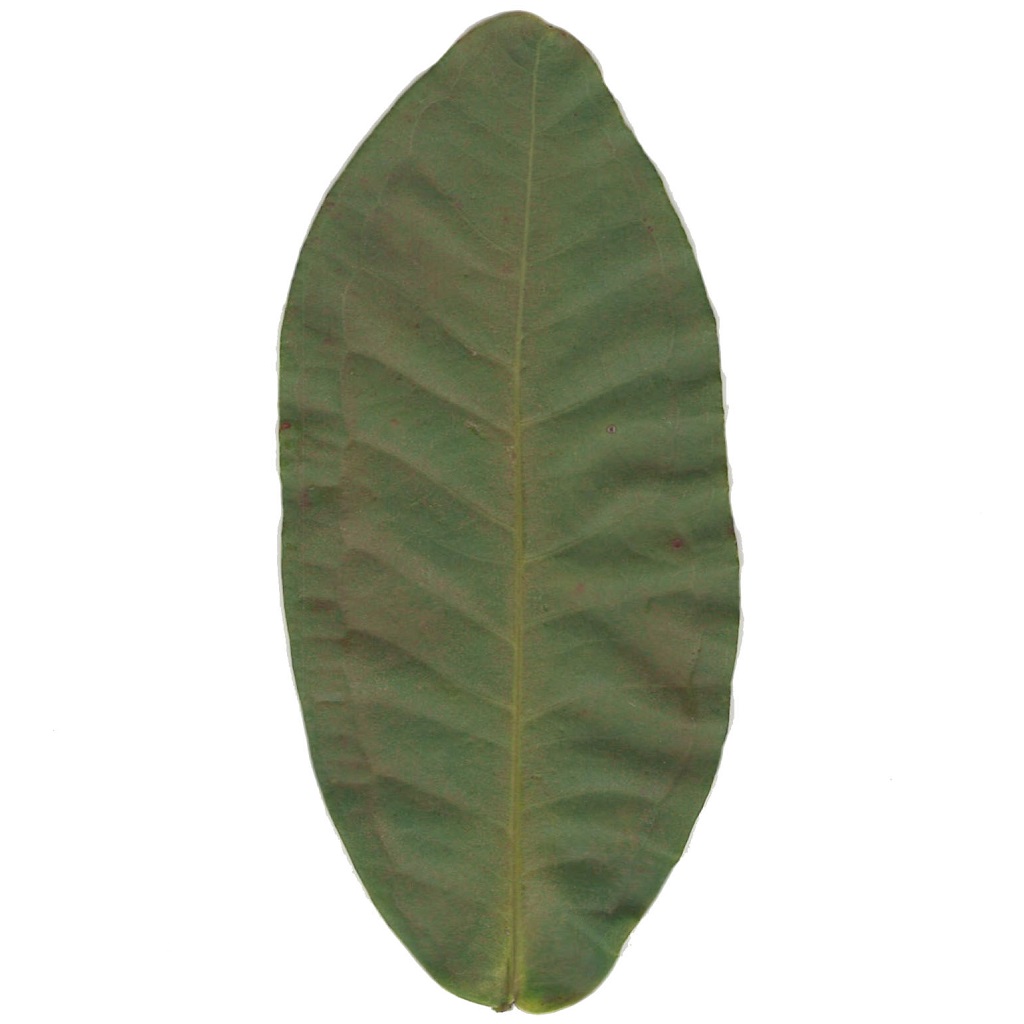

Supplement: S1 Data — (ZIP) [file pone.0293596.s001.zip › S1_data/Syzygium aqueum.jpg]

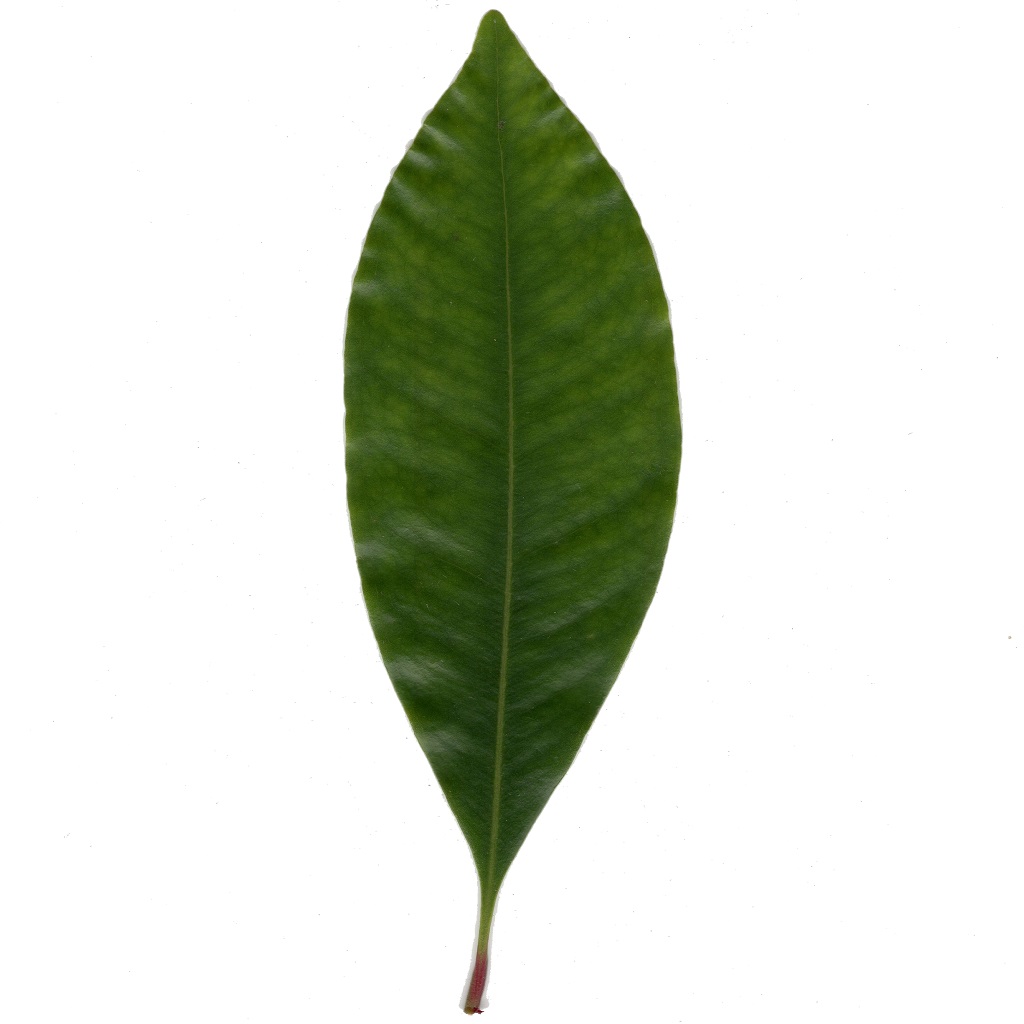

Supplement: S1 Data — (ZIP) [file pone.0293596.s001.zip › S1_data/Syzygium aromaticum.jpg]

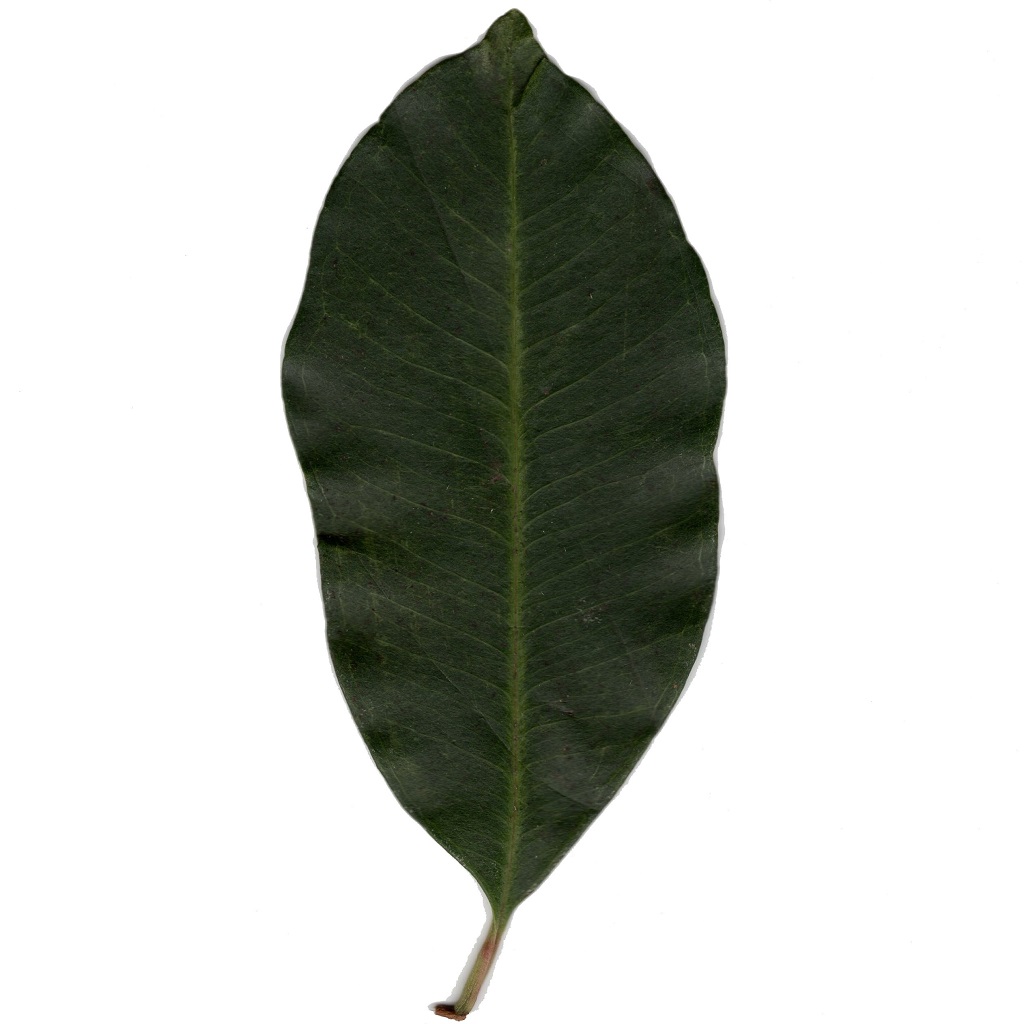

Supplement: S1 Data — (ZIP) [file pone.0293596.s001.zip › S1_data/Syzygium cumini.jpg]

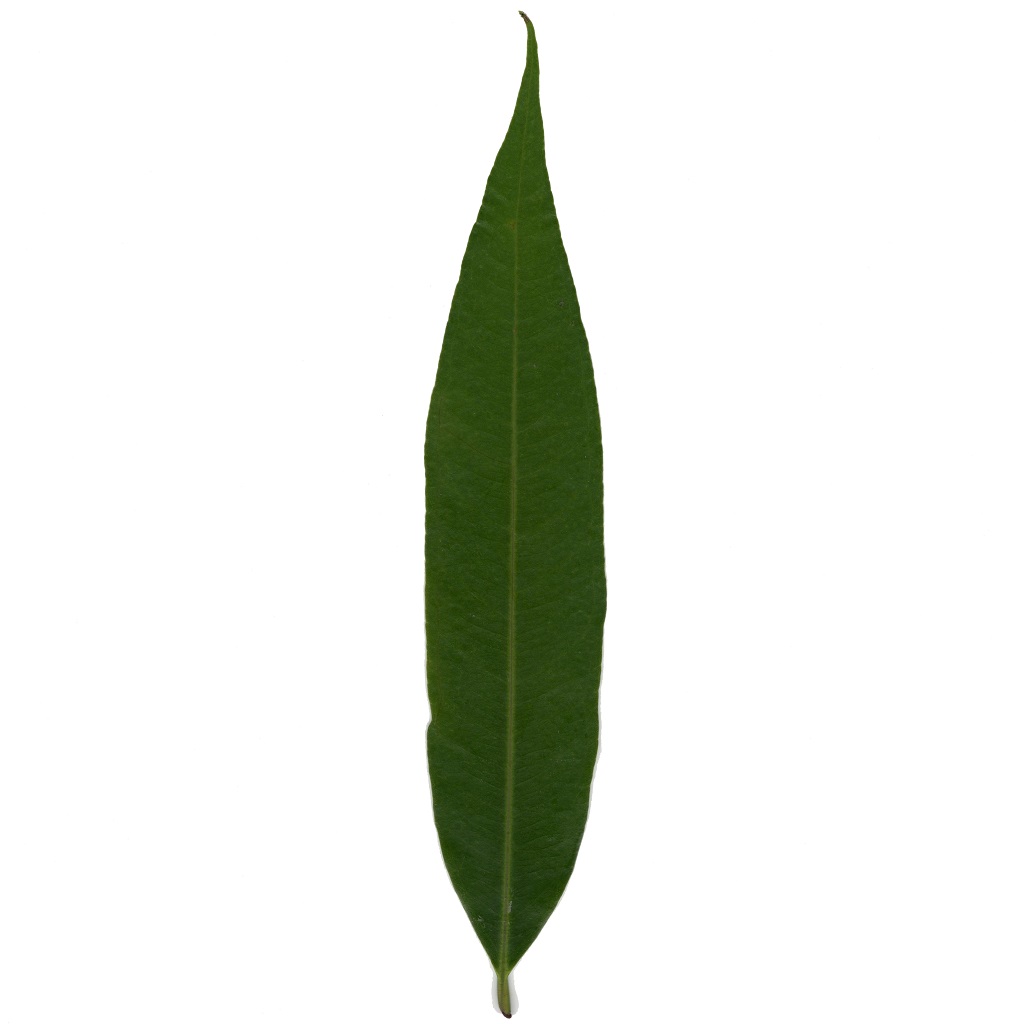

Supplement: S1 Data — (ZIP) [file pone.0293596.s001.zip › S1_data/Syzygium jambos.jpg]

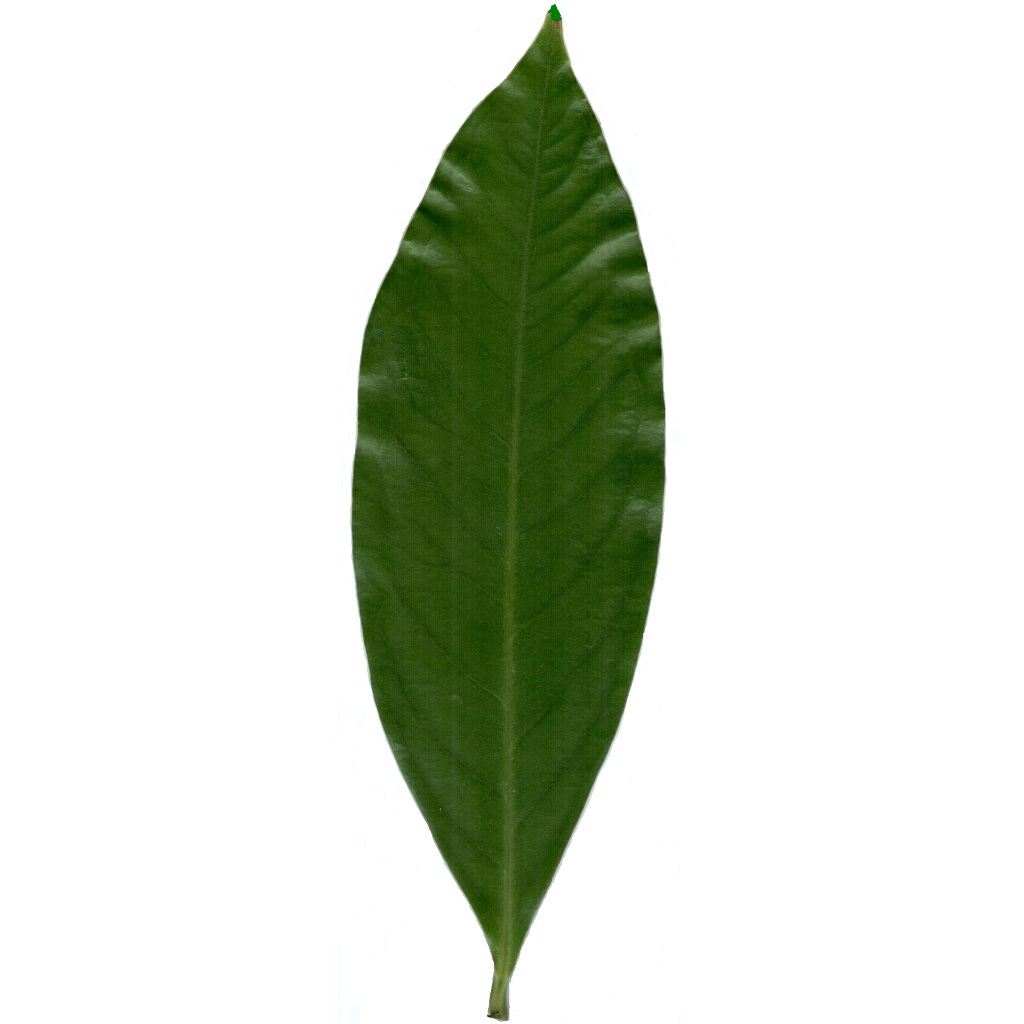

Supplement: S1 Data — (ZIP) [file pone.0293596.s001.zip › S1_data/Syzygium malaccense.jpg]

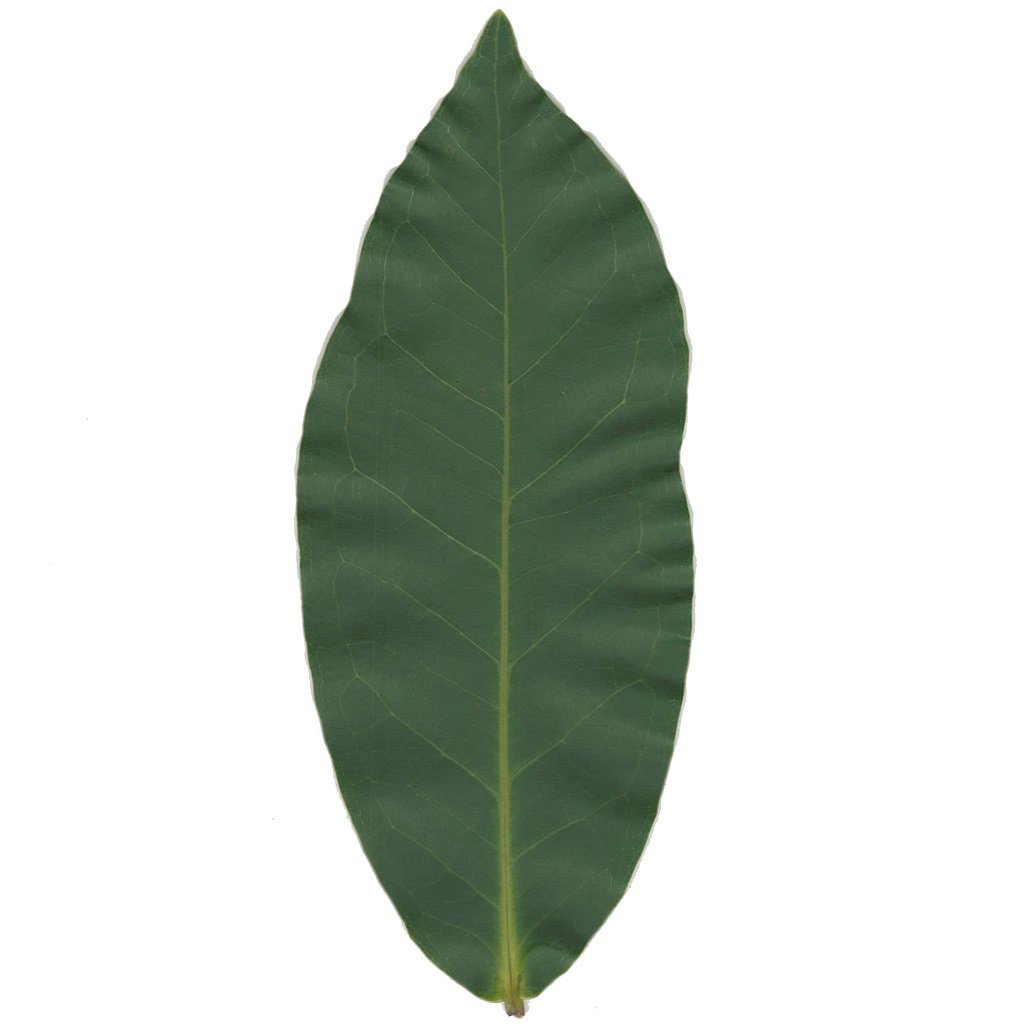

Supplement: S1 Data — (ZIP) [file pone.0293596.s001.zip › S1_data/Syzygium samarangense.jpg]
