# Supplementary material for: Geometric entropy of plant leaves: A measure of morphological complexity
Source: PLoS One. 2024 Jan 2;19(1):e0293596. doi: 10.1371/journal.pone.0293596 (PMC10760904; doi:10.1371/journal.pone.0293596)

PLOS ONE

Geometric Entropy of plant leaves: A measure of morphological complexity

--Manuscript Draft--

**Manuscript Number:**	PONE-D-23-07968
**Article Type:**	Research Article
**Full Title:**	Geometric Entropy of plant leaves: A measure of morphological complexity
**Short Title:**	Geometric Entropy of plant leaves
**Corresponding Author:**	Jaishanker Raghunathan Nair, Ph.D.<br>Digital University Kerala<br>Thiruvananthapuram, Kerala INDIA
**Keywords:**	Complexity, Leaf morphology, Geometric entropy, Leaf dissection index, Diffuse interfaces
**Abstract:**	Shape is an objective characteristic of an object. A boundary separates a physical object from its surroundings. It defines the shape and regulates energy flux into and from an object. Visual perception of a definite shape (geometry) of physical objects is an abstraction. While the perceived geometry at an object's sharp interface (macro) creates a Euclidian illusion of actual shape, the notion of diffuse interfaces (micro) allows an understanding of the realistic form of objects. Here we formulate a dimensionless geometric entropy of plant leaves (SL) by a 2-D description of a phase-field function. We applied this method to 109 tropical plant leaf images. SLwas estimated from the leaf perimeter (P) and leaf area (A). It correlates positively with a fractal dimensional measure of leaf complexity, viz., segmental fractal complexity. Leaves with a higher P: A ratio has higher SL and possesses complex morphology. The univariate cluster analysis of SL reveals the taxonomic relationship among the leaf shapes at the generic level. An increase in SL of plant leaves could be an evolutionary strategy. The results of morphological complexity presented in this paper will trigger discussion on the causal links between leaf adaptive stability/efficiency and complexity. We present SL as a derived plant trait to describe plant leaf complexity and adaptive stability. Integrating SL into other leaf physiological measures will help understand energy dynamics and information flow in ecological systems.
**Order of Authors:**	Vishnu M
	Sajeev C Rajan
	Jaishanker Raghunathan Nair, Ph.D.
**Additional Information:**	
**Question**	**Response**
**Financial Disclosure**<br><br>Enter a financial disclosure statement that describes the sources of funding for the work included in this submission. Review the submission guidelines for detailed requirements. View published research articles from *PLOS ONE* for specific examples.<br><br>This statement is required for submission and **will appear in the published article** if the submission is accepted. Please make sure it is accurate.	The author(s) received no specific funding for this work.

Powered by Editorial Manager® and ProduXion Manager® from Aries Systems Corporation

**Unfunded studies**<br>Enter: *The author(s) received no specific funding for this work.*<br><br>**Funded studies**<br>Enter a statement with the following details:<br>• Initials of the authors who received each award<br>• Grant numbers awarded to each author<br>• The full name of each funder<br>• URL of each funder website<br>• Did the sponsors or funders play any role in the study design, data collection and analysis, decision to publish, or preparation of the manuscript?<br>• **NO** - Include this sentence at the end of your statement: *The funders had no role in study design, data collection and analysis, decision to publish, or preparation of the manuscript.*<br>• **YES** - Specify the role(s) played.<br><br>* typeset	
**Competing Interests**<br><br>Use the instructions below to enter a competing interest statement for this submission. On behalf of all authors, disclose any competing interests that could be perceived to bias this work—acknowledging all financial support and any other relevant financial or non-financial competing interests.<br><br>This statement is required for submission and **will appear in the published article** if the submission is accepted. Please make sure it is accurate and that any funding sources listed in your Funding Information later in the submission form are also declared in your Financial Disclosure statement.<br><br>View published research articles from *PLOS ONE* for specific examples.	The authors have declared that no competing interests exist.

Powered by Editorial Manager® and ProduXion Manager® from Aries Systems Corporation

**NO authors have competing interests**<br><br>Enter: *The authors have declared that no competing interests exist.*<br><br>**Authors with competing interests**<br><br>Enter competing interest details beginning with this statement:<br><br>*I have read the journal's policy and the authors of this manuscript have the following competing interests: [insert competing interests here]*<br><br>* typeset	
**Ethics Statement**<br><br>Enter an ethics statement for this submission. This statement is required if the study involved:<br><br>• Human participants<br>• Human specimens or tissue<br>• Vertebrate animals or cephalopods<br>• Vertebrate embryos or tissues<br>• Field research<br><br>Write "N/A" if the submission does not require an ethics statement.<br><br>General guidance is provided below. Consult the submission guidelines for detailed instructions. **Make sure that all information entered here is included in the Methods section of the manuscript.**	N/A

Powered by Editorial Manager® and ProduXion Manager® from Aries Systems Corporation

**Format for specific study types**<br><br>**Human Subject Research (involving human participants and/or tissue)**<br>• Give the name of the institutional review board or ethics committee that approved the study<br>• Include the approval number and/or a statement indicating approval of this research<br>• Indicate the form of consent obtained (written/oral) or the reason that consent was not obtained (e.g. the data were analyzed anonymously)<br><br>**Animal Research (involving vertebrate animals, embryos or tissues)**<br>• Provide the name of the Institutional Animal Care and Use Committee (IACUC) or other relevant ethics board that reviewed the study protocol, and indicate whether they approved this research or granted a formal waiver of ethical approval<br>• Include an approval number if one was obtained<br>• If the study involved *non-human primates*, add *additional details* about animal welfare and steps taken to ameliorate suffering<br>• If anesthesia, euthanasia, or any kind of animal sacrifice is part of the study, include briefly which substances and/or methods were applied<br><br>**Field Research**<br><br>Include the following details if this study involves the collection of plant, animal, or other materials from a natural setting:<br>• Field permit number<br>• Name of the institution or relevant body that granted permission	
**Data Availability**<br><br>Authors are required to make all data underlying the findings described fully available, without restriction, and from the time of publication. PLOS allows rare exceptions to address legal and ethical concerns. See the PLOS Data Policy and FAQ for detailed information.	No - some restrictions will apply

A Data Availability Statement describing where the data can be found is required at submission. Your answers to this question constitute the Data Availability Statement and **will be published in the article**, if accepted.<br><br>**Important:** Stating 'data available on request from the author' is not sufficient. If your data are only available upon request, select 'No' for the first question and explain your exceptional situation in the text box.<br><br>Do the authors confirm that all data underlying the findings described in their manuscript are fully available without restriction?	
**Describe where the data may be found in full sentences. If you are copying our sample text, replace any instances of XXX with the appropriate details.**<br><br>• If the data are **held or will be held in a public repository**, include URLs, accession numbers or DOIs. If this information will only be available after acceptance, indicate this by ticking the box below. For example: *All XXX files are available from the XXX database (accession number(s) XXX, XXX.*).<br>• If the data are all contained **within the manuscript and/or Supporting Information files**, enter the following: *All relevant data are within the manuscript and its Supporting Information files.*<br>• If neither of these applies but you are able to provide **details of access elsewhere**, with or without limitations, please do so. For example:<br><br>*Data cannot be shared publicly because of [XXX]. Data are available from the XXX Institutional Data Access / Ethics Committee (contact via XXX) for researchers who meet the criteria for access to confidential data.*<br><br>*The data underlying the results presented in the study are available from (include the name of the third party*	The research was carried out as a part of the Ph.D. work. The work is not part of any research project. The author(s) received no specific funding for this work and have no option to seek funding to cover the Data Publishing Charges in data repositories.<br><br>The data that support the findings of this study are available upon reasonable request from the authors.

and contact information or URL).<br>• This text is appropriate if the data are owned by a third party and authors do not have permission to share the data.<br><br>* typeset	
Additional data availability information:	Tick here if your circumstances are not covered by the questions above and you need the journal's help to make your data available.

Powered by Editorial Manager® and ProduXion Manager® from Aries Systems Corporation

Manuscript Click here to access/download;Manuscript;Manuscript.docx

Geometric Entropy of plant leaves: A measure of morphological complexity

Vishnu M[1,2,3], Sajeev C Rajan[1], Jaishanker R[1,3,*]

[1]C V Raman Laboratory of Ecological Informatics, Indian Institute of Information Technology and Management - Kerala, Trivandrum, Kerala - 695581, India

[2]Cochin University of Science and Technology, Cochin, Kerala - 682022, India

[3]Kerala University of Digital Sciences, Innovation and Technology, Technopark Phase - IV, Thiruvananthapuram, Kerala - 695317, India

[*]Corresponding author

E-mail: jrnair@duk.ac.in (JR)

Abstract

Shape is an objective characteristic of an object. A boundary separates a physical object from its surroundings. It defines the shape and regulates energy flux into and from an object. Visual perception of a definite shape (geometry) of physical objects is an abstraction. While the perceived geometry at an object's sharp interface (macro) creates a Euclidian illusion of actual shape, the notion of diffuse interfaces (micro) allows an understanding of the realistic form of objects. Here we formulate a dimensionless geometric entropy of plant leaves (S_L) by a 2-D description of a phase-field function. We applied this method to 109 tropical plant leaf images. S_L was estimated from the leaf perimeter (P) and leaf area (A). It correlates positively with a fractal dimensional measure of leaf complexity, viz., segmental fractal complexity. Leaves with a higher $P: A$ ratio has higher S_L and possesses complex morphology. The univariate cluster analysis of S_L reveals the taxonomic relationship among the leaf shapes at the generic level. An increase in S_L of plant leaves could be an evolutionary strategy. The results of morphological complexity presented in this paper will trigger discussion on the causal links between leaf adaptive stability/efficiency and complexity. We present S_L as a derived plant trait to describe plant leaf complexity and adaptive stability. Integrating S_L into other leaf physiological measures will help understand energy dynamics and information flow in ecological systems.

Keywords: Complexity, Leaf morphology, Geometric entropy, Leaf dissection index, Diffuse interfaces

Introduction

Nature has invested heavily in diversity. It manifests along multiple dimensions (phenotypic, physiological) and tiers (molecular, individual, population, community). Physical appearance (morphology) is the most obvious trait that can be used to differentiate higher forms of life. The leaf shape of Angiosperms (flowering plants) is an easily discernable plant trait widely used by taxonomists to characterise plant species [1,2]. The different shapes of leaves have evolved through natural selection. These abstract forms (shapes) are not random. They have a mathematical soul that defines their physical form. Fibonacci numbers [3], the golden ratio [4], and fractals [5] provide mathematical descriptions of how biomass is arranged in numerical and structured geometric forms.

Mass and shape are fundamental attributes of living organisms. Biomass limited by a boundary bestows shape to organisms. Living objects exchange energy and matter with surroundings across boundaries. The exchange of energy and matter underpins evolutionary [6] and ecological processes [7]. Mereotopology is a theory combining mereology and topology. It deals with relations of things: parts, wholes, and their boundaries [8]. Mereotopology qualitatively describes the static relationship between neighbouring things by logical expressions, either "true (1)" or "false (0)".

The phase-field concept rooted in mathematical physics also describes the boundaries of neighbouring things [9]. In contrast to Mereotopology, the phase-field concept quantitatively describes geometric shapes, boundaries, and their dynamic evolution (transitions between object and boundary) [10]. Modeling using the phase-field approach belongs to the class involving phase transitions between states. Description of the nature or shape of the transition region of the phase-field function is achieved by the statistical distribution of gradients in the transition region [11]. The concept was first implemented in describing the evolution of complex dendritic structures [12] and later gained the attention of the material science fraternity. Nowadays, phase-field models are widely used to describe complex shapes, boundaries, and evolution [13,14].

In-depth knowledge of shape and size is a prerequisite to understanding the interaction between objects and their environment. Shape perception approaches focus exclusively on geometric and computational tools. The shape of any physical object is a perception rendered by human vision. Markosian et al. [15] suggest that only objects with spatial locations be considered physical objects. However, spatial bounds alone do not convey information (here, shape) of a physical object. Also, relying on visual perception to conceive the idea of a boundary is unscientific. Visual demarcation at the sharp macroscale interface of an object returns an illusion. Such shapes demarcated as object boundaries will change with magnification. A realistic boundary can be perceived as diffuse microscale interfaces with finite thickness [16–18]. Here we present a case study in complexity and use the notion of microscale interfaces and phase field transitions to arrive at the geometric entropy of plant leaf shapes.

Plant leaves exemplify remarkable complexity. Leaves are the primary sites that regulate photosynthesis and energy transfer. The amount of solar radiation incident on leaves depends on the geometry and inclination of individual leaves [19]. Converting complex leaf forms into simple geometric shapes can give valuable insights into leaf-radiation interaction. It allows an in-depth study of geometry and energy by the basic properties of Euclidean shapes. Information and entropy unify the idea of geometry and energy in all biological systems [20,21]. Understanding the joint descriptions of information, entropy, and geometry will open discussions on the direct causal links between leaf stability/efficiency and the complexity of plant leaves. Until the dimensionless form of Bekenstein - Hawking entropy was constructed using a phase-field approach [22], only the notion of entropy in the 'heterogeneity' sense [23] was related to the perception of geometry. In this paper, we consider plant leaves to be made up of 2-D non-linear elements and derive their geometric entropy through mathematical formulations based on the geometric approach of Schmitz [22].

Leaf as a 2-D Geometric Object

Like any physical object, a plant leaf has a mass, momentum, and temperature. We attempt to describe the geometric entropy of an individual plant leaf by the mathematical information of physical objects gathered

from the formulation of the Bekenstein-Hawking entropy [22]. The formulation is purely geometrical and is devoid of any of the attributes of physical objects.

The mean thickness and laminar length/width of plant leaves are in the order of 10^{-4} *m* and 10^{-2} *m*, respectively [24]. Since the thickness of the leaves is relatively smaller than their laminar dimensions, the visual perception of leaf morphological features is more laminar. Thus leaves exhibit remarkable complexity more by variations at their margin, leading to lobes or serrations. 2- D leaf shape features described along the laminar direction can be considered an excellent candidate to discriminate the leaves from others. Hence, we consider the plant leaf as a 2-D structure for analytical purposes. A 2-D leaf is described by an area confined by a boundary - the leaf margin. The leaf boundary distinguishes the bulk of the leaf from its surrounding environment.

As stated earlier, we consider the leaf as 2-D object made of non-linear elements. Circular objects can be described using a continuous 2-D extension of Heaviside and phase-field functions [25,26], thereby constructing the geometric entropy at its diffuse interfaces.

Description of a Circular Object

The Heaviside step function ($H(x)$) and the Phase-field function ($\Phi(x)$) are essential in describing the physical states (presence or absence) of a system and are used in the modeling and mechanics of complex structures [27,28]. The sharp interface property of the $H(x)$ yields the value '1' wherever the object is present and is '0' elsewhere (Fig 1). The geometry of a circle can be described using this property. Boundaries distinguish an object ('1') from its non-object state ('0'). A circle is an area confined by its periphery (boundary). We consider the boundary of the circle as a sharp interface. Beyond their boundary, there will be no presence of the circle. Thus,

The area A of a circle with a radius r_0 in circular coordinates is given by $H(x)$ as:

$$A = \iint H(r - r_0) r dr d\theta$$

(1)

Fig 1. Visualisation of the Heaviside step function H(x) (in Orange line) and the Phase-field functions Φ(x) (in Blue line).

The sharp interface property of *H(x)* reduces the limits of the integral from zero to r_0:

$$A = 2\pi \int_0^{\infty} H(r - r_0) r dr = 2\pi \int_0^{r_0} r dr = \pi {r_0}^2$$

(2)

Similarly, the perimeter *P* of the circle can be calculated using the gradient of *H(x)*. Since the distributional derivative of the *H(x)* results in the definition of the Dirac delta function (*δ(x)*) [29], gradients of *H(x)* will only appear at the boundaries (r_0) of the circular object.

i.e

$$\delta(x) = \frac{dH(x)}{dx}$$

(3)

The perimeter *P* of the circle therefore calculated as:

$$P = \iint \delta(r - r_0) r dr d\theta = 2\pi r_0$$

(4)

In contrast to the *H(x)*, Phase-field functions *(Φ(x))* is based on a continuous transition between two states, '1' (presence of the object) and '0' (absence of object), within a small transition zone η (Fig 1). However, *Φ(x)* can be treated as a continuous and 2-D formulation of *H(x)* at a very narrow transition width η.

$$\Phi(r - r_0) \approx H(r - r_0)$$

(5)

and

$$\nabla\Phi(r - r_0) \approx \nabla H(r - r_0) = \delta(r - r_0)$$

(6)

The gradient ∇ describes the one-dimensional derivative in the radial direction.

The following section derives the geometric entropy of circular objects based on the geometric description of the transition region (diffuse interface) in a phase-field function.

Geometric Entropy of Circular Object

Entropy is a ubiquitous concept that reveals a complicated picture in almost all fields ranging from information [30,31], thermodynamics [32,33], biology [34,35], materials [36,37], and economics [38,39]. While some of these are probability distribution functions, others are not. However, irrespective of the notion, all are defined in terms of the well-known logarithmic expression,

$$S = -\sum_{i=0}^{N} \Phi_i ln\Phi_i$$

(7)

Here we consider the boundary of a circular object as a diffuse interface, which assumes an ideal mixing of an infinite number of equiprobable states of object/non-object (Φ_i). The geometric entropy (S_{GE}) at the

circle's interface (phase field) is deduced by the geometric description of the diffuse interface using the Temkin model [40].

The Temkin model describes the entropy of the diffuse interface layers as:

$$S = -\sum_{n=-\infty}^{+\infty} (\Phi_{n-1} - \Phi_n) ln\,(\Phi_{n-1} - \Phi_n) \tag{8}$$

The corresponding gradient of the probable states *(Φ_i)* can be defined by introducing the notion of discretisation length (*l*) between two adjacent layers as (Fig 2):

$$d\Phi_n = \Phi_n - \Phi_{n-1} = \int_{(n-1)l}^{nl} \frac{d\Phi}{dr} dr = \frac{d\Phi_n}{dr}\int_{(n-1)l}^{nl} dr = l\frac{d\Phi_n}{dr} = l\nabla_r^n \Phi \tag{9}$$

Fig 2. Visualisation of the gradient Φ_n – Φ_{n-1} in Temkin's model of the entropy of a diffuse interface (Image concept from Schmitz [22]).

Therefore, the discrete entropy described in Eq (8) can be transformed into the continuous formulation for small discretisation length (*l*) and the infinite number of discrete cells (*n*) as:

$$S = -\sum_{n=-\infty}^{+\infty} \left\{l\frac{d\Phi(nl)}{dr}\right\} ln\left\{l\frac{d\Phi(nl)}{dr}\right\} \rightarrow -\int_{-\infty}^{+\infty} \left\{l\frac{d\Phi(nl)}{dr}\right\} ln\left\{l\frac{d\Phi(nl)}{dr}\right\} dn \tag{10}$$

$$\text{with } r(n) = r_0 + nl \qquad \text{and} \qquad dn = \frac{dr}{l} \tag{11}$$

Applying Eq (9)

$$S = -\int_{-\infty}^{+\infty} \{l\nabla_r \Phi(r - r_0)\}\, ln\{l\nabla_r \Phi(r - r_0)\} \frac{dr}{l}$$

(12)

Extending the entropy from one dimension to two dimensions in the Cartesian plane changes the radial product $l\nabla_r$ into the scalar product $\vec{l}\vec{\nabla}\phi$ and normalises the integration direction by some discretisation length as,

$$S = -\iint_{-\infty}^{+\infty} (\vec{l}\vec{\nabla}\phi)\, ln(\vec{l}\vec{\nabla}\phi) \frac{dx}{l_x}\frac{dy}{l_y}$$

(13)

We recall that the entropy formulation is purely geometrical and does not reveal any intrinsic structure (attributes) of the physical object. Thus, the discretisation lengths in the two dimensions can be considered to a generalised isotropic discretisation.

Taking isotropy of discretisation, i.e., $l_x = l_y = l_p$, changes the expression of S in the Cartesian plane into polar coordinates by,

$$S = -\iint (\vec{l}\vec{\nabla}\phi) ln(\vec{l}\vec{\nabla}\phi) \frac{rdrd\theta}{{l_p}^2}$$

(14)

Integrating over the angle $d\theta$ will give,

$$S = -2\pi \int_0^{\infty} \left(\vec{l}\vec{\nabla}\phi(r - r_0)\right) ln\left(\vec{l}\vec{\nabla}\phi(r - r_0)\right) \frac{rdr}{{l_p}^2}$$

(15)

Since finite values of $\nabla\phi$ can contribute to the integral only at the interface, a proportionality between the terms containing $\nabla\phi$ and the δ-function can be assumed for ϕ at small transition widths η.

$$\frac{l}{l_p}\left(\vec{l}\vec{\nabla}\phi(r-r_0)\right) ln\left(\vec{l}\vec{\nabla}\phi(r-r_0)\right) \propto \delta(r-r_0) \tag{16}$$

Including Eq (16) with an unknown constant of proportionality into Eq (15) yields,

$$S = -\frac{2\pi}{l_p}\int_0^\infty const \times \delta(r-r_0)rdr = -const \times \frac{2\pi r_0}{l_p} = -const \times \frac{P}{l_p} \tag{17}$$

where P is the perimeter of the circle.

The proportionality constant is estimated as *-1/4* in the formulation of Bekenstein-Hawking entropy of black holes by calculating the average gradient in diffuse interfaces [22].

Hence the geometric entropy of a circle takes the dimensionless form,

$$S_{GE} = \frac{1}{4} \times \frac{P}{l_p} \tag{18}$$

Geometric Entropy of Plant Leaf

Bulk and boundary constitute the structure of any 2-D object. S_{GE} developed at the 2-D interface is purely based on an informational approach and does not contain any temperature term. It is a configurational entropy that conveys information about shape (geometrical features). This information conveys the extent

of complexity/dissimilarity of shapes from the circularity. We consider the leaf-environment interface of the plant leaf as a narrow phase field and describe the structure of the geometric entropy of a plant leaf (S_L) by the S_{GE} of a circular object.

Generally, 2-D shape features are described only in the *x* and *y* direction. The parameters of S_L are subjective to the rationale of 2-D complexity and leaf characteristics. We consider the dimensions of the diffuse interface (in the *x* or *y* direction) to correspond to the leaf dimensions (length *L* or width *W*) (Fig 3). Finite element method (*FEM*) is an innovative concept to model the development of phase field surfaces. Taking the logic of isotropy of discretisation in 2-D non-Euclidean shapes from the finite element method [41], the generalised discretisation length (l_p) corresponds with the square root of the leaf area. Since the perimeter and leaf area play an important role in leaf physiology [42,43], we consider the two variables in geometric entropy (S_L) perimeter, and leaf area; as leaf traits. The perimeter is considered the leaf perimeter, and the discretisation length is the square root of the leaf area.

Fig 3. Visualisation of a narrow leaf-environment diffuse interface. We assume the dimensions of the diffuse interface (in *x* or *y* direction) correspond to the leaf dimensions (length *L* or width *W*).

The geometric entropy (S_L) of an ordinary leaf takes the form:

$$S_L = \frac{1}{4} \times \frac{P}{\sqrt{A}} \tag{19}$$

where *P* is the leaf perimeter and *A* is the leaf area.

Eq (19) resembles the Leaf Dissection Index (*LDI*) [44]. While *LDI* is commonly used to depict the complexity of plant leaf shape, we could not trace the assumptions and scientific derivation of *LDI*. Hence we consider Eq (19) as arriving at the geometric entropy through a scientific approach. In the following sections, we illustrate the use of geometric entropy (S_L) to analyse the complexity of plant leaf shapes.

Dataset and measurement

Mature, healthy leaves of 109 flat-leaved plant species in 40 families were collected from Trivandrum, Kerala, India (bounded by 8° 32' N, 77° 16' E and 8° 46' N, 76° 41' E) from June 2022 to December 2022 (Table 1). Trivandrum is situated on the southwest coast of India and has a tropical climate with diverse flora and fauna.

Table 1. Geometric entropy (S_L) and Segmental Fractal Complexity ($D_{\Sigma S}$) of the plant leaves collected from Trivandrum, Kerala, India.

Sl. No.	Plant Species	Genus	Family	Segmental Fractal Complexity ($D_{\Sigma S}$)	Geometric Entropy (S_L)	Leaf Type
1	*Justicia adhatoda L.*	Justicia	Acanthaceae	1.105	3.876	Simple
2	*Hydnocarpus pentandrus (Buch.-Ham.) Oken*	Hydnocarpus	Achariaceae	1.099	3.807	Simple
3	*Anacardium occidentale L.*	Anacardium	Anacardiaceae	1.057	2.73	Simple
4	*Mangifera indica L.*	Mangifera	Anacardiaceae	1.12	4.262	Simple, Narrow
5	*Spondias pinnata (L.fil.) Kurz*	Spondias	Anacardiaceae	1.092	3.215	Simple
6	*Annona muricata L.*	Annona	Annonaceae	1.067	3.029	Simple
7	*Annona reticulata L.*	Annona	Annonaceae	1.087	3.417	Simple
8	*Annona squamosa L.*	Annona	Annonaceae	1.081	3.413	Simple
9	*Monoon longifolium (Sonn.) B.Xue & R.M.K.Saunders*	Monoon	Annonaceae	1.121	4.312	Simple, Narrow
10	*Alstonia scholaris (L.) R. Br.*	Alstonia	Apocynaceae	1.099	3.701	Simple
11	*Calotropis gigantea (L.) W.T.Aiton*	Calotropis	Apocynaceae	1.044	2.636	Simple
12	*Plumeria rubra L.*	Plumeria	Apocynaceae	1.114	4.000	Simple, Narrow
13	*Tabernaemontana alternifolia L.*	Tabernaemontana	Apocynaceae	1.103	3.821	Simple
14	*Nerium oleander L.*	Nerium	Apocynaceae	1.119	4.652	Simple, Narrow

15	*Rhaphidophora tetrasperma Hook.f.*	Rhaphidophora	Araceae	1.081	3.972	Simple, Lobed
16	*Tagetes erecta L.*	Tagetes	Asteraceae	1.408	8.911	Unipinnate
17	*Tithonia diversifolia (Hemsl.) A.Gray*	Tithonia	Asteraceae	1.108	3.903	Simple, Lobed
18	*Handroanthus impetiginosus (Mart. ex DC.) Mattos*	Handroanthus	Bignoniaceae	1.100	3.594	Simple
19	*Jacaranda mimosifolia D.Don*	Jacaranda	Bignoniaceae	1.952	30.683	Bipinnate
20	*Tecoma stans (L.) Juss. ex Kunth*	Tecoma	Bignoniaceae	1.114	3.685	Simple, Toothed
21	*Trema tomentosa (Roxb.) H. Hara*	Trema	Cannabaceae	1.122	4.171	Simple, Toothed
22	*Carica papaya L.*	Carica	Caricaceae	1.135	4.44	Simple, Lobed
23	*Garcinia mangostana L.*	Garcinia	Clusiaceae	1.103	3.578	Simple
24	*Terminalia arjuna (Roxb. ex DC.) Wight & Arn.*	Terminalia	Combretaceae	1.082	3.619	Simple
25	*Terminalia bellirica (Gaertn.) Roxb.*	Terminalia	Combretaceae	1.068	3.044	Simple
26	*Merremia vitifolia (Burm. f.) Hallier f.*	Merremia	Convolvulaceae	1.077	3.177	Simple, Lobed
27	*Hopea ponga (Dennst.) Mabb.*	Hopea	Dipterocarpaceae	1.098	3.452	Simple
28	*Vateria indica L.*	Vateria	Dipterocarpaceae	1.120	3.437	Simple, Narrow
29	*Hevea brasiliensis (Willd. ex A.Juss.) Müll.Arg.*	Hevea	Euphorbiaceae	1.058	3.242	Simple

30	*Macaranga peltata (Roxb.) Müll.Arg.*	Macaranga	Euphorbiaceae	1.071	3.066	Simple
31	*Acacia auriculiformis Benth.*	Acacia	Fabaceae	1.127	4.208	Simple, Narrow
32	*Acacia mangium Willd.*	Acacia	Fabaceae	1.089	3.717	Simple
33	*Albizia odoratissima (L.f.) Benth.*	Albizia	Fabaceae	1.226	10.14	Bipinnate
34	*Bauhinia purpurea L.*	Bauhinia	Fabaceae	1.048	2.719	Simple, Lobed
35	*Caesalpinia sappan L.*	Caesalpinia	Fabaceae	1.173	9.299	Bipinnate
36	*Caesalpinia pulcherrima (L.) Sw.*	Caesalpinia	Fabaceae	1.16	8.54	Bipinnate
37	*Calliandra haematocephala Hassk.*	Calliandra	Fabaceae	1.233	6.356	Unipinnate
38	*Cassia fistula L.*	Cassia	Fabaceae	1.233	6.158	Unipinnate
39	*Centrosema molle Benth.*	Centrosema	Fabaceae	1.116	4.004	Trifoliate
40	*Gliricidia sepium (Jacq.) Kunth*	Gliricidia	Fabaceae	1.15	5.863	Unipinnate
41	*Indigofera hirsuta L.*	Indigofera	Fabaceae	1.206	5.574	Unipinnate
42	*Millettia pinnata (L.) Panigrahi*	Millettia	Fabaceae	1.086	3.456	Simple
43	*Senna occidentalis L.*	Senna	Fabaceae	1.215	6.333	Unipinnate
44	*Senna siamea (Lam.) H.S.Irwin & Barneby*	Senna	Fabaceae	1.186	6.896	Unipinnate
45	*Senna surattensis (Burm.f.) H.S.Irwin & Barneby*	Senna	Fabaceae	1.197	5.192	Unipinnate
46	*Sesbania grandiflora (L.) Pers.*	Sesbania	Fabaceae	1.227	6.979	Unipinnate
47	*Tamarindus indica L.*	Tamarindus	Fabaceae	1.125	5.328	Unipinnate

48	*Flacourtia indica (Burm.fil.) Merr.*	Salicaceae	Flacourtia	1.056	2.786	Simple, Toothed
49	*Holmskioldia sanguinea Retz.*	Holmskioldia	Lamiaceae	1.077	3.218	Simple, Toothed
50	*Vitex negundo L.*	Vitex	Lamiaceae	1.211	6.034	Palmate, 3-5 foliolate
51	*Cinnamomum tamala (Buch.-Ham.) T.Nees & Eberm.*	Cinnamomum	Lauraceae	1.098	3.515	Simple
52	*Cinnamomum verum J.Presl*	Cinnamomum	Lauraceae	1.055	2.852	Simple
53	*Strychnos nux-vomica L.*	Strychnos	Loganiaceae	1.08	3.1	Simple
54	*Dendrophthoe falcata (L.fil.) Blume*	Dendrophthoe	Loranthaceae	1.092	3.874	Simple
55	*Lagerstroemia speciosa (L.) Pers.*	Lagerstroemia	Lythraceae	1.051	2.734	Simple
56	*Lawsonia inermis L.*	Lawsonia	Lythraceae	1.068	2.890	Simple
57	*Durio zibethinus Murray*	Durio	Malvaceae	1.102	3.528	Simple
58	*Hibiscus cannabinus L.*	Hibiscus	Malvaceae	1.204	5.591	Simple, Lobed
59	*Hibiscus rosa-sinensis L.*	Hibiscus	Malvaceae	1.112	3.825	Simple, Toothed
60	*Hibiscus tiliaceus L.*	Hibiscus	Malvaceae	1.082	2.968	Simple
61	*Sterculia balanghas L.*	Sterculia	Malvaceae	1.079	3.148	Simple
62	*Thespesia populnea (L.) Sol. ex Corrêa*	Thespesia	Malvaceae	1.102	3.408	Simple
63	*Clidemia hirta (L.) D. Don*	Clidemia	Melastomataceae	1.059	2.716	Simple, Toothed
64	*Azadirachta indica A.Juss.*	Azadirachta	Meliaceae	1.23	7.642	Unipinnate
65	*Melia azedarach L.*	Melia	Meliaceae	1.352	10.292	Bipinnate
66	*Artocarpus heterophyllus Lam.*	Artocarpus	Moraceae	1.076	2.964	Simple

67	*Artocarpus hirsutus Lam.*	Artocarpus	Moraceae	1.049	2.686	Simple
68	*Ficus benghalensis L.*	Ficus	Moraceae	1.053	2.652	Simple
69	*Ficus elastica Roxb.*	Ficus	Moraceae	1.057	2.779	Simple
70	*Ficus exasperata Vahl*	Ficus	Moraceae	1.088	3.41	Simple
71	*Ficus hispida L.fil.*	Ficus	Moraceae	1.080	3.201	Simple
72	*Ficus religiosa L.*	Ficus	Moraceae	1.125	3.842	Simple
73	*Morus alba L.*	Morus	Moraceae	1.051	2.722	Simple, Toothed
74	*Morus macroura Miq.*	Morus	Moraceae	1.069	3.113	Simple, Toothed
75	*Moringa oleifera Lam.*	Moringa	Moringaceae	1.384	10.954	Tripinnate
76	*Eugenia victoriana Cuatrec.*	Eugenia	Myrtaceae	1.100	3.615	Simple
77	*Pimenta dioica (L.) Merr.*	Pimenta	Myrtaceae	1.066	2.854	Simple
78	*Psidium cattleianum Afzel. ex Sabine*	Psidium	Myrtaceae	1.060	2.987	Simple
79	*Psidium guajava L.*	Psidium	Myrtaceae	1.074	3.131	Simple
80	*Syzygium aqueum (Burm.fil.) Alston*	Syzygium	Myrtaceae	1.063	2.716	Simple
81	*Syzygium aromaticum (L.) Merr. & Perry*	Syzygium	Myrtaceae	1.090	3.545	Simple
82	*Syzygium cumini (L.) Skeels*	Syzygium	Myrtaceae	1.078	3.045	Simple
83	*Syzygium jambos (L.) Alston*	Syzygium	Myrtaceae	1.144	4.522	Simple, Narrow
84	*Syzygium malaccense (L.) Merr. & L.M.Perry*	Syzygium	Myrtaceae	1.092	3.511	Simple
85	*Syzygium samarangense (Blume) Merr. & L.M.Perry*	Syzygium	Myrtaceae	1.071	2.992	Simple

86	*Nyctanthes arbor-tristis L.*	Nyctanthes	Oleaceae	1.057	3.064	Simple
87	*Averrhoa bilimbi L.*	Averrhoa	Oxalidaceae	1.403	8.395	Unipinnate
88	*Averrhoa carambola L.*	Averrhoa	Oxalidaceae	1.117	4.812	Unipinnate
89	*Bridelia retusa (L.) A.Juss.*	Bridelia	Phyllanthaceae	1.051	2.581	Simple
90	*Phyllanthus acidus (L.) Skeels*	Phyllanthus	Phyllanthaceae	1.19	6.188	Unipinnate
91	*Phyllanthus emblica L.*	Phyllanthus	Phyllanthaceae	1.579	13.862	Unipinnate
92	*Sauropus androgynus (L.) Merr.*	Sauropus	Phyllanthaceae	1.135	5.832	Unipinnate
93	*Piper longum L.*	Piper	Piperaceae	1.076	3.279	Simple
94	*Piper nigrum L.*	Piper	Piperaceae	1.064	2.833	Simple
95	*Xanthophyllum flavescens Roxb.*	Xanthophyllum	Polygalaceae	1.085	3.295	Simple
96	*Carallia brachiata (Lour.) Merr.*	Carallia	Rhizophoraceae	1.062	3.174	Simple
97	*Mussaenda philippica A.Rich.*	Mussaenda	Rubiaceae	1.087	3.283	Simple
98	*Aegle marmelos (L.) Corrêa*	Aegle	Rutaceae	1.21	5.976	Compound, 3-5 foliolate
99	*Murraya koenigii (L.) Spreng.*	Murraya	Rutaceae	1.245	7.787	Unipinnate
100	*Santalum album L.*	Santalum	Santalaceae	1.091	3.217	Simple
101	*Nephelium lappaceum L.*	Nephelium	Sapindaceae	1.106	2.806	Simple
102	*Nephelium mutabile Blume*	Nephelium	Sapindaceae	1.086	3.456	Simple
103	*Chrysophyllum cainito L.*	Chrysophyllum	Sapotaceae	1.075	3.105	Simple
104	*Chrysophyllum oliviforme L.*	Chrysophyllum	Sapotaceae	1.057	2.726	Simple
105	*Manilkara zapota (L.) P.Royen*	Manilkara	Sapotaceae	1.122	3.882	Simple
106	*Mimusops elengi L.*	Mimusops	Sapotaceae	1.101	3.521	Simple
107	*Pouteria caimito (Ruiz & Pav.) Radlk.*	Pouteria	Sapotaceae	1.080	3.426	Simple

108	*Synsepalum dulcificum (Schumach. & Thonn.) Daniell*	Synsepalum	Sapotaceae	1.102	3.624	Simple
109	*Simarouba glauca DC.*	Simarouba	Simaroubaceae	1.216	6.482	Unipinnate

Leaves with petioles were scanned on a white background using a digital scanner (Epson L 360). The original RGB images were scaled to 1024 × 1024 pixels and converted into bitmap format (24-bit). The scaled colour images were transformed into grayscale images and converted to binary images using Otsu's threshold [45]. The binary images were used to estimate the Geometric entropy.

The perimeter and area of the binary leaf images were computed using MATLAB [46]. The area of the leaf corresponds to the total number of pixels in the leaf part in the binary images. However, the perimeter corresponds to the number of pixels along the periphery of the leaf parts. The geometric entropy (S_L) of the leaves was calculated using Eq (19).

Segmental fractal complexity ($D_{\Sigma S}$) is an improved leaf complexity measure from a fractal-thermodynamic system analogy [47]. It is an algebraic combination of the fractal dimensions of the components of the leaf images, viz., leaf lamina, the background, and leaf edges.

$$D_{\Sigma S} = D_{Background} + D_{Edge} - D_{Leaf} \tag{20}$$

where $D_{Background}$, D_{Edge}, and D_{Leaf} are the fractal dimensions of leaf background, edges, and lamina, respectively. $D_{\Sigma S}$ of the leaves was calculated using Eq (20). S_L was correlated with $D_{\Sigma S}$.

Results

Plant leaves used in this study exhibited remarkable morphological complexity (Fig 4). The Geometric entropy of the leaf images *(S_L)* varied between 2.581 and 30.683 (Table 1). The leaf of *Bridelia retusa* showed the lowest S_L, and that of *Jacaranda mimosifolia*, the highest. Deeply lobed broad leaves of *Bauhinia purpurea* (2.719) and *Merremia vitifolia* (3.177) showed lower S_L than many simple leaves in the
This data is already in the table
study. Narrow leaves of *Plumeria rubra* (3.999), *Acacia auriculiformis* (4.208), *Mangifera indica* (4.262), *Monoon longifolium* (4.312), *Syzygium jambos* (4.522), and *Nerium oleander* (4.652) showed higher S_L

than deeply lobed leaves of *Tithonia diversifolia* (3.903), *Rhaphidophora tetrasperma* (3.972). The S_L of pinnately compound leaves of *Cassia fistula*, *Phyllanthus acidus*, *Senna occidentalis*, *Calliandra haematocephala*, *Simarouba glauca*, *Senna siamea*, *Sesbania grandiflora*, *Azadirachta indica*, *Murraya koenigii*, *Averrhoa bilimbi*, *Caesalpinia pulcherrima*, *Tagetes erecta*, *Caesalpinia Sappan*, *Albizia odoratissima*, *Melia azedarach*, *Moringa oleifera*, *Phyllanthus emblica*, and *Jacaranda mimosifolia* were the highest and ranged from 6.158 to 30.683 (Table 1). The S_L values increase with decreasing leaf width and increasing leaf pinnation. S_L was comparable for leaves with similar morphology.

Fig 4. Morphological complexity of the plant leaves studied. Arrangements of the leaves were in the increasing order of geometric entropy (S_L).

Table 1 presents the segmental fractal complexity ($D_{\Sigma S}$) of the leaves studied. $D_{\Sigma S}$ varies between 1.044 and 1.952. The pinnately compound leaf of *Jacaranda mimosifolia* recorded the highest $D_{\Sigma S.}$ The lowest $D_{\Sigma S}$ was observed for the simple leaf of *Calotropis gigantea*. The $D_{\Sigma S}$ of the pinnately compound leaves of *Senna occidentalis*, *Simarouba glauca*, *Albizia odoratissima*, *Sesbania grandiflora*, *Azadirachta indica*, *Calliandra haematocephala*, *Cassia fistula*, *Murraya koenigii*, *Melia azedarach*, *Moringa oleifera*, *Averrhoa bilimbi*, *Tagetes erecta*, *Phyllanthus emblica*, *Jacaranda mimosifolia* ranged between 1.215 and 1.952 (Table 1). $D_{\Sigma S}$ also increases with decreasing leaf width and increasing leaf lobiness and pinnation. Figure 5 illustrates a strong monotonic linear relationship (ρ = 0.95) between S_L and $D_{\Sigma S}$.

Fig 5. Monotonic relationship between the Geometric Entropy (S_L) and Segmental Fractal Complexity ($D_{\Sigma S}$).

We classified the variation in S_L at the taxonomic level. Figure 6 depicts the similarity dendrogram. The high cophenetic correlation coefficient (0.97) indicates the quality of classification. The leaves of 109 species were grouped into 5 clusters (indicated with different colours in Fig 6) at 90% similarity threshold. Three-quarters of the species studied were clubbed as cluster 1 (red in Fig 6). Except for *Averrhoa carambola*, all other species in cluster 1 had simple leaves. The similarity of this cluster ranges from 96 –

100%. S_L in cluster 1 varies between only 2.581 and 4.812. However, the values increase with decreasing leaf width. Narrow simple leaves exhibited comparatively a higher S_L and were clubbed together in cluster 1. Read in an anticlockwise direction; narrow leaves predominantly occupied the tail end of cluster 1 (Fig 6). The following families included multiple species and were unique to cluster 1: Anacardiaceae (3 species), Annonaceae (4 species), Apocynaceae (4 species), Combretaceae (2 species), Dipterocarpaceae (2 species), Euphorbiaceae (2 species), Lauraceae (2 species), Lythraceae (2 species), Moraceae (9 species), Myrtaceae (10 species), Piperaceae (2 species), Sapindaceae (2 species), and Sapotaceae (6 species). Different species from the same genus of the above-listed families showed remarkable similarity in shape in this cluster (Table 2).

Fig 6. Dendrogram of geometric entropy of plant leaves (S_L). The dendrogram results in 5 clusters at a cutoff similarity of 90%.

Table 2. Distribution of geometric entropy (S_L) plant species across different generic clusters.

Family	Genera	Plant Species		
		**Cluster 1**	**Cluster 2**	**Cluster 3**
Annonaceae	Annona	*Annona muricata L.*		
		Annona squamosa L.		
		Annona reticulata L.		
Combretaceae	Terminalia	*Terminalia bellirica (Gaertn.) Roxb.*		
		Terminalia arjuna (Roxb. ex DC.) Wight & Arn.		
Fabaceae	Acacia	*Acacia mangium Willd.*		
		Acacia auriculiformis Benth.		
	Senna		*Senna surattensis (Burm.f.) H.S.Irwin & Barneby*	
			Senna occidentalis L.	
			Senna siamea (Lam.) H.S.Irwin & Barneby	
	Caesalpinia			*Caesalpinia pulcherrima (L.) Sw.*
				Caesalpinia sappan L.
Lauraceae	Cinnamomum	*Cinnamomum verum J.Presl*		
		Cinnamomum tamala (Buch.-Ham.) T.Nees & Eberm.		
Malvaceae	Hibiscus	*Hibiscus tiliaceus L.*		

		Hibiscus rosa-sinensis L.		
Moraceae	Ficus	*Ficus benghalensis L.*		
		Ficus elastica Roxb.		
		Ficus hispida L.fil.		
		Ficus exasperata Vahl		
		Ficus religiosa L.		
	Morus	*Morus alba L.*		
		Morus macroura Miq.		
Myrtaceae	Syzygium	*Syzygium aqueum (Burm.fil.) Alston*		
		Syzygium samarangense (Blume) Merr. & L.M.Perry		
		Syzygium cumini (L.) Skeels		
		Syzygium malaccense (L.) Merr. & L.M.Perry		
		Syzygium aromaticum (L.) Merr. & Perry		
		Syzygium jambos (L.) Alston		
	Psidium	*Psidium cattleianum Afzel. ex Sabine*		
		Psidium guajava L.		
Piperaceae	Piper	*Piper nigrum L.*		
		Piper longum L.		
Sapindaceae	Nephelium	*Nephelium lappaceum L.*		
		Nephelium mutabile Blume		
Sapotaceae	Chrysophyllum	*Chrysophyllum oliviforme L.*		

		Chrysophyllum cainito L.		

Clusters 4 and 5 are *simplicifolious* with *Phyllanthus emblica* and *Jacaranda mimosifolia*, respectively. Plant species from the Fabaceae family exhibit diverse leaf shapes across different genera and are thus included in multiple clusters.

Cluster 2 (blue in Fig 6) comprises predominantly uni-pinnate species. It does not include any simple or bi or tri-pinnate leaves. The similarity of this cluster ranges between 94 - 100%. Of these *Senna surattensis*, *Tamarindus indica*, *Indigofera hirsute*, *Gliricidia sepium*, *Cassia fistula*, *Senna occidentalis*, *Calliandra haematocephala*, *Senna siamea*, *Sesbania grandiflora* were from Fabaceae family. S_L of leaves of species from the Fabaceae family varies between 2.719 (*Bauhinia purpurea*) and 10.14 (*Albizia odoratissima*). Leaves of species under the Fabaceae family exhibit diverse shapes ranging from simple to bipinnate. The former was categorised into cluster 1 and the latter into cluster 3. Three species from the genus: Senna, *Senna surattensis* (S_L - 5.192), *Senna occidentalis* (S_L - 6.333), *Senna siamea* (S_L - 6.896) were grouped in cluster 2 (Table 2).

Cluster 3 (green in Fig 6) comprises only pinnately compound leaves, and their S_L varies between 8.395 and 10.954. The similarity of this cluster ranges from 93% to 100%. Of these *Averrhoa bilimbi*, *Tagetes erecta* were uni-pinnate, and *Caesalpinia pulcherrima*, *Caesalpinia Sappan*, *Albizia odoratissima*, and *Melia azedarach*, were bi-pinnate. However, the leaf of *Moringa oleifera* (S_L - 10.954) is tripinnate and is the distant *'leaf'*. Two species from the same genus: Caesalpinia (Fabaceae), *Caesalpinia pulcherrima* (S_L - 8.540), and *Caesalpinia Sappan (*S_L - 9.299), were also grouped in cluster 3 (Table 2).

Cluster 4 and 5 (magenta and yellow in Fig 6) constitutes only one clade each, *Phyllanthus emblica* (Phyllanthaceae, S_L - 13.862), and *Jacaranda mimosifolia* (Bignoniaceae, S_L - 30.683), respectively, and stand out as *simplicifolious* (dissimilarity 83% and 100% respectively). S_L of species' leaves from Phyllanthaceae varies from 2.581 (*Bridelia retusa*) to 13.862 (*Phyllanthus emblica*). Their shapes range from simple (*Bridelia retusa*) to uni-pinnate (*Sauropus androgynus*, *Phyllanthus acidus*, *Phyllanthus emblica*). Apart from cluster 4, they also belong to cluster 2. Similarly, *Handroanthus impetiginosus (*S_L - 3.594), *Tecoma stans* (S_L - 3.685) from Bignoniaceae, were also included in cluster 1. S_L of species from Bignoniaceae varies from 3.594 (*Handroanthus impetiginosus*) to 30.683 (*Jacaranda mimosifolia*). While the present study does not reveal well-differentiated clusters to discriminate S_L at the family level, it does reveal the well-differentiated clustering of S_L at the generic level (Table 2).

Discussion

Geometric shape (information) plays a vital role in ecological systems [48]. The morphological structure of life forms carries information imparted by its geometry to distinguish them from others. However, the geometry of every living object is bestowed by the boundary that limits their biomass. Geometric analysis of natural structures allows for determining their shape optimisation to maximise energy efficiency and visual appeal [49].

Defining boundaries is vital in morphometric analysis. In current knowledge, living forms are confined only to three dimensions. Boundaries distinguishing organisms (or any objects) from their environment (or non-objects) also have characteristic dimensions. One-dimensional objects are lines confined by a boundary consisting of two points (dimensionless). 2-D objects are planar and confined by a boundary which is a line (1-D). Similarly, 3-D objects are spatial and confined by a boundary which is an area (2-D). In general, an n-dimensional object is confined by an n-1 dimensional boundary.

We describe the geometry of a circular object (2-D) by the sharp interface property of the Heaviside step function ($H(x)$) (Fig 1). Considering the boundary of the circular object as a diffuse interface with an infinite number of equiprobable states of presence and absence of the object, the geometric entropy (S_{GE}) at the circle's interface is deduced by using a phase-field function (Fig 2). The S_{GE} (in Eq (18)) is composed of only two variables; circumference and the discretisation length. This entropy is associated only with the information contained in the structural form of objects.

Geometric entropy is not limited to circular objects. It can be constructed for objects of any dimension. In 2-D, an arc is part of the circumference of a circle. Since the area element in plane-polar coordinates is simple ($rdrd\theta$), S_{GE} can be determined directly from Temkin's model. Apart from a circle, every 2-D shape can be visualised as made of infinitesimally small arcs. Hence, their geometric entropy can be arrived at from Temkin's model. However, the progression of the area element ($rdrd\theta$) in plane-polar coordinates

complicates the formulation of the entropy of non-circular objects. Notwithstanding the above, the general structure of the geometric entropy of any 2-D shape will be similar to that of a circle (S_{GE}). The parameters (circumference and discretisation length) will remain the same, but the coefficient in the S_{GE} equation is subject to change. Hence the entropy of any 2-D object is comparable to S_{GE}. Here we focus on terrestrial plant leaves viewed as 2-D objects.

S_{GE} was transformed by considering the plant leaf-environment interface as a narrow phase field to describe the geometric entropy of plant leaves (S_L). Two leaf traits, viz. leaf area, and perimeter were selected as the parameters in the relation. They were selected based on their dimensionality and physiological importance.

Plant leaves absorb solar energy and process it to maintain a high organisation with lower entropy by photosynthesis [50]. The capture of energy and carbon assimilation in plant leaves depends on the geometry and positioning of the leaf lamina. An increase in leaf perimeter (by serration or lobes) increases the entropy in shape and geometric complexity. However, a high perimeter achieved by increasing the laminar area may not increase the leaf complexity unless accompanied by a significant increase in the number of serrations or lobes. Leaf perimeter influences its physiology in many ways [51–53]. Increasing the marginal serration without compromising the leaf area could be regarded as an adaptive strategy in terms of heating [54]. Deep serrations or lobes reduce the leaf area and maintain photosynthetic tissues closer to veins, thereby supporting high photosynthetic rates [55,56]. The leaf hydraulic resistance also describes the adaptive stability of dissected leaves. Lobed and dissected leaves have fewer minor veins that reduce hydraulic resistance than entire leaves, which is advantageous in dry environments [55,57]. Further, leaf margin optimisation with air temperature has been confirmed in many areas and utilised in many plant evolutionary studies [58,59].

Leaf energetics have focused more on leaf area than other traits such as leaf length, width, thickness, and perimeter. A larger leaf area is advantageous in light capture and photosynthetic productivity [60]. The seemingly obvious behaviour of small-size leaves in higher canopies [61] and larger leaves in lower

canopies [62,63] provide evidence of the importance of leaf surface area in solar energy absorption and photosynthesis [64]. Notably, smaller leaves with significant vein density are more efficient in nutrient transport and tolerant to leaf vein embolism [65].

The areal optimisation of the leaves also influences the leaf thermal regulation via boundary layer thickness [66,67]. Larger leaves have a thick leaf boundary layer that diminishes the convective heat loss and gas exchange between the leaf and the surrounding air compared to smaller leaves with a thin boundary layer [68]. Further, the decrease in leaf size with decreasing water availability [69] reduces the leaf temperature and avoids overheating in arid environments [70]. Therefore, having small leaves is generally advantageous in arid environments. However, large leaves with less energy exchange efficiency seem advantageous in humid environments [71,72].

Narrowing of leaves is an adaptive strategy to maintain a high perimeter within a finite area. Narrow leaves with optimal width have less photosynthetic productivity in terms of leaf area. However, the reduction in leaf area is compensated by the ability of elongated leaves to harvest water from fog [73] and tolerate strong shearing forces [74]. Leaf width and perimeter (lobiness) also describe the adaptive stability of plants by the boundary layer thickness of plant leaves. Lobed and narrow leaves usually have a thin leaf boundary layer than circular leaves [75,76]. When the microclimatic boundary layer becomes thin, leaves can track the surrounding air through efficient cooling and heating by convection [76,77]. Thus lobed and narrow leaves are less vulnerable to heating and freezing during the day and night [78].

Since the form of geometric entropy of any 2-D object resembles S_{GE}, and here we consider the plant leaf as a 2-D object (see above), the parameter perimeter was taken as the leaf perimeter (P), and the smallest discretisation length as the square root of the leaf area (A). S_{GE} described here is a straightforward formula that evolved from an information perspective. S_L increases with P and decreases with A. The leaf area in the denominator of S_L offsets the uncertainty of a large perimeter with entire leaf margins. It reveals the same structure as *LDI*. Leaves with a higher P/A ratio have higher geometric entropy and complexity,

ensuring more adaptive stability in changing environments (see above). Consequently, leaf geometry converges into fractal-like structures to accommodate excess leaf margin (within a finite area) by inducing waviness and lobiness along the edges. It is logical as fractal structures arise as a natural consequence of the most effective energy dissipation requirements [79,80]. Natural fractals are an innovative adaptive strategy that minimises energy/nutrient loss. Therefore, increasing geometric entropy opens discussions on the direct causal links between leaf stability/efficiency and complexity.

Leaves with similar morphological features have comparable S_L values (Table 1). Narrow and pinnately compound leaves had a higher *P/A* ratio and S_L values. Consequently, narrow-simple leaves used in the study recorded higher S_L than broad leaves with deep lobes. Irrespective of the size of the leaf laminar plane, S_L is scalable by the *P/A* ratio. Similar to the S_L values, $D_{\Sigma S}$ increases with decreasing leaf width and increasing lobiness and pinnation.

Though S_L *and* $D_{\Sigma S}$ were developed from two different notions, they are related. The strong linear relation between the S_L and $D_{\Sigma S}$ (Fig 5) reinforces the validity of S_L. S_L is developed from an information theory approach. It increases with increasing leaf perimeter and decreasing leaf area. Leaves with lobiness, dissection, narrow width, and high serrations have a high *P/A* ratio and, thereby, high S_L. $D_{\Sigma S}$ is based on a fractal-thermodynamic system analogy that comprises discrete fractal dimensions of leaf parts: lamina, background, and edge. The principle of fractal analysis stems from the space-filling capacity of fractal parts (leaves), which describes the scaling and distribution of leaf parts from the surrounding space. $D_{\Sigma S}$ is lower for simple leaves and increases with thinning, lobiness, and pinnation of leaves. The ability to capture the plant leaves' dissection, lobiness, and serration features underlines the analogy between S_L and $D_{\Sigma S}$.

Univariate cluster analysis of S_L distribution reveals the taxonomic relationship among the leaf shapes (Fig 6, Table 2). S_L was classified into 5 clusters at a threshold similarity of 90%. Three-quarters of the species were clubbed into cluster 1 (red in Fig 6), and most had simple leaves (Fig 4). Simple leaves generally have comparable length and width as compared to compound leaves. In such cases, the variations in S_L due to

the variations in perimeter and area (due to variations in length and width) will be less apparent in simple leaves than in compound leaves. This limits the S_L of leaves in cluster 1 to be grouped together. Leaves in other clusters show significant S_L variations (5.192 - 30.683). A consistent pattern of S_L could not be attributed to plant leaves belonging to the same family. The leaves of plants from the same family exhibit various leaf shapes. However, a pattern seems to emerge at the generic level. Plant leaves of the species belonging to the same genera exhibit similar shapes and S_L values.

S_L is an inherent complexity measure that outperforms other complex geometric morphometrics. Devoid of statistical techniques, S_L is free from time-consuming preprocessing techniques and can account for leaf shape regardless of the size of the leaves. It posits a prospective method to quantify the extent of variation in leaf shapes, especially by the influence of deep lobiness, serrations, and dissections on leaf perimeter. Moreover, the theoretical maximum of $D_{\Sigma S}$ of extreme narrow leaves and the Peano curve-shaped leaves with a high *P/A* ratio [47] consolidates S_L. Since complex leaves (high *P/A* ratio) have more adaptive stability in changing environments [81,82], S_L can be considered a derived plant trait to describe leaf complexity and adaptive stability.

The evolution of leaf shapes is not by chance. It is a side-product of functional perfection [83]. The knowledge of the shape and size of leaf forms has a vast potential for understanding geometric information and its link with energy capture. The joint descriptions of information and energy will answer pertinent questions: How and why does nature use such a design procedure? How are radiation absorption and entropy emission processing effective? Our computation of S_L is based on an informational description of a shape descriptive function. Integrating shape-related approaches of S_L into other leaf physiological measures such as photosynthesis, respiration, and evaporation can open pathways to understanding energy flow and interaction between plant leaves and their surroundings. Resolving these gaps between information, entropy, and energy will allow advances to reveal the dynamics of information exchange in ecological systems.

Conclusion

We presented a mathematical framework to estimate the geometric entropy of plant leaves. The geometric entropy contains information on leaf geometry, represented by two physiologically important traits, leaf perimeter and leaf area. The geometric entropy of the leaf reveals the physical basis of its dissection index and accounts for the connection between complexity and adaptive stability in plants. However, leaves exhibit diverse leaf shapes at the family level. That limits S_L to any taxonomic relationship at the family level. A consistent pattern of S_L seems to emerge at the generic level. Further, we propose geometric entropy as a derived plant trait to discriminate leaf shapes and denote the adaptive stability of plants in rapidly changing environments. The relevance of this morphological trait needs to be tested to explore adaptive plant morphogenesis and obtain a clearer picture of function-based Eco-Devo studies.

Acknowledgments

We thank Dr. Saji Gopinath, Vice-Chancellor, Kerala University of Digital Sciences, Innovation and Technology, India, for providing all necessary support to carry out the study. We further gratefully acknowledge the support of Mr. Aiswaria G R, Kerala University of Digital Sciences, Innovation, and Technology, for valuable comments that substantially improved the manuscript.

References

1. Bell AD, Bryan A. Plant Form: An Illustrated Guide to Flowering Plant Morphology. Timber Press; 2008.

2. Thyagharajan KK, Kiruba Raji I. A Review of Visual Descriptors and Classification Techniques Used in Leaf Species Identification. Arch Comput Methods Eng. 2019;26: 933–960. doi:10.1007/s11831-018-9266-3

3. Posamentier AS, Lehmann I. The Fabulous Fibonacci Numbers. Amherst, NY: Prometheus Books; 2007.

4. Abbas S. Golden ratio: A measure of physical beauty. Resonance. 2017;22: 51–60. doi:10.1007/s12045-017-0432-y

5. Pickover CA. The Pattern Book: Fractals, Art and Nature. World Scientific Publishing Company; 1995.

6. Baverstock K, Rönkkö M. The evolutionary origin of form and function. J Physiol. 2014;592: 2261–2265. doi:10.1113/jphysiol.2014.271775

7. Barnes AD, Jochum M, Lefcheck JS, Eisenhauer N, Scherber C, O’Connor MI, et al. Energy Flux: The Link between Multitrophic Biodiversity and Ecosystem Functioning. Trends Ecol Evol. 2018;33: 186–197. doi:10.1016/j.tree.2017.12.007

8. Smith B. Mereotopology: A theory of parts and boundaries. Data Knowl Eng. 1996;20. doi:10.1016/S0169-023X(96)00015-8

9. Garcke H, Nestler B, Stoth B. Multiphase field concept: Numerical simulations of moving phase boundaries and multiple junctions. SIAM J Appl Math. 1999;60. doi:10.1137/S0036139998334895

10. Schmitz GJ. A Phase-Field Perspective on Mereotopology. AppliedMath. 2022;2: 54–103. doi:10.3390/appliedmath2010004

11. Schmitz GJ. Thermodynamics of Diffuse Interfaces. In: Emmerich H, Nestler B, Schreckenberg M, editors. Interface and Transport Dynamics, Computational Modelling, Lecture Notes in Computational Science and Engineering. Springer Berlin, Heidelberg; 2003. pp. 47–64. doi:https://doi.org/10.1007/978-3-662-07969-0

12. Kobayashi R. Modeling and numerical simulations of dendritic crystal growth. Phys D Nonlinear Phenom. 1993;63: 410–423. doi:10.1016/0167-2789(93)90120-P

13. Steinbach I, Pezzolla F, Nestler B, Seeßelberg M, Prieler R, Schmitz GJ, et al. A phase field concept for multiphase systems. Phys D Nonlinear Phenom. 1996;94. doi:10.1016/0167-2789(95)00298-7

14. Böttger B, Apel M, Budnitzki M, Eiken J, Laschet G, Zhou B. Calphad coupled phase-field model with mechano-chemical contributions and its application to rafting of γ' in CMSX-4. Comput Mater Sci. 2020;184: 109909. doi:10.1016/j.commatsci.2020.109909

15. Markosian N. What are Physical Objects? Philos Phenomenol Res. 2000;61: 375. doi:10.2307/2653656

16. Sekerka RF. Morphology: From sharp interface to phase field models. J Cryst Growth. 2004;264: 530–540. doi:10.1016/j.jcrysgro.2003.12.033

17. Campelo F, Hernández-Machado A. Shape instabilities in vesicles: A phase-field model. Eur Phys J Spec Top. 2007;143: 101–108. doi:10.1140/epjst/e2007-00077-y

18. Ansari TQ, Huang H, Shi S-Q. Phase field modeling for the morphological and microstructural evolution of metallic materials under environmental attack. npj Comput Mater. 2021;7: 143. doi:10.1038/s41524-021-00612-7

19. Johnson DM, Smith WK, Vogelmann TC, Brodersen CR. Leaf architecture and direction of incident light influence mesophyll fluorescence profiles. Am J Bot. 2005;92: 1425–1431. doi:10.3732/ajb.92.9.1425

20. Crofts AR. Life, information, entropy, and time: Vehicles for semantic inheritance. Complexity. 2007;13: 14–50. doi:10.1002/cplx.20180

21. Jost J. Biology, geometry and information. Theory Biosci. 2022;141: 65–71. doi:10.1007/s12064-021-00351-9

22. Schmitz GJ. Entropy and geometric objects. Entropy. 2018;20. doi:10.3390/e20060453

23. Altieri L, Cocchi D, Roli G. Advances in spatial entropy measures. Stoch Environ Res Risk Assess. 2019;33. doi:10.1007/s00477-019-01686-y

24. Onoda Y, Westoby M, Adler PB, Choong AMF, Clissold FJ, Cornelissen JHC, et al. Global patterns of leaf mechanical properties. Ecol Lett. 2011;14: 301–312. doi:10.1111/j.1461-0248.2010.01582.x

25. Boettinger WJ, Warren JA, Beckermann C, Karma A. Phase-Field Simulation of Solidification. Annu Rev Mater Res. 2002;32: 163–194. doi:10.1146/annurev.matsci.32.101901.155803

26. Zhang W, Zhou Y. Level-set functions and parametric functions. 1st ed. The Feature-Driven Method for Structural Optimization. 1st ed. Elsevier; 2020.

27. Steinbach I. Phase-field models in materials science. Model Simul Mater Sci Eng. 2009;17. doi:10.1088/0965-0393/17/7/073001

28. Liu S, Qiao H. Topology optimization of continuum structures with different tensile and compressive properties in bridge layout design. Struct Multidiscip Optim. 2011;43: 369–380. doi:10.1007/s00158-010-0567-x

29. Arfken GB, Weber HJ, Harris FE. Mathematical Methods for Physicists. 7th ed. Academic Press;

2012.

30. Yang W, Li W, Cao Y, Luo Y, He L. Real-time production and logistics self-adaption scheduling based on information entropy theory. Sensors (Switzerland). 2020;20: 1–17. doi:10.3390/s20164507

31. Ben-Gal I, Kagan E. Information Theory: Deep Ideas, Wide Perspectives, and Various Applications. Entropy. 2021;23: 232. doi:10.3390/e23020232

32. Tan Z, Camati PA, Cauquil GC, Auffèves A, Dotsenko I. Alternative experimental ways to access entropy production. Phys Rev Res. 2021;3. doi:10.1103/PhysRevResearch.3.043076

33. Ding LJ, Zhong Y. Critical Scaling of Entropy and Thermal Drude Weight in Anisotropic Heisenberg Antiferromagnets: A Thermodynamic Quest for Quantum Criticality. Front Phys. 2022;10. doi:10.3389/fphy.2022.843627

34. Wimmer JLE, Xavier JC, Vieira A d. N, Pereira DPH, Leidner J, Sousa FL, et al. Energy at Origins: Favorable Thermodynamics of Biosynthetic Reactions in the Last Universal Common Ancestor (LUCA). Front Microbiol. 2021;12. doi:10.3389/fmicb.2021.793664

35. Vanchurin V, Wolf YI, Koonin E V., Katsnelson MI. Thermodynamics of evolution and the origin of life. Proc Natl Acad Sci U S A. 2022;119. doi:10.1073/pnas.2120042119

36. Saidi WA, Shadid W, Veser G. Optimization of High-Entropy Alloy Catalyst for Ammonia Decomposition and Ammonia Synthesis. J Phys Chem Lett. 2021;12: 5185–5192. doi:10.1021/acs.jpclett.1c01242

37. Sun Y, Dai S. High-entropy materials for catalysis: A new frontier. Sci Adv. 2021;7. doi:10.1126/sciadv.abg1600

38. Rosser JB. Entropy and econophysics. Eur Phys J Spec Top. 2016;225: 3091–3104. doi:10.1140/epjst/e2016-60166-y

39. Yan J, Feng L, Denisov A, Steblyanskaya A, Oosterom JP. Complexity theory for the modern Chinese economy from an information entropy perspective: Modeling of economic efficiency and growth potential. PLoS One. 2020;15. doi:10.1371/journal.pone.0227206

40. Temkin DE. Crystallization processes. In: Sirota NN, Gorskii FK, Varikash VM, editors. New York, NY, USA: Consultants Bureau; 1966.

41. Daryl L. Logan. A First Course in the Finite Element Method. 6th ed. Stamford: Cengage Learning; 2016.

42. Araus JL, Alegre L, Tapia L, Calafell R, Serret MD. Relationships between Photosynthetic Capacity and Leaf Structure in Several Shade Plants. Am J Bot. 1986;73: 1760. doi:10.2307/2444243

43. Zheng Y, Jiang W, Silva EN, Mao L, Hannaway DB, Lu H. Optimization of shade condition and harvest time for Dendrobium candidum plants based on leaf gas exchange, alkaloids and polysaccharides contents. Plant Omics. 2012;5: 253–260.

44. Kincaid DT, Schneider RB. Quantification of leaf shape with a microcomputer and Fourier transform. Can J Bot. 1983;61: 2333–2342. doi:10.1139/b83-256

45. Otsu N. Threshold Selection Method from Gray-Level Histograms. IEEE Trans Syst Man Cybern. 1979;9: 62–66. doi:10.1109/tsmc.1979.4310076

46. Benjamin B. Leaf Size (Area) and Shape Code. [cited 14 Sep 2020]. Available: http://www.benjaminblonder.org/leafarea

47. Vishnu M, Jaishanker R. Fractal-Thermodynamic System Analogy and Complexity of Plant Leaves. bioRxiv. 2022; 2022.07.05.498782. doi:10.1101/2022.07.05.498782

48. Sutton JE. What is geometric information and how do animals use it? Behav Processes. 2009;80: 339–343. doi:10.1016/j.beproc.2008.11.007

49. Pérez García AJ, Gómez Martínez F. Natural structures: strategies for geometric and morphological optimization. In: DOMINGO A, LAZARO C, editors. Symposium of the International Association for Shell and Spatial Structures (50th 2009 Valencia) Evolution and Trends in Design, Analysis and Construction of Shell and Spatial Structures: Proceedings. Universidad Politecnica de Valencia, Spain; 2009.

50. Erwin Schrodinger. What is Life? Cambridge, UK: Cambridge University Press; 1992.

51. Lewis MC. The Physiological Significance of Variation in Leaf Structure. Sci Prog (1933-). 1972;60: 25–51. Available: http://www.jstor.org/stable/43420122

52. Gurevitch J, Schuepp PH. Boundary layer properties of highly dissected leaves: an investigation using an electrochemical fluid tunnel. Plant Cell Environ. 1990;13: 783–792. doi:10.1111/j.1365-3040.1990.tb01094.x

53. Royer DL, Wilf P. Why do toothed leaves correlate with cold climates? Gas exchange at leaf margins provides new insights into a classic paleotemperature proxy. Int J Plant Sci. 2006;167: 11–18. doi:10.1086/497995

54. Givnish TJ, Vermeij GJ. Sizes and Shapes of Liane Leaves. Am Nat. 1976;110: 743–778. doi:10.1086/283101

55. Brodribb TJ, Feild TS, Sack L. Viewing leaf structure and evolution from a hydraulic perspective. Funct Plant Biol. 2010;37: 488. doi:10.1071/FP10010

56. Chitwood DH, Sinha NR. Evolutionary and Environmental Forces Sculpting Leaf Development. Curr Biol. 2016;26: R297–R306. doi:10.1016/j.cub.2016.02.033

57. Nicotra AB, Leigh A, Boyce CK, Jones CS, Niklas KJ, Royer DL, et al. The evolution and functional significance of leaf shape in the angiosperms. Funct Plant Biol. 2011;38: 535–552.

doi:10.1071/FP11057

58. Royer DL, Wilf P, Janesko DA, Kowalski EA, Dilcher DL. Correlations of climate and plant ecology to leaf size and shape: Potential proxies for the fossil record. Am J Bot. 2005;92: 1141–1151. doi:10.3732/ajb.92.7.1141

59. Schmerler SB, Clement WL, Beaulieu JM, Chatelet DS, Sack L, Donoghue MJ, et al. Evolution of leaf form correlates with tropical-temperate transitions in Viburnum (Adoxaceae). Proc R Soc B Biol Sci. 2012;279: 3905–3913. doi:10.1098/rspb.2012.1110

60. Koyama K, Kikuzawa K. Is whole-plant photosynthetic rate proportional to leaf area? a test of scalings and a logistic equation by leaf demography census. Am Nat. 2009;173: 640–649. doi:10.1086/597604

61. Jensen KH, Zwieniecki MA. Physical limits to leaf size in tall trees. Phys Rev Lett. 2013;110. doi:10.1103/PhysRevLett.110.018104

62. Reich PB, Wright IJ, Cavender- Bares J, Craine JM, Oleksyn J, Westoby M, et al. The Evolution of Plant Functional Variation: Traits, Spectra, and Strategies. Int J Plant Sci. 2003;164: S143–S164. doi:10.1086/374368

63. Feng Y, Van Kleunen M. Responses to shading of naturalized and non-naturalized exotic woody species. Ann Bot. 2014;114: 981–989. doi:10.1093/aob/mcu163

64. Gommers CMM, Visser EJW, Onge KRS, Voesenek LACJ, Pierik R. Shade tolerance: when growing tall is not an option. Trends Plant Sci. 2013;18: 65–71. doi:10.1016/j.tplants.2012.09.008

65. Scoffoni C, Rawls M, Mckown A, Cochard H, Sack L. Decline of leaf hydraulic conductance with dehydration: Relationship to leaf size and venation architecture. Plant Physiol. 2011;156: 832–843. doi:10.1104/pp.111.173856

66. Niinemets Ü, Portsmuth A, Tobias M. Leaf shape and venation pattern alter the support investments within leaf lamina in temperate species: A neglected source of leaf physiological differentiation? Funct Ecol. 2007;21: 28–40. doi:10.1111/j.1365-2435.2006.01221.x

67. Wright IJ, Dong N, Maire V, Prentice IC, Westoby M, Díaz S, et al. Global climatic drivers of leaf size. Science (80-). 2017;357: 917–921. doi:10.1126/science.aal4760

68. Leigh A, Sevanto S, Close JD, Nicotra AB. The influence of leaf size and shape on leaf thermal dynamics: does theory hold up under natural conditions? Plant Cell Environ. 2017;40: 237–248. doi:10.1111/pce.12857

69. Cramer MD, Hawkins H-J, Verboom GA. The importance of nutritional regulation of plant water flux. Oecologia. 2009;161: 15–24. doi:10.1007/s00442-009-1364-3

70. Niinemets Ü, Kull K. Leaf weight per area and leaf size of 85 Estonian woody species in relation to shade tolerance and light availability. For Ecol Manage. 1994;70: 1–10. doi:10.1016/0378-1127(94)90070-1

71. Gates DM. Biophysical Ecology. New York, NY: Springer New York; 1980. doi:10.1007/978-1-4612-6024-0

72. Ackerly DD, Knight CA, Weiss SB, Barton K, Starmer KP. Leaf size, specific leaf area and microhabitat distribution of chaparral woody plants: Contrasting patterns in species level and community level analyses. Oecologia. 2002;130. doi:10.1007/s004420100805

73. Martorell C, Ezcurra E. The narrow-leaf syndrome: A functional and evolutionary approach to the form of fog-harvesting rosette plants. Oecologia. 2007;151. doi:10.1007/s00442-006-0614-x

74. Van Steenis CGGJ. Rheophytes of the World. 1st ed. Springer Dordrecht; 1981.

75. Givnish T. On the Adaptive Significance of Leaf Form. In: Solbrig OT, Jain S, Johnson GB, Raven

PH, editors. Topics in Plant Population Biology. London: Macmillan; 1979.

76. Schuepp PH. Tansley Review No. 59 Leaf boundary layers. New Phytol. 1993;125. doi:10.1111/j.1469-8137.1993.tb03898.x

77. Vogel S. Convective cooling at low airspeeds and the shapes of broad leaves. J Exp Bot. 1970;21. doi:10.1093/jxb/21.1.91

78. Nobel PS. Physicochemical and Environmental Plant Physiology. 5th ed. Cambridge, UK: Academic Press; 2020. doi:10.1016/C2018-0-04662-9

79. Rodriguez- Iturbe I, Rinaldo A, Rigon R, Bras RL, Ijjasz- Vasquez E, Marani A. Fractal structures as least energy patterns: The case of river networks. Geophys Res Lett. 1992;19: 889–892. doi:10.1029/92GL00938

80. Rinaldo A, Banavar JR, Maritan A. Trees, networks, and hydrology. Water Resour Res. 2006;42. doi:10.1029/2005WR004108

81. Tian M, Yu G, He N, Hou J. Leaf morphological and anatomical traits from tropical to temperate coniferous forests: Mechanisms and influencing factors. Sci Rep. 2016;6. doi:10.1038/srep19703

82. Liu Y, Xiang H, Huang Z, Xiang X, Yu Y, Wang M, et al. Analysis of leaf-architecture characteristics and ecological adaptability of tree species in the upper reaches of the Chishui River. Ecol Indic. 2022;135. doi:10.1016/j.ecolind.2022.108563

83. Falster DS, Westoby M. Leaf size and angle vary widely across species: What consequences for light interception? New Phytol. 2003;158. doi:10.1046/j.1469-8137.2003.00765.x

 Click here to access/download;Figure;Fig 1.tif

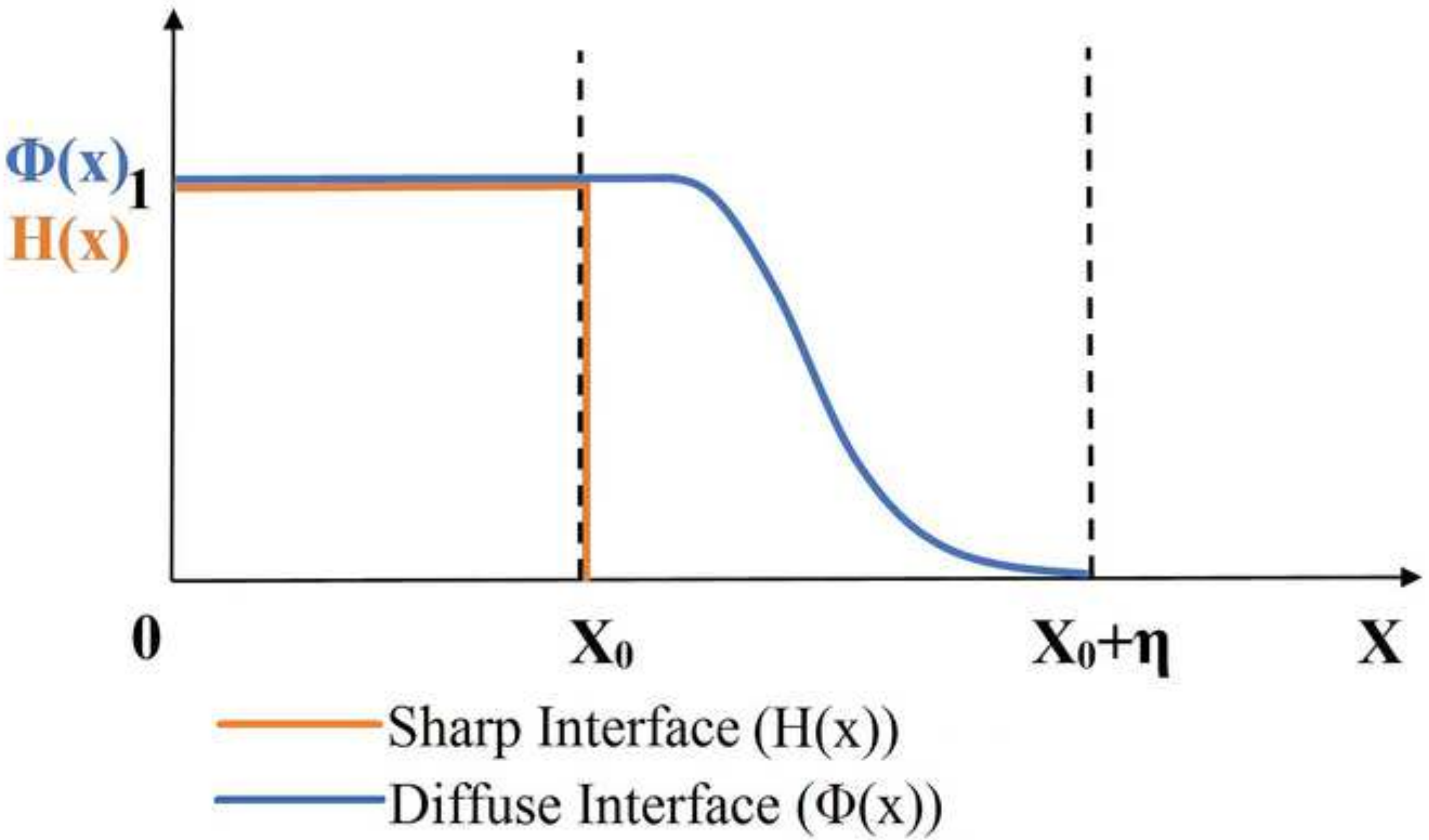

Figure Click here to access/download;Figure;Fig 2.tif

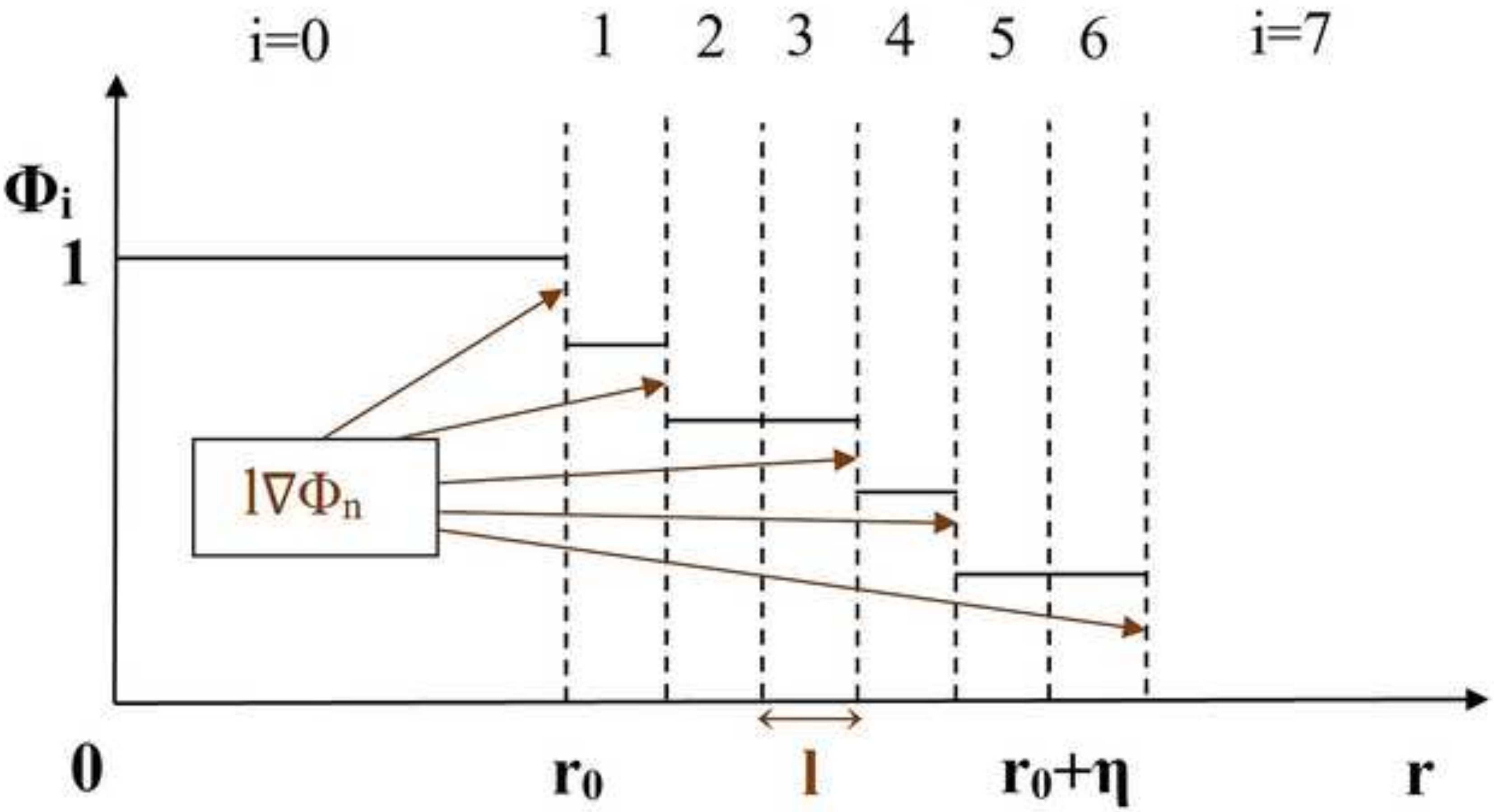

Click here to access/download;Figure;Fig 3.tif

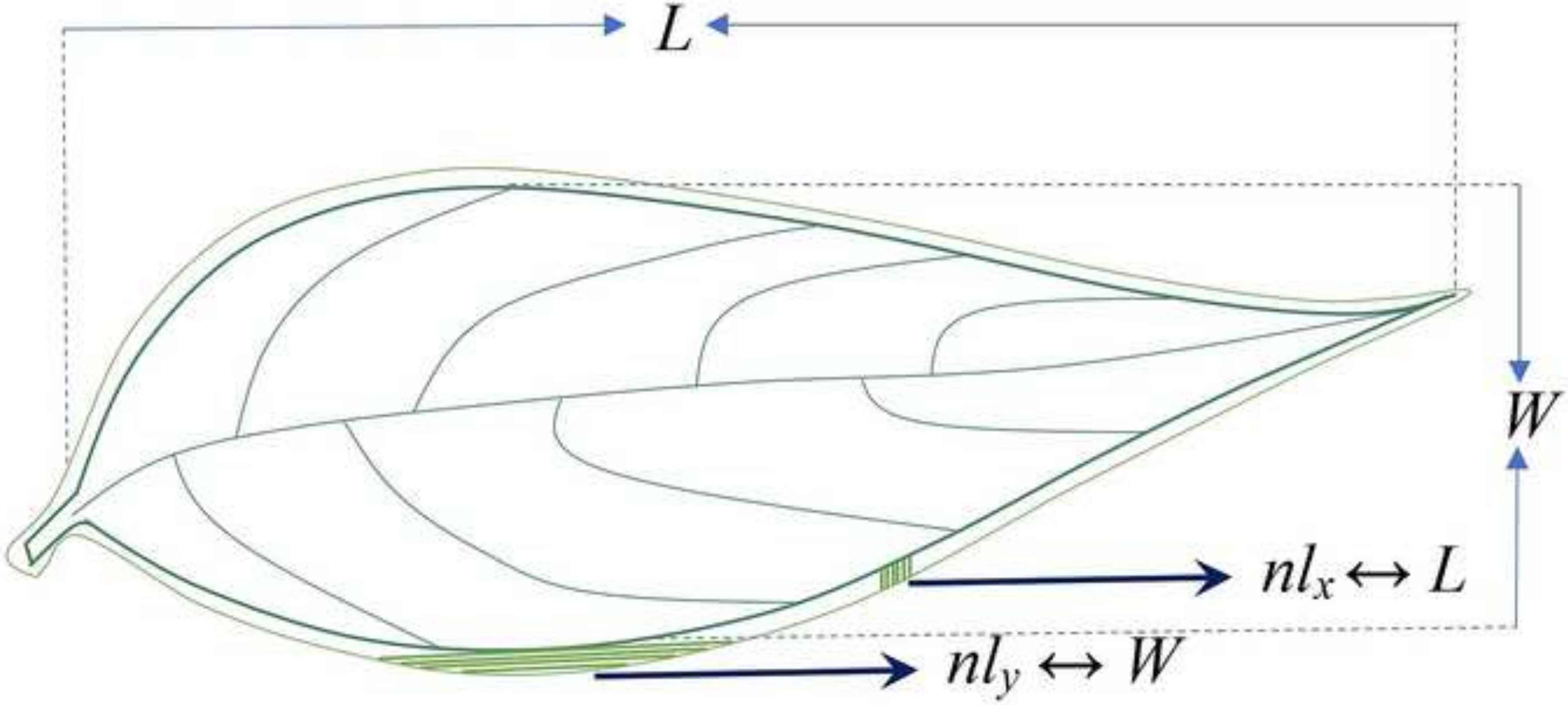

Click here to access/download;Figure;Fig 4.tif

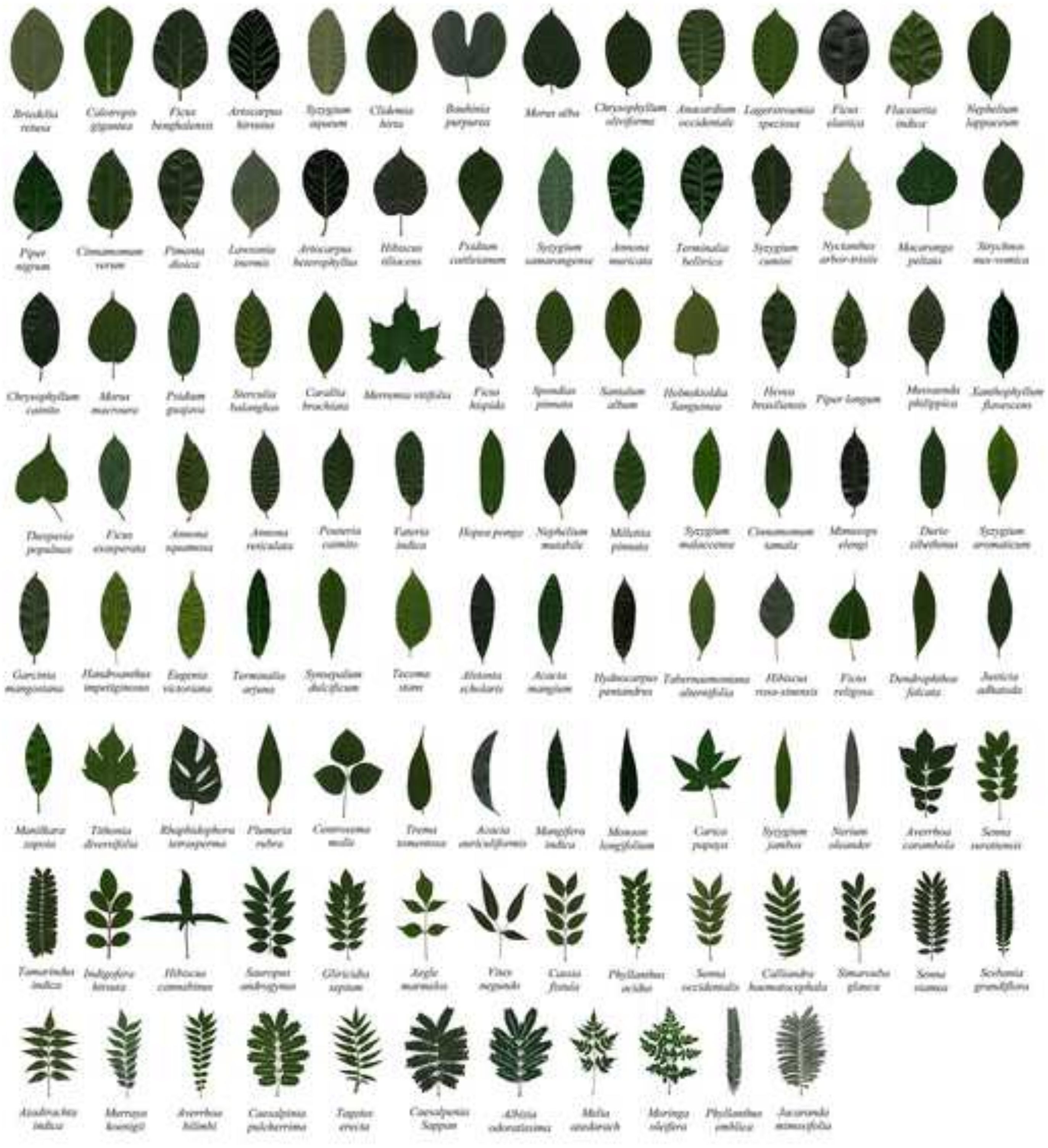

Figure

Click here to access/download;Figure;Fig 5.tif

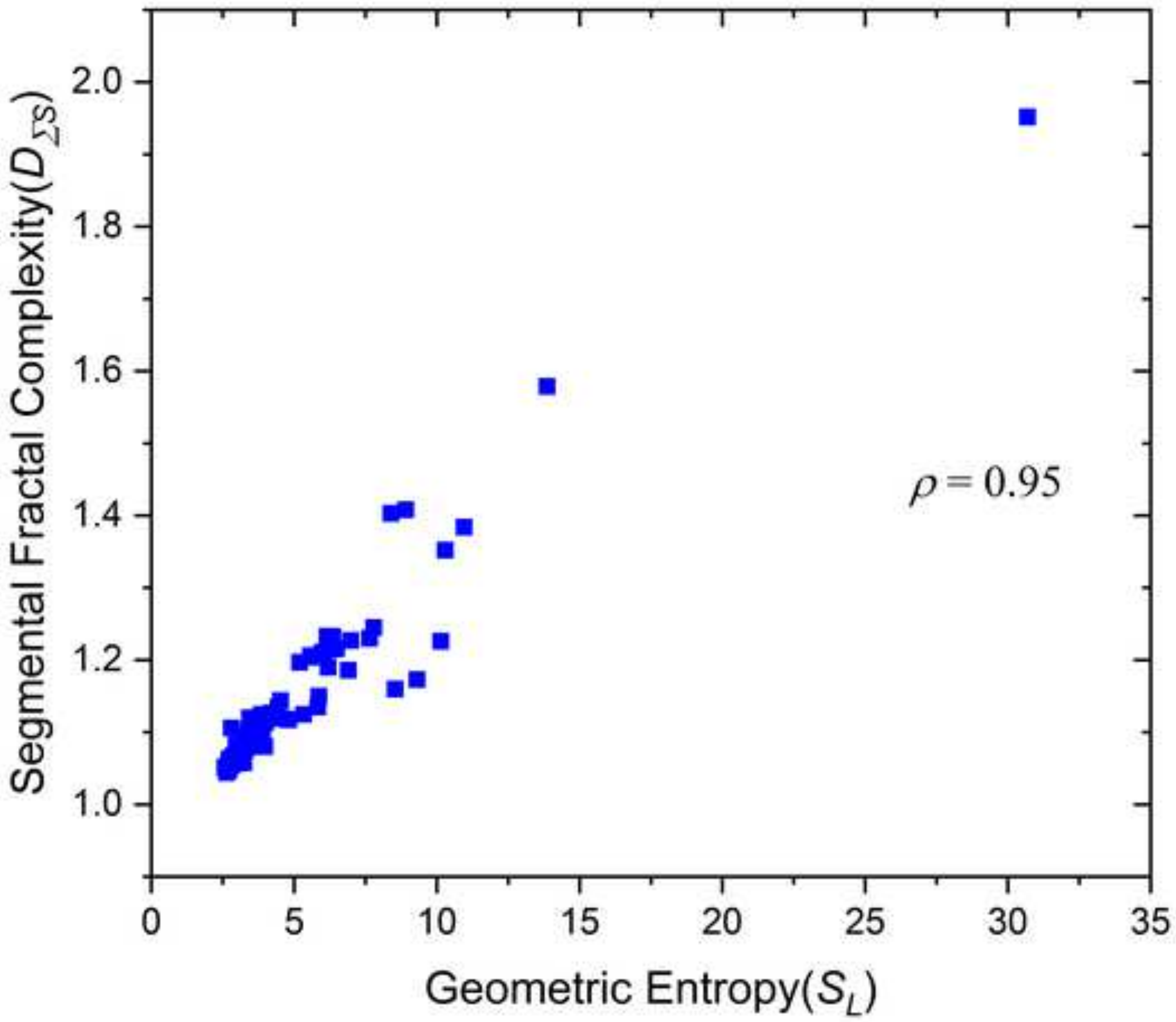

Figure

Click here to access/download;Figure;Fig 6.tif

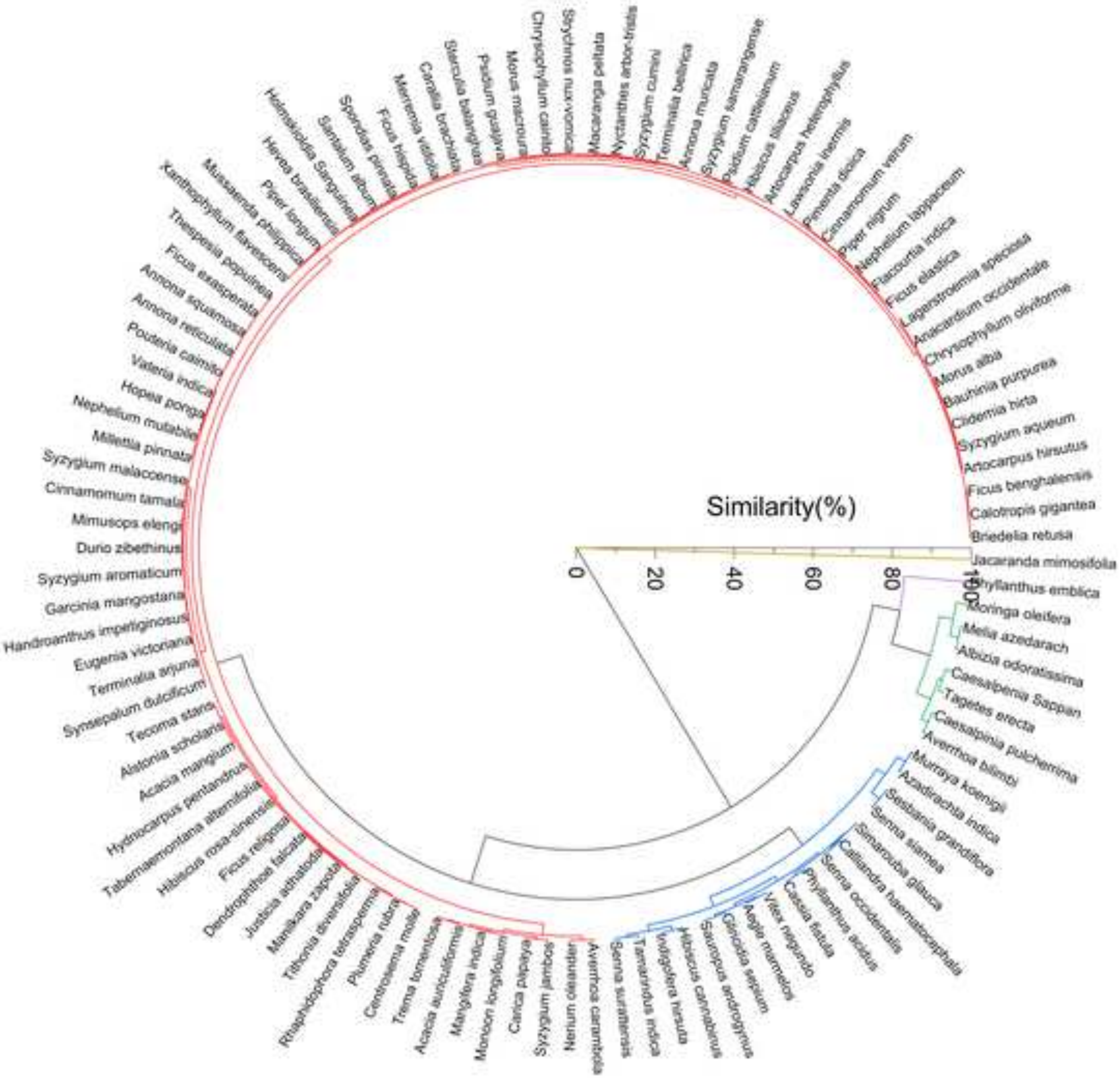

Supplement: Attachment — Submitted filename: PONE-D-23-07968_reviewer (1).pdf [file pone.0293596.s003.pdf]
