# Supplementary material for: Geometric entropy of plant leaves: A measure of morphological complexity
Source: PLoS One. 2024 Jan 2;19(1):e0293596. doi: 10.1371/journal.pone.0293596 (PMC10760904; doi:10.1371/journal.pone.0293596)
Supplement: Attachment — Submitted filename: Response to Reviewers.docx [file pone.0293596.s002.docx]

**REVIEWER REPORT(S):**

Kindly note: Our response to the reviewer’s comments is solely based on the email received. It did not have any attachments.

**COMMENTS TO THE AUTHOR(S)**

**Reviewer: 1**

The paper presents a mathematical framework to estimate the geometric entropy of plant leaves. The geometric entropy contains information on leaf geometry, represented by two physiologically important traits, leaf perimeter and leaf area. However the benefit and the application from the new measure are not well explaine and not justified

Thank you very much for taking the time to review our manuscript and for your insightful comments.

We added four more plant leaves into the dataset and accommodated them in a single plate (Fig 4). We reanalysed the work with 112 leaves, which did not affect/alter the result or conclusion.

A detailed explanation of the benefits and application of geometric entropy measures with justification by references was included in the discussion (Page 31. line 433-467).

A section justifying the assumptions and scientific derivation of the geometric entropy of plant leaves (*S_L_*) from geometric entropy of circular objects is replaced from ‘Discussion’ into ‘Geometric entropy of plant leaf’ (Page 11. line 192-203).

All the changes were incorporated in the manuscript under tracked changes.

**Additional Editor Comments:**

The reviewer is critical with your manuscript. We give you a chance to revise it.

Please reply satisfactorily to the reviewer, in particular explain and justify the benefit arising from these measures and their application(s).

Thank you for offering the chance to revise the manuscript.

We have accounted for the suggestions by the reviewer. A detailed explanation of the benefits and application of geometric entropy measures with justification by references was included in the discussion (Page 31. line 433-467).

A section justifying the assumptions and scientific derivation of the geometric entropy of plant leaves (*S_L_*) from geometric entropy of circular objects is replaced from ‘Discussion’ into ‘Geometric entropy of plant leaf’ (Page 11. line 192-203).

Further, we reanalyzed the work by including four additional plant leaves, which did not affect/alter the result or conclusion. All the changes were incorporated in the manuscript under tracked changes.
