# Supplementary material for: Geometric entropy of plant leaves: A measure of morphological complexity
Source: PLoS One. 2024 Jan 2;19(1):e0293596. doi: 10.1371/journal.pone.0293596 (PMC10760904; doi:10.1371/journal.pone.0293596)
Supplement: Attachment — Submitted filename: Response to Reviewers.docx [file pone.0293596.s004.docx]

**REVIEWER REPORT(S):**

**COMMENTS TO THE AUTHOR(S)**

**Journal Requirements:**

Please review your reference list to ensure that it is complete and correct. If you have cited papers that have been retracted, please include the rationale for doing so in the manuscript text, or remove these references and replace them with relevant current references. Any changes to the reference list should be mentioned in the rebuttal letter that accompanies your revised manuscript. If you need to cite a retracted article, indicate the article's retracted status in the References list and also include a citation and full reference for the retraction notice.

We have reviewed all the references. The reference list is complete and correct. We have not cited any papers that have been retracted.

**Reviewer: 2**

All comments have been addressed.

Thank you very much for taking the time to review our manuscript and your positive feedback.

The work is simple, but relevant as it can provide a parameter for understanding physiological processes that involve the leaf surface, such as gas exchange and heat.

Thank you very much for taking the time to review our manuscript and for your insightful comments. We have added a few supporting lines in the discussion (page: 32, line: 444-451) to improve the flow and clarity.

All the changes were incorporated in the manuscript under tracked changes.

**Additional Editor Comments:**

Please see the reviewer's attached file.

We have considered the reviewer's attached file and accounted for the suggestions by the reviewer (page:20-21, line: 249-252).

All the changes were incorporated in the manuscript under tracked changes.
